# Alkoxysulfonium Salts Unlock Access to New Aryl Sulfonium Salts for Cross‐Coupling

**DOI:** 10.1002/anie.202525298

**Published:** 2026-03-17

**Authors:** Rachel E. Brown, Navpreet Kaur, Anne‐Chloe M. A. Nassoy, Ciro Romano, David J. Procter

**Affiliations:** ^1^ Department of Chemistry The University of Manchester Manchester UK; ^2^ Oncology Targeted Discovery AstraZeneca UK Limited Cambridge UK

**Keywords:** cross‐coupling, Grignard, ligand‐coupling, photochemistry, sulfonium salt

## Abstract

Aryl sulfonium salts are indispensable partners in cross‐coupling reactions, allowing access to aryl metal or aryl radical intermediates, and offering advantages over aryl halides or other pseudohalides. Routes to aryl sulfonium salts are limited; the standard approach involves activation of arenes using a sulfoxide‐derived electrophile. While the activation of non‐prefunctionalized arene partners is attractive, the method is limited to arenes that are sufficiently electron‐rich to engage with sulfur electrophiles and is often dominated by *para*‐selectivity when monosubstituted benzenes are used. This limited access to aryl sulfonium salts leads to biased substrate scopes and restrictions on accessible chemical space. We describe a route to otherwise hard‐to‐access (hetero)aryl sulfonium salts that exploits bench‐stable alkoxysulfonium salts in combination with the well‐established magnesiation of readily available aryl bromides. The approach allows access to aryl sulfonium salts bearing electron‐deficient aryl and heteroaryl rings, or electron‐rich systems in which the sulfonium motif resides at an alternative position on the ring to that delivered by electrophilic arene sulfenylation. To illustrate their value, the new sulfonium salts have been used to expand the scope of a selection of coupling processes.

## Graphical abstract

Hard‐to‐access (hetero)aryl sulfonium salts are prepared using stable methoxysulfonium salts in combination with the magnesiation of (hetero)aryl bromides. The approach allows access to aryl sulfonium salts bearing electron‐deficient (hetero)aryl rings, or electron‐rich aryl motifs in which the sulfonium motif resides at an alternative position on the ring to that delivered by electrophilic sulfenylation. The new sulfonium salts extend the scope of photocatalytic, photochemical, and ligand‐coupling processes.

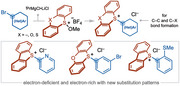

## 1 Introduction

**SCHEME 1:**
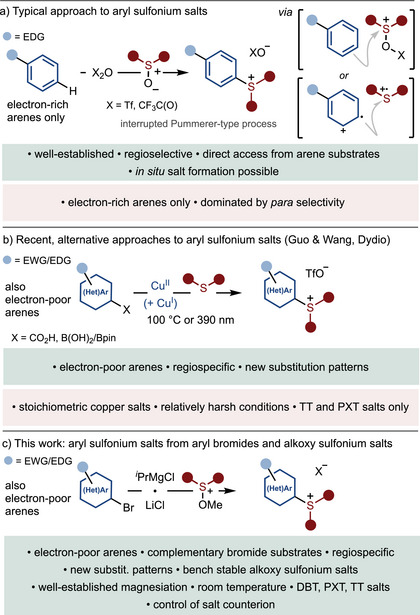
(a) Arylsulfonium salts from non‐prefunctionalized electron‐rich arenes using in situ activated sulfoxides. (b) Recent alternative syntheses of arylsulfonium salts from electron‐poor and electron‐rich (hetero)aryl boronic and carboxylic acids. (c) A complementary route to aryl sulfonium salts from electron‐poor and electron‐rich (hetero)aryl bromides using alkoxy sulfonium salts. Tf, 1,1,1‐trifluoromethanesulfonyl; DBT, dibenzothiophenium; TT, thianthrenium; PXT, phenoxathiinium.

The emergence of aryl sulfonium salts as electrophilic partners for cross‐coupling has allowed new chemical transformations to be discovered and known approaches to be expanded [1, 2, 3, 4, 5, 6, 7, 8, 9]. This includes key roles as enabling substrates in metal‐catalyzed cross‐couplings [10, 11, 12, 13, 14, 15, 16, 17, 18, 19, 20, 21, 22, 23], photocatalytic processes [24, 25, 26, 27, 28, 29, 30, 31, 32], photochemical transformations [33, 34, 35, 36, 37, 38, 39, 40, 41, 42, 43, 44], metallo‐photoredox chemistry [45, 46, 47, 48, 49], and ligand couplings [50, 51, 52, 53, 54, 55, 56, 57, 58, 59, 60]. The bench‐stability and crystallinity of aryl sulfonium salts, combined with their ability to furnish organometallic, radical, and sulfurane intermediates under mild conditions, make them versatile partners and ensure they feature heavily in contemporary methods. However, it is in the preparation of aryl sulfonium salts that significant challenges remain [1, 2, 3, 4, 5, 6, 7, 8, 9]; the most common method of preparation involves the reaction of arenes with a sulfoxide (typically activated using Tf_2_O or TFAA) in an interrupted Pummerer‐type process [61, 62, 63, 64, 65]. While the activation of non‐prefunctionalized arene partners is attractive in that it delivers formal C─H‐type coupling processes, this route to aryl sulfonium salts is inherently limited to arenes that are sufficiently electron‐rich to engage with sulfur electrophiles and such sulfenylation of monosubstituted arenes is often dominated by para‐selectivity; alternative *ortho* functionalization is challenging when the *para*‐position is free (Scheme 1a) [10, 27, 66, 67]. This leads to biased substrate scopes and restrictions on accessible chemical space [1, 2, 3, 4, 5, 6, 7, 8, 9]. Electron‐deficient (hetero)arenes are privileged motifs in medicinal chemistry; their incorporation often increases activity relative to their phenyl counterparts, and they are less rapidly oxidised in the body to inactive forms [68, 69, 70, 71]. Therefore, it is crucial that new cross‐coupling processes can incorporate such motifs, and thus, methods that deliver aryl sulfonium salts bearing electron‐deficient (hetero)aryl groups are vital.

In response to this challenge, the team of Wang and Guo developed a Cu(II)‐mediated synthesis of new arylsulfonium salts from arylboronic acids and thianthrene/phenoxathiin (Scheme 1b) [72]. A related Cu(II)/Cu(I)‐mediated approach was reported by Dydio and co‐workers using thianthrene and aryl carboxylic acids (Scheme 1b) [73]. These two promising approaches deliver aryl sulfonium salts bearing electron‐deficient (hetero)aryl rings, or electron‐rich systems in which the sulfonium motif resides at an alternative position on the ring to that delivered by electrophilic arene sulfenylation. While the approaches have been used to prepare thianthrenium (TT) and phenoxathiinium (PXT) salts, the synthesis of commonly‐used dibenzothiophenium (DBT) salts was not described [72, 73]. Furthermore, limitations could arise when wanting to access aryl sulfonium salts from unstable boronic acids that are unstable. For example, 2‐pyridyl boron reagents are notoriously unstable [74, 75] and tend to react poorly in cross‐couplings [76]. Thus, despite the importance of the 2‐pyridyl group in pharmaceuticals, natural products, and metal‐complexing ligands [77, 78, 79, 80, 81], to our knowledge, formation of 2‐pyridyl sulfonium salts by pyridine‐sulfur bond formation has not previously been reported [55].

Here, we describe a complementary approach to hard‐to‐make sulfonium salts that exploits alkoxysulfonium salts and the established magnesiation chemistry of readily‐available (hetero)aryl bromides (Scheme 1c). The approach allows regiospecific access to new electron‐deficient (hetero)aryl sulfonium salts and electron‐rich aryl sulfonium salts in which the sulfonium motif resides at alternative positions on the ring to that delivered by electrophilic sulfenylation. The value of the new (hetero)aryl sulfonium salts is illustrated through their use in expanding the scope of a selection of recent literature transformations.

## 2 Results and Discussion

**SCHEME 2:**
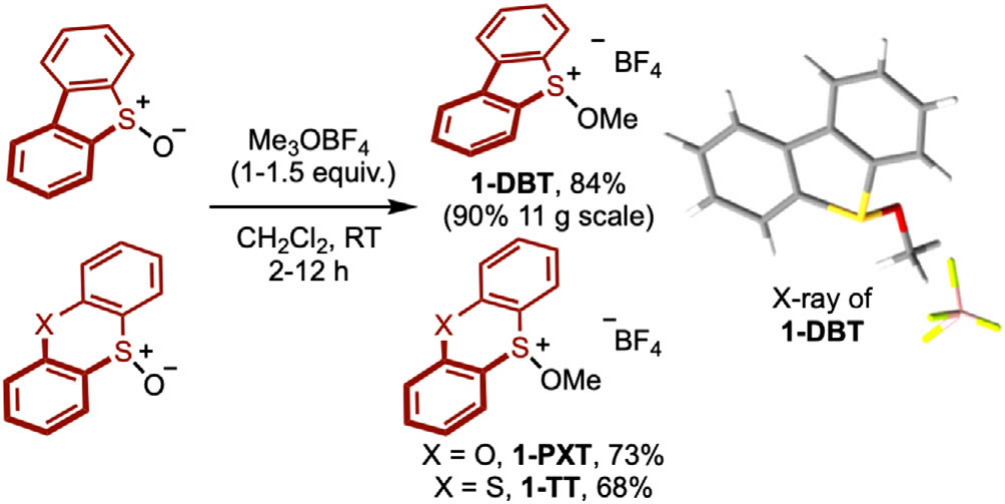
Formation of methoxy sulfonium salts. Reaction conditions: S‐oxides (1.0 equiv.), CH_2_Cl_2_ (0.2 M), Me_3_OBF_4_ (1.0 to 1.5 equiv.), 2 or 12 h, RT. RT, room temperature.

DBT, PXT, and TT methoxy sulfonium tetrafluoroborate salts **1‐DBT**, **1‐PXT**, and **1‐TT** were prepared in 84%, 73%, and 68% yield, respectively, from the corresponding starting sulfoxides using trimethyloxonium tetrafluoroborate (Meerwein's salt) [82, 83, 84, 85]. Moving to a larger scale, **1‐DBT** was prepared in 90% yield on an 11 g scale. The structure of **1‐DBT** was confirmed by X‐ray crystallographic analysis (Scheme 2) [86]. Alternatively, methoxy sulfonium salts and other alkoxysulfonium salts can be formed by activation of sulfoxides using trifluoromethanesulfonic anhydride, followed by reaction with methanol, in an interrupted Pummerer process [85]. Using this approach, the corresponding triflate alkoxysulfonium salts were obtained. Stability studies revealed that all methoxy sulfonium salts were bench stable for at least one month, and **1‐DBT** was stable for at least 1 year in the freezer under air.

**SCHEME 3:**
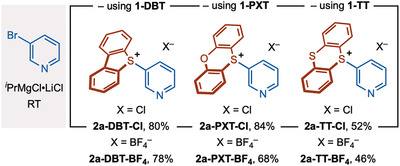
A convenient route to new aryl sulfonium salts. Magnesiation of 3‐bromopyridine and addition to methoxy sulfonium salts give 3‐pyridyl DBT, PXT, and TT sulfonium chloride salts. An alternative workup gives the analogous 3‐pyridyl DBT, PXT, and TT sulfonium tetrafluoroborate salts.

Combining the use of methoxy sulfonium salts **1** with established modern methods for selective magnesiation [87, 88, 89, 90, 91], we sought to prepare hard‐to‐access electron‐deficient aryl sulfonium salts and electron‐rich aryl sulfonium salts with different substitution patterns. In early studies on the chemistry of sulfuranes, a limited number of simple aryl sulfonium salts were prepared by the addition of aryl Grignard reagents to activated S‐oxides; however, an excess of unfunctionalized aryl Grignard reagent and high temperature were employed, and low yields of salt were obtained [92, 93]. In addition, isolated reports describe the addition of unfunctionalized aryl Grignards to a methoxysulfonium salt to prepare simple aryl sulfonium salts [83, 84]. Inspired by these early reports, we set about developing an approach for use in contemporary synthesis. Magnesiation of 3‐bromopyridine using iPrMgCl•LiCl [87, 88, 89, 90, 91] and coupling of the resultant Grignard with **1‐DBT** gave 3‐pyridyl sulfonium salt **2a‐DBT‐Cl** in 80% isolated yield (Scheme 3). The process could be run on a gram scale, delivering **2a‐DBT‐Cl** in 77% yield. Chloride was identified as the major counterion (97:3; BF_4_^–^ minor counterion) by elemental analysis and quantitative fluorine NMR studies (see Supporting Information). Finally, the structure of **2a‐DBT‐Cl** was confirmed by x‐ray crystallographic analysis [86]. By varying the methoxysulfonium salt, **2a‐PXT‐Cl** and **2a‐TT‐Cl** could similarly be prepared in 84% and 52% yield, respectively.

The lower yield for the formation of **2a‐TT‐Cl** appears to arise from the lower electrophilicity of sulfur in the thianthrenium system, which leads to the formation of byproducts thianthrene S‐oxide and thianthrene. Employing the same reaction conditions, but adding NaBF_4_ in the workup procedure, gave the analogous tetrafluoroborate salts, **2a‐DBT‐BF**_**4**_, **2a‐PXT‐BF**_**4**_, and **2a‐TT‐BF**_**4**_, in 78%, 68%, and 46% yield, respectively. Thus, the counterion of the sulfonium salts can be controlled (Scheme 3). Swapping the Cl counterion for BF_4_ typically improved the solubility of aryl sulfonium salts in organic solvents. Transmetallation of the 3‐pyridyl magnesium to the corresponding heteroaryl zinc and coupling with **1‐DBT** gave **2a‐DBT‐Cl** in 30% yield, showing the potential for also exploiting aryl zincs—and their complementary properties—in our approach. Given the importance of DBT aryl sulfonium salts, and their absence from previous protocols for the synthesis of new aryl sulfonium salts [1, 2, 3, 4, 5, 6, 7, 8, 9], **1‐DBT** was selected to explore the scope of (hetero)aryl sulfonium salt formation, although it is important to note that the corresponding aryl PXT/TT sulfonium salts should also be available by a simple switch to the use of **1‐PXT**/**1‐TT** (vide supra).

**SCHEME 4:**
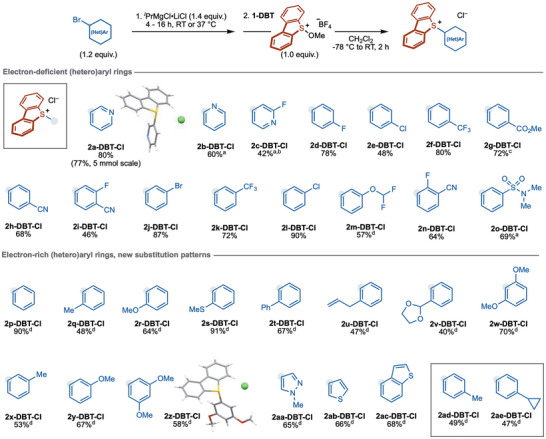
Scope of aryl sulfonium salt formation from (hetero)aryl bromides and **1‐DBT**. ^a^ **1‐DBT** (1.0 equiv.) was dissolved in CH_2_Cl_2_:THF (1:1) (0.1 M). ^b^The resulting solution was stirred at −78°C to −50°C for 20 h. ^c^Prepared from methyl 4‐iodobenzoate. Magnesiation was carried out at −40°C for 2 h. ^d^Magnesiation was carried out at 37°C. Reaction conditions: ^i^PrMgCl•LiCl (1.4 equiv.) was added to neat bromoarene (1.2 equiv.), **1‐DBT** (1.0 equiv.) was dissolved in CH_2_Cl_2_ (0.1 M), and the preformed aryl Grignard solution was added at −78°C. The resulting solution was stirred at −78°C for 10 min before the cold bath was removed and the reaction allowed to warm and stirred for a further 2 h.

A range of aryl sulfonium salts bearing electron‐deficient (hetero)aryl groups was prepared. (Scheme 4) 3‐Pyridyl (**2a‐DBT‐Cl**), 2‐pyridyl (**2b‐DBT‐Cl**), and 4‐pyridyl (**2c‐DBT‐Cl**) sulfonium salts were prepared in moderate to good yield. Of note, 2‐pyridyl sulfonium salt **2b‐DBT‐Cl** was found to be stable under acidic conditions that result in protodeborylation of 2‐pyridylboronic acid [74, 75, 76], thus highlighting the value of **2b‐DBT‐Cl** as a complementary reagent for installation of the important 2‐pyridyl motif (see Supporting Information). Aryl sulfonium salts bearing para electron‐withdrawing groups, including fluoro (**2d‐DBT‐Cl**), chloro (**2e‐DBT‐Cl**), trifluoromethyl (**2f‐DBT‐Cl**), carbomethoxy (**2g‐DBT‐Cl**), and cyano (**2h‐DBT‐Cl** and **2i‐DBT‐Cl**), were obtained in 46%–80% yield. Aryl sulfonium salts bearing meta electron‐withdrawing groups, including fluoro (**2i‐DBT‐Cl**), bromo (**2j‐DBT‐Cl**), trifluoromethyl (**2k‐DBT‐Cl**), chloro (**2l‐DBT‐Cl**), difluoromethoxy (**2m‐DBT‐Cl**), cyano (**2n‐DBT‐Cl**), and sulfonamido (**2o‐DBT‐Cl**), were isolated in 46%–90% yield. The preparation of sulfonium salt **2n‐DBT‐Cl** in 64% yield shows that the presence of an ortho fluoro substituent is tolerated. Of note, **2j‐DBT‐Cl** was efficiently prepared from 1,3‐dibromobenzene by selective mono‐magnesiation.

We next turned to the preparation of aryl sulfonium salts bearing electron‐rich (hetero)aryl groups, in which the sulfonium motif is installed at an alternative position on the ring to that delivered by electrophilic sulfenylation. The magnesiation of electron‐rich aryl bromides proceeded more efficiently at 37°C, rather than RT, under otherwise standard conditions. In addition to simple phenyl dibenzothiophenium salt **2p‐DBT‐Cl**, three tolyl sulfonium salts could be prepared regiospecifically from the corresponding aryl bromides. The synthesis of tolyl sulfonium salts from toluene by electrophilic sulfenylation with sulfoxides can lead to mixtures of regioisomers [66], although it is known to be highly para‐selective when using thianthrene S‐oxide [66]. Using our magnesiation/methoxy sulfonium salt approach, 2‐tolyl (**2q‐DBT‐Cl**), 3‐tolyl (**2x‐DBT‐Cl**), and 4‐tolyl (**2ad‐DBT‐Cl**) sulfonium salts were prepared in isolated yields of 48%–53%. While aryl sulfonium salt formation using mono‐substituted benzenes in electrophilic sulfenylation approaches is typically dominated by para selectivity, regioisomeric by‐products can result that are hard to remove. The magnesiation/methoxy sulfonium salt approach from aryl bromides provides a complementary regiospecific approach to para‐substituted aryl sulfonium salts (**2ad‐DBT‐Cl** and **2ae‐DBT‐Cl**). Aryl sulfonium salts bearing ortho electron‐donating groups, including methoxy (**2r‐DBT‐Cl**), thiomethyl (**2s‐DBT‐Cl**), phenyl (**2t‐DBT‐Cl**), allyl (**2u‐DBT‐Cl**), and 1,3‐dioxolyl (**2v‐DBT‐Cl**), were isolated in 40%–91% yield. Of note, aryl sulfonium **2w‐DBT‐Cl**, bearing two ortho methoxy groups, was prepared in 70% yield despite the significant steric encumbrance involved. Aryl sulfonium salts bearing meta electron‐donating groups, including methyl (**2x‐DBT‐Cl**) and methoxy (**2y‐DBT‐Cl**), were also prepared in moderate isolated yield. Aryl sulfonium **2z‐DBT‐Cl**, bearing two meta methoxy groups, was prepared in 58% yield and its structure confirmed by X‐ray crystallographic analysis [86]. Finally, sulfonium salts bearing electron‐rich 4‐pyrazolyl (**2aa‐DBT‐Cl**), 3‐thienyl (**2ab‐DBT‐Cl**), and 4‐benzothienyl (**2ac‐DBT‐Cl**) units were isolated in 65%–68% yield, respectively.

**SCHEME 5:**
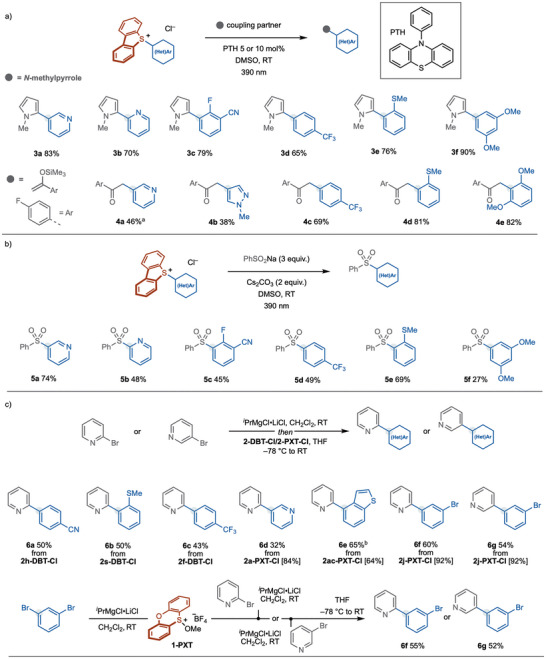
Application of new aryl sulfonium salts. (a) Photocatalytic C─H arylation and coupling with a silyl enol ether. (b) Photochemical sulfonylation using an EDA complex formation/photoactivation approach. (c) Preparation of heterobiaryls by a ligand‐coupling approach. Yields in square brackets are those obtained for the preparation of the indicated PXT salts using **1‐PXT**. ^a^0.2 M, 2,6‐lutidine (1.5 equiv.); ^b^3 equiv. of 2‐pyridyl Grignard was used. DMSO, dimethylsufoxide; THF, tetrahydrofuran.

With access to several new aryl sulfonium salts, their ability to expand the scope of photocatalytic, photochemical, and ligand‐coupling processes was investigated (Scheme 5). First, we used a representative selection of (hetero)aryl sulfonium salts in cross‐couplings with *N*‐methyl pyrrole under photocatalytic conditions using the organic photocatalyst 10‐phenylphenothiazine (PTH) (10 mol%) (Scheme 5a) [27, 94]. Under the conditions previously developed in our group, the 3‐ and 2‐pyridyl heterobiaryl products **3a** and **3b** were isolated in 83% and 70% yield. Sulfonium salts bearing electron‐deficient aryl groups underwent coupling with *N*‐methyl pyrrole to give **3c** and **3d** in 79% and 65% yield, respectively. Switching to the use of sulfonium salts bearing electron‐rich aryl groups with new substitution patterns, thioanisole and anisole products **3e** and **3f** were isolated in 76% and 90% yield. These photocatalytic couplings were not efficient when using analogous heteroaryl bromides [95, 96, 97, 98, 99, 100]. Using a silyl enol ether trap [32, 43], the photocatalytic coupling, using PTH (5 mol%), of a selection of (hetero)aryl sulfonium salts, gave ketone products **4a**‐**4e** in moderate to good yield (Scheme 5a).

Recent studies have exploited arylsulfonium salts in electron‐donor‐acceptor (EDA) complex formation and photoactivation [3]. Adopting the conditions reported by Molander [40] in which sodium phenyl sulfinate acts as a donor in EDA complex formation, we subjected a panel of new (hetero)aryl sulfonium salts to photochemical sulfonylation (Scheme 5b). 3‐Pyridyl and 2‐pyridyl sulfonium salts underwent sulfonylation to give **5a** and **5b** in 74% and 48% yield, respectively. Of note, 2‐pyridyl sulfonium salt **2b‐DBT‐Cl** underwent significant background sulfonylation in the absence of light, presumably through a S_N_Ar process (38% yield of **5b**). In contrast, sulfonylation of the analogous 3‐pyridyl and 4‐trifluoromethylphenyl sulfonium salts, **2a‐DBT‐Cl** and **2f‐DBT‐Cl**, did not proceed without light. Sulfonylation of arylsulfonium salts bearing electron‐withdrawing groups, such as cyano, fluoro and trifluoromethyl, proceeded to give **5c** and **5d** in moderate yields. Sulfonylation of arylsulfonium salts bearing electron‐donating substituents, at unusual positions relative to the sulfonium group, gave **5e** and **5f** in 69% and 27% yield, respectively.

Moving away from radical chemistry, we explored the use of the new (hetero)aryl sulfonium salts in ligand‐coupling processes involving sulfurane intermediates, as popularized by Qin [60, 101] and McGarrigle [55, 57] (Scheme 5c). A range of DBT salts and PXT salts underwent cross‐coupling upon treatment with 2‐pyridyl and 3‐pyridyl magnesiums to give heterobiaryls **6a‐g**, containing cyano, methylsulfanyl, trifluoromethyl, benzothienyl, and bromo functionalities. In general, PXT salts were found to give higher selectivity for the desired ligand‐coupling. This could be attributed to the greater electron‐density of the PXT backbone reducing its ability to accommodate negative charge build‐up, and thus, providing higher site‐selectivity during ligand‐coupling. In addition, the greater conformational flexibility of PXT salts, compared to DBT‐salts, likely allows for more facile pseudo‐rotation of the sulfurane intermediate to adopt the required conformation for productive ligand‐ligand coupling [55, 57]. Our approach to new (hetero)aryl sulfonium salts was also compatible with a one‐pot, telescoped process; magnesiation of 1,3‐dibromobenzene, quenching with **1‐PXT**, followed by addition of 2‐pyridyl or 3‐pyridyl magnesiums, gave **6f** and **6g** in 55% and 52% overall yield, respectively (Scheme 5c).

Finally, although we have chosen to focus on transition metal‐free processes, in a preliminary study, we have shown that 3‐pyridyl sulfonium salt **2a‐DBT‐Cl** undergoes palladium‐catalyzed cross‐coupling with 4‐trifluoromethylphenyl boronic acid (see Supporting Information) [65].

## 3 Conclusion

In summary, a route to otherwise hard‐to‐access (hetero)aryl sulfonium salts exploits bench‐stable alkoxysulfonium salts in combination with the well‐established magnesiation of readily available (hetero)aryl bromides. The approach allows access to aryl sulfonium salts bearing electron‐deficient aryl and heteroaryl rings, or electron‐rich aryl motifs in which the sulfonium motif resides at an alternative position on the ring to that delivered by electrophilic arene sulfenylation. The synthetic value of the new sulfonium salts has been showcased through their use in reported photocatalytic, photochemical, and ligand‐coupling processes, significantly extending their scope.

## Conflicts of Interest

The authors declare no conflicts of interest.

## Supporting information



The authors have cited additional references within the Supporting Information. Crystallographic data for compounds **1‐DBT**, **2a‐DBT‐Cl**, and **2z‐DBT‐Cl** have been deposited with the Cambridge Crystallographic Data Centre, with deposition numbers CCDC 2492905, 2492906, and 2492916, respectively.

## Data Availability

The data that support the findings of this study are available in the supplementary material of this article.
