# Supplementary material for: Alkoxysulfonium Salts Unlock Access to New Aryl Sulfonium Salts for Cross‐Coupling
Source: Angew Chem Int Ed Engl. 2026 Mar 17;65(17):e25298. doi: 10.1002/anie.202525298 (PMC13098308; doi:10.1002/anie.202525298)
Supplement: Supplementary file 1 — The authors have cited additional references within the Supporting Information. Crystallographic data for compounds 1‐DBT, 2a‐DBT‐Cl, and 2z‐DBT‐Cl have been deposited with the Cambridge Crystallographic Data Centre, with deposition numbers CCDC 2492905, 2492906, and 2492916, respectively. [file ANIE-65-e25298-s001.pdf]

Supporting Information

Alkoxysulfonium Salts Unlock Access to New Aryl Sulfonium Salts for Cross-Coupling

Rachel E. Brown[a], Navpreet Kaur[a], Anne-Chloe M. A. Nassoy[b], Ciro Romano[a], David J. Procter*[a]

[a] – Department of Chemistry, The University of Manchester, Oxford Road, Manchester, M13 9PL (UK).

[b] – AstraZeneca UK Limited, Francis Crick Avenue, Cambridge Biomedical Campus, Cambridge, CB2 0AA

E-mail: david.j.procter@manchester.ac.uk

Table of Contents

1. General Information 4

2. Experimental Procedures 5

2.1 General procedure for aryl sulfonium salt formation using a methoxy sulfonium salt – GP1……....5

2.2 General procedure for coupling with *N*-methyl pyrrole – GP2……...5

2.3 General procedure for alkylation using silyl enol ether– GP3……………………………………….....6

2.4 General procedure for sulfonylation – GP4……………..…………………………………………….....6

2.5 General procedure for ligand coupling – GP5…………………………...……………………….......7

3. Methoxy sulfonium salt stability studies 8

4. Studies into salt purity and counterion……………………………………………...…19

5. Stability studies on 2b-DBT-Cl…………………………………………………..………27

6. Synthesis of S-oxides……………………………………………………………….……27

7. Synthesis of Alkoxy Sulfonium Salts……………………………………….………….…29

8. Synthesis of Aryl Sulfonium Salts……….……….………………………………...…31

9. Proposed Mechanism for the Conversion of Methoxysulfonium salts 1 into (Hetero)Aryl Sulfonium Salts 2……………………………………………………………………….…...60

10.Cyclic Voltametry studies………………………………………………………………..…61

11. Control Experiments with a hetero(aryl)bromide and hetero(aryl) sulfonium salt…………………………………………...…...……...…………………………...…63

12. Application of the new (hetero)aryl sulfonium salts ……………..………………...….66

12.1 Scope for Arylation……….………………………………………………….………..…….66

12.2 Scope for Alkylation……………………………………………………………….….…….71

12.3 Scope for Sulfonylation………..……………………………………………………..….….75

12.4 Scope for Ligand Coupling…...………………………………………………….……..…….79

12.5 Preliminary study- a Pd-catalysed cross coupling……………………………….…………84

13. Telescoped Approach to Ligand Coupling ……….……………………..…………….….86

14. X-ray Crystal Structures………………..……….……..………………………………88

15. NMR Spectra……………………………………………………….………………...95

16. References……………………………………………………………..………………188

1. General Information

Experiments were performed under an atmosphere of nitrogen using anhydrous solvents and standard Schlenk manifold techniques, unless otherwise stated. THF was freshly distilled before use. THF was distilled over sodium wire and benzophenone. All other solvents and reagents used were purchased from commercial suppliers at 99.8% purity and used according to relevant guidelines. Photochemical reactions were subjected to irradiation from light-emitting diode (LED) lamps: Kessil PR 160 390 nm, Kessil PR 160 456 nm, and Kessil A160WE Tuna Blue, with the reaction tube placed approximately 2 cm from the bulb, and a fan used for cooling.

^{1}H NMR spectra were obtained at room temperature on a Bruker 400 or 500 MHz spectrometer. ^{13}C NMR spectra were obtained at 101 or 126 MHz, respectively. ^{19}F NMR spectra were obtained at 471 or 376 MHz. All NMR spectra were processed using Mestrenova© NMR software. Chemical shifts are reported in parts per million (ppm) downfield from trimethylsilane, relative to residual chloroform (δH = 7.27 and δC = 77.0), acetonitrile (δH = 1.94 and δC = 1.32) or DMSO (δH = 2.50 and δC = 39.5) as internal standards, and coupling constants (*J*) are reported in Hz. Splitting patterns are reported as follows: singlet (s), doublet (d), triplet (t), quartet (q), heptet (hept), broad singlet (brs), broad doublet (brd), broad triplet (brt), double of doublets (dd), doublet of triplets (dt), doublet of quartets (dq), triplet of triplets (tt), quartet of doublets (qd), doublet of doublets of doublets (ddd), doublet of doublets of triplets (ddt), doublet of triplets of doublets (dtd), doublet of quartets of doublets (dqd), triplet of doublets of doublets (tdd), doublet of doublets of doublets of doublets (dddd) and multiplet (m). Column chromatography was carried out using 35–70 μm, 60 Å silica gel. TLC analysis was carried out on aluminium sheets coated with silica gel 60 F254, 0.2 mm thickness and visualised using potassium permanganate, phosphomolybdic acid, or cerium ammonium molybdate solution and heating, and/or UV light at 254 nm. Mass spectra were obtained using

positive and negative electrospray (ESI±) or atmospheric pressure chemical ionisation (APCI) methodology. Infra-red spectra were recorded as evaporated films or neat using a FT/IR spectrometer and values are reported in cm^{-1}.

2. Experimental Procedures

2.1 General procedure for aryl sulfonium salt formation using a methoxy sulfonium salt – GP1

To an oven-dried vial containing bromoarene (1.2 equiv.) at RT was added 1.3 M isopropylmagnesium(II) chloride lithium chloride in THF (1.4 equiv.). The reaction was stirred for 5–18 hours at RT or 37 ˚C before use.

In a separate flask, a solution of methoxysulfonium salt (1.0 equiv.) in CH_2Cl_2 (0.1 M) was cooled to -78 °C, and the preformed aryl Grignard reagent (1.2 equiv.) was added. The reaction mixture was stirred at -78 °C for 10 min, then was warmed to RT and stirred for a further 2 h. After this time methanol was added to quench, then saturated aqueous NH_4Cl (50 mL) and a solution of 10% IPA in $CHCl_3$ (45 mL for a 1.0 mmol scale reaction) were also added and the layers separated. The aqueous layer was extracted with a solution of 10% IPA in $CHCl_3$ (3 x 45 mL), the combined organic layers were dried with Na_2SO_4 and the solvent was removed *in vacuo*. The crude product was purified by column chromatography on silica gel with CH_2Cl_2 to 9:1 CH_2Cl_2:MeOH as eluent.

2.2 General procedure for coupling with *N*-methyl pyrrole – GP2

Aryl sulfonium salt (0.2 mmol, 1.0 equiv.) and 10-phenyl-phenothiazine (10 mol%) were added to a reaction vial equipped with a magnetic stirring bar and the vial sealed with a crimp cap. Then, the vial was evacuated and backfilled with nitrogen three times. DMSO (0.5 mL) was added followed by *N*-methyl pyrrole (0.36 mL, 4.0 mmol, 20 equiv.), and the crimp cap was sealed with parafilm. The reaction mixture was irradiated with a Kessil PR 160 LED lamp (λ

centred at 390 nm, 100% irradiance) for 16 h with a cooling fan, before being quenched with saturated aqueous $NaHCO_3$ (10 mL) and diluted with EtOAc (10 mL). The aqueous layer was extracted with EtOAc (10 mL), then the combined organic layers were washed with brine (10 mL), dried using $MgSO_4$, filtered, and concentrated *in vacuo*, to give the crude product, which was purified by column chromatography on silica gel.

2.3 General procedure for alkylation using silyl enol ether– GP3

Aryl sulfonium salt (0.20 mmol, 1.0 equiv.) and 10-phenyl-phenothiazine (2.8 mg, 0.01 mmol, 5 mol%) were added to an oven-dried reaction vial equipped with a magnetic stirring bar and the vial was sealed with a crimp cap. The vial was then evacuated and backfilled with nitrogen three times. Next, dry DMSO (0.5 mL) was added under N_2 followed by (1-(4-fluorophenyl)vinyl)trimethylsilane (210 mg, 1.0 mmol, 5.0 equiv.). The crimp cap was sealed with parafilm. After irradiating with a Kessil PR 160 LED lamp (λ centred at 390 nm, 100% irradiance) at 2 cm away for 16 h under a cooling fan, the reaction mixture was quenched by addition of saturated aqueous $NaHCO_3$ (10 mL) and extracted with CH_2Cl_2 (3 x 10 mL). The combined organic layers were washed with brine (10 mL), then dried with Na_2SO_4 and concentrated *in vacuo*. The desired product was isolated from the crude mixture by column chromatography with pentane to 9:1 pentane:acetone as the eluent.

2.4 General procedure for sulfonylation – GP4

Aryl sulfonium salt (0.20 mmol, 1.0 equiv.), Cs_2CO_3 (130 mg, 0.40 mmol, 2.0 equiv.), and sodium sulfinate (99 mg, 0.60 mmol, 3.0 equiv.) were added to an oven-dried reaction vial equipped with a magnetic stirring bar and the vial was sealed with a crimp cap. Then, the vial was evacuated and backfilled with N_2 three times. Next, dry DMSO (0.13 M) was added, and the crimp cap was sealed with parafilm. After irradiating with a Kessil PR 160 LED lamp (λ centred at 390 nm, 100% irradiance) at 2 cm away for 16 h under a cooling fan, brine (10 mL) and CH_2Cl_2 (10 mL) were added, and the layers separated. The aqueous layer was extracted with CH_2Cl_2 (2 x 10 mL), the combined organic layers were dried with Na_2SO_4 and

concentrated *in vacuo*. The desired product was isolated from the crude mixture by column chromatography.

2.5 General procedure for ligand-coupling – GP5

To an oven-dried vial containing bromoarene (0.40 mmol, 1 equiv.) at RT was added 1.3 M isopropylmagnesium(II) chloride lithium chloride in THF (0.48 mmol, 1.2 equiv.). The reaction was stirred for 3 hours at RT before use.

In a separate flask, a solution of aryl sulfonium salt (0.20 mmol, 1.0 equiv.) in THF (0.1 M) was cooled to -78 °C, and the preformed aryl Grignard reagent (0.4 mmol, 2.0 equiv.) was added. The reaction mixture was stirred at -78 °C for 10 min, then was warmed to RT and stirred for a further 2 h. After this time saturated aqueous NH_4Cl was added to quench, and the aqueous layer was extracted with CH_2Cl_2 (10 mL). The combined organic layers were washed with brine (10 mL), dried using $MgSO_4$, filtered, and concentrated *in vacuo*, to give the crude product, which was purified by column chromatography on silica gel.

3. Methoxy sulfonium salt stability studies

Studies into the stability of DBT, PXT, and TT methoxy salts **1-DBT**, **1-PXT** and **1-TT** were conducted. Vials containing the neat methoxy salts were placed in three locations: a fume hood exposed to light (with the internal light turned off), a cupboard (wrapped in aluminium foil to prevent any light exposure), and the freezer (wrapped in aluminium foil at -20 °C). Samples (0.05 mmol) were taken after one week, then after one month with 1,3,5-trimethoxybenzene (0.1 mmol) used as the internal standard for ^{1}H NMR analysis of purity.

For DBT methoxy salt **1-DBT**, all samples showed no change in their physical appearance or ^{1}H NMR spectrum after one week. After one month a colour change of yellow to yellow/blue was observed for the sample left in the fume hood exposed to light, but the ^{1}H NMR spectrum remained unchanged. For the sample kept in the dark and the sample kept in the freezer, no physical changes, or changes in the ^{1}H NMR spectrum were observed. When left in the freezer for one year, the DBT methoxy salts purity remained >80% (Supplementary Figures 1–4).

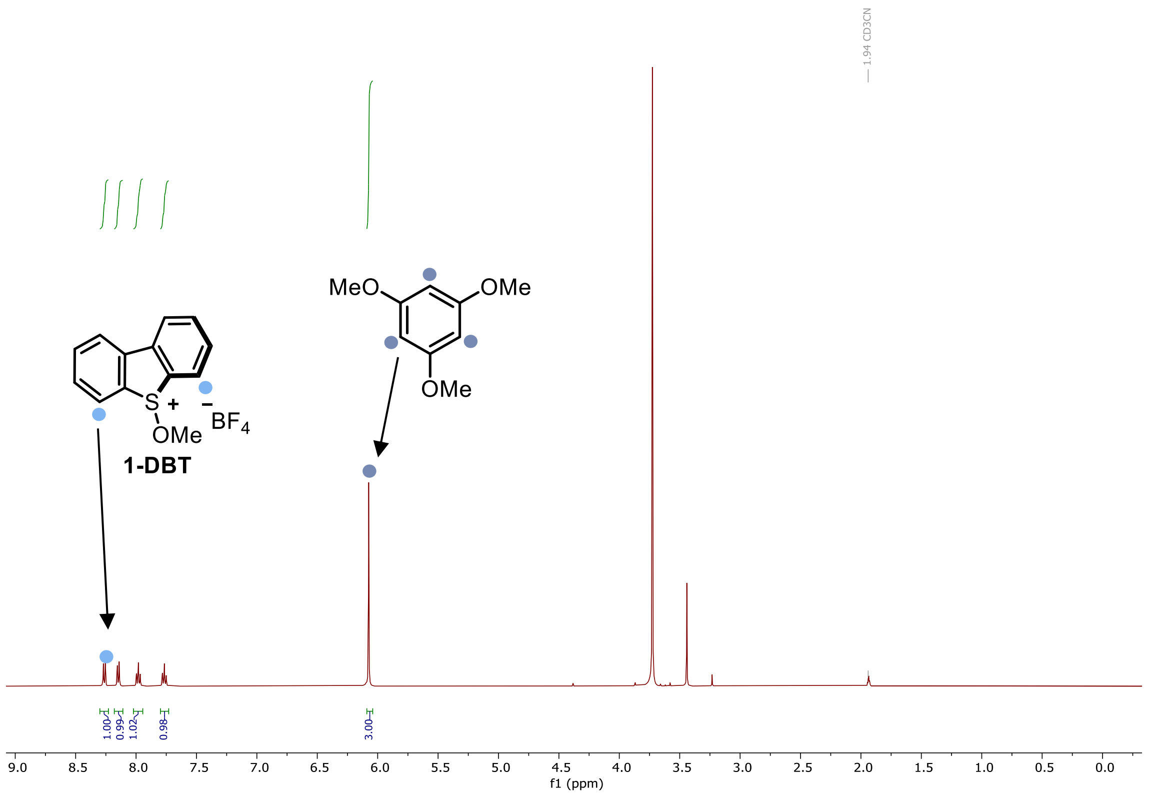

Supplementary Figure 1: ^{1}H qNMR spectrum (in CD_3CN) of a 0.05 mmol sample of methoxy DBT salt **1-DBT** using 0.1 mmol of trimethoxybenzene as the internal standard after being left in a fume hood for one month**.**

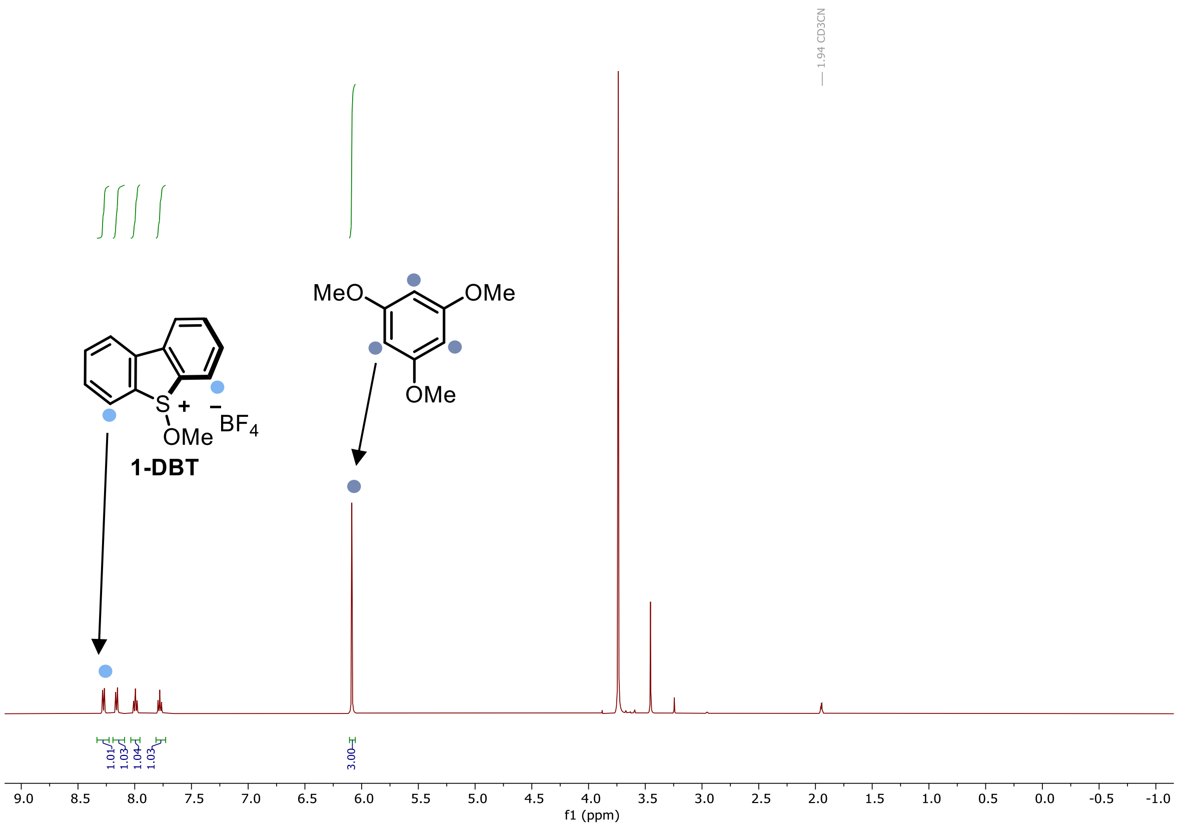

Supplementary Figure 2: ^{1}H qNMR spectrum (in CD_3CN) of a 0.05 mmol sample of methoxy DBT salt **1-DBT** using 0.1 mmol of trimethoxybenzene as the internal standard after being left in a closed cupboard for one month.

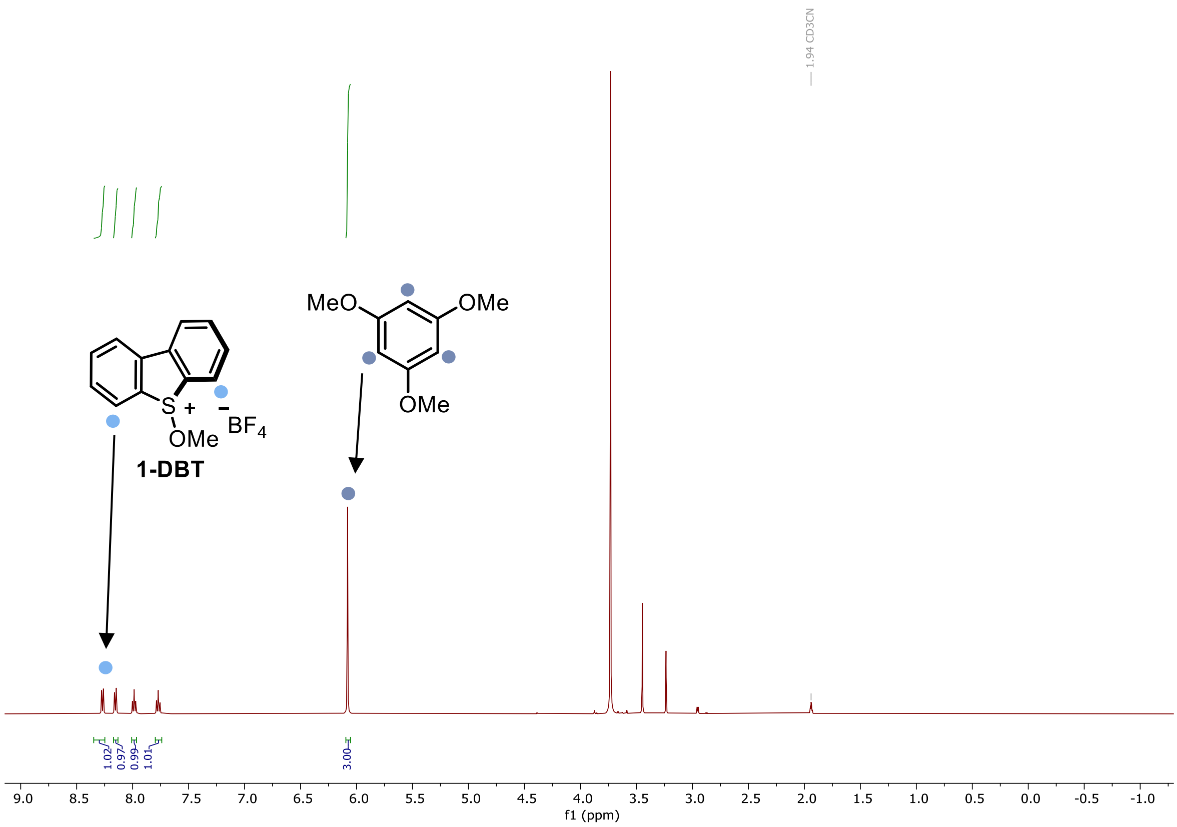

Supplementary Figure 3: ^{1}H qNMR spectrum (in CD_3CN) of a 0.05 mmol sample of methoxy DBT salt **1-DBT** using 0.1 mmol of trimethoxybenxene as the internal standard after being left for one month in a freezer.

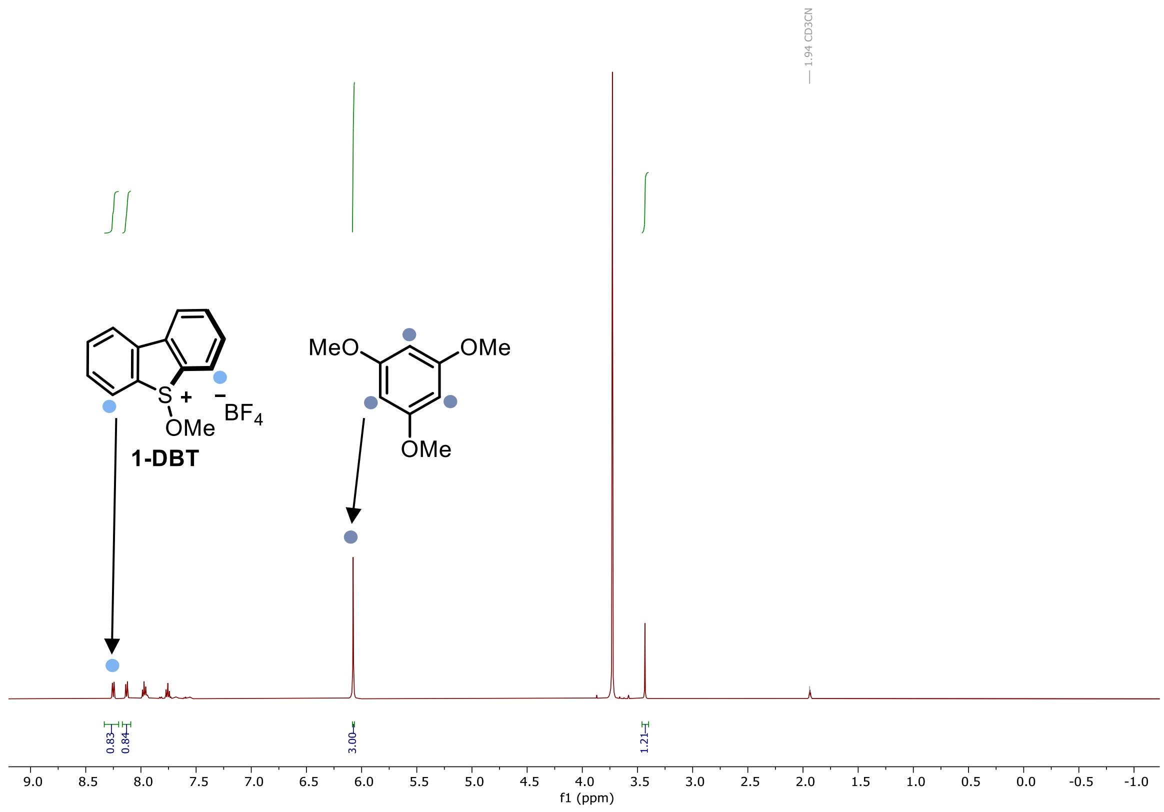

Supplementary Figure 4: ^{1}H qNMR spectrum (in CD_3CN) of a 0.05 mmol sample of methoxy DBT salt **1-DBT** using 0.1 mmol of trimethoxybenzene as the internal standard after being left for one year in a freezer.

For PXT methoxy salt **1-PXT**, after one week left in a fume hood (exposed to light), cupboard (wrapped in aluminium foil) and the freezer (wrapped in aluminium foil) there were no changes in the physical appearance or ^{1}H NMR spectrum of the salt. After one month, there were no changes in the physical appearance of any of the samples. However, after one month, the samples stored in the fume hood and cupboard showed a reduced purity of 95% by ^{1}H NMR analysis. The sample kept in the freezer remained at 100% purity by ^{1}H NMR analysis (Supplementary Figures 5–7).

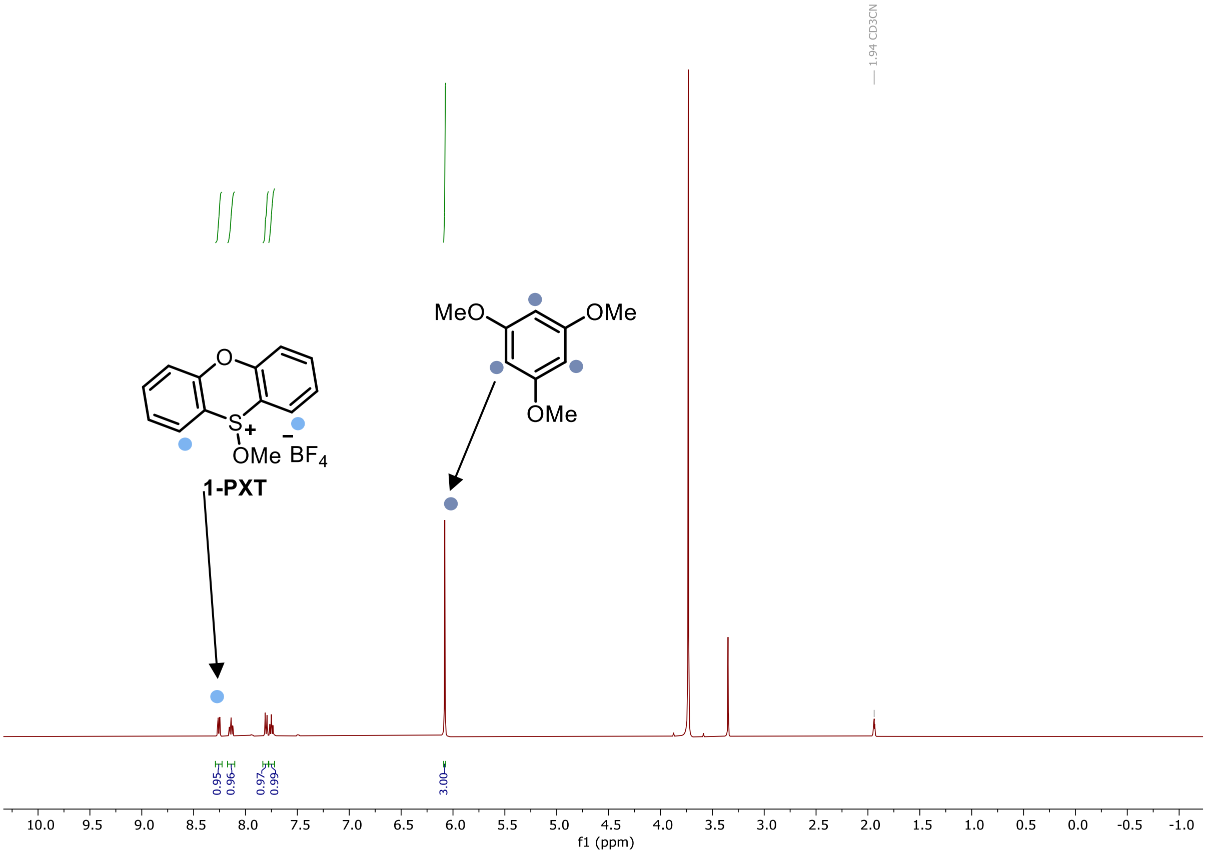

Supplementary Figure 5: ^{1}H qNMR spectrum (in CD_3CN) of a 0.05 mmol sample of methoxy PXT salt **1-PXT** using 0.1 mmol of trimethoxybenzene as the internal standard after being left in a fume hood for one month.

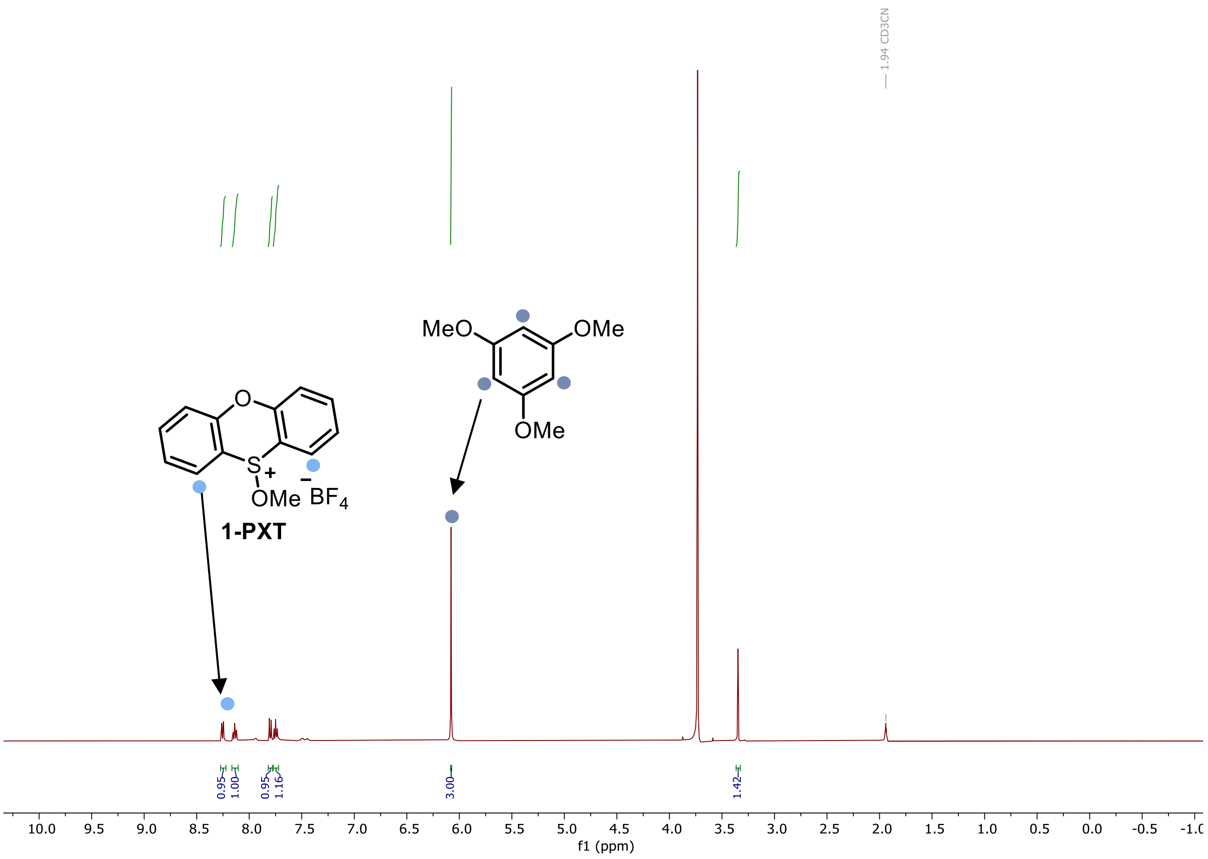

Supplementary Figure 6: ^{1}H qNMR spectrum (in CD_3CN) of a 0.05 mmol sample of methoxy PXT salt **1-PXT** using 0.1 mmol of trimethoxybenzene as the internal standard after being left in a closed cupboard for one month.

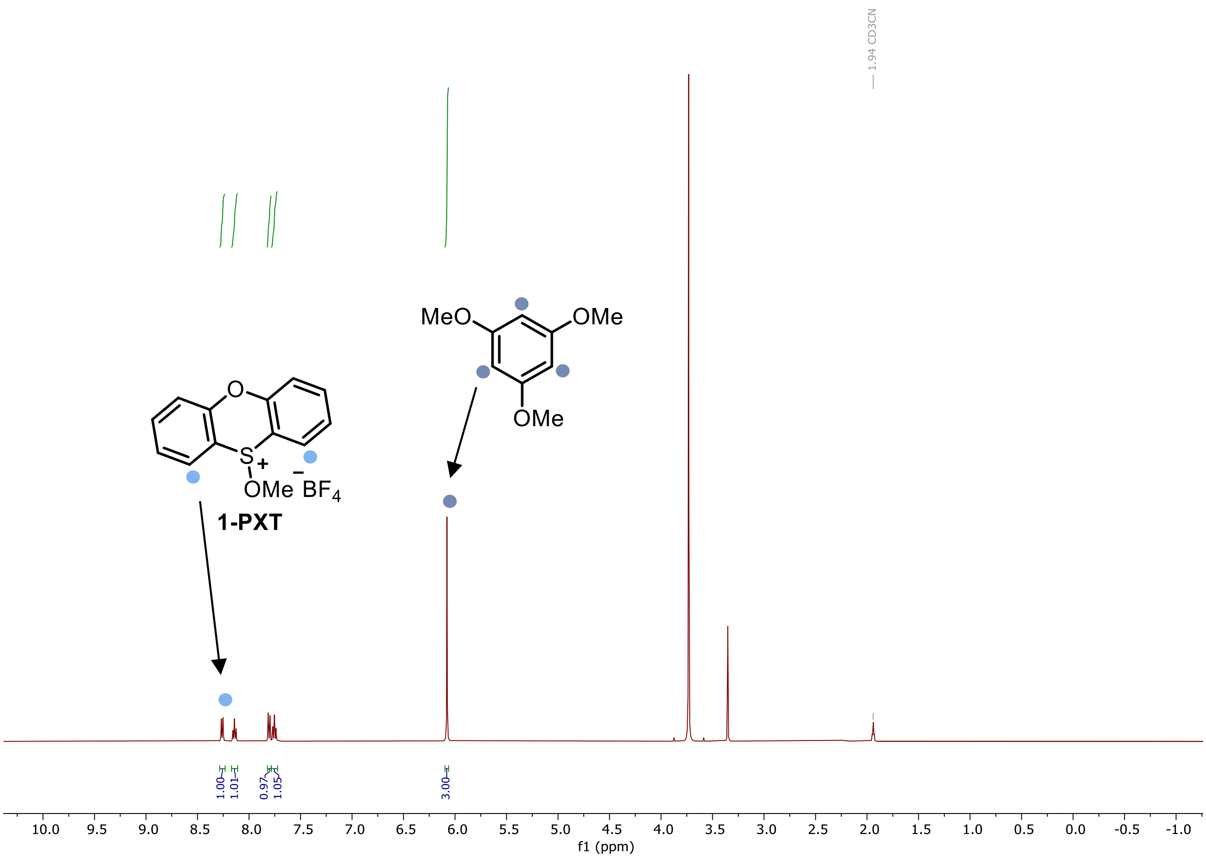

Supplementary Figure 7: ^{1}H qNMR spectrum (in CD_3CN) of a 0.05 mmol sample of methoxy PXT salt **1-PXT** using 0.1 mmol of trimethoxybenzene as the internal standard after being left in a freezer for one month.

For TT methoxy salt **1-TT**, after one week stored in a fume hood (exposed to light), cupboard (wrapped in aluminium foil) and the freezer (wrapped in aluminium foil) there were no changes in the physical appearance or ^{1}H NMR spectrum of the salt. After one month, there were no changes in the physical appearance of any of the sample. After one month, there were no changes in the physical appearance of any of the samples. However, after one month, the samples kept in the fume hood and cupboard showed a reduced purity of 90% and 97% by ^{1}H NMR analysis, respectively. The sample kept in the freezer remained at 100% purity by ^{1}H NMR analysis (Supplementary Figures 8–10).

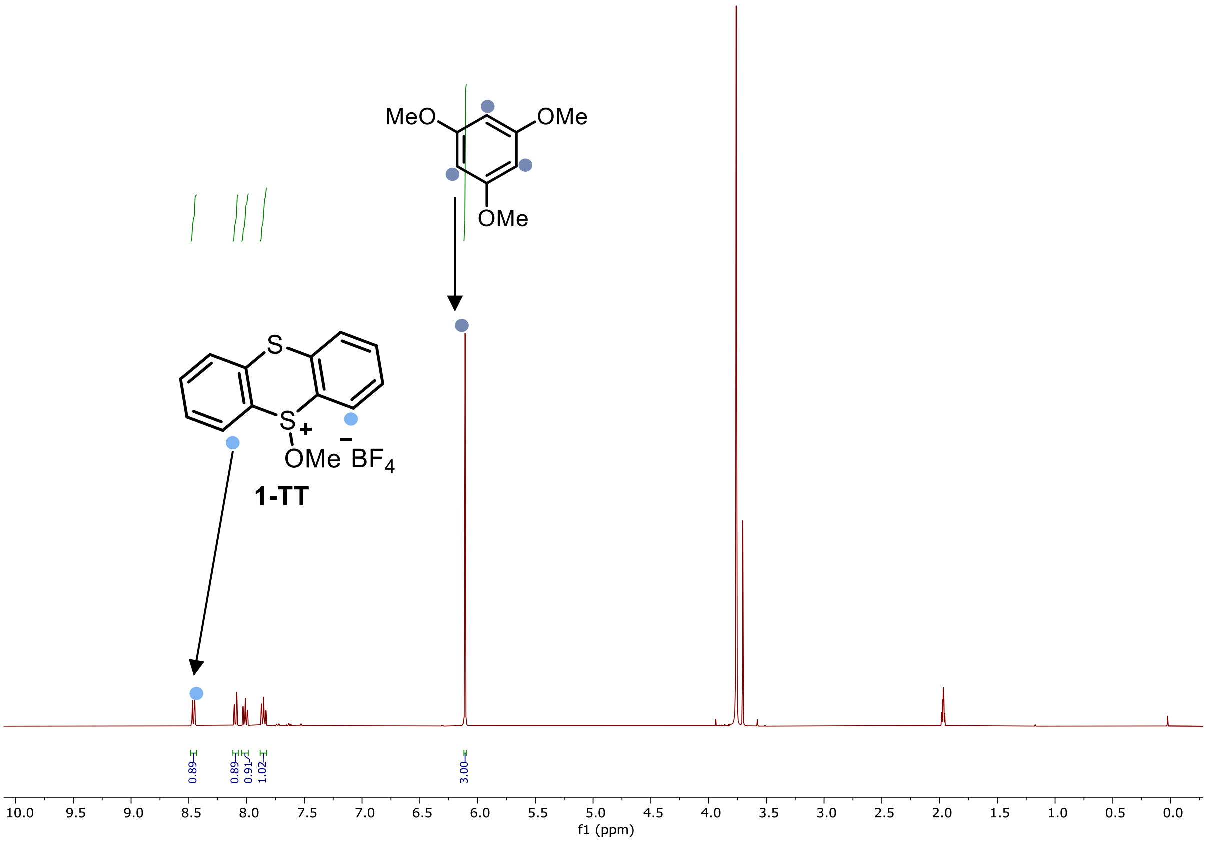

Supplementary Figure 8: ^{1}H qNMR spectrum (in CD_3CN) of a 0.05 mmol sample of methoxy TT salt **1-TT** using 0.1 mmol of trimethoxybenzene as the internal standard after being left in a fume hood for one month.

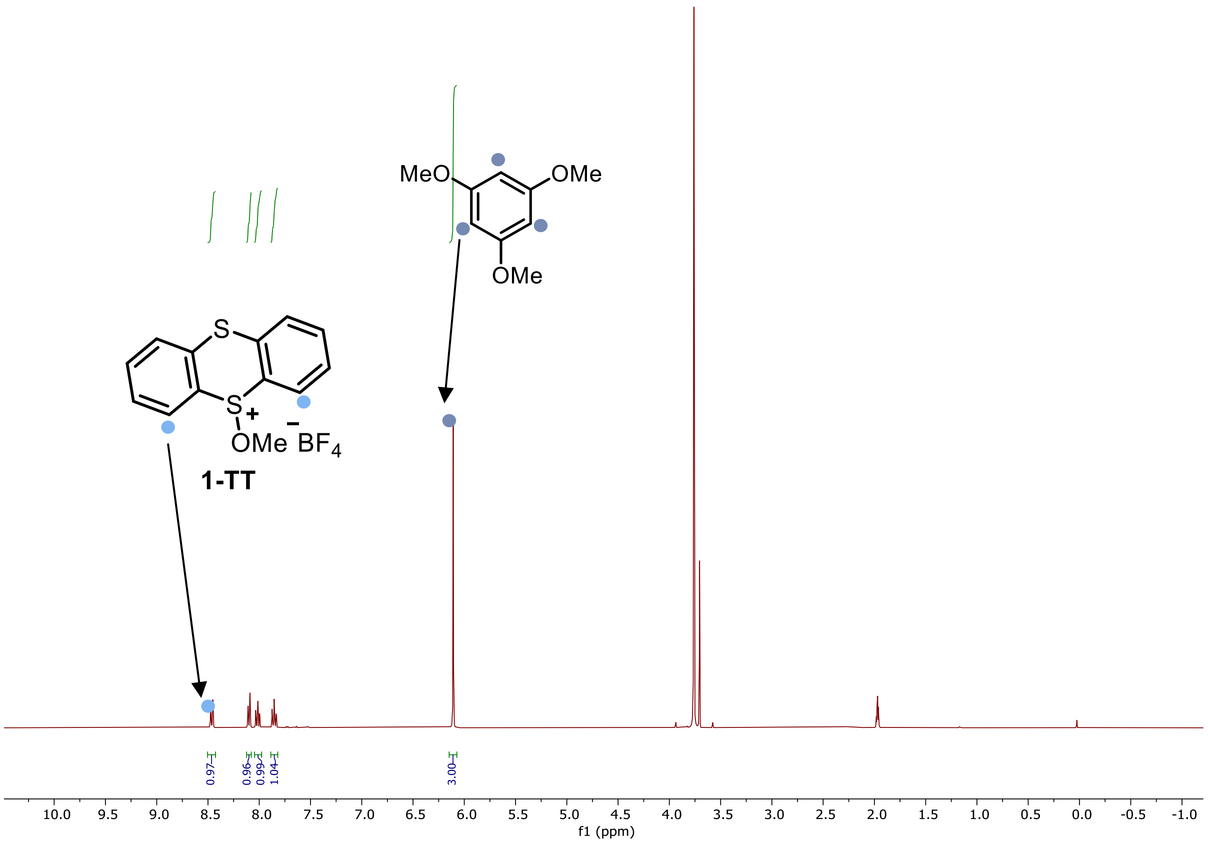

Supplementary Figure 9: ^{1}H qNMR spectrum (in CD_3CN) of a 0.05 mmol sample of methoxy TT salt **1-TT** using 0.1 mmol of trimethoxybenzene as the internal standard after being left in a closed cupboard for one month.

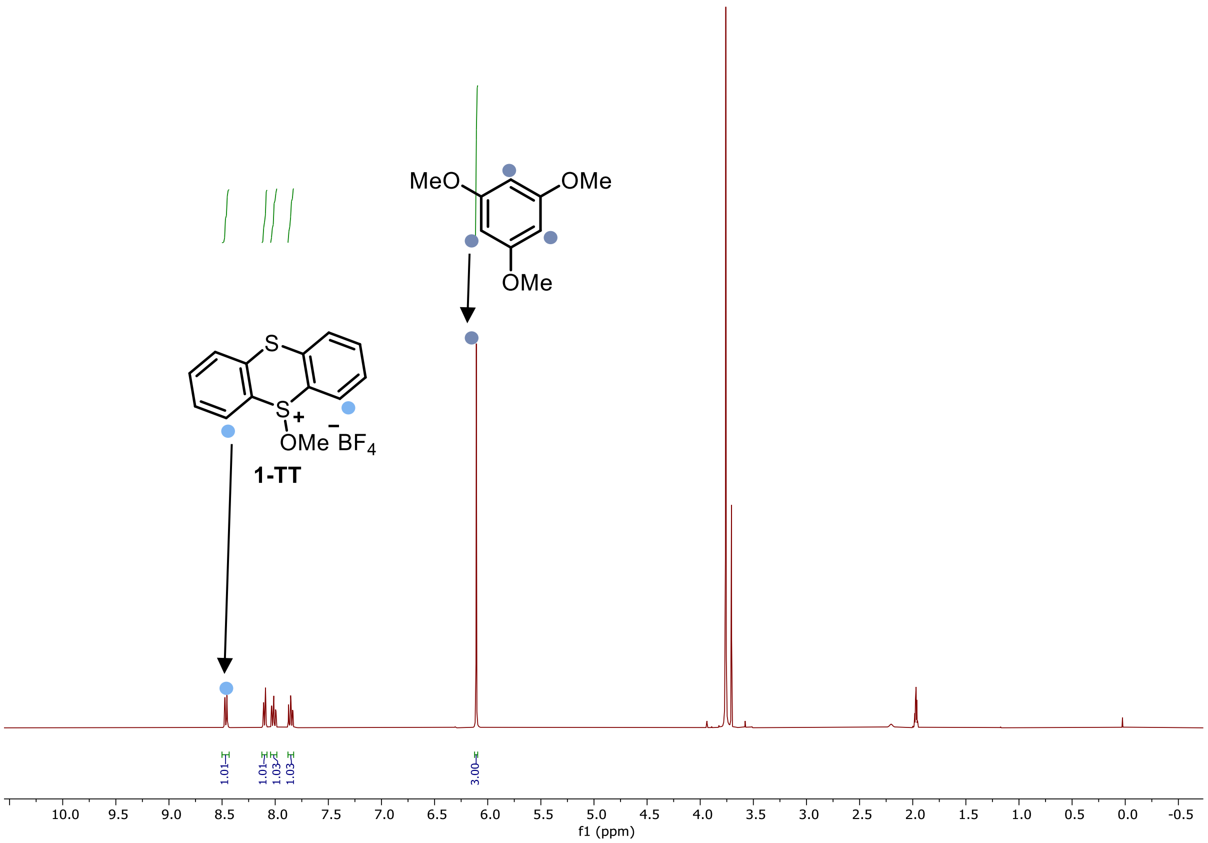

Supplementary Figure 10: ^{1}H qNMR spectrum (in CD_3CN) of a 0.05 mmol sample of methoxy TT salt **1-TT** using 0.1 mmol of trimethoxybenzene as the internal standard after being left in a freezer for one month.

4. Studies into salt purity and counterion

The purity of 3-pyridyl DBT salt **2a-DBT-BF$_4$** was shown to be 60% by ^{1}H qNMR analysis following trituration with CH_2Cl_2 as the sole method of purification (Supplementary Figure 11).

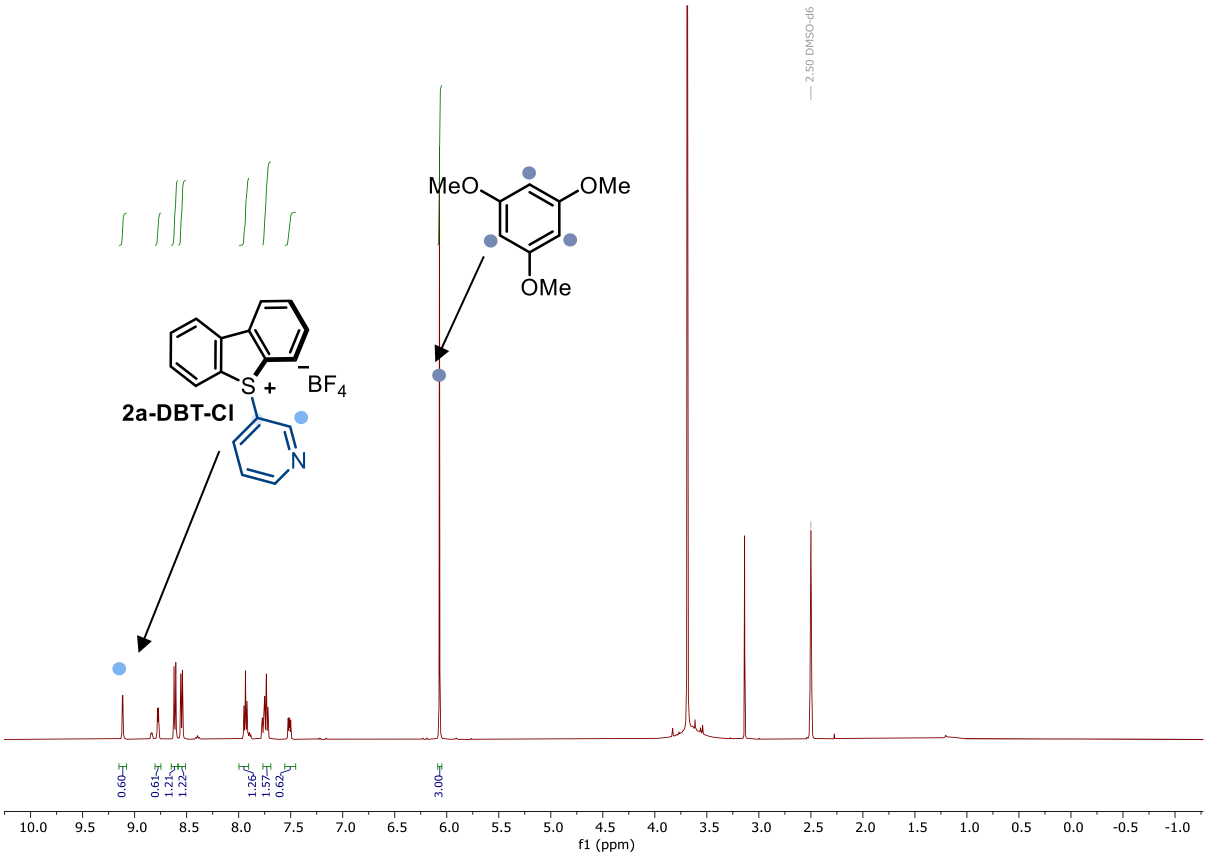

Supplementary Figure 11: ^{1}H qNMR spectrum (in DMSO-d$_6$) of a 0.1 mmol sample of 3-pyridyl DBT salt **2a-DBT-BF$_4$** using 0.1 mmol of trimethoxybenzene as the internal standard after purification by CH_2Cl_2 trituration.

Following an initial trituration with CH_2Cl_2 to remove the organic impurities, hot IPA was added to the sample until most of the solid was dissolved. Then, a hot filtration was performed on the resultant solution to remove the remaining solids. This gave a sample of 3-pyridyl DBT salt **2a-DBT-BF₄** with a >99% purity using 1H NMR analysis, however since this method resulted in poor yields of the salt following purification was not developed further (Supplementary Figure 12).

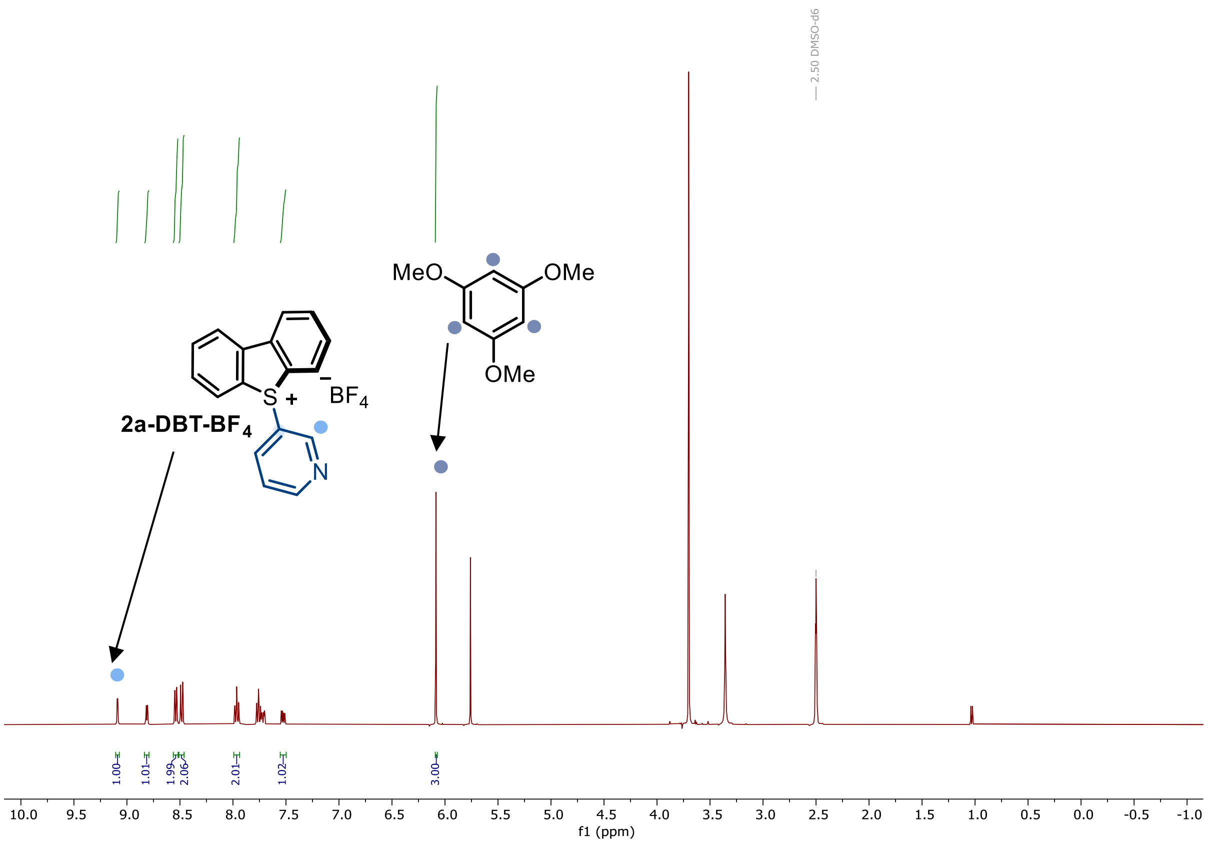

Supplementary Figure 12: ^{1}H qNMR spectrum (in DMSO-d_6) of a 0.1 mmol sample of 3-pyridyl DBT salt **2a-DBT-BF₄** using 0.1 mmol of trimethoxybenzene as the internal standard after purification by hot IPA trituration.

The purity of 3-pyridyl DBT salt **2a-DBT-BF$_4$** was shown to be 94% by ^{1}H qNMR analysis following purification by column chromatography with CH_2Cl_2 to 9:1 CH_2Cl_2:MeOH as the eluent (Supplementary Figure 13). Elemental metal analysis performed on an ICP-OES, Thermo Scientific iCAP 6300 Duo spectrometer showed the presence of Lithium: 0.17% and Magnesium: 4.84% following column chromatography with CH_2Cl_2 to 9:1 CH_2Cl_2:MeOH as the eluent. Elemental metal analysis of 3-pyridyl DBT salt **2a-DBT-BF$_4$** performed on an ICP-OES, Thermo Scientific iCAP 6300 Duo spectrometer following purification by column chromatography with EtOAc to IPA as the eluent showed the presence of Lithium: 1.94% and Magnesium: 3.63% column chromatography with EtOAc to IPA as the eluent.

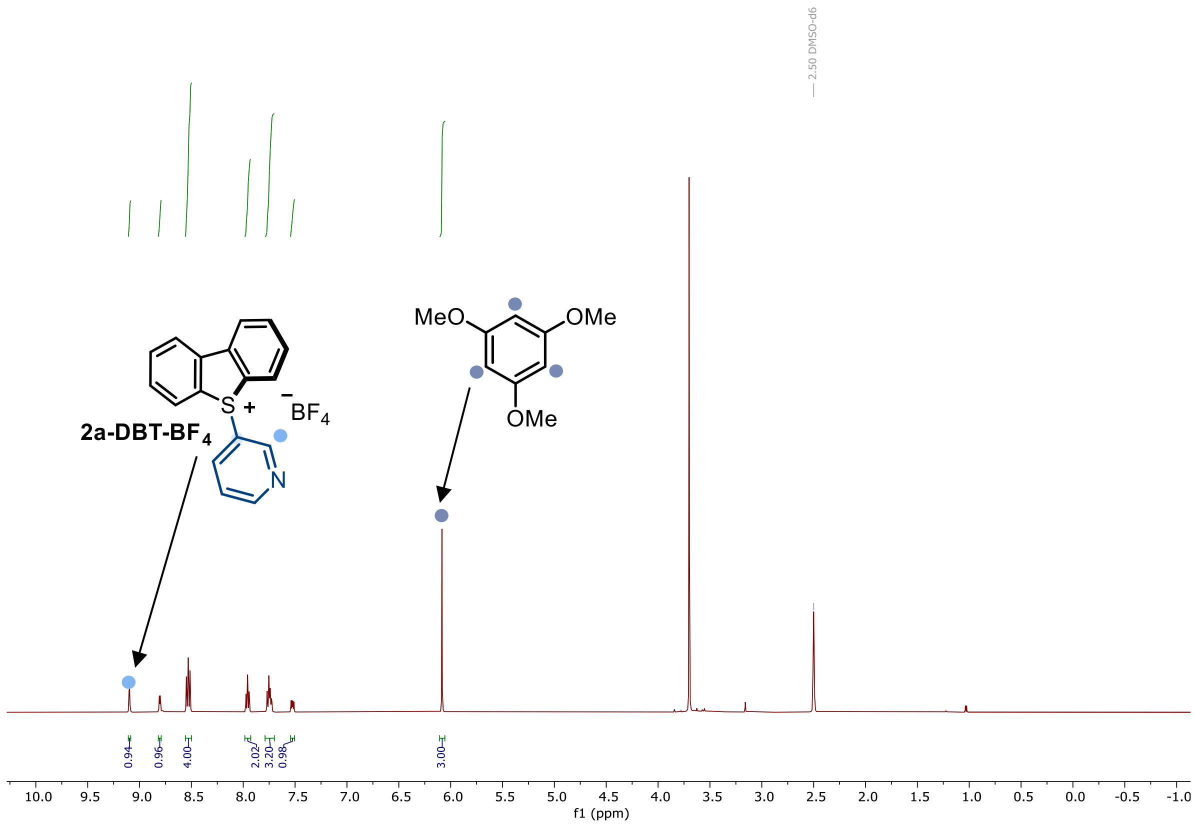

Supplementary Figure 13: ^{1}H qNMR spectrum (in DMSO-d$_6$) of a 0.1 mmol sample of 3-pyridyl DBT salt **2a-DBT-BF$_4$** using 0.1 mmol of trimethoxybenzene as the internal standard after purification by column chromatography with CH_2Cl_2 to 9:1 CH_2Cl_2:MeOH in as the eluent.

The purity of 3-pyridyl DBT salt **2a-DBT-Cl** was shown to be >99% by ^{1}H qNMR analysis following purification by NH_4Cl work-up and column chromatography with CH_2Cl_2 to 9:1 CH_2Cl_2:MeOH as the eluent (Supplementary Figure 14). Elemental metal analysis performed on an ICP-OES, Thermo Scientific iCAP 6300 Duo spectrometer showed the presence of Lithium: <0.1% and Magnesium: <0.1%.

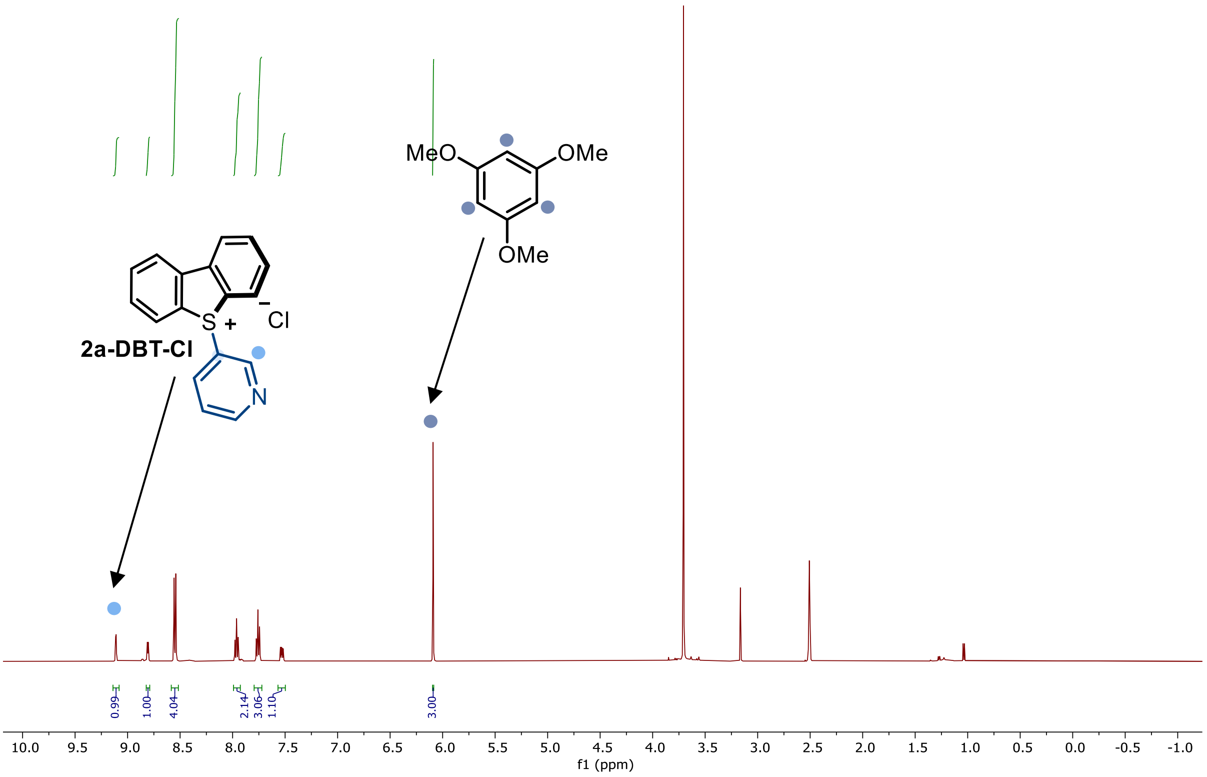

Supplementary Figure 14: ^{1}H qNMR spectrum (in DMSO-d_6) of a 0.1 mmol sample of 3-pyridyl DBT salt **2a-DBT-Cl** after purification by NH_4Cl work-up and column chromatography using 0.1 mmol of trimethoxybenzene as the internal standard.

The major counterion of the sulfonium salts following purification by NH_4Cl work-up and column chromatography was determined to be chloride. ^{19}F qNMR showed the amount of BF_4 to be 3% (Supplementary Figure 15) and halide analysis performed on the Metrohm 888 triturator showed 0% bromine and 12.05 % chloride (expected 11.90%) supporting the conclusion that chloride is the major counterion.

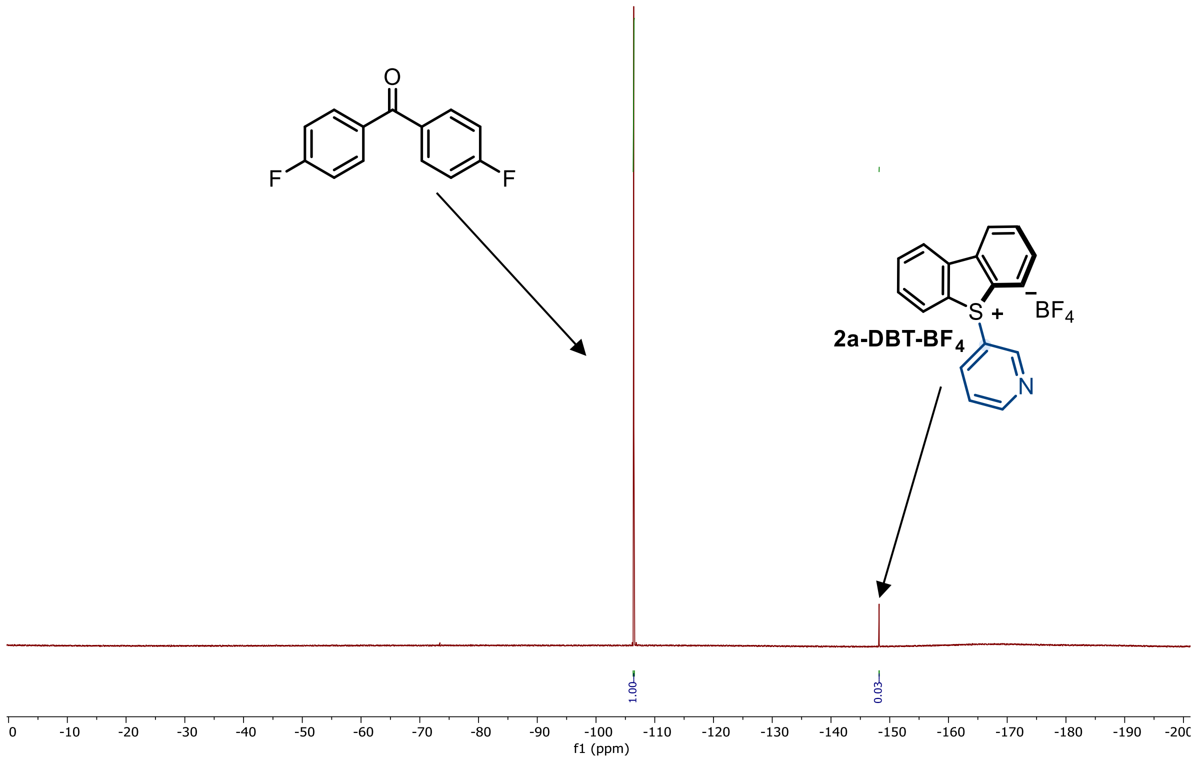

Supplementary Figure 15: ^{19}F qNMR spectrum (in DMSO-d_6) of a 0.05 mmol sample of 3-pyridyl DBT salt **2a-DBT-BF$_4$** using 0.1 mmol of 4,4'-difluorobenzophenone as the internal standard after purification by NH_4Cl work-up and column chromatography.

The major counterion of 3-pyridyl salt **2a-DBT** following by $NaBF_4$ work-up and column chromatography was determined to be tetrafluoroborate. ^{19}F qNMR showed the amount of BF_4 to be 96% by ^{19}F qNMR analysis and halide analysis performed on the Metrohm 888 triturator showed 0% bromide and 0% chloride supporting the conclusion that tetrafluoroborate is the major counterion (Supplementary Figure 16).

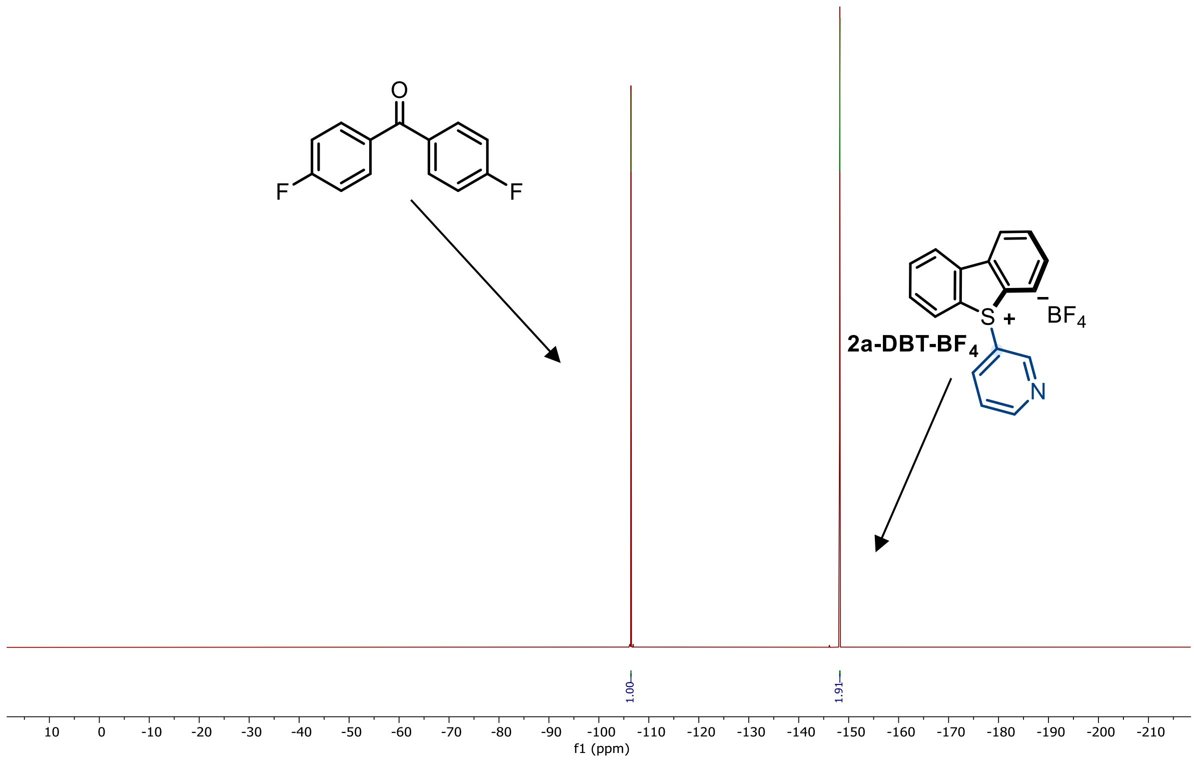

Supplementary Figure 16: ^{19}F qNMR spectrum (in DMSO-d_6) of a 0.1 mmol sample of 3-pyridyl DBT salt **2a-DBT-BF$_4$** using 0.1 mmol of 4,4'-difluorobenzophenone as the internal standard after purification by $NaBF_4$ work-up and column chromatography.

The purity of 3-methoxy DBT salt **2x-DBT-Cl** following NH_4Cl work-up and column chromatography was determined to be 98% by ^{1}H qNMR analysis (Supplementary Figure 17).

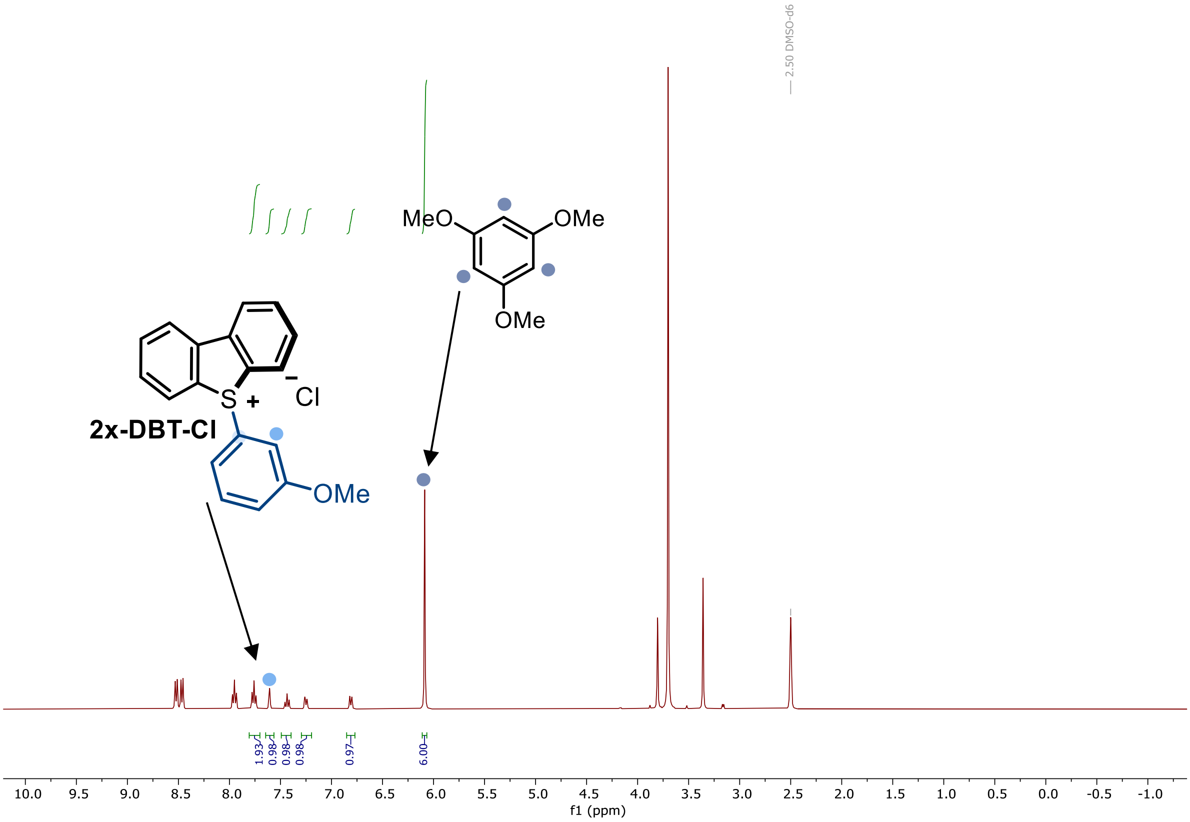

Supplementary Figure 17: ^{1}H qNMR spectrum (in DMSO-d$_6$) of a 0.05 mmol sample of 3-methoxy DBT salt **2x-DBT-Cl** after purification by NH_4Cl work-up and column chromatography using 0.1 mmol of trimethoxybenzene as the internal standard.

The major counterion of 3-methoxy DBT salt **2x-DBT-Cl** following NH_4Cl work-up and column chromatography was determined to be chloride. ^{19}F qNMR showed the amount of sulfonium salt containing the BF_4 counterion to be 2% and halide analysis performed on the Metrohm 888 triturator showed 0% bromide and 10.35% chloride (10.85% expected) (Supplementary Figure 18). The combination of halide and ^{19}F qNMR analysis supports the conclusion that chloride is the major counterion of aryl sulfonium salts containing electron-rich aryl rings.

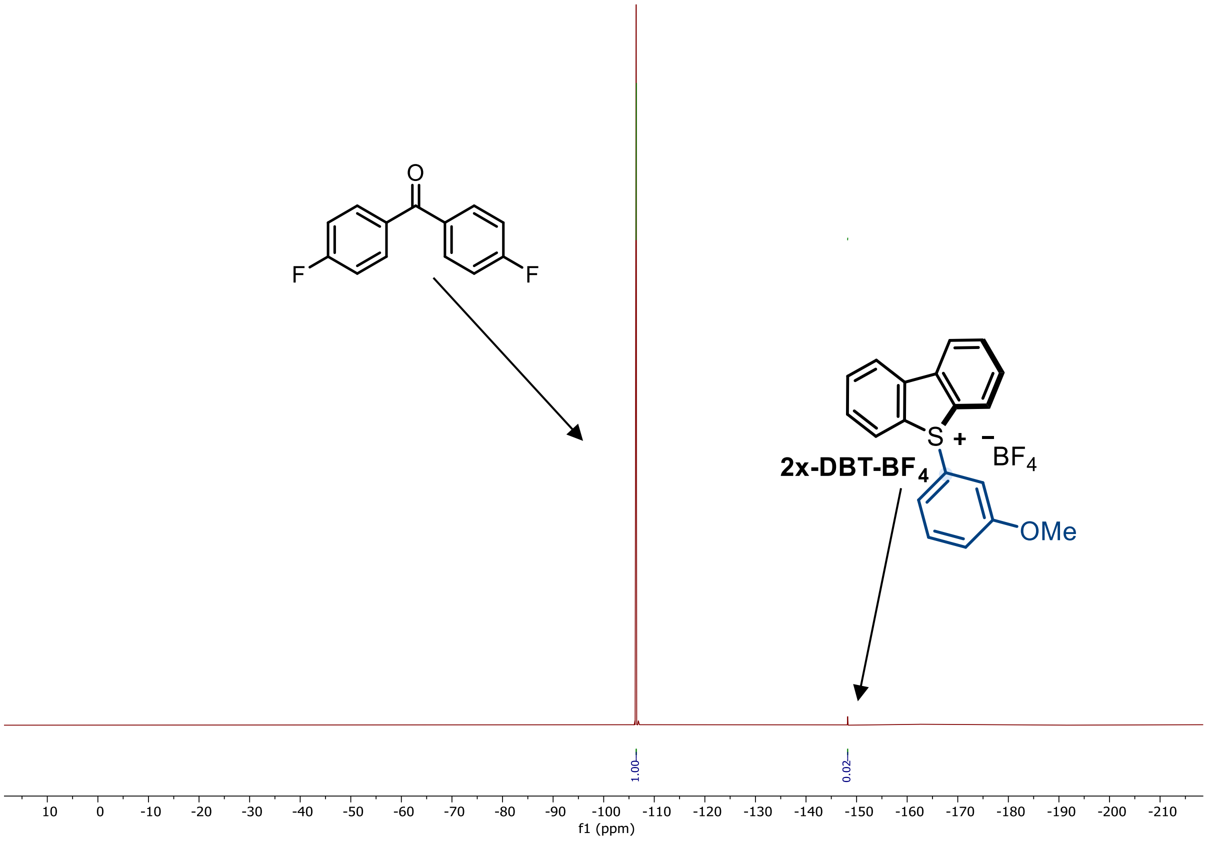

Supplementary Figure 18: ^{19}F qNMR spectrum (in DMSO-d_6) of a 0.05 mmol sample of 3-methoxy DBT salt **2x-DBT-BF$_4$** using 0.1 mmol of 4,4'-difluorobenzophenone as the internal standard after purification by NH_4Cl work-up and column chromatography.

5. Stability studies on 2b-DBT-Cl

Pyridyl sulfonium salt **2b-DBT-Cl** was treated with 1 M HCl (pH 3–4) in DMSO solvent (0.2 M), and the mixture stirred at room temperature for 30–45 min. No decomposition was observed by ^{1}H NMR analysis, indicating that the sulfonium salt is stable under these acidic conditions.

In contrast, when 2-pyridylboronic acid was subjected to identical conditions (1 M HCl, pH 3–4, 0.2 M DMSO, 30–45 min at room temperature), complete decomposition was observed. ^{11}B NMR analysis showed no detectable boron signal, indicating loss of the boronic acid moiety, and ^{1}H NMR analysis indicated the presence of pyridinium species. To confirm protodeboronation and the formation of pyridine, the solution was washed with a small amount of triethylamine and the presence of pyridine was confirmed by ^{1}H NMR.

6. Synthesis of S-Oxides

Dibenzo[*b*,*d*]thiophene-5-oxide S1

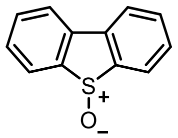

S1

Dibenzothiophene (9.21 g, 50.0 mmol, 1.00 equiv.) was dissolved in CH_2Cl_2 (125 mL) and the solution cooled to 0 °C. *m*-CPBA (11.8 g, 52.5 mmol, 1.05 equiv.) was dissolved in CH_2Cl_2 (125 mL) and was added portionwise over 1 hour at 0 °C. The resulting suspension was stirred for 2 h. The reaction mixture was quenched with saturated aqueous $NaHCO_3$ (125 mL) and the aqueous layer extracted with CH_2Cl_2 (125 mL × 2). The combined organic layers were washed with brine (125 mL), dried over $MgSO_4$, and concentrated *in vacuo*. The crude product was purified by recrystallization from refluxing EtOAc to give dibenzo[*b,d*]thiophene 5-oxide **S1** (9.01 g, 45.0 mmol, 90%) as a white solid; **^{1}H NMR** (400 MHz, $CDCl_3$) δ 8.00 (d, J = 7.5 Hz, 2H, ArCH), 7.82 (d, J = 7.5 Hz, 2H, ArCH), 7.61 (brt, J = 7.5 Hz, 2H, ArCH), 7.51 (brt, J = 7.5 Hz, 2H, ArCH); **^{13}C NMR** (101 MHz, $CDCl_3$) δ 145.3 (ArC), 137.3 (ArC), 132.7 (ArCH),

129.7 (ArCH), 127.7 (ArCH), 122.0 (ArCH); **HRMS** (ESI) $C_{12}H_8OSNa$ [M+Na]$^+$: calculated 223.0188, found 223.0183; ***v*max** (thin film/cm^{-1}) 3053, 1441, 1124, 1065, 1019, 751, 712. Data consistent with the literature[1][2]

Phenoxathiin 10-oxide S2

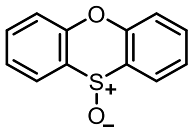

S2

Phenoxathiin (3.00 g, 15.0 mmol, 1.00 equiv.) was dissolved in CH_2Cl_2 (75 mL) and cooled to 0 °C. *m*-CPBA (3.72 g, 15.8 mmol, 1.05 equiv.) was dissolved in CH_2Cl_2 (75 mL) and was added portionwise over 1 hour at 0 °C and the resulting suspension was stirred for 2 h. The reaction mixture was quenched with saturated aqueous $NaHCO_3$ (75 mL) and the aqueous layer extracted with CH_2Cl_2 (75 mL x 2). The combined organic layers were washed with brine (75 mL), dried over $MgSO_4$, and concentrated *in vacuo*. The crude product was purified by recrystallization from refluxing EtOAc to give phenoxathiin 10-oxide **S2** (2.79 g, 12.9 mmol, 86%) as a white solid; **^{1}H NMR** (400 MHz, $CDCl_3$) δ 7.93 (dd, *J* = 8.0 Hz, 1.5, 2H, ArCH), 7.63 (ddd, *J* = 8.5, 7.5, 1.5 Hz, 2H, ArCH), 7.43 (dd, *J* = 8.5, 1.0 Hz, 2H, ArCH), 7.38 (ddd, *J* = 8.0, 7.5, 1.0 Hz, 2H, ArCH); **^{13}C NMR** (101 MHz, $CDCl_3$) δ 149.6 (ArC), 133.9 (ArCH), 131.2 (ArCH), 125.0 (ArCH), 123.8 (ArC), 119.0 (ArCH); **HRMS** (ESI) $C_{12}H_8O_2S$ [M+H]$^+$: calculated 217.0318, found 217.0318; ***v*max** (thin film/cm^{-1}) 3072, 1585, 1450, 1313, 1267, 1133, 1022, 881, 755. Data consistent with the literature[3]

Thianthrene 5-oxide S3

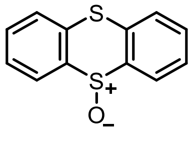

S3

A 100 mL round bottom flask was charged with thianthrene (4 g, 18.49 mmol, 1.00 equiv.) $Fe(NO_3)_3.9H_2O$ (7.47 g, 18.49 mmol, 1 equiv.) and NaBr (0.076 g, 0.739 mmol, 4 mol %) CH_2Cl_2 (37 mL) and AcOH (0.8 mL) were then added. The reaction mixture was stirred at RT until thianthrene was consumed. The reaction mixture was diluted with CH_2Cl_2 and washed with water. The aqueous layer extracted with CH_2Cl_2 (75 mL x 2). The combined organic layers were washed with brine (75 mL), dried over $MgSO_4$, and concentrated *in vacuo*. The crude product was purified by recrystallization from refluxing EtOAc to give thianthrene 5-oxide **S3** (3.1 g, 13.34 mmol, 72%) as a white solid; **^{1}H NMR** (400 MHz, $CDCl_3$) δ 7.93 (dd, *J* = 7.8, 1.4 Hz, 2H, ArCH), 7.63 (dd, *J* = 7.7, 1.2 Hz, 2H, ArCH), 7.56 (td, *J* = 7.6, 1.2 Hz, 2H, ArCH), 7.43 (td, *J* = 7.5, 1.4 Hz, 2H, ArCH); **^{13}C NMR** (126 MHz, $CDCl_3$) δ 141.4 (ArC), 129.8 (ArCH), 129.0 (ArCH), 128.4 (ArCH), 124.4 (ArCH); **HRMS** (APCI) $C_{12}H_9OS_2$ $[M+H]^+$: calculated 233.0089, found 233.0090; $\boldsymbol{v}_{\mathbf{max}}$ (thin film/cm^{-1}) 3051, 1568, 1432, 1247, 1116, 1073, 1032, 746, 552. Data consistent with the literature[4]

7. Synthesis of Alkoxy Sulfonium Salts

5-Methoxy-5*H*-dibenzo[*b,d*]thiophen-5-ium tetrafluoroborate 1-DBT

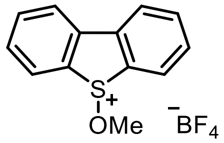

1-DBT

To a solution of dibenzothiophene *S*-oxide **S1** (8.01 g, 40.0 mmol, 1.0 equiv.) in CH_2Cl_2 (200 mL) was added trimethyloxonium tetrafluoroborate (5.92 g, 40.0 mmol, 1.0 equiv.), and the reaction mixture was stirred at RT for 2 h in the dark. The solvent was removed *in vacuo* and to the residue was added ethyl acetate (50 mL). The resulting precipitate was decanted, washed with ethyl acetate (2 x 50 mL), and dried *in vacuo* to afford methoxy sulfonium salt **1-DBT** (10.9 g, 36.1 mmol, 90%) as a yellow solid; **^{1}H NMR** (500 MHz, CD_3CN) *δ* 8.27 (dt, *J* = 8.0, 1.0 Hz, 2H, ArCH), 8.16 (dt, *J* = 8.0, 1.0 Hz, 2H, ArCH), 7.99 (td, *J* = 7.5, 1.0 Hz, 2H,

ArCH), 7.78 (td, J = 7.5, 1.0 Hz, 2H, ArCH), 3.44 (s, 3H, OCH_3); **^{13}C NMR** (126 MHz, CD_3CN) δ 141.6 (ArC), 138.4 (ArCH), 132.5 (ArCH), 131.4 (ArCH), 128.6 (ArC), 124.9 (ArCH), 117.9 (ArCH), 58.3 (OCH_3); **^{19}F NMR** (376 MHz, CD_3CN) δ -151.50; **HRMS** (ESI) $C_{13}H_{11}OS$ $[M+H]^+$: calculated 215.0525, found 215.0525; $\boldsymbol{v}_{\mathbf{max}}$ (thin film/cm^{-1}) 3089, 1582, 1445, 1291, 1033, 936, 787, 737.

10-Methoxy-10*H*-phenoxathiin-10-ium tetrafluoroborate 1-PXT

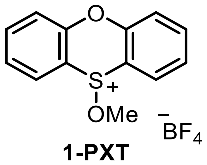

1-PXT

To a solution of phenoxathiin *S*-oxide **S2** (1.77 g, 8.20 mmol, 1.0 equiv.) in CH_2Cl_2 (12 mL) was added trimethyloxonium tetrafluoroborate (1.21, 8.20 mmol, 1.0 equiv.), and the reaction mixture was stirred at RT for 2 h in the dark. The solvent was removed *in vacuo* and to the residue was added EtOAc (15 mL). The resulting precipitate was decanted, washed with EtOAc (2 x 15 mL), and dried *in vacuo* to afford methoxy sulfonium salt **1-PXT** (2.00 g, 6.29 mmol, 77%) as a white solid; **^{1}H NMR** (400 MHz, CD_3CN) δ 8.30 (dd, J = 8.1, 1.6 Hz, 2H, ArCH), 8.18 (ddd, J = 8.8, 7.3, 1.6 Hz, 2H, ArCH), 7.84 (dd, J = 8.6, 1.2 Hz, 2H, ArCH), 7.79 (ddd, J = 8.3, 7.3, 1.1 Hz, 2H, ArCH), 3.38 (s, 3H, OCH_3); **^{13}C NMR** (101 MHz, CD_3CN) δ 152.6 (ArC), 140.4 (ArCH), 133.0 (ArCH), 128.0 (ArCH), 120.5 (ArCH), 117.9 (ArCH), 105.2 (ArC), 57.2 (OCH_3); **^{19}F NMR** (376 MHz, CD_3CN) δ -151.58; **HRMS** (ESI) $C_{13}H_{11}O_2S$ $[M+H]^+$: calculated 231.0474, found 231.0471; $\boldsymbol{v}_{\mathbf{max}}$ (thin film/cm^{-1}) 3092, 1588, 1448, 1275, 1023, 932, 763, 704.

5-Methoxy-5*H*-thianthren-5-ium tetrafluoroborate 1-TT

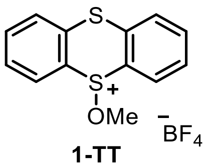

1-TT

To a solution of thianthrene 5-oxide **S3** (0.40 g, 1.72 mmol, 1.0 equiv.) in CH_2Cl_2 (9 mL) was added trimethyloxonium tetrafluoroborate (0.38 g, 2.58 mmol, 1.5 equiv.), and the reaction mixture was stirred at RT for 12 h in the dark. The solvent was removed *in vacuo* and to the residue was added EtOAc (15 mL). The resulting precipitate was decanted, washed with EtOAc (2 x 15 mL), and dried *in vacuo* to afford methoxy sulfonium salt **1-TT** (0.390 g, 1.17 mmol, 68%) as a white solid; **^{1}H NMR** (400 MHz, CD_3CN) δ 8.45 (dt, *J* = 8.2, 1.7 Hz, 2H, ArCH), 8.09 (d, *J* = 8.2 Hz, 2H, ArCH), 8.04 – 7.96 (m, 2H, ArCH), 7.84 (t, *J* = 7.7 Hz, 2H, ArCH), 3.69 (s, 3H, OCH_3); **^{13}C NMR** (126 MHz, CD_3CN) δ 137.2 (ArCH), 136.5 (ArC), 135.7 (ArCH), 129.8 (ArCH), 129.8 (ArCH), 119.0 (ArC), 117.9 (ArCH), 60.94 (OCH_3); **^{19}F NMR** (471 MHz, CD_3CN) δ -151.45; **HRMS** (ESI) $C_{13}H_{11}OS_2$ $[M+H]^+$: calculated 247.0246, found 247.0253; $\boldsymbol{v}_{\mathbf{max}}$ (thin film/cm^{-1}) 3084, 1572, 1452, 1297, 1032, 927, 760, 724.

8. Synthesis of Aryl Sulfonium Salts

5-(Pyridin-3-yl)-5*H*-dibenzo[*b,d*]thiophen-5-ium chloride 2a-DBT-Cl

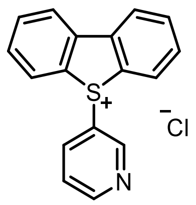

2a-DBT-Cl

Following GP1, with a magnesiation time of 5 h at RT, 3-bromopyridine (0.4 mL, 3.97 mmol, 1.2 equiv.), 1.3 M isopropylmagnesium(II) chloride lithium chloride in THF (3.5 mL, 4.63 mmol, 1.4 equiv.) and 5-methoxy-5*H*-dibenzo[*b,d*]thiophen-5-ium tetrafluoroborate **1-DBT** (1.0 g, 3.31 mmol, 1.0 equiv.) gave sulfonium salt **2a-DBT-Cl** (0.8 g, 2.68 mmol, 80%) as a cream solid. **^{1}H NMR** (500 MHz, DMSO-d_6) δ 9.10 (d, *J* = 2.5 Hz, 1H, ArCH), 8.80 (d, *J* = 5.0, 1H, ArCH), 8.55 (m, 4H, 2 x ArCH), 7.95 (t, *J* = 7.5, 2H, ArCH), 7.75 (m, 3H, 2 x ArCH), 7.52 (dd, *J* = 8.5, 4.5, 1H, ArCH); **^{13}C NMR** (126 MHz, DMSO-d_6) δ 153.9 (ArCH), 151.0 (ArCH), 139.4 (ArC), 136.8 (ArCH), 133.9 (ArCH), 133.1 (ArC), 131.2 (ArCH), 128.6 ArCH), 128.2 (ArC),

125.9 (ArCH), 124.5 (ArCH); **HRMS** (ESI) $C_{17}H_{12}NS$ $[M]^+$: calculated 262.0685, found 262.0676; ***v****max* (thin film/cm^{-1}) 3071, 1607, 1537, 1468, 1291, 1198, 1024, 767, 663.

Synthesis of 2a-DBT-Cl using arylzinc – Preliminary Study

To an oven-dried vial containing 3-bromopyridine (0.11 mL, 1.2 mmol, 1.2 equiv.) at RT was added 1.3 M isopropylmagnesium(II) chloride lithium chloride in THF (1.1 mL, 1.4 mmol, 1.4 equiv.). The reaction was stirred for 5 h at RT. Then, $ZnCl_2$ (0.5 M in THF) (2.8 mL, 1.4 mmol, 1.4 equiv.) was then added at 0 °C and the solution stirred for 1.5 h.

In a separate flask, a solution of methoxysulfonium salt (0.300 g, 1.0 mmol, 1.0 equiv.) in CH_2Cl_2 (0.1 M) was cooled to -78 °C, and the preformed aryl zinc reagent was added. The reaction mixture was stirred at -78 °C for 10 min, then was warmed to RT and stirred for a further 2 h. After this time methanol was added to quench, then saturated aqueous NH_4Cl (50 mL) and a solution of 10% IPA in $CHCl_3$ (45 mL for a 1.0 mmol scale reaction) were also added and the layers separated. The aqueous layer was extracted with a solution of 10% IPA in $CHCl_3$ (3 x 45 mL), the combined organic layers were dried with Na_2SO_4 and the solvent was removed *in vacuo*. The crude product was purified by column chromatography on silica gel with CH_2Cl_2 to 9:1 CH_2Cl_2:MeOH as eluent and gave sulfonium salt **2a-DBT-Cl** (90 mg, 0.30 mmol, 30%).

5-(Pyridin-3-yl)-5*H*-dibenzo[*b,d*]thiophen-5-ium tetrafluoroborate 2a-DBT-BF$_4$

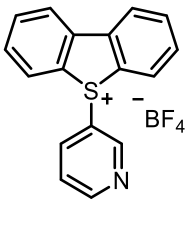

2a-DBT-BF$_4$

To an oven-dried vial containing 3-bromopyridine (0.12 mL, 1.20 mmol, 1.2 equiv.) at RT was added 1.3 M isopropylmagnesium(II) chloride lithium chloride in THF (1.1 mL, 1.40 mmol, 1.4 equiv). The reaction was stirred for 5 h at RT before use.

In a separate flask a solution of methoxy DBT salt **1-DBT** (302 mg, 1.00 mmol, 1.0 equiv.) in CH_2Cl_2 (10 mL) was cooled to -78 °C, and the preformed aryl Grignard reagent (1.2 equiv.) was added. The reaction mixture was stirred at -78 °C for 10 min, then was warmed to RT and stirred for a further 2 hours. After this time methanol was added to quench, then 10% w/w $NaBF_4$ solution (100 mL) and a solution of 10% IPA in $CHCl_3$ (45 mL) were added, and the layers separated. The aqueous layer was extracted with a solution of 10% IPA in $CHCl_3$ (3 x 45 mL), the combined organic layers were dried with Na_2SO_4, and the solvent was removed *in vacuo*. The crude product was purified by column chromatography using CH_2Cl_2 to 9:1 CH_2Cl_2:MeOH as eluent to give sulfonium salt **2a-DBT-BF$_4$** (272 mg, 0.78 mmol, 78%) as a cream solid. **^{1}H NMR** (400 MHz, DMSO-d$_6$) δ 9.07 (d, *J* = 2.5 Hz, 1H, ArCH), 8.83 (dd, *J* = 4.7, 1.4 Hz, 1H, ArCH), 8.54 (brd, *J* = 7.8 Hz, 2H, ArCH), 8.41 (brd, *J* = 8.2, 1.0 Hz, 2H, ArCH), 7.97 (td, *J* = 7.6, 1.1 Hz, 2H, ArCH), 7.81–7.73 (m, 2H, ArCH), 7.69 (ddd, *J* = 8.4, 2.5, 1.4 Hz, ArCH), 7.53 (dd, *J* = 8.4, 4.7 Hz, 1H, ArCH); **^{13}C NMR** (101 MHz, DMSO-d$_6$) δ 154.2 (ArCH), 150.9 (ArCH), 139.4 (ArC), 136.7 (ArCH), 134.0 (ArCH), 133.0 (ArC), 131.4 (ArCH), 128.4 (ArCH), 127.8 (ArC), 126.1 (ArCH), 124.6 (ArCH); **^{19}F NMR** (376 MHz, DMSO-d$_6$) δ -148.18; **HRMS** (ESI) $C_{17}H_{12}NS$ $[M]^+$: calculated 262.0685, found 262.0684; $\boldsymbol{\nu_{max}}$ (thin film/cm^{-1}) 2920, 1572, 1451, 1471, 1200, 1027, 756, 614, 421.

10-(Pyridin-3-yl)-10*H*-phenoxathiin-10-ium chloride 2a-PXT-Cl

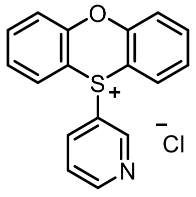

2a-PXT-Cl

Following GP1, with a magnesiation time of 5 h at RT, 3-bromopyridine (0.23 mL, 2.40 mmol, 1.2 equiv.), 1.3 M isopropylmagnesium(II) chloride lithium chloride in THF (2.2 mL, 2.80 mmol, 1.4 equiv.), and 10-methoxy-10*H*-phenoxathiin-10-ium tetrafluoroborate **1-PXT** (636 mg, 2.00 mmol, 1.0 equiv.) gave sulfonium salt **2a-PXT-Cl** (527 mg, 1.68 mmol, 84%) as a beige solid;

^{1}H NMR (400 MHz, DMSO-d$_6$) δ 9.02 (d, J = 2.5 Hz, 1H, ArCH), 8.81 (dd, J = 5.0, 1.5 Hz, 1H, ArCH), 8.40 (dd, J = 8.0, 1.5 Hz, 2H, 2 x ArCH), 7.99 (ddd, J = 8.5, 2.5, 1.5 Hz, 1H, ArCH), 7.91 (ddd, J = 8.5, 7.5, 1.5 Hz, 2H, 2 x ArCH), 7.73 (dd, J = 8.5, 1.0 Hz, 2H, 2 x ArCH), 7.66–7.55 (m, 3H, 3 x ArCH); **^{13}C NMR** (126 MHz, DMSO-d$_6$) δ 154.0 (ArCH), 150.3 (ArCH), 149.1 (ArC), 136.8 (ArCH), 136.5 (ArCH), 132.0 (ArCH), 131.0 (ArC), 126.9 (ArCH), 126.1 (ArC), 120.0 (ArCH), 106.2 (ArC); **HRMS** (ESI) $C_{17}H_{12}ONS$ $[M]^+$: calculated 278.0634, found 278.0636; $\boldsymbol{\nu}_{\mathbf{max}}$ (thin film/cm^{-1}) 3063, 1581, 1460, 1336, 1270, 1220, 1020, 882, 756.

10-(Pyridin-3-yl)-10*H*-phenoxathiin-10-ium tetrafluoroborate 2a-PXT-BF$_4$

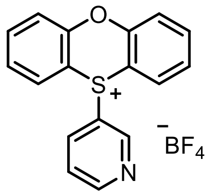

2a-PXT-BF$_4$

To an oven-dried vial containing 3-bromopyridine (0.36 mL, 3.77 mmol, 1.2 equiv.) at RT was added 1.3 M isopropylmagnesium(II) chloride lithium chloride in THF (3.3 mL, 4.39 mmol, 1.4 equiv). The reaction was stirred for 5 h at RT before use.

In a separate flask, a solution of methoxy DBT salt **1-PXT** (1.0 g, 3.10 mmol, 1.0 equiv.) in CH_2Cl_2 (30 mL) was cooled to -78 °C, and the preformed aryl Grignard reagent (1.2 equiv.) was added. The reaction mixture was stirred at -78 °C for 10 min, then was warmed to RT and stirred for a further 2 hours. After this time methanol was added to quench, then 10% w/w $NaBF_4$ solution (50 mL) and a solution of 10% IPA in $CHCl_3$ (100 mL) were added, and the layers separated. The aqueous layer was extracted with a solution of 10% IPA in $CHCl_3$ (3 x 50 mL). The combined organic layers were dried with Na_2SO_4, and the solvent was removed *in vacuo*. The crude product was purified by column chromatography using CH_2Cl_2 to 9:1 CH_2Cl_2:MeOH as eluent to give sulfonium salt **2a-PXT-BF$_4$** (780 mg, 2.14 mmol, 68%) as a cream solid. **^{1}H NMR** (400 MHz, DMSO-d$_6$) δ 9.03 (dd, J = 2.6, 0.8 Hz, 1H, ArCH), 8.84 (dd,

J = 4.7, 1.4 Hz, 1H, ArCH), 8.36 (dt, *J* = 8.1, 1.6 Hz, 2H, ArCH), 8.01 – 7.96 (m, 1H, ArCH), 7.93 (ddd, *J* = 8.8, 7.4, 1.6 Hz, 2H, ArCH), 7.75 (dd, *J* = 8.4, 1.2 Hz, 2H, ArCH), 7.68 – 7.57 (m, 3H, ArCH); **^{13}C NMR** (101 MHz, DMSO-d_6) δ 154.5 (ArCH), 150.8 (ArC), 149.5 (ArCH), 137.2 (ArCH), 137.0 (ArCH), 132.3 (ArCH), 131.4 (ArC), 127.4 (ArCH), 126.6 (ArCH), 120.5 (ArCH), 106.5 (ArC); **^{19}F NMR** (376 MHz, DMSO-d_6) δ -148.23; **HRMS** (ESI) $C_{17}H_{12}ONS$ [M]$^+$: calculated 278.0634, found 278.0623; ***v*max** (thin film/cm^{-1}) 3090, 1582, 1459, 1318, 1271, 1228, 1029, 883, 753.

5-(Pyridin-3-yl)-5*H*-thianthren-5-ium chloride 2a-TT-Cl

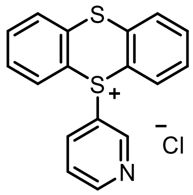

2a-TT-Cl

Following GP1, with a magnesiation time of 5 h at RT, 3-bromopyridine (0.1 mL, 1.07 mmol, 1.2 equiv.), 1.3 M isopropylmagnesium(II) chloride lithium chloride in THF (0.96 mL, 1.25 mmol, 1.4 equiv.), and 5-methoxy-5*H*-thianthren-5-ium tetrafluoroborate **1-TT** (300 mg, 0.897 mmol, 1.0 equiv.) gave sulfonium salt **2a-TT-Cl** (144 mg, 0.436 mmol, 48%) as a beige solid; **^{1}H NMR** (400 MHz, DMSO-d_6) δ 8.76 (dd, *J* = 4.7, 1.4 Hz, 1H, ArCH), 8.68 – 8.61 (m, 2H, ArCH), 8.37 (d, *J* = 2.5 Hz, 1H, ArCH), 8.09 (dd, *J* = 7.8, 1.4 Hz, 2H, ArCH), 7.94 (td, *J* = 7.7, 1.5 Hz, 2H, ArCH), 7.88 (td, *J* = 7.7, 1.5 Hz, 2H, ArCH), 7.66 (ddd, *J* = 8.4, 2.7, 1.4 Hz, 1H, ArCH), 7.58 (ddd, *J* = 8.4, 4.8, 0.8 Hz, 1H, ArCH); **^{13}C NMR** (101 MHz, DMSO-d_6) δ 153.1 (ArCH), 148.5 (ArCH), 137.0 (ArCH), 136.6 (ArCH), 135.9 (ArC), 135.33 (ArCH), 130.81 (ArCH), 130.0 (ArCH), 125.3 (ArCH), 123.8 (ArC), 119.6 (ArC); **HRMS** (ESI) $C_{17}H_{12}NS_2$ [M]$^+$: calculated 294.0406, found 294.0399; **v_{max}** (thin film/cm^{-1}) 3319, 1634, 1446, 1414, 1289, 1195, 1009, 762, 693.

5-(Pyridin-3-yl)-5*H*-thianthren-5-ium tetrafluoroborate 2a-TT-BF$_4$

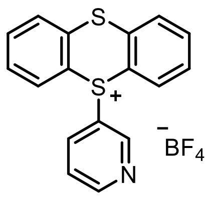

2a-TT-BF$_4$

To an oven-dried vial containing 3-bromopyridine (0.1 mL, 1.07 mmol, 1.2 equiv.) at RT was added 1.3 M isopropylmagnesium(II) chloride lithium chloride in THF (0.96 mL, 1.25 mmol, 1.4 equiv). The reaction was stirred for 5 h at RT before use.

In a separate flask, a solution of methoxy DBT salt **1-TT** (300 mg, 0.897 mmol, 1.0 equiv.) in CH_2Cl_2 (9 mL) was cooled to -78 °C, and the preformed aryl Grignard reagent (1.2 equiv.) was added. The reaction mixture was stirred at -78 °C for 10 min, then was warmed to RT and stirred for a further 2 hours. After this time, methanol was added to quench, then 10% w/w $NaBF_4$ solution (20 mL) and a solution of 10% IPA in $CHCl_3$ (50 mL) were added, and the layers separated. The aqueous layer was extracted with a solution of 10% IPA in $CHCl_3$ (3 x 50 mL). The combined organic layers were dried with Na_2SO_4, and the solvent was removed *in vacuo*. The crude product was purified by column chromatography using CH_2Cl_2 to 9:1 CH_2Cl_2:MeOH as eluent to give sulfonium salt **2a-TT-BF$_4$** (158 mg, 0.414 mmol, 46%) as a cream solid. **^{1}H NMR** (400 MHz, DMSO-d$_6$) δ 8.77 (dd, *J* = 4.7, 1.5 Hz, 1H, ArCH), 8.60 (dd, *J* = 7.9, 1.5 Hz, 2H, ArCH), 8.36 (dd, *J* = 2.6, 0.8 Hz, 1H, ArCH), 8.08 (dd, *J* = 7.8, 1.4 Hz, 2H, ArCH), 7.94 (td, *J* = 7.6, 1.6 Hz, 2H, ArCH), 7.88 (td, *J* = 7.7, 1.5 Hz, 2H, ArCH), 7.64 (ddd, *J* = 8.4, 2.7, 1.5 Hz, 1H, ArCH), 7.58 (ddd, *J* = 8.4, 4.7, 0.8 Hz, 1H, ArCH); **^{13}C NMR** (101 MHz, DMSO-d$_6$) δ 153.2 (ArCH), 148.5 (ArCH), 136.9 (ArCH), 136.4 (ArCH), 135.9 (ArC), 135.3 (ArCH), 130.8 (ArCH), 130.1 (ArCH), 125.4 (ArCH), 123.7 (ArC), 119.4 (ArC); **^{19}F NMR** (376 MHz, DMSO-d$_6$) δ -148.22; **HRMS** (APCI) $C_{17}H_{12}NS_2$ $[M]^+$: calculated 294.0406, found 294.0399; ***ν*$_{max}$** (thin film/cm^{-1}) 3082, 1565, 1447, 1415, 1264, 1172, 1027, 808, 763.

5-(Pyridine-2-yl)-5*H*-dibenzo[*b,d*]thiophen-5-ium chloride 2b-DBT-Cl

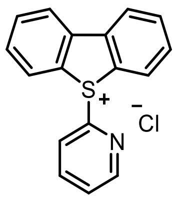

2b-DBT-Cl

Following GP1, with a magnesiation time of 18 h at RT, 2-bromopyridine (0.12 mL, 1.20 mmol, 1.2 equiv.), 1.3 M isopropylmagnesium(II) chloride lithium chloride in THF (1.1 mL, 1.40 mmol, 1.4 equiv.) and 5-methoxy-5*H*-dibenzo[*b,d*]thiophen-5-ium tetrafluoroborate **1-DBT** (302 mg, 1.00 mmol, 1.0 equiv.) gave sulfonium salt **2b-DBT-Cl** (178 mg, 0.60 mmol, 60%) as a cream solid; **^{1}H NMR** (400 MHz, DMSO-d_6) δ 8.52–8.44 (m, 5H, 3 x ArCH), 8.36 (brd, *J* = 8.1, 1H, ArCH), 8.17 (td, *J* = 7.8, 1.9 Hz, 1H, ArCH), 7.92 (td, *J* = 7.6, 1.1 Hz, 2H, ArCH), 7.74 (td, *J* = 7.7, 1.2 Hz, 2H, ArCH), 7.66 (ddd, *J* = 7.6, 4.7, 1.0 Hz, 1H, ArCH); **^{13}C NMR** (101 MHz, DMSO-d_6) δ 151.8 (ArCH), 150.9 (ArC), 140.5 (ArCH), 140.0 (ArC), 133.7 (ArCH), 131.5 (ArC), 130.9 (ArCH), 128.8 (ArCH), 128.1 (ArCH), 127.6 (ArCH), 124.1 (ArCH); **HRMS** (ESI) $C_{17}H_{12}NS$ $[M]^+$: calculated 262.0685, found 262.0675; $\boldsymbol{v_{max}}$ (thin film/cm^{-1}) 3035, 1576, 1420, 1048, 773, 526, 423.

10-(Pyridin-2-yl)-10*H*-phenoxathiin-10-ium chloride 2b-PXT-Cl

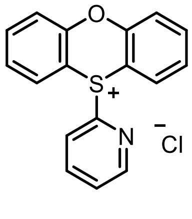

2b-PXT-Cl

Following GP1, with a magnesiation time of 5 h at RT, 2-bromopyridine (0.1 mL, 1.13 mmol, 1.2 equiv.), 1.3 M isopropylmagnesium(II) chloride lithium chloride in THF (0.9 mL, 1.22 mmol, 1.3 equiv.), and 10-methoxy-10*H*-phenoxathiin-10-ium tetrafluoroborate **1-PXT** (300 mg, 0.943 mmol, 1.0 equiv.) gave sulfonium salt **2b-PXT-Cl** (180 mg, 0.573 mmol, 60%) as a beige

solid; ^{1}H NMR (400 MHz, DMSO-d_6) δ 8.58 (ddd, *J* = 4.7, 1.8, 0.8 Hz, 1H, ArCH), 8.47 (dd, *J* = 8.0, 1.6 Hz, 2H, ArCH), 8.25 (dt, *J* = 8.1, 1.1 Hz, 1H, ArCH), 8.16 (td, *J* = 7.7, 1.8 Hz, 1H, ArCH), 7.90 (ddd, *J* = 8.7, 7.3, 1.6 Hz, 2H, ArCH), 7.70 (dd, *J* = 8.4, 1.2 Hz, 2H, ArCH), 7.68 – 7.64 (m, 1H, ArCH), 7.57 (ddd, *J* = 8.3, 7.3, 1.3 Hz, 2H, ArCH); ^{13}C NMR (101 MHz, DMSO-d_6) δ 152.8 (ArC), 152.4 (ArCH), 151.3 (ArC), 140.9 (ArCH), 136.8 (ArCH), 132.9 (ArCH), 128.6 (ArCH), 126.9 (ArCH), 125.7 (ArCH), 120.1 (ArCH), 106.1 (ArC); **HRMS** (ESI) $C_{17}H_{12}ONS$ $[M]^+$: calculated 278.0634, found 278.0623; $\boldsymbol{v_{max}}$ (thin film/cm^{-1}) 3372, 1643, 1466, 1326, 1274, 1229, 1064, 887, 763.

5-(2-fluoropyridin-4-yl)-5*H*-dibenzo[*b,d*]thiophen-5-ium chloride 2c-DBT-Cl

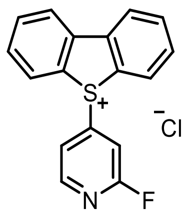

2c-DBT Cl

Following GP1, with a magnesiation time of 3 h at RT, 4-bromo-2-fluoropyridine (66 µL, 0.64 mmol, 1.3 equiv.), 1.3 M isopropylmagnesium(II) chloride lithium chloride in THF (0.6 mL, 0.74 mmol, 1.5 equiv.) and 5-methoxy-5*H*-dibenzo[*b,d*]thiophen-5-ium tetrafluoroborate **1-DBT** (150 mg, 0.50 mmol, 1.0 equiv.) gave sulfonium salt **2c-DBT-Cl** (66 mg, 0.21 mmol, 42%) as a cream solid; ^{1}H NMR (400 MHz, DMSO-d_6) δ 8.64 (d, *J* = 7.9 Hz, 2H, ArCH), 8.54 (dd, *J* = 7.9, 1.2 Hz, 2H, ArCH), 8.41 (d, *J* = 5.5 Hz, 1H, ArCH), 8.05 – 7.94 (m, 3H, ArCH), 7.80 (td, *J* = 7.7, 1.2 Hz, 2H, ArCH), 7.39 (dt, *J* = 5.5, 1.4 Hz, 1H, ArCH); ^{13}C NMR (101 MHz, DMSO-d_6) δ 163.2 (d, $J_{C\text{-}F}$ = 240.4 Hz, ArC), 150.7 (d, $J_{C\text{-}F}$ = 15.3 Hz, ArCH), 147.3 (d, $J_{C\text{-}F}$ = 8.8 Hz, ArC), 140.1 (ArC), 134.6 (ArCH), 131.9 (ArC), 131.7 (ArCH), 129.5 (ArCH), 125.1 (ArCH), 120.3 (d, $J_{C\text{-}F}$ = 4.8 Hz, ArCH), 111.4 (d, $J_{C\text{-}F}$ = 42.8 Hz, ArCH); **^{19}F NMR** (376 MHz, DMSO) δ -63.79; **HRMS** (ESI) $C_{17}H_{11}NFS$ $[M]^+$: calculated 280.0591, found 280.0582; $\boldsymbol{v_{max}}$ (thin film/cm^{-1}) 3061, 1563, 1473, 1389, 1294, 1225, 1025, 877, 759.

5-(4-Fluorophenyl)-5*H*-dibenzo[*b,d*]thiophen-5-ium chloride 2d-DBT-Cl

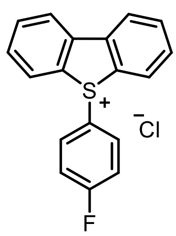

2d-DBT-Cl

Following GP1, with a magnesiation time of 18 h at RT, 1-bromo-4-fluorobenzene (66 μL, 0.60 mmol, 1.2 equiv.), 1.3 M isopropylmagnesium(II) chloride lithium chloride in THF (0.54 mL, 0.70 mmol, 1.4 equiv.) and 5-methoxy-5*H*-dibenzo[*b,d*]thiophen-5-ium tetrafluoroborate **1-DBT** (151 mg, 0.50 mmol, 1.0 equiv.) gave sulfonium salt **2d-DBT-Cl** (122 mg, 0.39 mmol, 78%) as a cream solid; **^{1}H NMR** (400 MHz, DMSO-d$_6$) δ 8.55 (d, *J* = 8.0, Hz, 2H, ArCH), 8.48 (d, *J* = 8.0 Hz, 2H, ArCH), 7.95 (t, *J* = 7.6, Hz, 2H, ArCH), 7.79–7.69 (m, 4H, 2 x ArCH), 7.45 (t, *J* = 8.8 Hz, 2H, ArCH); **^{13}C NMR** (101 MHz, DMSO-d$_6$) δ 164.9 (d, $J_{C–F}$ = 254 Hz, ArC), 139.1 (ArC), 133.9 (ArCH), 133.6 (ArC), 133.1 (d, $J_{C–F}$ = 3 Hz, ArCH), 131.2 (ArCH), 128.5 (ArCH), 124. 9 (d, $J_{C–F}$ = 10 Hz, ArC), 124.5 (ArCH), 118.5 (d, $J_{C–F}$ = 23 Hz, ArCH); **^{19}F NMR** (376 MHz, DMSO-d$_6$) δ -104.18; **HRMS** (ESI) $C_{18}H_{12}FS$ [M]$^+$: calculated 279.0638, found 279.0628; ***ν*** ν_{max} (thin film/cm^{-1}) 3017, 1492, 1227, 1170, 840, 766, 529, 474.

5-(4-Chlorophenyl)-5*H*-dibenzo[*b,d*]thiophen-5-ium chloride 2e-DBT-Cl

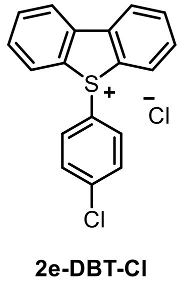

2e-DBT-Cl

Following GP1, with a magnesiation time of 5 h at RT, 1-bromo-4-chlorobenzene (115 mg, 0.60 mmol, 1.2 equiv.), 1.3 M isopropylmagnesium(II) chloride lithium chloride in THF (0.54 mL, 0.70 mmol, 1.4 equiv.) and 5-methoxy-5*H*-dibenzo[*b,d*]thiophen-5-ium tetrafluoroborate

1-DBT (151 mg, 0.50 mmol, 1.0 equiv.) gave sulfonium salt **2e-DBT-Cl** (80 mg, 0.24 mmol, 48%) as a cream solid; **^{1}H NMR** (400 MHz, DMSO-d$_6$) δ 8.53 (d, *J* = 7.8 Hz, 2H, ArCH), 8.42 (d, *J* = 8.0 Hz, 2H, ArCH), 7.96 (t, *J* = 7.1 Hz, 2H, ArCH), 7.76 (t, *J* = 7.1 Hz, 2H, ArCH), 7.69–7.61 (m, 4H, 2 x ArCH); **^{13}C NMR** (101 MHz, DMSO-d$_6$) δ 139.6 (ArC), 139.3 (ArC), 134.3 (ArCH), 133.7 (ArC), 132.1 (ArCH), 131.7 (ArCH), 131.4 (ArCH), 129.0 (ArCH), 128.9 (ArC), 124.9 (ArCH); **HRMS** (ESI) $C_{18}H_{12}ClS$ $[M]^+$: calculated 295.0348, found 295.0356; $\boldsymbol{v}_{max}$ (thin film/cm^{-1}) 2922, 1476, 1392, 1088, 1002, 825, 761, 521, 418.

5-(4-(Trifluoromethyl)phenyl)-5*H*-dibenzo[*b,d*]thiophen-5-ium chloride 2f-DBT-Cl

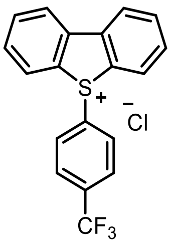

2f-DBT-Cl

Following GP1, with a magnesiation time of 5 h at RT, 4-bromobenzotrifluoride (0.17 mL, 1.20 mmol, 1.2 equiv.), 1.3 M isopropylmagnesium(II) chloride lithium chloride in THF (1.1 mL, 1.40 mmol, 1.4 equiv.) and 5-methoxy-5*H*-dibenzo[*b,d*]thiophen-5-ium tetrafluoroborate **1-DBT** (302 mg, 1.0 mmol, 1.0 equiv.) gave sulfonium salt **2f-DBT-Cl** (216 mg, 0.59 mmol, 60%) as a cream solid; **^{1}H NMR** (500 MHz, DMSO-d$_6$) δ 8.58–8.52 (m, 4H, 2 x ArCH), 8.00–7.93 (m, 4H, 2 x ArCH), 7.90 (d, *J* = 8.6 Hz, 2H, ArCH), 7.77 (ddd, *J* = 8.4, 7.5, 1.2 Hz, 2H, ArCH); **^{13}C NMR** (126 MHz, DMSO-d$_6$) δ 139.4 (ArC), 135.2 (ArC), 134.0 (ArCH), 133.1 (q, $J_{C\text{–}F}$ = 32 Hz ArC), 132.8 (ArC) 131.3 (ArCH), 130.6 (ArCH), 128.7 (ArCH), 127.8 (q, $J_{C\text{–}F}$ = 4 Hz, ArCH), 124.5 (ArCH), 123.1 (q, $J_{C\text{–}F}$ = 273 Hz, CF_3); **^{19}F NMR** (376 MHz, DMSO-d$_6$) δ -61.36; **HRMS** (ESI) $C_{19}H_{12}F_3S$ $[M]^+$: calculated 329.0606, found 329.0600; $\mathbf{v}_{max}$ (thin film/cm^{-1}); 3024, 1602, 1449, 1321, 1170, 1128, 1060, 844, 759, 527, 415.

5-(4-(methoxycarbonyl)phenyl)-5*H*-dibenzo[b,d]thiophen-5-ium chloride (2g-DBT-Cl)

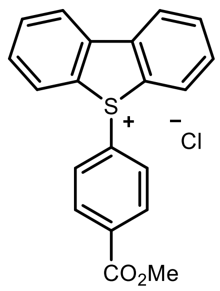

2g-DBT-Cl

Following GP1, with a magnesiation time of 2 h at -40 ˚C, methyl 4-iodobenzoate (104 mg, 0.39 mmol, 1.2 equiv.), 1.3 M isopropylmagnesium(II) chloride lithium chloride in THF (0.36 mL, 0.46 mmol, 1.4 equiv.) and 5-methoxy-5*H*-dibenzo[*b,d*]thiophen-5-ium tetrafluoroborate **1-DBT** (100 mg, 0.33 mmol, 1.0 equiv.) gave sulfonium salt **2g-DBT-Cl** (85 mg, 0.24 mmol, 72%) as a white solid; **^{1}H NMR** (400 MHz, DMSO-d$_6$) δ 8.55 (d, *J* = 7.8 Hz, 2H, ArCH), 8.48 (d, *J* = 8.1 Hz, 2H, ArCH), 8.07 (d, *J* = 8.6 Hz, 2H, ArCH), 7.97 (t, *J* = 7.6 Hz, 2H, ArCH), 7.84 – 7.72 (m, 4H, ArCH), 3.84 (s, 3H, OCH_3).; **^{13}C NMR** (101 MHz, DMSO-d$_6$) δ 165.2 (C=O), 139.8 (ArC), 135.3 (ArC), 134.4 (ArCH), 133.2 (ArC), 131.8 (ArCH), 131.7 (ArCH), 130.4 (ArCH), 129.1 (ArCH), 125.0 (ArCH), 53.2 (OCH_3); **HRMS** (ESI) $C_{20}H_{15}O_2S$ $[M]^+$: calculated 319.0793, found 319.0807; ***v*max** (thin film/cm^{-1}) 3389, 3003, 2159, 1711, 1432, 1399, 1281, 1106, 871, 753, 533, 414.

5-(4-Cyanophenyl)-5*H*-dibenzo[*b,d*]thiophen-5-ium chloride 2h-DBT-Cl

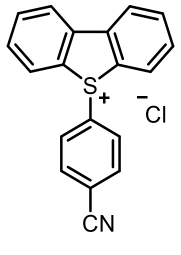

2h-DBT-Cl

Following GP1, with a magnesiation time of 5 h at RT, 4-bromobenzonitrile (91 mg, 0.60 mmol, 1.2 equiv.), 1.3 M isopropylmagnesium(II) chloride lithium chloride in THF (0.54 mL, 0.70

mmol, 1.4 equiv.) and 5-methoxy-5*H*-dibenzo[*b,d*]thiophen-5-ium tetrafluoroborate **1-DBT** (151 mg, 0.50 mmol, 1.0 equiv.) gave sulfonium salt **2h-DBT-Cl** (110 mg, 0.34 mmol, 68%) as a cream solid; **^{1}H NMR** (500 MHz, DMSO-d_6) δ 8.56–8.48 (m, 4H, 2 x ArCH), 8.04 (d, *J* = 8.7 Hz, 2H, ArCH), 7.96 (td, *J* = 7.6, 1.1 Hz, 2H, ArCH), 7.86 (d, *J* = 8.8 Hz, 2H, ArCH), 7.80–7.73 (m, 2H, ArCH); **^{13}C NMR** (126 MHz, DMSO-d_6) δ 139.4 (ArC), 135.7 (ArC), 134.6 (ArCH), 134.0 (ArCH), 132.6 (ArC), 131.3 (ArCH), 130.2 (ArCH), 128.7 (ArCH), 124.6 (ArCH), 117.2 (CN), 115.9 (ArC); **HRMS** (ESI) $C_{19}H_{12}NS$ $[M]^+$: calculated 286.0685, found 286.0677; $\boldsymbol{\nu_{max}}$ (thin film/cm^{-1}) 3008, 2232, 1622, 1490, 1399, 1294, 831, 765, 549, 416.

5-(4-Cyano-3-fluorophenyl)-5*H*-dibenzo[*b,d*]thiophen-5-ium chloride 2i-DBT-Cl

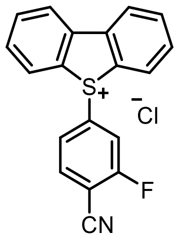

2i-DBT-Cl

Following GP1, with a magnesiation time of 5 h at RT, 4-bromo-2-fluorobenzonitrile (240 mg, 1.20 mmol, 1.2 equiv.), 1.3 M isopropylmagnesium(II) chloride lithium chloride in THF (1.1 mL, 1.40 mmol, 1.4 equiv.) and 5-methoxy-5*H*-dibenzo[*b,d*]thiophen-5-ium tetrafluoroborate **1-DBT** (302 mg, 1.00 mmol, 1.0 equiv.) gave sulfonium salt **2i-DBT-Cl** (156 mg, 0.46 mmol, 46%) as a cream solid. **^{1}H NMR** (400 MHz, DMSO-d_6) δ 8.57 (d, *J* = 8.0, Hz, 2H, ArCH), 8.53 (d, *J* = 8.0, Hz, 2H, ArCH), 8.31 (dd, *J* = 9.0, 2.0 Hz, 1H, ArCH), 8.08 (dd, *J* = 8.4, 6.6 Hz, 1H, ArCH), 7.97 (td, *J* = 7.6, 1.1 Hz, 2H, ArCH), 7.77 (ddd, *J* = 8.4, 7.5, 1.2 Hz, 2H, ArCH), 7.43 (dd, *J* = 8.5, 1.9 Hz, 1H, ArCH); **^{13}C NMR** (101 MHz, DMSO-d_6) δ 161.9 (d, $J_{C–F}$ = 261.6 Hz, ArC), 139.6 (ArC), 138.3 (d, $J_{C–F}$ = 8.3 Hz, ArC), 136.1 (ArCH), 134.1 (ArCH), 132.2 (ArC), 131.3 (ArCH), 128.9 (ArCH), 125.1 (d, $J_{C–F}$ = 3.9 Hz, ArCH), 124. 6 (ArCH), 118.7 (d, $J_{C–F}$ = 24.5 Hz, ArCH), 112.7 (CN), 105.0 (d, $J_{C–F}$ = 15.2 Hz, ArC); **^{19}F NMR** (376 MHz, DMSO-d_6) δ -103.31; **HRMS** (ESI) $C_{19}H_{11}NFS$ $[M]^+$: calculated 304.0591, found 304.0585, $\boldsymbol{\nu_{max}}$ (thin film/cm^{-1}) 2999, 2242, 1603, 1571, 1483, 1415, 1228, 869, 761, 521, 424.

5-(3-Bromophenyl)-5*H*-dibenzo[*b,d*]thiophen-5-ium chloride 2j-DBT-Cl

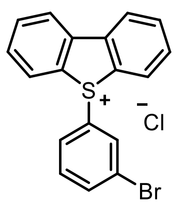

2j-DBT-Cl

Following GP1, with a magnesiation time of 5 h at RT, 1,3-dibromobenzene (73 μL, 0.60 mmol, 1.2 equiv.), 1.3 M isopropylmagnesium(II) chloride lithium chloride in THF (0.54 mL, 0.70 mmol, 1.4 equiv.) and 5-methoxy-5*H*-dibenzo[*b,d*]thiophen-5-ium tetrafluoroborate **1-DBT** (151 mg, 0.50 mmol, 1.0 mmol) gave sulfonium salt **2j-DBT-Cl** (163 mg, 0.44 mmol, 87%) as a cream solid; **^{1}H NMR** (400 MHz, DMSO-d$_6$) δ 8.53 (d, *J* = 7.9, 2H, ArCH), 8.47 (d, *J* = 8.1, 2H, ArCH), 8.25 (t, *J* = 1.9 Hz, 1H, ArCH), 7.96 (td, *J* = 7.6, 1.1 Hz, 2H, ArCH), 7.89 (dd, *J* = 8.1, 1.1, 1H, ArCH), 7.81–7.70 (m, 2H, ArCH), 7.45 (t, *J* = 8.1 Hz, 1H, ArCH), 7.26 (ddd, *J* = 8.2, 2.0, 0.9 Hz, 1H, ArCH); **^{13}C NMR** (101 MHz, DMSO-d$_6$) δ 139.3 (ArC), 136.7 (ArCH), 134.0 (ArCH), 133.2 (ArCH), 133.1 (ArCH), 132.9 (ArC), 131.8 (ArC), 131.3 (ArCH), 128.6 (ArCH), 127.3 (ArCH), 124.6 (ArCH), 122.9 (ArC); **HRMS** (ESI) $C_{18}H_{12}BrS$ [M]$^+$: calculated 338.9843, found 338.9841; ***ν*$_{max}$** (thin film/cm^{-1}) 2922, 1560, 1458, 1062, 767, 666, 533, 424.

10-(3-Bromophenyl)-10*H*-phenoxathiin-10-ium chloride 2j-PXT-Cl

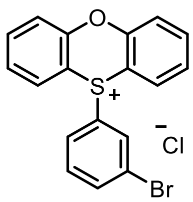

2j-PXT-Cl

Following GP1, with a magnesiation time of 5 h at RT, 1,3-dibromobenzene (0.14 mL, 1.13 mmol, 1.2 equiv.), 1.3 M isopropylmagnesium(II) chloride lithium chloride in THF (1.0 mL, 1.32 mmol, 1.4 equiv.), and 10-methoxy-10*H*-phenoxathiin-10-ium tetrafluoroborate **1-PXT** (300 mg, 0.943 mmol, 1.0 equiv.) gave sulfonium salt **2j-PXT-Cl** (340 mg, 0.867 mmol, 92%) as a

beige solid; **^{1}H NMR** (400 MHz, DMSO-d_6) δ 8.43 (d, *J* = 8.1 Hz, 2H, ArCH), 8.23 (t, *J* = 1.9 Hz, 1H, ArCH), 7.96 – 7.88 (m, 3H, ArCH), 7.74 (dd, *J* = 8.4, 1.2 Hz, 2H, ArCH), 7.60 (ddd, *J* = 8.4, 7.3, 1.3 Hz, 2H, ArCH), 7.55 (t, *J* = 8.0 Hz, 1H, ArCH), 7.49 (ddd, *J* = 8.2, 2.0, 1.0 Hz, 1H, ArCH); **^{13}C NMR** (101 MHz, DMSO-d_6) δ 150.7 (ArC), 137.0 (ArCH), 136.9 (ArCH), 135.3 (ArC), 133.7 (ArCH), 132.5 (ArCH), 131.9 (ArCH), 127.9 (ArCH), 127.3 (ArCH), 123.7 (ArC), 120.4 (ArCH), 106.8 (ArC); **HRMS** (ESI) $C_{18}H_{12}OBrS$ [M]$^+$: calculated 354.9787, found 354.9783; ***v*$_{max}$** (thin film/cm^{-1}) 3376, 3060, 1590, 1459, 1271, 1226, 1062, 992, 765, 651, 533, 470.

5-(3-(Trifluoromethyl)phenyl)-5*H*-dibenzo[*b,d*]thiophen-5-ium chloride 2k-DBT-Cl

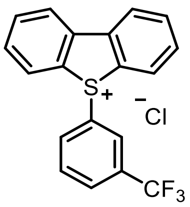

2k-DBT-Cl

Following GP1, with a magnesiation time of 5 h at RT, 3-bromobenzotrifluoride (84 µL, 0.60 mmol, 1.2 equiv.), 1.3 M isopropylmagnesium(II) chloride lithium chloride in THF (0.54 mL, 0.70 mmol, 1.4 equiv.) and 5-methoxy-5*H*-dibenzo[b,d]thiophen-5-ium tetrafluoroborate **1-DBT** (151 mg, 0.50 mmol) gave sulfonium salt **2k-DBT-Cl** (132 mg, 0.36 mmol, 72%) as a cream solid; **^{1}H NMR** (500 MHz, DMSO-d_6) δ 8.66 (brs, 1H, ArCH), 8.54 (d, *J* = 9.1 Hz, 2H, ArCH), 8.49 (d, *J* = 9.3 Hz, 2H, ArCH), 8.07 (d, *J* = 7.8 Hz, 1H, ArCH), 7.97 (td, *J* = 7.6, 1.1 Hz, 2H, ArCH), 7.77 (ddd, *J* = 8.4, 7.5, 1.2 Hz, 2H, ArCH), 7.70 (t, *J* = 8.0 Hz, 1H, ArCH), 7.37 (d, *J* = 5.9 Hz, 1H, ArCH); **^{13}C NMR** (126 MHz, DMSO-d_6) δ 139.4 (ArC), 134.0 (ArCH), 133.0 (ArC), 132.4 (ArCH), 131.8 (ArC), 131.7 (ArCH), 131.3 (ArCH), 130.6 (q, $J_{C\text{–}F}$ = 33.2 Hz, ArC), 130.5 (m, ArCH), 128.9 (m, ArCH), 128.7 (ArCH), 124.6 (ArCH), 122.60 (q, $J_{C\text{–}F}$ = 272.1 Hz, CF_3); **^{19}F NMR** (376 MHz, DMSO-d_6) δ -61.36; **HRMS** (ESI) $C_{19}H_{12}F_3S$ [M]$^+$: calculated 329.0606, found 329.0605; ***v*$_{max}$** (thin film/cm^{-1}); 2922, 1425, 1309, 1169, 1064, 768, 687, 527, 228.

5-(3-Chlorophenyl)-5*H*-dibenzo[*b,d*]thiophen-5-ium chloride 2l-DBT-Cl

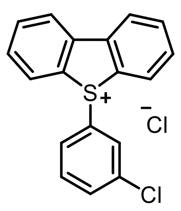

2l-DBT-Cl

Following GP1, with a magnesiation time of 5 h at RT, 1-bromo-3-chlorobenzene (0.14 mL, 1.20 mmol, 1.2 equiv.), 1.3 M isopropylmagnesium(II) chloride lithium chloride in THF (1.1 mL, 1.40 mmol, 1.4 equiv.) and 5-methoxy-5*H*-dibenzo[*b,d*]thiophen-5-ium tetrafluoroborate **1-DBT** (302 mg, 1.00 mmol, 1.0 equiv.) gave sulfonium salt **2l-DBT-Cl** (298 mg, 0.90 mmol, 90%) as a cream solid; **^{1}H NMR** (400 MHz, DMSO-d$_6$) δ 8.53 (d, *J* = 8.0 Hz, 2H, ArCH), 8.48 (d, *J* = 8.2 Hz, 2H, ArCH), 8.12 (t, *J* = 2.0 Hz, 1H, ArCH), 7.96 (td, *J* = 7.6, 1.1 Hz, 2H, ArCH), 7.81–7.72 (m, 3H, 2 x ArCH), 7.54 (t, *J* = 8.1 Hz, 1H, ArCH), 7.26 (ddd, *J* = 8.2, 2.0, 0.9 Hz, 1H, ArCH); **^{13}C NMR** (101 MHz, DMSO-d$_6$) δ 139.3 (ArC), 134.7 (ArC), 134.0 (ArCH), 133.8 (ArCH), 132.82 (ArCH), 132.80 (ArC), 131.7 (ArC), 131.3 (ArCH), 130.3 (ArCH), 128.6 (ArCH), 127.0 (ArCH), 124.6 (ArCH); **HRMS** (ESI) $C_{18}H_{12}ClS$ [M]$^+$: 295.0348, found 329.0355; ***ν*$_{max}$** (thin film/cm^{-1}) 2918, 1571, 1459, 1151, 762, 666, 527, 419.

5-(3-(Difluoromethoxy)phenyl)-5*H*-dibenzo[b,d]thiophen-5-ium chloride 2m-DBT-Cl

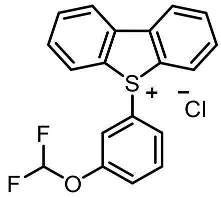

2m-DBT-Cl

Following GP1, with a magnesiation time of 18 h at 37 °C, 1-bromo-3-(difluoromethoxy)benzene (0.17 mL, 1.20 mmol, 1.2 equiv.), 1.3 M isopropylmagnesium(II) chloride lithium chloride in THF (1.1 mL, 1.40 mmol mmol, 1.4 equiv.) and 5-methoxy-5*H*-dibenzo[*b,d*]thiophen-5-ium tetrafluoroborate **1-DBT** (302 mg, 1.00 mmol, 1.0 equiv.) gave

sulfonium salt **2m-DBT-Cl** (179 mg, 0.57 mmol, 57%) as a cream solid; **^{1}H NMR** (500 MHz, DMSO-d_6) δ 8.54 (d, *J* = 7.8 Hz, 2H, ArCH), 8.50 (d, *J* = 8.1 Hz, 2H, ArCH), 7.97 (t, *J* = 7.6 Hz, 2H, ArCH), 7.88 (t, *J* = 2.2 Hz, 1H, ArCH), 7.81–7.73 (m, 2H, ArCH), 7.59 (t, *J* = 8.2 Hz, 1H, ArCH), 7.55–7.49 (m, 1H, ArCH), 7.39 (t, *J* = 73.0 Hz, 1H, $OCHCF_2$), 7.18 (dd, *J* = 7.8, 1.9 Hz, 1H, ArCH); **^{13}C NMR** (126 MHz, DMSO-d_6) δ 151.5 (t, *J* = 3.5 Hz, ArC), 139.3 (ArC), 134.0 (ArCH), 132.8 (ArCH, ArC), 131.4 (ArC), 131.3 (ArCH), 128.6 (ArCH), 124.6 (ArCH), 124.5 (ArCH), 123.5 (ArCH), 120.9 (ArCH), 115.91 (t, $J_{C–F}$ = 260.1 Hz, $OCHCF_2$); **^{19}F NMR** (376 MHz, DMSO) δ -83.32 (d, *J* = 72.9 Hz); **HRMS** (ESI) $C_{19}H_{13}OF_2S$ [M]$^+$: calculated 327.0650, found 327.0655; $\boldsymbol{v}_{\mathbf{max}}$ (thin film/cm^{-1}) 2982, 1592, 1474, 1292, 1116, 1053, 776, 523, 424.

5-(3-Cyano-2-fluorophenyl)-5*H*-dibenzo[*b,d*]thiophen-5-ium chloride 2n-DBT-Cl

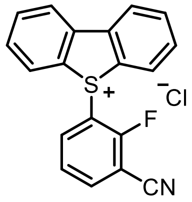

2n-DBT-Cl

Following GP1, with a magnesiation time of 5 h at RT, 3-bromo-2-fluorobenzonitrile (120 mg, 0.60 mmol, 1.2 equiv.), 1.3 M Isopropylmagnesium(II) chloride lithium chloride in THF (0.54 mL, 0.70 mmol, 1.4 equiv.) and 5-methoxy-5*H*-dibenzo[*b,d*]thiophen-5-ium tetrafluoroborate **1-DBT** (151 mg, 0.50 mmol, 1.0 equiv.) gave sulfonium salt **2n-DBT-Cl** (108 mg, 0.33 mmol, 65%) as a cream solid; **^{1}H NMR** (500 MHz, DMSO-d_6) δ 8.56 (d, *J* = 7.9 Hz, 2H, ArCH), 8.48 (d, *J* = 8.1 Hz, 2H, ArCH), 8.30 (ddd, *J* = 7.8, 6.1, 1.7 Hz, 1H, ArCH), 7.98 (t, *J* = 7.6 Hz, 2H, ArCH), 7.77 (t, *J* = 7.8 Hz, 2H, ArCH), 7.69 (ddd, *J* = 8.4, 6.8, 1.7 Hz, 1H, ArCH), 7.54 (t, *J* = 8.1 Hz, 1H, ArCH); **^{13}C NMR** (101 MHz, DMSO-d_6) δ 161.3 (d, $J_{C–F}$ = 265.3 Hz, ArC), 139.8 (ArC), 139.7 (ArCH), 136.4 (ArCH), 134.2 (ArCH), 131.4 (ArCH), 131.3 (ArC), 128.6 (ArCH), 127.5 (d, $J_{C–F}$= 4.1 Hz, ArCH), 124.6 (ArCH), 118.6 (d, $J_{C–F}$ = 12.7 Hz, ArC), 112.4 (CN), 102.8 (d, $J_{C–F}$ = 14.2 Hz, ArC); **^{19}F NMR** (471 MHz, DMSO-d_6) δ -103.84; **HRMS** (ESI) $C_{19}H_{11}NFS$

[M]$^+$: calculated 304.0591, found 304.0587; ***v*~max~** (thin film/cm^{-1}) 2982, 2239, 1599, 1466, 1449, 1238, 780, 589, 424.

5-(3-(*N,N*-Dimethylsulfamoyl)phenyl)-5*H*-dibenzo[*b,d*]thiophen-5-ium chloride 2o-DBT-Cl

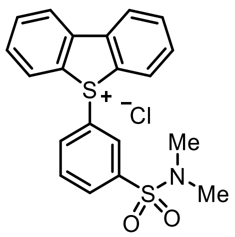

2o-DBT-Cl

Following GP1 with a magnesiation time of 18 h at 37 °C, 3-bromo-*N,N*-dimethylbenzenesulfonamide (158 mg, 0.60 mmol, 1.2 equiv.), 1.3 M isopropylmagnesium(II) chloride lithium chloride in THF (0.54 mL, 0.70 mmol, 1.4 equiv.) and 5-methoxy-5*H*-dibenzo[*b,d*]thiophen-5-ium tetrafluoroborate **1-DBT** (151 mg, 0.50 mmol, 1.0 equiv.) gave sulfonium salt **2o-DBT-Cl** (139 mg, 0.35 mmol, 69%) as a cream solid; **^{1}H NMR** (400 MHz, DMSO-d$_6$) δ 8.55 (dd, *J* = 7.8, 1.2 Hz, 2H, ArCH), 8.52–8.43 (m, 3H, 2 x ArCH), 8.04–7.94 (m, 3H, 2 x ArCH), 7.81–7.69 (m, 3H, 2 x ArCH), 7.44 (ddd, *J* = 8.3, 2.1, 1.0 Hz, 1H, ArCH), 2.63 (s, 6H, NCH$_3$); **^{13}C NMR** (101 MHz, DMSO-d$_6$) δ 139.3 (ArC), 136.9 (ArC), 134.0 (ArCH), 133.2 (ArC), 132.5 (ArCH), 132.1 (ArCH), 132.0 (ArCH), 131.9 (ArC), 131.3 (ArCH), 130.4 (ArCH), 128.6 (ArCH), 124.5 (ArCH), 37.5 (NCH$_3$); **HRMS** (ESI) $C_{20}H_{18}O_2NS_2$ [M]$^+$: calculated 368.0773, found 368.0775; ***v*~max~** (thin film/cm^{-1}) 2992, 1461, 1446, 1407, 1346, 1165, 954, 770, 712, 579, 486, 430.

5-Phenyl-5*H*-dibenzo[*b,d*]thiophen-5-ium chloride 2p-DBT-Cl

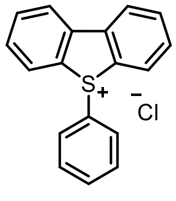

2p-DBT-Cl

Following GP1, with a magnesiation time of 18 h at 37 °C, bromobenzene (0.13 mL, 1.20 mmol, 1.2 equiv.), 1.3 M isopropylmagnesium(II) chloride lithium chloride in THF (1.1 mL, 1.40 mmol, 1.4 equiv.) and 5-methoxy-5*H*-dibenzo[*b,d*]thiophen-5-ium tetrafluoroborate **1-DBT** (302 mg, 1.00 mmol, 1.0 equiv.) gave sulfonium salt **2p-DBT-Cl** (268 mg, 0.90 mmol, 90%) as a cream solid; **^{1}H NMR** (500 MHz, DMSO-d_6) δ 8.54 (dd, *J* = 7.9, 1.2 Hz, 2H, ArCH), 8.44 (dd, *J* = 8.2, 1.1 Hz, 2H, ArCH), 7.95 (td, *J* = 7.6, 1.0 Hz, 2H, ArCH), 7.75 (ddd, *J* = 8.4, 7.4, 1.2 Hz, 2H, ArCH), 7.70–7.67 (m, 1H, ArCH), 7.64 (dd, *J* = 7.3, 1.7 Hz, 2H, ArCH), 7.62–7.54 (m, 2H, ArCH); **^{13}C NMR** (126 MHz, DMSO-d_6) δ 139.2 (ArC), 133.9 (ArCH), 133.8 (ArCH), 133.2 (ArC), 131.23 (ArCH), 131.15 (ArCH), 129.7 (ArCH), 129.3 (ArC), 128.5 (ArCH), 124.5 (ArCH); **HRMS** (ESI) $C_{18}H_{13}S$ $[M]^+$: calculated 261.0725, found 261.0732; ***v*max** (thin film/cm^{-1}) 2919, 1481, 1448, 1047, 755, 707, 526, 421.

5-(*o*-Tolyl)-5*H*-dibenzo[*b,d*]thiophen-5-ium chloride 2q-DBT-Cl

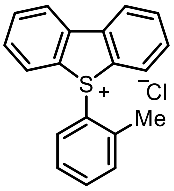

2q-DBT-Cl

Following GP1, with a magnesiation time of 18 h at 37 °C, 2-bromotoluene (0.14 mL, 1.20 mmol, 1.2 equiv.), 1.3 M isopropylmagnesium(II) chloride lithium chloride in THF (1.1 mL, 1.40 mmol, 1.4 equiv.) and 5-methoxy-5*H*-dibenzo[*b,d*]thiophen-5-ium tetrafluoroborate **1-DBT** (302 mg, 1.00 mmol, 1.0 equiv.) gave sulfonium salt **2q-DBT-Cl** (149 mg, 0.48 mmol, 48%) as a cream solid; **^{1}H NMR** (500 MHz, DMSO-d_6) δ 8.56 (dd, *J* = 7.9, 1.2 Hz, 2H, ArCH), 8.38 (dd, *J* = 8.2, 1.1 Hz, 2H, ArCH), 7.97 (td, *J* = 7.6, 1.0 Hz, 2H, ArCH), 7.75 (ddd, *J* = 8.3, 7.4, 1.2 Hz, 2H, ArCH), 7.64 (d, *J* = 7.8 Hz, 1H, ArCH), 7.59 (t, *J* = 7.5 Hz, 1H, ArCH), 7.26–7.19 (m, 1H, ArCH), 6.55 (d, *J* = 8.2 Hz, 1H, ArCH), 3.01 (s, 3H, CH_3); **^{13}C NMR** (126 MHz, DMSO-d_6) δ 142.1 (ArC), 139.2 (ArC), 134.2 (ArCH), 133.9 (ArCH), 133.0 (ArCH), 132.9 (ArCH), 131.4 (ArCH), 129.2 (ArCH), 128.2 (ArCH), 127.9 (ArC), 127.0 (ArC), 124.6 (ArCH), 19.9 (CH-

3). **HRMS** (ESI) $C_{19}H_{15}S$ $[M]^+$: calculated 275.0894, found 275.0905, ν_{max} (thin film/cm^{-1}) 2921, 1605, 1448, 1206, 1052, 756, 521, 423.

5-(2-Methoxyphenyl)-5*H*-dibenzo[*b,d*]thiophen-5-ium chloride 2r-DBT-Cl

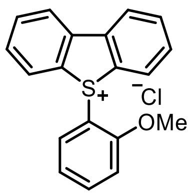

2r-DBT-Cl

Following GP1, with a magnesiation time of 18 h at 37 °C, 2-bromoanisole (75 µL, 0.60 mmol, 1.2 equiv.), 1.3 M isopropylmagnesium(II) chloride lithium chloride in THF (0.54 mL, 0.70 mmol, 1.4 equiv.) and 5-methoxy-5*H*-dibenzo[*b,d*]thiophen-5-ium tetrafluoroborate **1-DBT** (151 mg, 0.50 mmol, 1.0 equiv.) gave sulfonium salt **2r-DBT-Cl** (105 mg, 0.32 mmol, 64%) as a cream solid; **^{1}H NMR** (400 MHz, DMSO-d$_6$) δ 8.53 (dd, *J* = 7.9, 1.2 Hz, 2H, ArCH), 8.32 (dd, *J* = 8.1, 1.1 Hz, 2H, ArCH), 7.96 (td, *J* = 7.6, 1.1 Hz, 2H, ArCH), 7.77 (ddd, *J* = 8.4, 7.5, 1.2 Hz, 2H, ArCH), 7.70 (ddd, *J* = 8.7, 7.3, 1.6 Hz, 1H, ArCH), 7.44 (dd, *J* = 8.5, 1.2 Hz, 1H, ArCH), 7.05 (ddd, *J* = 8.4, 7.4, 1.1 Hz, 1H, ArCH), 6.85 (dd, *J* = 8.1, 1.6 Hz, 1H, ArCH), 4.05 (s, 3H, OCH_3); **^{13}C NMR** (101 MHz, DMSO-d$_6$) δ 158.4 (ArC), 139.5 (ArC), 136.0 (ArCH), 133.8 (ArCH), 131.21 (ArCH), 131.17 (ArC), 128.2 (ArCH), 128.0 (ArCH), 124.5 (ArCH), 122.8 (ArCH), 116.1 (ArC), 114.1 (ArCH), 57.4 (OCH_3); **HRMS** (ESI) $C_{19}H_{15}OS$ $[M]^+$: calculated 291.0838, found 291.0833; ν_{max} (thin film/cm^{-1}) 2921, 1584, 1481, 1284, 1164, 707, 532, 419.

5-(2-Methylthiophenyl)-5*H*-dibenzo[*b,d*]thiophen-5-ium chloride 2s-DBT-Cl

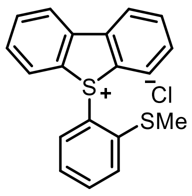

2s-DBT-Cl

Following GP1, with a magnesiation time of 18 h at 37 °C, 2-bromothioanisole (0.32 mL, 2.40 mmol, 1.2 equiv.), 1.3 M isopropylmagnesium(II) chloride lithium chloride in THF (2.2 mL, 2.80 mmol, 1.4 equiv.) and 5-methoxy-5*H*-dibenzo[*b,d*]thiophen-5-ium tetrafluoroborate **1-DBT** (604 mg, 2.00 mmol, 1.0 equiv.) gave sulfonium salt **2s-DBT-Cl** (624 mg, 1.81 mmol, 91%) as a cream solid; **^{1}H NMR** (400 MHz, DMSO-d_6) δ 8.57 (d, *J* = 7.9 Hz, 2H, ArCH), 8.42 (d, *J* = 8.0 Hz, 2H, ArCH), 7.97 (t, *J* = 7.7 Hz, 2H, ArCH), 7.88 (d, *J* = 8.0 Hz, 1H, ArCH), 7.78 (t, *J* = 7.8 Hz, 2H, ArCH), 7.66 (t, *J* = 7.8 Hz, 1H, ArCH), 7.30–7.21 (m, 1H, ArCH), 6.56 (brs, 1H, ArCH), 2.86 (s, 3H, SCH_3); **^{13}C NMR** (101 MHz, DMSO-d_6) δ 141.4 (ArC), 139.4 (ArC), 134.3 (ArCH), 134.0 (ArCH), 131.9 (ArC), 131.5 (ArCH), 131.5 (ArCH), 130.3 (ArC), 129.1 (ArCH), 128.0 (ArCH), 126.4 (ArCH), 124.7 (ArCH), 17.9 (SCH_3); **HRMS** (ESI) $C_{19}H_{15}S_2$ $[M]^+$: calculated 307.0610, found 307.0604; ***ν*max** (thin film/cm^{-1}) 2983, 1571, 1439, 1251, 1163, 973, 804, 765, 534, 417.

5-([1,1'-Biphenyl]-2-yl)-5*H*-dibenzo[*b,d*]thiophen-5-ium chloride 2t-DBT-Cl

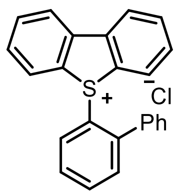

2t-DBT-Cl

Following GP1, with a magnesiation time of 18 h at 37 °C, 2-bromobiphenyl (0.14 mL, 0.60 mmol, 1.2 equiv.), 1.3 M isopropylmagnesium(II) chloride lithium chloride in THF (0.54 mL, 0.70 mmol, 1.4 equiv.) and 5-methoxy-5*H*-dibenzo[*b,d*]thiophen-5-ium tetrafluoroborate **1-DBT** (151 mg, 0.50 mmol, 1.0 equiv.) gave sulfonium salt **2t-DBT-Cl** (125 mg, 0.34 mmol, 67%) as a cream solid; **^{1}H NMR** (500 MHz, DMSO-d_6) δ 8.51 (dd, *J* = 7.9, 1.2 Hz, 2H, ArCH), 8.08 (d, *J* = 8.0 Hz, 2H, ArCH), 8.00–7.91 (m, 4H, 2 x ArCH), 7.81 (td, *J* = 7.5, 1.2 Hz, 1H, ArCH), 7.74 (td, *J* = 7.8, 1.3 Hz, 3H, 2 x ArCH), 7.70–7.59 (m, 3H, 2 x ArCH), 7.49 (ddd, *J* = 8.6, 7.3, 1.6 Hz, 1H, ArCH), 6.86 (d, *J* = 8.3 Hz, 1H, ArCH); **^{13}C NMR** (126 MHz, DMSO-d_6) δ 145.3 (ArC), 139.2 (ArC), 136.4 (ArC), 134.4 (ArCH), 134.0 (ArCH), 132.9 (ArCH), 132.6

(ArC), 131.3 (ArCH), 130.9 (ArCH), 130.4 (ArCH), 129.3 (ArCH), 129.2 (ArCH), 127.8 (ArCH), 127.4 (ArCH), 127.1 (ArC), 124.8 (ArCH); **HRMS** (ESI) $C_{24}H_{17}S$ $[M]^+$: calculated 337.1045, found 337.1040; $\boldsymbol{v}_{\mathbf{max}}$ (thin film/cm^{-1}) 2981, 1460, 1161, 1061, 922, 757, 538, 430.

5-(2-Allylphenyl)-5*H*-dibenzo[*b,d*]thiophen-5-ium chloride 2u-DBT-Cl

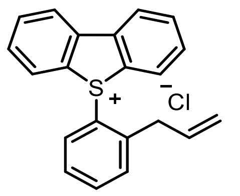

2u-DBT-Cl

Following GP1, with a magnesiation time of 18 h at 37 °C, 1-allyl-2-bromobenzene (0.18 mL, 1.20 mmol, 1.2 equiv.), 1.3 M isopropylmagnesium(II) chloride lithium chloride in THF (1.1 mL, 1.40 mmol, 1.4 equiv.) and 5-methoxy-5*H*-dibenzo[*b,d*]thiophen-5-ium tetrafluoroborate **1-DBT** (302 mg, 1.00 mmol, 1.0 equiv.) gave sulfonium salt **2u-DBT-Cl** (160 mg, 0.47 mmol, 47%) as a cream solid; **^{1}H NMR** (500 MHz, DMSO-d_6) δ 8.58 (d, *J* = 7.8 Hz, 2H, ArCH), 8.25 (d, *J* = 8.4 Hz, 2H, ArCH), 7.98 (t, *J* = 7.6 Hz, 2H, ArCH), 7.76 (t, *J* = 7.8 Hz, 2H, ArCH), 7.70–7.62 (m, 2H, 2 x ArCH), 7.27 (ddd, *J* = 8.5, 6.4, 2.3 Hz, 1H, ArCH), 6.56 (d, *J* = 8.2 Hz, 1H, ArCH), 6.32 (ddt, *J* = 16.7, 10.1, 6.4 Hz, 1H, –CH=), 5.44 (dd, *J* = 10.1, 1.7 Hz, 1H, =CH_2 (cis)), 5.33 (dq, *J* = 17.0, 1.6 Hz, 1H, =CH_2(trans)), 4.25 (d, *J* = 6.4 Hz, 2H, CH_2); **^{13}C NMR** (126 MHz, DMSO-d_6) δ 144.5 (ArC), 139.3 (ArC), 137.4 (–CH=), 134.6 (ArCH), 134.0 (ArCH), 133.5 (ArC), 132.3 (ArCH), 131.4 (ArCH), 130.1 (ArCH), 128.0 (ArCH), 127.9 (ArCH), 127.3 (ArC), 124.8 (ArCH), 118.7 (=CH_2), 37.2 (CH_2); **HRMS** (ESI) $C_{21}H_{17}S$ $[M]^+$: calculated 301.1045, found 301.1043; $\boldsymbol{v}_{\mathbf{max}}$ (thin film/cm^{-1}) 2916, 1445, 1156, 998, 757, 529, 415.

5-(2-(1,3-Dioxolan-2-yl)phenyl)-5*H*-dibenzo[*b,d*]thiophen-5-ium chloride 2v-DBT-Cl

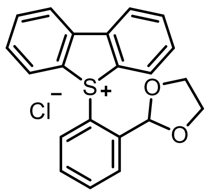

2v-DBT-Cl

Following GP1, with a magnesiation time of 18 h at 37 °C, 2-(2-bromophenyl)-1,3-dioxolane (90 µL, 0.60 mmol, 1.2 equiv.), 1.3 M isopropylmagnesium(II) chloride lithium chloride in THF (0.54 mL, 0.70 mmol, 1.4 equiv.) and 5-methoxy-5*H*-dibenzo[*b,d*]thiophen-5-ium tetrafluoroborate **1-DBT** (151 mg, 0.50 mmol, 1.0 equiv.) gave sulfonium salt **2v-DBT-Cl** (74 mg, 0.20 mmol, 40%) as a cream solid; **^{1}H NMR** (400 MHz, DMSO-d$_6$) δ 8.58 (dd, *J* = 7.9, 1.2 Hz, 2H, ArCH), 8.28–8.20 (m, 2H, ArCH), 7.98 (td, *J* = 7.6, 1.0 Hz, 2H, ArCH), 7.88 (dd, *J* = 7.7, 1.5 Hz, 1H, ArCH), 7.80–7.68 (m, 3H, 2 x ArCH), 7.47–7.39 (m, 1H, ArCH), 6.71 (s, 1H, CH), 6.65 (dd, *J* = 8.2, 1.1 Hz, 1H, ArCH), 4.44–4.35 (m, 2H, CH$_2$), 4.32–4.21 (m, 2H, CH$_2$); **^{13}C NMR** (101 MHz, DMSO-d$_6$) δ 141.1 (ArC), 139.4 (ArC), 134.2 (ArCH), 134.0 (ArCH), 133.2 (ArC), 132.5 (ArCH), 131.5 (ArCH), 129.2 (ArCH), 128.01 (ArCH), 127.95 (ArCH), 127.2 (ArC), 124.7 (ArCH), 101.1 (CH), 65.6 (CH$_2$); **HRMS** (ESI) $C_{21}H_{11}O_2S$ [M]$^+$: calculated 333.0944, found 333.0949; ***ν*$_{max}$** (thin film/cm^{-1}) 2929, 1586, 1444, 1286, 1166, 1064, 1022, 752, 556, 417.

5-(2,6-Dimethoxyphenyl)-5*H*-dibenzo[*b,d*]thiophen-5-ium chloride 2w-DBT-Cl

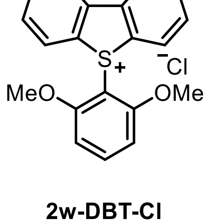

2w-DBT-Cl

Following GP1, with a magnesiation time of 18 h at 37 °C, 2-bromo-1,3-dimethoxybenzene (260 mg, 1.20 mmol, 1.2 equiv.), 1.3 M isopropylmagnesium(II) chloride lithium chloride in

THF (1.1 mL, 1.40 mmol, 1.4 equiv.) and 5-methoxy-5*H*-dibenzo[*b,d*]thiophen-5-ium tetrafluoroborate **1-DBT** (302 mg, 1.00 mmol, 1.0 equiv.) gave sulfonium salt **2w-DBT-Cl** (250 mg, 0.70 mmol, 70%) as a cream solid; **^{1}H NMR** (400 MHz, DMSO-d$_6$) δ 8.51 (d, *J* = 7.8 Hz, 2H, ArCH), 8.17 (d, *J* = 8.1 Hz, 2H, ArCH), 7.91 (t, *J* = 7.6 Hz, 2H, ArCH), 7.75 (t, *J* = 8.5 Hz, 1H, ArCH), 7.70 (dd, *J* = 8.4, 7.4 Hz, 2H, ArCH), 6.90 (brs, 2H, ArCH), 3.60 (s, 6H, OCH$_3$); **^{13}C NMR** (101 MHz, DMSO-d$_6$) δ 160.9 (ArC), 140.0 (ArC), 138.6 (ArCH), 133.1 (ArCH), 130.8 (ArCH), 130.6 (ArC), 126.7 (ArCH), 123.8 (ArCH), 106.3 (ArCH), 97.9 (ArC), 57.1 (OCH$_3$); **HRMS** (ESI) $C_{20}H_{17}O_2S$ [M]$^+$: calculated 321.0938, found 321.0944; ***v*$_{max}$** (thin film/cm^{-1}) 2992, 1574, 1477, 1263, 1097, 891, 767, 517, 420.

5-(*m*-Tolyl)-5*H*-dibenzo[*b,d*]thiophen-5-ium chloride 2x-DBT-Cl

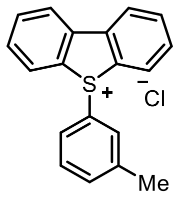

2x-DBT-Cl

Following GP1, with a magnesiation time of 18 h at 37 °C, 3-bromotoluene (0.15 mL, 1.20 mmol, 1.2 equiv.), 1.3 M isopropylmagnesium(II) chloride lithium chloride in THF (1.1 mL, 1.40 mmol, 1.4 equiv.) and 5-methoxy-5*H*-dibenzo[*b,d*]thiophen-5-ium tetrafluoroborate **1-DBT** (302 mg, 1.00 mmol, 1.0 equiv.) gave sulfonium salt **2x-DBT-Cl** (178 mg, 0.57 mmol, 57%) as a cream solid; **^{1}H NMR** (500 MHz, DMSO-d$_6$) δ 8.53 (dd, *J* = 7.9, 1.2 Hz, 2H, ArCH), 8.40 (dd, *J* = 8.1, 1.1 Hz, 2H, ArCH), 7.96 (td, *J* = 7.6, 1.1 Hz, 2H, ArCH), 7.75 (ddd, *J* = 8.4, 7.4, 1.2 Hz, 2H, ArCH), 7.51 (m, 2 x ArCH), 7.49–7.43 (m, 1H, ArCH), 7.35 (d, *J* = 8.2 Hz, 1H, ArCH), 2.30 (s, 3H, CH$_3$); **^{13}C NMR** (126 MHz, DMSO-d$_6$) δ 141.2 (ArC), 139.2 (ArC), 134.7 (ArCH), 133.8 (ArCH), 133.0 (ArC), 131.2 (ArCH), 131.0 (ArCH), 129.8 (ArCH), 128.8 (ArC), 128.4 (ArCH), 126.5 (ArCH), 124.5 (ArCH), 20.8 (CH$_3$); **HRMS** (ESI) $C_{19}H_{15}S$ [M]$^+$: calculated 275.0894, found 275.0908; ***v*$_{max}$** (thin film/cm^{-1}) 2995, 1449, 1296, 1163, 1095, 792, 743, 517, 431.

5-(3-Methoxyphenyl)-5*H*-dibenzo[*b,d*]thiophen-5-ium chloride 2y-DBT-Cl

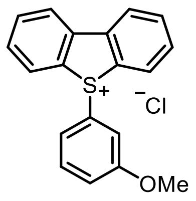

2y-DBT-Cl

Following GP1, with a magnesiation time of 18 h at 37 °C, 3-bromoanisole (76 µL, 0.60 mmol, 1.2 equiv.), 1.3 M isopropylmagnesium(II) chloride lithium chloride in THF (0.54 mL, 0.70 mmol, 1.4 equiv.) and 5-methoxy-5*H*-dibenzo[*b,d*]thiophen-5-ium tetrafluoroborate **1-DBT** (151 mg, 0.50 mmol, 1.0 equiv.) gave sulfonium salt **2y-DBT-Cl** (109 mg, 0.34 mmol, 67%) as an off-white solid; **^{1}H NMR** (400 MHz, DMSO-d$_6$) δ 8.52 (d, *J* = 7.9 Hz, 2H, ArCH), 8.44 (d, *J* = 8.1 Hz, 2H, ArCH), 7.96 (td, *J* = 7.6, 1.1 Hz, 2H, ArCH), 7.76 (ddd, *J* = 8.4, 7.5, 1.2 Hz, 2H, ArCH), 7.58 (t, *J* = 2.2 Hz, 1H, ArCH), 7.44 (t, *J* = 8.2 Hz, 1H, ArCH), 7.26 (ddd, *J* = 8.4, 2.6, 0.8 Hz, 1H, ArCH), 6.81 (ddd, *J* = 8.0, 2.0, 0.8 Hz, 1H, ArCH), 3.80 (s, 3H, OCH$_3$); **^{13}C NMR** (126 MHz, DMSO-d$_6$) δ 160.2 (ArC), 139.2 (ArC), 133.9 (ArCH), 132.9 (ArC), 132.2 (ArCH), 131.2 (ArCH), 130.5 (ArC), 128.6 (ArCH), 124.5 (ArCH), 119.6 (ArCH), 119.1 (ArCH), 116.6 (ArCH), 56.0 (OCH$_3$); **HRMS** (ESI) $C_{19}H_{15}OS$ [M]$^+$: calculated 291.0838, found 291.0832; ***v*$_{max}$** (thin film/cm^{-1}) 2852, 1458, 1426, 1289, 1235, 1025, 865.

5-(3,5-Dimethoxyphenyl)-5*H*-dibenzo[*b,d*]thiophen-5-ium chloride 2z-DBT-Cl

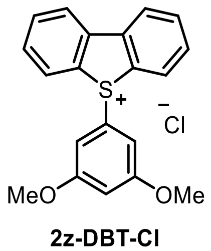

2z-DBT-Cl

Following GP1, with a magnesiation time of 18 h at 37 °C, (260 mg, 1.20 mmol, 1.2 equiv.), 1.3 M Isopropylmagnesium(II) chloride lithium chloride in THF (1.1 mL, 1.40 mmol, 1.4 equiv.) and 5-methoxy-5*H*-dibenzo[*b,d*]thiophen-5-ium tetrafluoroborate **1-DBT** (302 mg, 1.00 mmol,

1.0 equiv.) gave sulfonium salt **2z-DBT-Cl** (208 mg, 0.58 mmol, 58%) as an off-white solid; **^{1}H NMR** (500 MHz, DMSO-d$_6$) δ 8.51 (m, 4H, 2 x ArCH), 7.94 (td, *J* = 7.6, 1.1 Hz, 2H, ArCH), 7.76 (ddd, *J* = 8.5, 7.4, 1.2 Hz, 2H, ArCH), 6.79 (t, *J* = 2.1 Hz, 1H, ArCH), 6.77 (d, *J* = 2.2 Hz, 2H, ArCH), 3.72 (s, 6H, OCH$_3$); **^{13}C NMR** (126 MHz, DMSO-d$_6$) δ 161.5 (ArC), 139.2 (ArC), 133.9 (ArCH), 132.5 (ArC), 131.3 (ArC), 131.2 (ArCH), 128.6, (ArCH), 124.5 (ArCH), 107.1 (ArCH), 104.5 (ArCH), 56.1 (OCH$_3$). **HRMS** (ESI) $C_{20}H_{17}O_2S$ [M]$^+$: calculated 321.0944, found 321.0940; ***v*max** (thin film/cm^{-1}) 2922, 1600, 1573, 1466, 1425, 1292, 1163, 763, 670, 523, 422.

5-(1-Methyl-1H-pyrazol-4-yl)-5H-dibenzo[b,d]thiophen-5-ium chloride 2aa-DBT-Cl

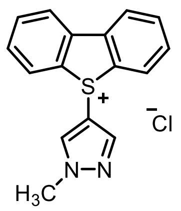

2aa-DBT-Cl

Following GP1, with a magnesiation time of 5 h at 37 ˚C, 4-bromo-2-fluoropyridine (61 µL, 0.60 mmol, 1.2 equiv.), 1.3 M isopropylmagnesium(II) chloride lithium chloride in THF (0.5 mL, 0.74 mmol, 1.4 equiv.) and 5-methoxy-5*H*-dibenzo[*b,d*]thiophen-5-ium tetrafluoroborate **1-DBT** (150 mg, 0.50 mmol, 1.0 equiv.) gave sulfonium salt **2aa-DBT-Cl** (98 mg, 0.33 mmol, 65%) as a cream solid; ^{1}H NMR (400 MHz, DMSO-d$_6$) δ 8.50 (dd, *J* = 7.7, 1.2 Hz, 2H, ArCH), 8.43 – 8.37 (m, 3H, ArCH), 7.93 (td, *J* = 7.6, 1.1 Hz, 2H, ArCH), 7.77 – 7.71 (m, 3H, ArCH), 3.85 (s, 3H, CH$_3$); ^{13}C NMR (101 MHz, DMSO-d$_6$) δ 140.8 (ArC), 138.8 (ArCH), 136.3 (ArC), 135.2 (ArCH), 133.9 (ArCH), 131.5 (ArCH), 128.2 (ArCH), 124.7 (ArCH), 102.9 (ArC), 40.0 (CH$_3$); **HRMS** (ESI) $C_{16}H_{13}N_2S$ [M]$^+$: calculated 265.0794, found 265.0781; ***v*max** (thin film/cm^{-1}) 3338, 1640, 1525, 1446, 1333, 1239, 1112, 952, 756, 528, 435.

5-(Thiophen-3-yl)-5H-dibenzo[b,d]thiophen-5-ium chloride 2ab-DBT-Cl

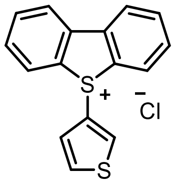

2ab-DBT-Cl

Following GP1, with a magnesiation time of 18 h at 37 °C, 3-bromothiophene (0.11 mL, 1.20 mmol, 1.2 equiv.), 1.3 M isopropylmagnesium(II) chloride lithium chloride in THF (1.1 mL, 1.40 mmol, 1.4 equiv.) and 5-methoxy-5*H*-dibenzo[*b,d*]thiophen-5-ium tetrafluoroborate **1-DBT** (302 mg, 1.00 mmol, 1.0 equiv.) gave sulfonium salt **2ab-DBT-Cl** (201 mg, 0.66 mmol, 66%) as a cream solid; **^{1}H NMR** (400 MHz, DMSO-d$_6$) δ 8.71 (dd, *J* = 3.1, 1.4 Hz, 1H, ArCH), 8.52 (d, *J* = 7.7 Hz, 2H, ArCH), 8.43 (d, *J* = 8.0 Hz, 2H, ArCH), 7.98–7.91 (m, 2H, ArCH), 7.79–7.72 (m, 3H, 2 x ArCH), 6.80 (dd, *J* = 5.4, 1.4 Hz, 1H, ArCH); **^{13}C NMR** (101 MHz, DMSO) δ 138.8 (ArC), 137.0 (ArCH), 133.7 (ArCH, ArC), 131.3 (ArCH), 131.1 (ArCH), 128.1 (ArCH), 125.0 (ArCH), 124.4 (ArCH), 121.6 (ArC); **HRMS** (ESI) $C_{16}H_{11}S_2$ $[M]^+$: calculated 267.0297, found 267.0285; ***v*$_{max}$** (thin film/cm^{-1}) 2920, 1448, 1236, 993, 854, 758, 520, 485, 420.

5-(Benzo[b]thiophen-4-yl)-5*H*-dibenzo[*b,d*]thiophen-5-ium chloride 2ac-DBT-Cl

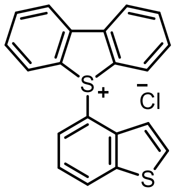

2ac-DBT-Cl

Following GP1, with a magnesiation time of 18 h at 37 °C, 4-bromobenzo[*b*]thiophene (127.8 mg, 0.60 mmol, 1.2 equiv.), 1.3 M isopropylmagnesium(II) chloride lithium chloride in THF (0.54 mL, 0.70 mmol, 1.4 equiv.) and 5-methoxy-5*H*-dibenzo[*b,d*]thiophen-5-ium tetrafluoroborate **1-DBT** (151 mg, 0.50 mmol, 1.0 equiv.) gave sulfonium salt **2ac-DBT-Cl** (135 mg, 0.34 mmol, 68%) as a cream solid; **^{1}H NMR** (500 MHz, DMSO-d$_6$) δ 8.60 (dd, *J* = 7.9, 1.2

Hz, 2H, ArCH), 8.47 (dt, *J* = 8.1, 0.9 Hz, 1H, ArCH), 8.33–8.28 (m, 2H, ArCH), 8.24 (d, *J* = 5.6 Hz, 1H, ArCH), 7.99 (td, *J* = 7.7, 1.1 Hz, 2H, ArCH), 7.74 (ddd, *J* = 8.4, 7.5, 1.2 Hz, 2H, ArCH), 7.61 (brs, 1H, ArCH), 7.51 (t, *J* = 7.9 Hz, 1H, ArCH), 7.33 (brs, 1H, ArCH); **^{13}C NMR** (101 MHz, DMSO-d$_6$) δ 142.1 (ArC), 139.2 (ArC), 137.9 (ArC), 134.1 (ArCH), 133.8 (ArCH), 131.9 (ArCH), 131.3 (ArCH), 129.2 (ArC), 128.3 (ArCH), 125.5 (ArCH), 124.6 (ArCH), 120.9 (ArC), 119.6 (ArCH); **HRMS** (ESI) $C_{20}H_{13}S_2$ [M]$^+$: calculated 317.0453, found 317.0449; ***v*max** (thin film/cm^{-1}) 2986, 1612, 1410, 1218, 864, 763, 571, 423.

10-(Benzo[*b*]thiophen-4-yl)-10*H*-phenoxathiin-10-ium chloride 2ac-PXT-Cl

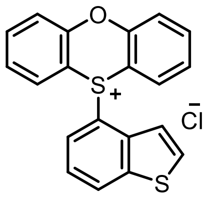

2ac-PXT-Cl

Following GP1, with a magnesiation time of 5 h at 37 °C, 4-bromobenzo[*b*]thiophene (0.200 g , 0.94 mmol, 1.2 equiv.), 1.3 M isopropylmagnesium(II) chloride lithium chloride in THF (0.8 mL, 1.10 mmol, 1.4 equiv.), and 10-methoxy-10*H*-phenoxathiin-10-ium tetrafluoroborate **1-PXT** (250 mg, 0.78 mmol, 1.0 equiv.) gave sulfonium salt **2ac-PXT-Cl** (185 mg, 0.50 mmol, 64%) as a beige solid; **^{1}H NMR** (500 MHz, DMSO-d$_6$) δ 8.45 (d, *J* = 8.0 Hz, 1H, ArCH), 8.36 – 8.29 (m, 3H, ArCH), 8.22 (d, *J* = 5.6 Hz, 1H, ArCH), 7.92 (ddd, *J* = 8.6, 7.3, 1.6 Hz, 2H, ArCH), 7.79 (dd, *J* = 8.5, 1.2 Hz, 2H, ArCH), 7.56 (td, *J* = 7.3, 2.5 Hz, 3H, ArCH), 7.35 (d, *J* = 7.8 Hz, 1H, ArCH); **^{13}C NMR** (101 MHz, DMSO-d$_6$) δ 151.2 (ArC), 142.6 (ArC), 137.0 (ArCH), 136.8 (ArC), 134.6 (ArCH), 132.1 (ArCH), 129.5 (ArCH), 127.4 (ArCH), 126.6 (ArCH), 126.0 (ArCH), 125.0 (ArC), 120.5 (ArCH), 106.4 (ArC); **HRMS** (ESI) $C_{20}H_{13}OS_2$ [M]$^+$: calculated 333.0402, found 333.0396; ***v*max** (thin film/cm^{-1}) 3345, 1581, 1465, 1435, 1310, 1272, 1213, 1063, 882, 769,554.

5-(*p*-Tolyl)-5*H*-dibenzo[*b,d*]thiophen-5-ium chloride 2ad-DBT-Cl

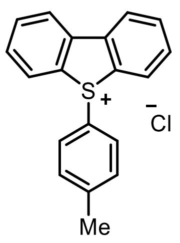

2ad-DBT-Cl

Following GP1, with a magnesiation time of 18 h at 37 °C, 4-bromotoluene (74 µL, 0.60 mmol, 1.2 equiv.), 1.3 M isopropylmagnesium(II) chloride lithium chloride in THF (0.54 mL, 0.70 mmol, 1.4 equiv.) and 5-methoxy-5*H*-dibenzo[*b,d*]thiophen-5-ium tetrafluoroborate **1-DBT** (151 mg, 0.50 mmol, 1.0 equiv.) gave sulfonium salt **2ad-DBT-Cl** (76 mg, 0.24 mmol, 49%) as a cream solid; **^{1}H NMR** (400 MHz, DMSO-d$_6$) δ 8.52 (d, *J* = 7.9 Hz, 2H, ArCH), 8.37 (d, *J* = 8.1 Hz, 2H, ArCH), 7.95 (td, *J* = 7.6, 1.1 Hz, 2H, ArCH), 7.74 (ddd, *J* = 8.4, 7.5, 1.2 Hz, 2H, ArCH), 7.55–7.45 (m, 2H, ArCH), 7.39 (d, *J* = 8.4 Hz, 2H, ArCH), 2.33 (s, 3H, CH$_3$); **^{13}C NMR** (101 MHz, DMSO-d$_6$) δ 145.0 (ArC), 139.1 (ArC), 133.8 (ArC), 133.5 (ArCH), 131.7 (ArCH), 131.2 (ArCH), 129.8 (ArCH), 128.3 (ArCH), 125.3 (ArC), 124.4 (ArCH), 20.9 (CH$_3$); **HRMS** (ESI) $C_{19}H_{15}S$ [M]$^+$: calculated 275.0894, found 275.0904; ***v*max** (thin film/cm^{-1}) 2922, 1588, 1446, 1185, 1063, 759, 707, 525, 421.

5-(4-Cyclopropylphenyl)-5*H*-dibenzo[*b,d*]thiophen-5-ium chloride 2ae-DBT-Cl

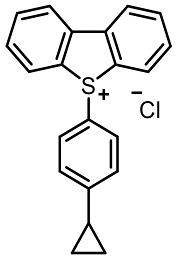

2ae-DBT-Cl

Following GP1, with a magnesiation time of 18 h at 37 °C, 1-bromo-4-cyclopropylbenzene (80 µL, 1.2 mmol, 1.2 equiv.), 1.3 M isopropylmagnesium(II) chloride lithium chloride in THF (0.54

mL, 1.4 mmol, 1.4 equiv.) and 5-methoxy-5*H*-dibenzo[*b,d*]thiophen-5-ium tetrafluoroborate **1-DBT** (151 mg, 0.50 mmol, 1.0 equiv.) gave sulfonium salt **2ae-DBT-Cl** (79 mg, 0.47 mmol, 47%) as a cream solid; **^{1}H NMR** (500 MHz, DMSO-d$_6$) δ 8.53 (d, *J* = 7.9 Hz, 2H, ArCH), 8.37 (d, *J* = 8.1 Hz, 2H, ArCH), 7.94 (td, *J* = 7.6, 1.1 Hz, 2H, ArCH), 7.74 (ddd, *J* = 8.4, 7.5, 1.2 Hz, 2H, ArCH), 7.45 (d, *J* = 8.6 Hz, 2H, ArCH), 7.26 (d, *J* = 8.7 Hz, 2H, ArCH), 1.96 (tt, *J* = 8.4, 5.0 Hz, 1H, CH), 1.02 (m, 2H, CH$_2$) 0.79 – 0.59 (m, 2H, CH$_2$); **^{13}C NMR** (126 MHz, DMSO-d$_6$) δ 151.6 (ArC), 139.0 (ArC), 133.8 (ArCH), 133.6 (ArC), 131.2 (ArCH), 129.9 (ArCH), 128.3 (ArCH), 127.8 (ArCH), 124.4 (ArCH), 124.3 (ArC), 15.2 (CH), 11.0 (CH$_2$); **HRMS** (ESI) $C_{21}H_{17}S$ $[M]^+$: calculated 301.1045, found 301.1041; ***v***$_{max}$ (thin film/cm^{-1}) 2993, 1587, 1447, 1221, 1041, 894, 767, 552, 420.

9. Proposed Mechanism for the Conversion of Methoxysulfonium salts 1 into (Hetero)Aryl Sulfonium Salts 2

Anderson, Acheson and Stubbs have proposed that the conversion of diarylalkoxy sulfonium salts to triaryl sulfonium salts by the addition of Grignard reagents proceeds by reaction at sulfur.[5,6] This likely proceeds by sulfurane formation followed by loss of the methoxy substituent, or alternatively, by direct displacement of the methoxy substituent, with both pathways promoted by Mg(II) salts.

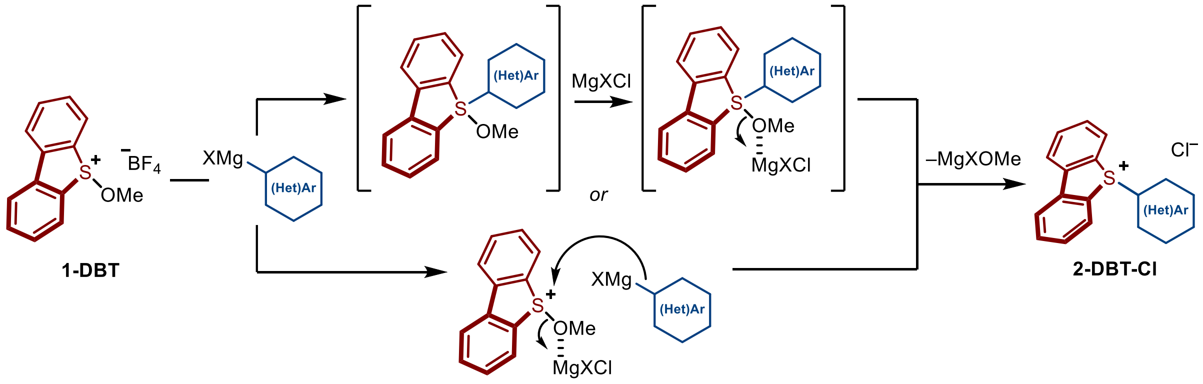

10.Cyclic Voltametry Studies

Cyclic voltammetry was conducted on an MultiTrace (PalmSens) potentiostat using a 3-electrode cell configuration. A glassy carbon working electrode was employed alongside a platinum wire counter electrode and a Ag/AgCl reference electrode. All the solutions were degassed by bubbling N_2 prior to measurements. 5 mM solutions of the desired compounds were freshly prepared in dry solvent along with 0.1 M of tetrabutylammonium hexafluorophosphate as supporting electrolyte and were examined at a scan rate of 0.1 V s^{-1}. Ferrocene (E1/2 = +0.42 V vs SCE) was added at the end of the measurements as an internal standard to determine the precise potential scale. Potential values are given versus the saturated calomel electrode (SCE). Irreversible reduction waves were obtained in all cases; therefore, the potentials were obtained from the maximum current, $E_p$max.

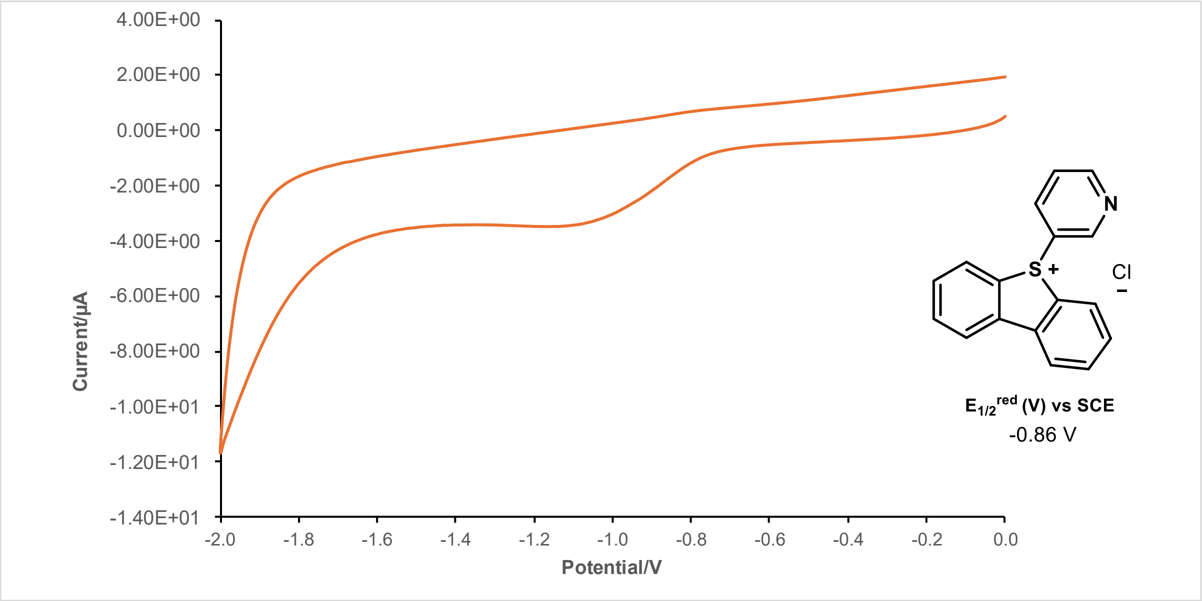

Figure 1: Cyclic voltammetry study of **2a-DBT-Cl** (5.0 mM) in DMSO.

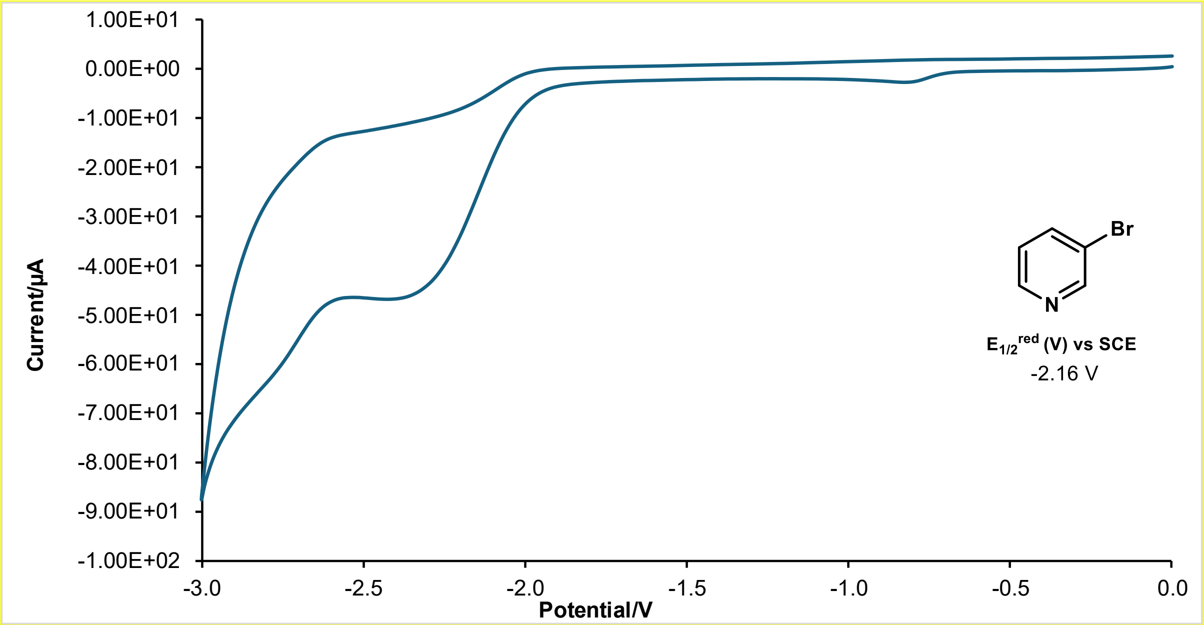

Figure 1: Cyclic voltammetry study of 3-bromopyridine (5.0 mM) in DMSO.

11. Control experiments with a (hetero)aryl bromide and a (hetero)aryl sulfonium salt

Control experiment 1 – Attempted formation of 3a from 3-bromopyridine

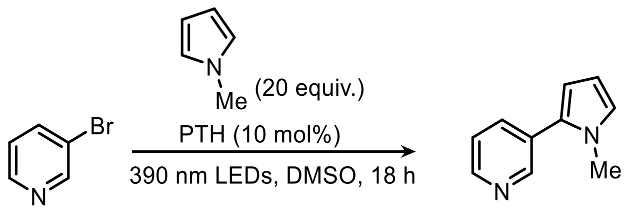

3a

10-Phenyl-phenothiazine (5.5 mg, 0.02 mmol, 10 mol%) was added to a reaction vial which was sealed with a crimp camp then evacuated and backfilled with nitrogen three times. DMSO (0.5 mL) was added followed by *N*-methyl pyrrole (0.36 mL, 4.00 mmol, 20.0 equiv.) and 3-bromopyridine (19 μL, 0.20 mmol, 1.0 equiv.). The crimp cap was then sealed with parafilm. The reaction mixture was irradiated with a Kessil PR 160 LED lamp (λ centred at 390 nm, 100% irradiance) for 18 h before quenching with saturated aqueous $NaHCO_3$ (10 mL) and diluting with EtOAc (10 mL). The aqueous layer was extracted with EtOAc (10 mL), then the combined organic layers were washed with brine (10 mL), dried using $MgSO_4$, filtered, and concentrated *in vacuo*. Purification by column chromatography using hexanes to 7:3 hexanes:EtOAc as eluent gave **3a** (5.2 mg, 0.033 mmol, 17%).

Control experiment 2 – Attempted formation of 5a from 3-bromopyridine

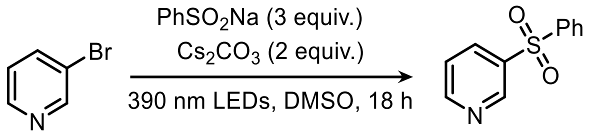

5a

Cs_2CO_3 (130 mg, 0.40 mmol, 2.0 equiv.), and sodium benzenesulfinate (99 mg, 0.60 mmol, 3.0 equiv.) were added to an oven-dried reaction vial equipped with a magnetic stirring bar and the vial was sealed with a crimp cap. Then, the vial was evacuated and backfilled with nitrogen three times. Next, dry DMSO (0.13 M) was added followed by 3-bromopyridine (19 μL, 0.20 mmol, 1.0 equiv.), and the crimp cap was sealed with parafilm. After irradiating with a Kessil PR 160 LED lamp (λ centred at 390 nm, 100% irradiance) at 2 cm away for 18 h under a cooling fan, brine (10 mL) and CH_2Cl_2 (10 mL) were added, and the layers separated. The aqueous layer was extracted with CH_2Cl_2 (2 x 10 mL), the combined organic layers were dried with Na_2SO_4 and concentrated *in vacuo*. Purification by column chromatography using hexanes to 7:3 hexanes:EtOAc as eluent gave **5a** (17 mg, 0.077 mmol, 39%).

Control experiment 3 – Attempted formation of 5b from 2b-DBT-Cl without light

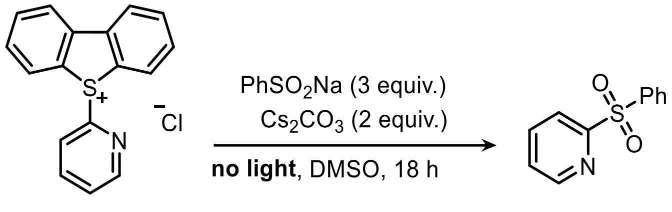

2b-DBT-Cl **5b**

2b-DBT-Cl (60 mg, 0.20 mmol, 1.0 equiv.), Cs_2CO_3 (130 mg, 0.40 mmol, 2.0 equiv.), and sodium benzenesulfinate (99 mg, 0.60 mmol, 3.0 equiv.) were added to an oven-dried reaction vial equipped with a magnetic stirring bar and the vial was sealed with a crimp cap. Then, the vial was evacuated and backfilled with nitrogen three times. Next, dry DMSO (0.13 M) was added, and the crimp cap was sealed with parafilm. The reaction was stirred for 18 h without light source (in dark). Brine (10 mL) and CH_2Cl_2 (10 mL) were added, and the layers separated.

The aqueous layer was extracted with CH_2Cl_2 (2 x 10 mL), the combined organic layers were dried with Na_2SO_4 and concentrated *in vacuo*. Purification by column chromatography using hexanes to 7:3 hexane:EtOAc as eluent gave **5b** (16 mg, 0.075 mmol, 38%).

12. Application of the new (hetero)aryl sulfonium salts

12.1 Scope for Arylation

3-(1-Methyl-1*H*-pyrrol-2-yl)pyridine 3a

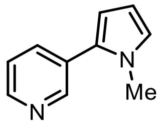

3a

Following GP2, aryl sulfonium salt **2a-DBT-Cl** (60 mg, 0.20 mmol, 1.0 equiv.), 10-phenyl-phenothiazine (5.5 mg, 0.02 mmol, 10 mol%) and *N*-methyl pyrrole (0.36 mL, 4.00 mmol, 20 equiv.) gave the crude product, which was purified by column chromatography on silica gel with hexanes to 7:3 hexanes:EtOAc as eluent to give 3-(1-methyl-1*H*-pyrrol-2-yl)pyridine **3a** (26 mg, 0.17 mmol, 83%) as a yellow oil; **^{1}H NMR** (400 MHz, $CDCl_3$) δ 8.69 (d, *J* = 2.0 Hz, 1H, ArCH), 8.53 (dd, *J* = 5.0, 1.5 Hz, 1H, ArCH), 7.70 (dt, *J* = 8.0, 2.0 Hz, 1H, ArCH), 7.32 (ddd, *J* = 8.0, 5.0, 1.0 Hz, 1H, ArCH), 6.77 (dd, *J* = 2.5, 2.0 Hz, 1H, ArCH), 6.30 (dd, *J* = 3.5, 2.0 Hz, 1H, ArCH), 6.23 (dd, *J* = 4.0, 3.0 Hz, 1H, ArCH), 3.68 (s, 3H, NCH_3); **^{13}C NMR** (101 MHz, $CDCl_3$) δ 149.4 (ArCH), 147.9 (ArCH), 135.6 (ArCH), 131.0 (ArC), 129.4 (ArC), 124.9 (ArCH), 123.4 (ArCH), 109.9 (ArCH), 108.4 (ArCH), 35.2 (NCH_3); **HRMS** (ESI) $C_{10}H_{11}N_2$ $[M+H]^+$ calculated 159.0924, found 159.0924; $\boldsymbol{\nu_{max}}$ (thin film/cm^{-1}) 2924, 1608, 1468, 1445, 1198, 1050, 814, 767, 735, 662. Data consistent with the literature[7]

2-(1-Methyl-1*H*-pyrrol-2-yl)pyridine 3b

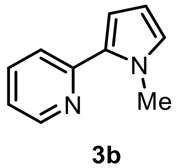

3b

Following GP2, aryl sulfonium salt **2b-DBT-Cl** (60 mg, 0.20 mmol, 1.0 equiv.), 10-phenyl-phenothiazine (5.5 mg, 0.02 mmol, 10 mol%) and *N*-methyl pyrrole (0.36 mL, 4.00 mmol, 20

equiv.) gave the crude product, which was purified by column chromatography on silica gel using hexanes to 7:3 hexanes:$CHCl_3$ as eluent to give 2-(1-methyl-1*H*-pyrrol-2-yl)pyridine **3b** (22 mg, 0.14 mmol, 70%) as a yellow oil; **^{1}H NMR** (400 MHz, $CDCl_3$) δ 8.56 (d, *J* = 5.7 Hz, 1H, ArCH), 7.63 (td, *J* = 7.8, 1.9 Hz, 1H, ArCH), 7.52 (dt, *J* = 8.1, 1.1 Hz, 1H, ArCH), 7.07 (ddd, *J* = 7.5, 4.9, 1.2 Hz, 1H, ArCH), 6.73 (t, *J* = 2.2 Hz, 1H, ArCH), 6.56 (dd, *J* = 3.8, 1.8 Hz, 1H, ArCH), 6.18 (dd, *J* = 3.8, 2.6 Hz, 1H, ArCH), 4.00 (s, 3H, NCH_3); **^{13}C NMR** (101 MHz, $CDCl_3$) δ 152.8 (ArC), 148.7 (ArCH), 136.3 (ArCH), 132.5 (ArC), 126.5 (ArCH), 121.6 (ArCH), 120.4 (ArCH), 110.8 (ArCH), 107.7 (ArCH), 37.0 (NCH_3); **HRMS** (ESI) $C_{10}H_{11}N_2$ $(M+H)^+$: calculated 159.0924, found 159.0925; ***v_{max}*** (thin film/cm^{-1}) 2926, 1588, 1490, 1437, 1318, 1152, 720, 607. Data consistent with the literature[7]

2-Fluoro-3-(1-methyl-1*H*-pyrrol-2-yl)benzonitrile 3c

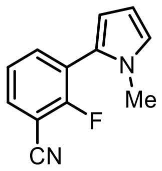

3c

Following GP2, aryl sulfonium salt **2n-DBT-Cl** (68 mg, 0.20 mmol, 1.0 equiv.), 10-phenyl-phenothiazine (5.5 mg, 0.02 mmol, 10 mol%) and *N*-methyl pyrrole (0.36 mL, 4.00 mmol, 20 equiv.) gave the crude product, which was purified by column chromatography on silica gel using hexanes to 6:4 hexanes:$CHCl_3$ as eluent to give 2-fluoro-3-(1-methyl-1*H*-pyrrol-2-yl)benzonitrile **3c** (19 mg, 0.094 mmol, 47%) as a yellow oil. **^{1}H NMR** (500 MHz, $CDCl_3$) δ 7.64–7.57 (m, 2H, 2 x ArCH), 7.30 (t, *J* = 7.7 Hz, 1H, ArCH), 6.81 (t, *J* = 2.3 Hz, 1H, ArCH), 6.31–6.22 (m, 2H, 2 x ArCH), 3.59 (d, *J* = 1.8 Hz, 3H, NCH_3); **^{13}C NMR** (126 MHz, $CDCl_3$) δ 160.3 (d, $J_{C–F}$ = 258.9 Hz, ArC), 136.9 (d, $J_{C–F}$ = 3.9 Hz, ArCH), 132.4 (ArCH), 125.9 (ArC), 125.0 (ArCH), 124.9 (ArCH), 122.9 (d, $J_{C–F}$ = 13.9 Hz, ArC), 114.1 (CN), 111.5 (ArCH), 108.6 (ArCH), 102.2 (d, $J_{C–F}$ = 16.7 Hz, ArC), 35.0 (d, $J_{C–F}$ = 4.9 Hz, NCH_3); **^{19}F NMR** (376 MHz, $CDCl_3$) δ -107.10; **HRMS** (ESI) $C_{12}H_{10}N_2F$ $[M+H]^+$: calculated 201.0823, found 201.0821; ***v_{max}*** (thin film/cm^{-1}) 2920, 2235, 1542, 1469, 1315, 1093, 720, 607.

1-Methyl-2-(4-(trifluoromethyl)phenyl)-1*H*-pyrrole 3d

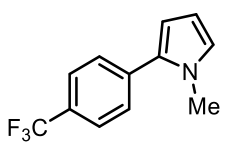

3d

Following GP2, aryl sulfonium salt **2f-DBT-Cl** (73 mg, 0.20 mmol, 1.0 equiv.), 10-phenyl-phenothiazine (5.5 mg, 0.2 mmol, 10 mol%) and *N*-methyl pyrrole (0.36 mL, 4.00 mmol, 20 equiv.) gave the crude product, which was purified by column chromatography on silica gel using hexanes to 9:1 hexanes:$CHCl_3$ as eluent to give 1-methyl-2-(4-(trifluoromethyl)phenyl)-1*H*-pyrrole **3d** (29 mg, 0.13 mmol, 65%) as an yellow amorphous solid; **^{1}H NMR** (500 MHz, $CDCl_3$) δ 7.65 (d, *J* = 8.0 Hz, 2H, ArCH), 7.52 (d, *J* = 8.0 Hz, 2H, ArCH), 6.77 (brs, 1H, ArCH), 6.32 (brs, 1H, ArCH), 6.23 (brs, 1H, ArCH), 3.71 (s, 3H, CH_3), **^{13}C NMR** (126 MHz, $CDCl_3$) δ 136.9 (ArC), 133.2 (ArC), 128.6 (q, $J_{C–F}$ = 32.1 Hz, ArC), 128.5 (ArCH), 125.5 (q, $J_{C–F}$ = 3.8 Hz, ArCH), 125.1 (ArCH), 124.9 (q, $J_{C–F}$ = 271.9 Hz, CF_3), 110.1 (ArCH), 108.4 (ArCH), 35.4 (NCH_3); **^{19}F NMR** (471 MHz, $CDCl_3$) δ -62.41; **HRMS** (ESI) $C_{12}H_{11}NF_3$ $[M+H]^+$: calculated 226.0838, found 226.0838; ***v*max** (thin film/cm^{-1}) 2928, 1612, 1477, 1426, 1323, 1164, 1070, 1068, 845, 725, 476. Data consistent with the literature[8]

1-Methyl-2-(2-(methylthio)phenyl)-1*H*-pyrrole 3e

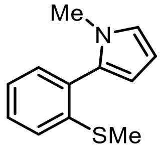

3e

Following GP2, aryl sulfonium salt **2s-DBT-Cl** (69 mg, 0.20 mmol, 1.0 equiv.), 10-phenyl-phenothiazine (5.5 mg, 0.02 mmol, 10 mol%) and *N*-methyl pyrrole (0.36 mL, 4.00 mmol, 20 equiv.) gave the crude product, which was purified by column chromatography on silica gel using hexanes to 8:2 hexanes:$CHCl_3$ as eluent to give 1-methyl-2-(2-(methylthio)phenyl)-1*H*-

pyrrole **3e** (29 mg, 0.13 mmol, 65%) as an off-white amorphous solid; **^{1}H NMR** (500 MHz, $CDCl_3$) δ 7.43–7.36 (m, 1H, ArCH), 7.30–7.24 (m, 2H, 2 x ArCH,), 7.20 (t, *J* = 7.5 Hz, 1H, ArCH), 6.77 (brs, 1H, ArCH), 6.30–6.25 (m, 1H, ArCH), 6.20 (brs, 1H, ArCH), 3.48 (s, 3H, NCH_3), 2.42 (s, 3H, SCH_3); **^{13}C NMR** (126 MHz, $CDCl_3$) δ 140.9 (ArC), 131.9 (2 x ArC), 131.6 (ArCH), 128.8 (ArCH), 124.3 (ArCH), 124.2 (ArCH), 122.3 (ArCH), 109.3 (ArCH), 107.5 (ArCH), 34.3 (NCH_3), 15.3 (SCH_3); **HRMS** (ESI) $C_{12}H_{14}NS$ $[M+H]^+$: calculated 204.0847, found 204.0847; ν_{max} (thin film/cm^{-1}) 2920, 1589, 1486, 1309, 1086, 756, 712, 608.

2-(3,5-Dimethoxyphenyl)-1-methyl-1*H*-pyrrole 3f

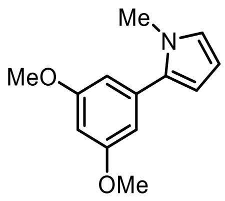

3f

Following GP2, aryl sulfonium salt **2z-DBT-Cl** (71 mg, 0.20 mmol, 1.0 equiv.), 10-phenyl-phenothiazine (5.5 mg, 0.02 mmol, 10 mol%) and *N*-methyl pyrrole (0.36 mL, 4.00 mmol, 20 equiv.) gave the crude product, which was purified by column chromatography on silica gel using hexanes to 6:4 hexanes:$CHCl_3$ as eluent to give 1-methyl-2-(2-(methylthio)phenyl)-1*H*-pyrrole **3f** (29 mg, 0.13 mmol, 65%) as a colourless oil; **^{1}H NMR** (500 MHz, CDCl3) δ 6.62 (t, *J* = 2.3 Hz, 1H, ArCH), 6.47 (d, *J* = 2.3 Hz, 2H, ArCH), 6.34 (t, *J* = 2.3 Hz, 1H, ArCH), 6.15 (dd, *J* = 3.6, 1.8 Hz, 1H, ArCH), 6.13–6.09 (m, 1H, ArCH), 3.73 (s, 6H, OCH_3), 3.59 (s, 3H, NCH_3); **^{13}C NMR** (126 MHz, $CDCl_3$) δ 160.7 (ArC), 135.3 (ArC), 134.6 (ArC), 123.9 (ArCH), 108.8 (ArCH), 107.8 (ArCH), 107.0 (ArCH), 99.0 (ArCH), 55.5 (OCH_3), 35.2 (NCH_3); **HRMS** (ESI) $C_{13}H_{15}NO_2$ $[M+H]^+$: calculated 218.1179, found 218.1178; ν_{max} (thin film/cm^{-1}) 2935, 1591, 1456, 1279, 1204, 1154, 844, 715, 609. Data consistent with the literature[9]

1-(4-Fluorophenyl)vinyl)oxy)trimethylsilane S5

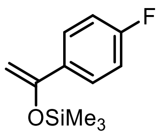

S5

An oven dried round bottom flask containing a mixture of 1-(4-fluorophenyl)ethan1-one (2.4 mL, 20.0 mmol, 1.0 equiv.) and dry NaI (3.6 g, 24.0 mmol, 1.2 equiv.) was evacuated and back-filled with N_2. Then, MeCN (40 ml) was added and the solution stirred for 5 min at RT. To the resulting solution, anhydrous NEt_3 (4.2 mL, 30.0 mmol, 1.5 equiv.) was added, followed by TMSCl (3.1 mL, 24.0 mmol, 1.2 equiv.). The reaction mixture was stirred for 16 h at room temperature. The reaction was quenched with a cold saturated solution of ammonium chloride (25 mL) and extracted with pentane (3 × 50 mL). The residue was concentrated *in vacuo* to give ((1-(4-fluorophenyl)vinyl)oxy)trimethylsilane **S5** (3.87 g, 18.4 mmol, 92%) as a colourless oil, which was used without further purification; **^{1}H NMR** (500 MHz, $CDCl_3$) δ 7.59–7.52 (m, 2H, ArH), 7.05–6.96 (m, 2H, ArH), 4.83 (d, J = 1.9 Hz, 1H, CH), 4.40 (d, J = 1.9 Hz, 1H, CH), 0.27 (s, 9H, CH_3); **^{13}C NMR** (126 MHz, $CDCl_3$) δ 163.0 (d, $J_{C–F}$ = 247.2 Hz, ArC), 154.9 (=C–O), 133.8 (d, $J_{C–F}$ = 3.3 Hz, ArC), 127.1 (d, $J_{C–F}$ = 8.0 Hz, ArCH), 115.1 (d, J = 21.6 Hz, ArCH), 90.8 (d, $J_{C–F}$ = 1.7 Hz, =CH_2), 0.20 (CH_3); **^{19}F NMR** (471 MHz, $CDCl_3$) δ -114.05; **HRMS** (ESI) $C_{11}H_{16}OFS$ $[M+H]^+$: calculated 211.0949, found 211.0953. Data in accordance with the literature.[9]

12.2 Scope for Alkylation

1-(4-Fluorophenyl)-2-(pyridin-3-yl)ethan-1-one 4a

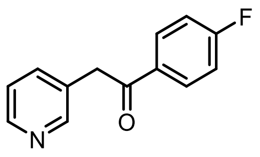

4a

Following GP3, aryl sulfonium salt **2a-DBT-Cl** (60 mg, 0.20 mmol, 1.0 equiv.), PTH (2.8 mg, 0.01 mmol, 5 mol%), 2,6-lutidine (35 μL, 0.30 mmol, 1.5 equiv.), silyl enol ether **S5** (210 mg, 1.00 mmol, 5.0 equiv.), and DMSO (1 mL) gave the crude product. The product was purified by column chromatography on silica gel using 90:10 hexane:acetone as eluent to give 1-(4-fluorophenyl)-2-(pyridin-3-yl)ethan-1-one **4a** (20 mg, 0.092 mmol, 46%) as a colorless oil. **^{1}H NMR** (400 MHz, $CDCl_3$) δ 8.54 – 8.49 (m, 2H, ArCH), 8.09 – 8.00 (m, 2H, ArCH), 7.59 (dt, J = 7.9, 2.0 Hz, 1H, ArCH), 7.30 – 7.26 (m, 1H, ArCH), 7.18 – 7.11 (m, 2H, ArCH), 4.27 (s, 2H, CH_2); **^{13}C NMR** (101 MHz, $CDCl_3$) δ 194.8 (C=O), 165.9 (d, $J_{C\text{-}F}$ = 255.8 Hz, ArC), 150.5 (ArCH), 148.3 (ArCH), 137.2 (ArCH), 132.6 (d, $J_{C\text{-}F}$ = 3.0 Hz, ArC), 131.1 (d, $J_{C\text{-}F}$ = 9.4 Hz, ArCH), 130.0 (ArC), 123.5 (ArCH), 115.9 (d, J = 22.0 Hz, ArCH), 42.2 (CH_2); **^{19}F NMR** (376 MHz, $CDCl_3$) δ -104.16; **HMRS** (APCI) $C_{13}H_{11}ONF$ $[M+H]^+$: calculated 216.0819, found 216.0823; ν_{max} (thin film/cm^{-1}) 2999, 1681, 1597, 1507, 1427, 1328, 1209, 1156, 993, 906, 725, 566. Data consistent with the literature[10]

1-(4-Fluorophenyl)-2-(1-methyl-1H-pyrazol-4-yl)ethan-1-one 4b

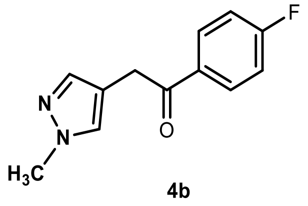

4b

Following GP3, aryl sulfonium salt **2aa-DBT-Cl** (60 mg, 0.20 mmol, 1.0 equiv.), PTH (2.8 mg, 0.01 mmol, 5 mol%), silyl enol ether **S5** (210 mg, 1.00 mmol, 5.0 equiv.), and DMSO (0.5 mL)

gave the crude product. The product was purified by column chromatography on silica gel using 80:20 hexane:acetone as eluent to give 1-(4-fluorophenyl)-2-(1-methyl-1H-pyrazol-4-yl)ethan-1-one **4b** (17 mg, 0.077 mmol, 38%) as a yellow solid; **^{1}H NMR** (400 MHz, $CDCl_3$) δ 8.08 – 8.02 (m, 2H, ArCH), 7.42 (s, 1H, ArCH), 7.36 (s, 1H, ArCH), 7.20 – 7.10 (m, 2H, ArCH), 4.14 (s, 2H, CH_2), 3.89 (s, 3H, CH_3); **^{13}C NMR** (101 MHz, $CDCl_3$) δ 195.6 (C=O), 165.8 (d, J_{C-F} = 255.2 Hz, ArCF), 139.3 (ArCH), 132.79 (d, J_{C-F} = 2.9 Hz, ArC), 131.16 (d, J_{C-F} = 9.3 Hz, ArCH), 129.65 (ArCH), 115.8 (d, J_{C-F} = 21.9 Hz, ArCH), 113.2 (ArC), 38.9 (CH_2), 34.5 (CH_3); **^{19}F NMR** (376 MHz, $CDCl_3$) δ -104.89; **HRMS** (ESI) $C_{12}H_{11}ON_2F$ $[M+H]^+$: calculated 218.0934, found 219.0933; $\boldsymbol{v_{max}}$ (thin film/cm^{-1}) 2873, 1685, 1595, 1507, 1447, 1358, 1217, 1206, 1158, 991, 833, 559.

1-(4-Fluorophenyl)-2-(4-(trifluoromethyl)phenyl)ethan-1-one 4c

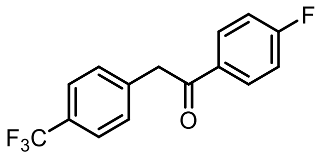

4c

Following GP3, aryl sulfonium salt **2f-DBT-Cl** (73 mg, 0.20 mmol, 1.0 equiv.), PTH (2.8 mg, 0.01 mmol, 5 mol%) and silyl enol ether **S5** (210 mg, 1.00 mmol, 5.0 equiv.) gave the crude product, which was purified by column chromatography on silica gel using 95:5 pentane:acetone as eluent to give 1-(4-fluorophenyl)-2-(4-(trifluoromethyl)phenyl)ethan-1-one **4c** (39 mg, 0.14 mmol, 69%) as a white solid; **^{1}H NMR** (400 MHz, $CDCl_3$) δ 8.04 (m, 2H, ArCH), 7.60 (d, J = 8.0 Hz, 2H, ArCH), 7.37 (d, J = 7.9 Hz, 2H, ArCH), 7.21–7.10 (m, 2H, ArCH), 4.33 (s, 2H, CH_2); **^{13}C NMR** (101 MHz, $CDCl_3$) δ 195.2 (C=O), 166.1 (d, J_{C-F} = 255.7 Hz, ArC), 138.4 (ArC), 132.9 (d, J_{C-F} = 3.1 Hz, ArC), 131.3 (d, J_{C-F} = 9.3 Hz, ArCH), 130.1 (ArCH), 129.5 (q, J_{C-F} = 32.5 Hz, ArC), 125.8 (q, J_{C-F} = 3.8 Hz, ArCH), 124. 7 (q, J_{C-F} = 271.9 Hz, CF_3), 116.1 (d, J_{C-F} = 21.9 Hz, ArCH), 45.2 (CH_2); **^{19}F NMR** (376 MHz, $CDCl_3$) δ -104.27, -62.54; **HRMS** (ESI) $C_{15}H_9OF_4$ $[M-H]^-$: calculated 281.0592, found 281.0592; $\boldsymbol{v_{max}}$ (thin film/cm^{-1}) 2922, 1689, 1597, 1506, 1474, 1324, 1204, 1155, 834, 541.

1-(4-Fluorophenyl)-2-(2-(methylthio)phenyl)ethan-1-one 4d

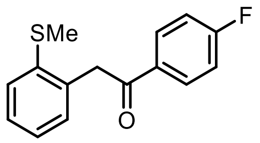

4d

Following GP3, aryl sulfonium salt **2s-DBT-Cl** (69 mg, 0.20 mmol, 1.0 equiv.), PTH (2.8 mg, 0.01 mmol, 5 mol%) and silyl enol ether **S5** (210 mg, 1.00 mmol, 5.0 equiv.) gave the crude product, which was purified by column chromatography on silica gel using 95:5 pentane:acetone as eluent to give 1-(4-fluorophenyl)-2-(2-(methylthio)phenyl)ethan-1-one **4d** (42 mg, 0.16 mmol, 81%) as a yellow waxy solid; **^{1}H NMR** (400 MHz, $CDCl_3$) δ 8.07 (dd, *J* = 7.4, 5.3 Hz, 2H, ArCH), 7.36–7.28 (m, 2H, 2 x ArCH), 7.20–7.09 (m, 4H, 3 x ArCH), 4.40 (s, 2H, CH_2), 2.44 (s, 3H, SCH_3); **^{13}C NMR** (101 MHz, $CDCl_3$) δ 196.2 (C=O), 166.2 (d, $J_{C–F}$ = 254.8 Hz, ArC), 138.1 (ArC), 134.2 (ArC), 133.6 (d, $J_{C–F}$ = 2.9 Hz, ArC), 131.51 (d, $J_{C–F}$ = 9.3 Hz, ArCH), 130.9 (ArCH), 128.4 (ArCH), 127.8 (ArCH), 126.1 (ArCH), 116.18 (d, $J_{C–F}$ = 21.9 Hz, ArCH), 43.9 (CH_2), 17.1 (SCH_3); **^{19}F NMR** (376 MHz, $CDCl_3$) δ -105.23; **HRMS** (APCI) $C_{15}H_{14}OFS$ $[M+H]^+$: calculated 261.0744, found 261.0743; ν_{max} (thin film/cm^{-1}) 2920, 1687, 1596, 1505, 1327, 1213, 1156, 744, 569.

2-(2,6-Dimethoxyphenyl)-1-(4-fluorophenyl)ethan-1-one 4e

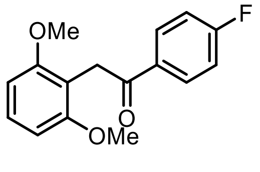

4e

Following GP5, aryl sulfonium salt **2w-DBT-Cl** (71 mg, 0.20 mmol, 1.0 equiv.), PTH (2.8 mg, 0.01 mmol, 5 mol%) and silyl enol ether **S5** (210 mg, 1.00 mmol, 5.0 equiv.) gave the crude product, which was purified by column chromatography on silica gel using 95:5 pentane:acetone as eluent to give 2-(2,6-dimethoxyphenyl)-1-(4-fluorophenyl)ethan-1-one **4e**

(45 mg, 0.16 mmol, 82%) as a white solid; **^{1}H NMR** (400 MHz, $CDCl_3$) δ 8.14–8.05 (m, 2H, ArCH), 7.31–7.22 (m, 1H, ArCH), 7.19–7.09 (m, 2H, ArCH), 6.60 (d, J = 8.3 Hz, 2H, ArCH), 4.32 (s, 2H, CH_2), 3.79 (d, J = 1.1 Hz, 6H, OCH_3); **^{13}C NMR** (101 MHz, $CDCl_3$) δ 196.6 (C=O), 165.5 (d, $J_{C\text{–}F}$ = 253.7 Hz, ArC), 158.3 (ArC), 133.8 (d, $J_{C\text{–}F}$ = 3.0 Hz, ArC), 130.8 (d, $J_{C\text{–}F}$ = 9.3 Hz ArCH), 128.3 (ArCH), 115.4 (d, $J_{C\text{–}F}$ = 21.9 Hz, ArCH), 112.0 (ArC), 103.8 (ArCH), 55.8 (OCH_3), 33.9 (CH_2); **^{19}F NMR** (376 MHz, $CDCl_3$) δ -106.74; **HMRS** (ESI) $C_{16}H_{15}FO_3Na$ $[M+Na]^+$: calculated 297.0911, found 297.0911; $\boldsymbol{v}_{\mathbf{max}}$ (thin film/cm^{-1}) 2937, 1688, 1594, 1473, 1329, 1103, 759, 540.

12.3 Scope for Sulfonylation

3-(Phenylsulfonyl)pyridine 5a

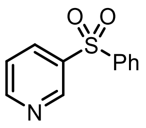

5b

Following GP4, aryl sulfonium salt **2a-DBT-Cl** (60 mg, 0.20 mmol, 1.0 equiv.), Cs_2CO_3 (65 mg, 0.40 mmol, 2.0 equiv.) and sodium phenylsulfinate (99 mg, 0.60 mmol, 3.0 equiv.) gave the crude product, which was purified by column chromatography on silica gel using pentane to 7:3 pentane:EtOAc as eluent to give 3-(phenylsulfonyl)pyridine **5a** (29 mg, 0.15 mmol, 74%) as a white solid. **^{1}H NMR** (500 MHz, $CDCl_3$) δ 9.14 (d, *J* = 2.3 Hz, 1H, ArCH), 8.77 (dd, *J* = 4.8, 1.7 Hz, 1H, ArCH), 8.24–8.18 (m, 1H, ArCH), 8.01–7.94 (m, 2H, ArCH), 7.65–7.57 (m, 1H, ArCH), 7.58–7.49 (m, 2H, ArCH), 7.44 (ddd, *J* = 8.0, 4.9, 0.9 Hz, 1H, ArCH); **^{13}C NMR** (126 MHz, $CDCl_3$) δ 153.8 (ArCH), 148.8 (ArCH), 140.9 (ArC), 138.4 (ArC), 135.4 (ArCH), 134.0 (ArCH), 129.7 (ArCH), 127.9 (ArCH), 124.0 (ArCH); **HRMS** (ESI) $C_{11}H_{10}O_2NS$ $[M+H]^+$: calculated 220.0427, found 220.0426; ν_{max} (thin film/cm^{-1}) 3062, 1572, 1446, 1415, 1310, 1167, 739, 591. Data consistent with the literature[11]

2-(Phenylsulfonyl)pyridine 5b

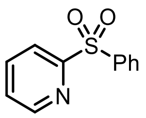

5b

Following GP3, aryl sulfonium salt **2b-DBT-Cl** (60 mg, 0.20 mmol, 1.0 equiv.), Cs_2CO_3 (65 mg, 0.40 mmol, 2.0 equiv.) and sodium phenylsulfinate (99 mg, 0.60 mmol, 3.0 equiv.) gave the crude product, which was purified by column chromatography on silica gel using pentane to 7:3 pentane:EtOAc as eluent to give 2-(phenylsulfonyl)pyridine **5b** (21 mg, 0.10 mmol, 48%) as a white solid. **^{1}H NMR** (400 MHz, $CDCl_3$) δ 8.70 – 8.65 (m, 1H, ArCH), 8.21 (d, *J* = 7.9 Hz,

1H, ArCH), 8.07 (dd, J = 7.1, 1.6 Hz, 2H, ArCH), 7.93 (td, J = 7.8, 1.7 Hz, 1H, ArCH), 7.66 – 7.59 (m, 1H, ArCH), 7.54 (t, J = 7.5 Hz, 2H, ArCH), 7.46 (ddd, J = 7.7, 4.7, 1.1 Hz, 1H, ArCH); **^{13}C NMR** (101 MHz, $CDCl_3$) δ 158.8 (ArC), 150.5 (ArCH), 138.9 (ArC), 138.1 (ArCH), 133.7 (ArCH), 129.1 (ArCH), 128.9 (ArCH), 126.9 (ArCH), 122.2 (ArCH); **HRMS** (ESI) $C_{11}H_{10}O_2NS$ $[M+H]^+$: calculated 220.0427, found 220.0422; $\boldsymbol{v}_{\mathbf{max}}$ (thin film/cm^{-1}) 2923, 1577, 1447, 1426, 1308, 1167, 1125, 743, 590, 565. Data consistent with the literature[11]

2-Fluoro-3-(phenylsulfonyl)benzonitrile 5c

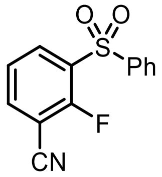

5c

Following GP3 3, aryl sulfonium salt **2n-DBT-Cl** (68 mg, 0.20 mmol, 1.0 equiv.), Cs_2CO_3 (65 mg, 0.40 mmol, 2.0 equiv.) and sodium phenylsulfinate (99 mg, 0.60 mmol, 3.0 equiv.) gave the crude product, which was purified by column chromatography on silica gel using pentane to 7:3 pentane:EtOAc as eluent to give 2-fluoro-3-(phenylsulfonyl)benzonitrile **5c** (24 mg, 0.092 mmol, 45%) as a white solid. **^{1}H NMR** (500 MHz, $CDCl_3$) δ 8.07 (d, J = 7.4 Hz, 2H, ArCH), 7.66–7.56 (m, 3H, 3 x ArCH), 7.52 (t, J = 7.8 Hz, 2H, ArCH), 7.32 (ddd, J = 9.7, 7.7, 1.9 Hz, 1H, ArCH); **^{13}C NMR** (126 MHz, $CDCl_3$) δ 160.0 (d, $J_{C\text{–}F}$ = 260.7 Hz, ArC), 140.1 (ArC), 135.6 (d, $J_{C\text{–}F}$ = 10.2 Hz, ArCH), 134.7 (ArCH), 132.2 (d, $J_{C\text{–}F}$ = 3.5 Hz, ArCH), 131.6 (d, $J_{C\text{–}F}$ = 15.7 Hz, ArC), 129.5 (ArCH), 128.3 (d, $J_{C\text{–}F}$ = 2.0 Hz, ArCH), 122.4 (d, $J_{C\text{–}F}$ = 23.2 Hz, ArCH), 115.1 (d, $J_{C\text{–}F}$ = 4.2 Hz, CN), 113.5 (d, $J_{C\text{–}F}$ = 1.9 Hz, ArC); **^{19}F NMR** (471 MHz, $CDCl_3$) δ -101.53; **HRMS** (ESI) $C_{13}H_8O_2NFNaS$ $[M+Na]^+$: calculated 284.0152, found 284.0148; $\boldsymbol{v}_{\mathbf{max}}$ (thin film/cm^{-1}) 3066, 2235, 1573, 1461, 1447, 1326, 1155, 1115, 960, 767, 567, 493.

1-(Phenylsulfonyl)-4-(trifluoromethyl)benzene 5d

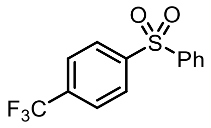

5d

Following GP3, aryl sulfonium salt **2f-DBT-Cl** (73 mg, 0.20 mmol, 1.0 equiv.), Cs_2CO_3 (65 mg, 0.40 mmol, 2.0 equiv.) and sodium phenylsulfinate (99 mg, 0.60 mmol, 3.0 equiv.) gave the crude product, which was purified by column chromatography on silica gel using pentane to 7:3 pentane:EtOAc as eluent to give 1-(phenylsulfonyl)-4-(trifluoromethyl)benzene **5d** (28 mg, 0.13 mmol, 65%) as a white solid. **^{1}H NMR** (400 MHz, $CDCl_3$) δ 8.07 (d, J = 8.1 Hz, 2H, ArCH), 8.00–7.92 (m, 2H, ArCH), 7.77 (d, J = 8.2 Hz, 2H, ArCH), 7.65–7.58 (m, 1H, ArCH), 7.58–7.48 (m, 2H, ArCH); **^{13}C NMR** (101 MHz, $CDCl_3$) δ 145.2 (ArC), 140.6 (ArC), 134.8 (q, $J_{C–F}$ = 33.1 Hz, ArC), 133.8 (ArCH), 129.5 (ArCH), 128.2 (ArCH), 127.9 (ArCH), 126.4 (q, $J_{C–F}$ = 3.7 Hz, ArCH), 123.1 (q, J = 273.0 Hz, CF_3); **^{19}F NMR** (376 MHz, $CDCl_3$) δ -63.21; **HRMS** (ESI) $C_{13}H_{10}O_2F_3S$ $[M+H]^+$: calculated 287.0348 found 287.0347; $\boldsymbol{\nu}_{\mathbf{max}}$ (thin film/cm^{-1}) 3075, 1582, 1477, 1446, 1317, 1143, 1058, 1017, 845, 721, 589, 558, 421. Data consistent with the literature[12]

Methyl(2-(phenylsulfonyl)phenyl)sulfane 5e

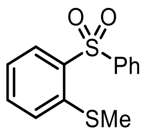

5e

Following GP3, aryl sulfonium salt **2s-DBT-Cl** (69 mg, 0.20 mmol, 1.0 equiv.), Cs_2CO_3 (65 mg, 0.40 mmol, 2.0 equiv.) and sodium phenylsulfinate (99 mg, 0.60 mmol, 3.0 equiv.) gave the crude product, which was purified by column chromatography on silica gel using pentane to 7:3 pentane:EtOAc as eluent to give methyl(2-(phenylsulfonyl)phenyl)sulfane **5e** (37 mg, 0.14

mmol, 69%) as an white solid. **^{1}H NMR** (500 MHz, $CDCl_3$) δ 8.18 (dd, *J* = 7.9, 1.5 Hz, 1H, ArCH), 7.94–7.90 (m, 2H, ArCH), 7.53–7.47 (m, 1H, ArCH), 7.47–7.37 (m, 3H, 2 x ArCH), 7.23 (td, *J* = 7.6, 1.1 Hz, 1H, ArCH), 7.21–7.14 (m, 1H, ArCH), 2.29 (s, 3H, SCH_3); **^{13}C NMR** (126 MHz, $CDCl_3$) δ 140.5 (ArC), 140.3 (ArC), 137.4 (ArC), 133.7 (ArCH), 133.3 (ArCH), 130.7 (ArCH), 128.8 (ArCH), 128.4 (ArCH), 126.6 (ArCH), 124.8 (ArCH), 16.1 (SCH_3); **HRMS** (ESI) $C_{13}H_{13}O_2S_2$ $[M+H]^+$: calculated 265.0351, found 265.0351; ν_{max} (thin film/cm^{-1}) 2917, 1447, 1306, 1154, 746, 687, 589, 567.

1,3-Dimethoxy-5-(phenylsulfonyl)benzene 5f

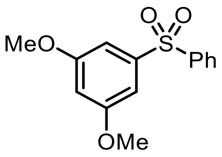

5f

Following GP3, aryl sulfonium salt **2z-DBT-Cl** (71 mg, 0.20 mmol, 1.0 equiv.), Cs_2CO_3 (65 mg, 0.40 mmol, 2.0 equiv.) and sodium phenylsulfinate (99 mg, 0.60 mmol, 3.0 equiv.) gave the crude product, which was purified by column chromatography on silica gel using pentane to 7:3 pentane:EtOAc as eluent to give 1,3-dimethoxy-5-(phenylsulfonyl)benzene **5f** (15 mg, 0.054 mmol, 27%) as a white solid; **^{1}H NMR** (400 MHz, $CDCl_3$) δ 7.94 (d, *J* = 7.6 Hz, 2H, ArCH), 7.61–7.46 (m, 3H, ArCH), 7.06 (d, *J* = 2.3 Hz, 2H, ArCH), 6.59 (d, *J* = 2.2 Hz, 1H, ArCH), 3.81 (s, 6H, OCH_3); **^{13}C NMR** (101 MHz, $CDCl_3$) δ 161.3 (ArC), 143.4 (ArC), 141.6 (ArC), 133.4 (ArCH), 129.4 (ArCH), 127.5 (ArCH), 105.6 (ArCH), 105.5 (ArCH), 55.9 (OCH_3); **HRMS** (ESI) $C_{14}H_{15}O_4S$ $[M+H]^+$: calculated 279.0686, found 279.0684; ν_{max} (thin film/cm^{-1}) 2922, 1600, 1459, 1425, 1342, 1205, 1150, 1037, 720, 581. Data consistent with the literature[13]

12.4 Scope for Ligand Coupling

2-(4-(Cyano)phenyl)pyridine 6a

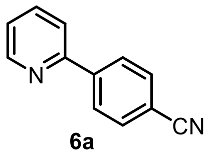

6a

Following GP5, 2-bromopyridine (61 µL, 0.63 mmol, 1 equiv.) at RT was added 1.3 M isopropylmagnesium(II) chloride lithium chloride in THF (0.58 mL, 0.76 mmol, 1.2 equiv.). The reaction was stirred for 3 hours at RT before use. In a separate flask, a solution of **2h-DBT-Cl** (65 mg, 0.2 mmol, 1.0 equiv.) in THF (2 mL) was cooled to -78 °C, and the preformed aryl Grignard reagent (0.4 mL, 0.4 mmol, 2.0 equiv.) was added. The product was purified by column chromatography on silica gel using 15:85 acetone:hexanes as eluent to give 2-(4-(cyano)phenyl)pyridine **6a** (18 mg, 0.099 mmol, 50%) as a white solid; **^{1}H NMR** (400 MHz, $CDCl_3$) δ 8.73 (dt, *J* = 4.9, 1.4 Hz, 1H, ArCH), 8.15 – 8.09 (m, 2H, ArCH), 7.82 (td, *J* = 7.6, 1.8 Hz, 1H, ArCH), 7.79 – 7.74 (m, 3H, ArCH), 7.32 (ddd, *J* = 7.3, 4.8, 1.4 Hz, 1H, ArCH); **^{13}C NMR** (126 MHz, $CDCl_3$) δ 155.2 (ArC), 150.0 (ArCH), 143.4 (ArC), 137.1 (ArCH), 132.5 (ArCH), 127.4 (ArCH), 123.3 (ArCH), 120.9 (ArCH), 118.8 (ArC), 112.4 (ArCN); **HRMS** (APCI) $C_{12}H_9N_2$ $[M+H]^+$: calculated 181.0760, found 181.0762; $\boldsymbol{v}_{\mathbf{max}}$ (thin film/cm^{-1}) 2227, 1608, 1465, 1432, 1304, 1016, 989, 773, 717, 619. Data consistent with the literature[14]

2-(2-(Methylthio)phenyl)pyridine 6b

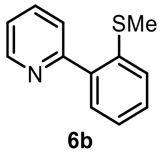

6b

Following GP5, to 2-bromopyridine (61 µL, 0.63 mmol, 1 equiv.) at RT was added 1.3 M isopropylmagnesium(II) chloride lithium chloride in THF (0.58 mL, 0.76 mmol, 1.2 equiv.). The reaction was stirred for 3 hours at RT before use. In a separate flask, a solution of **2s-DBT-**

Cl (72 mg, 0.20 mmol, 1.0 equiv.) in THF (2 mL) was cooled to -78 °C, and the preformed aryl Grignard reagent (0.4 mL, 0.40 mmol, 2.0 equiv.) was added. The product was purified by column chromatography on silica gel using 15:85 acetone:hexanes as eluent to give 2-(2-(methylthio)phenyl)pyridine **6b** (20 mg, 0.099 mmol, 50%) as a colorless oil. **^{1}H NMR** (500 MHz, $CDCl_3$) δ 8.65 (d, J = 4.9 Hz, 1H, ArCH), 7.68 (td, J = 7.7, 1.8 Hz, 1H, ArCH), 7.49 (dt, J = 7.9, 1.0 Hz, 1H, ArCH), 7.36 (dd, J = 7.5, 1.5 Hz, 1H, ArCH), 7.32 – 7.26 (m, 2H, ArCH), 7.23 – 7.13 (m, 2H, ArCH), 2.32 (s, SCH_3); **^{13}C NMR** (126 MHz, $CDCl_3$) δ 158.4 (ArC), 149.1 (ArCH), 139.6 (ArC), 137.3 (ArC), 136.1 (ArCH), 129.9 (ArCH), 128.8 (ArCH), 126.0 (ArCH), 124.9 (ArCH), 124.1 (ArCH), 122.1 (ArCH), 16.4 (CH_3); **HRMS** (APCI) $C_{12}H_{11}NS$ $[M+H]^+$: calculated 202.0685, found 202.0688; $\boldsymbol{\nu}_{\mathbf{max}}$ (thin film/cm^{-1}) 3056, 2926, 1581, 1478, 1463, 1429, 1254, 1086, 989, 802, 747,682, 631. Data consistent with the literature[15]

2-(4-(Trifluoromethyl)phenyl)pyridine 6c

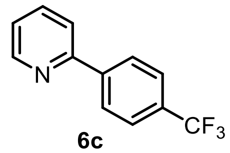

6c

Following GP5, to 2-bromopyridine (61 µL, 0.63 mmol, 1 equiv.) at RT was added 1.3 M isopropylmagnesium(II) chloride lithium chloride in THF (0.58 mL, 0.76 mmol, 1.2 equiv.). The reaction was stirred for 3 hours at RT before use. In a separate flask, a solution of **2f-DBT-Cl** (73 mg, 0.20 mmol, 1.0 equiv.) in THF (2 mL) was cooled to -78 °C, and the preformed aryl Grignard reagent (0.4 mL, 0.40 mmol, 2.0 equiv.) was added. The product was purified by column chromatography on silica gel using 15:85 acetone:hexanes as eluent to give 2-(4-(trifluoromethyl)phenyl)pyridine **6c** (19 mg, 0.086 mmol, 43%) as a white solid; **^{1}H NMR** (400 MHz, $CDCl_3$) δ 8.73 (d, J = 4.8 Hz, 1H, ArCH), 8.11 (d, J = 8.1 Hz, 2H, ArCH), 7.83 – 7.71 (m, 4H, ArCH), 7.30 (ddd, J = 6.7, 4.7, 1.6 Hz, 1H, ArCH); **^{13}C NMR** (101 MHz, $CDCl_3$) δ 155.9 (ArC), 149.9 (ArCH), 142.6 (ArC), 137.0 (ArCH), 130.8 (q, $J_{C\text{–}F}$ = 32.5 Hz, ArC), 127.2 (ArCH), 125.7 (q, $J_{C\text{–}F}$ = 3.5 Hz, ArCH), 124.2 (q, $J_{C\text{–}F}$ = 271.9 Hz, CF_3), 122.9 (ArCH), 120.8 (ArCH);

^{19}**F NMR** (376 MHz, $CDCl_3$) δ -62.56; **HRMS** (ESI) $C_{12}H_9NF_3$ $[M]^+$: calculated 224.0682, found 224.0684; ν_{max} (thin film/cm^{-1}) 2924, 1615, 1581, 1466, 1438, 1404, 1325, 1155, 1105, 1071, 989, 854, 780, 730, 656. Data consistent with the literature[16]

2,3'-Bipyridine 6d

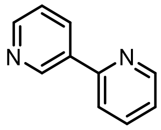

6d

Following GP5, to 2-bromopyridine (61 µL, 0.63 mmol, 1 equiv.) at RT was added 1.3 M isopropylmagnesium(II) chloride lithium chloride in THF (0.58 mL, 0.76 mmol, 1.2 equiv.). The reaction was stirred for 3 hours at RT before use. In a separate flask, a solution of **2a-PXT-Cl** (63 mg, 0.2 mmol, 1.0 equiv.) in THF (2 mL) was cooled to -78 °C, and the preformed aryl Grignard reagent (0.4 mL, 0.4 mmol, 2.0 equiv.) was added. The product was purified by column chromatography on silica gel using 40:60 acetone:hexanes as eluent to give 2,3'-bipyridine **6d** (10 mg, 0.064 mmol, 32%) as a yellow oil; ^{1}**H NMR** (400 MHz, $CDCl_3$) δ 9.21 (d, *J* = 2.3 Hz, 1H, ArCH), 8.79 – 8.64 (m, 2H, ArCH), 8.35 (d, *J* = 9.9 Hz, 1H, ArCH), 7.85 – 7.75 (m, 2H, ArCH), 7.43 (dd, *J* = 8.0, 4.8 Hz, 1H, ArCH), 7.33 (dt, *J* = 7.1, 3.2 Hz, 1H, ArCH); ^{13}**C NMR** (101 MHz, $CDCl_3$) δ 154.8 (ArC), 150.1 (ArCH), 149.9 (ArCH), 148.2 (ArCH), 137.0 (ArCH), 134.9 (ArC), 134.3 (ArCH), 123.6 (ArCH), 122.8 (ArCH), 120.64 (ArCH); **HRMS** (APCI) $C_{10}H_9N_2$ $[M+H]^+$: calculated 157.0760, found 157.0763; ν_{max} (thin film/cm^{-1}) 3056, 2223, 1587, 1460, 1432, 1404, 1017, 905, 770, 723, 645, 554. Data consistent with the literature[17]

2-(Benzo[*b*]thiophen-4-yl)pyridine 6e

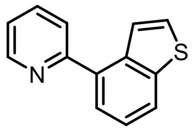

6e

Following GP5, to 2-bromopyridine (61 µL, 0.63 mmol, 1 equiv.) at RT was added 1.3 M isopropylmagnesium(II) chloride lithium chloride in THF (0.58 mL, 0.76 mmol, 1.2 equiv.). The reaction was stirred for 3 hours at RT before use. In a separate flask, a solution of **2ac-PXT-Cl** (73 mg, 0.20 mmol, 1.0 equiv.) in THF (2 mL) was cooled to -78 °C, and the preformed aryl Grignard reagent (0.4 mL, 0.60 mmol, 3.0 equiv.) was added. The product, was purified by column chromatography on silica gel using 20:80 acetone:hexanes as eluent to give 2-(benzo[*b*]thiophen-4-yl)pyridine **6e** (28 mg, 0.130 mmol, 65%) as a colorless oil; **^{1}H NMR** (400 MHz, $CDCl_3$) δ 8.81 – 8.75 (m, 1H, ArCH), 7.95 (dt, *J* = 8.0, 1.0 Hz, 1H, ArCH), 7.87 – 7.83 (m, 1H, ArCH), 7.80 (dd, *J* = 7.7, 1.9 Hz, 1H, ArCH), 7.69 (dt, *J* = 7.9, 1.1 Hz, 1H, ArCH), 7.63 (dd, *J* = 7.3, 1.0 Hz, 1H, ArCH), 7.51 (d, *J* = 5.5 Hz, 1H, ArCH), 7.45 (t, *J* = 7.7 Hz, 1H, ArCH), 7.30 (ddd, *J* = 7.5, 4.9, 1.2 Hz, 1H, ArCH); **^{13}C NMR** (101 MHz, $CDCl_3$) δ 158.8 (ArC), 149 (ArCH), 141.0 (ArC), 137.5 (ArC), 136.6 (ArCH), 135.9 (ArC), 126.9 (ArCH), 124.9 (ArCH), 124.2 (ArCH), 123.6 (ArCH), 123.5 (ArCH), 122.9 (ArCH), 122.0 (ArCH); **HRMS** (APCI) $C_{13}H_{10}NS$ $[M+H]^+$: calculated 212.0528, found 212.0531; ***ν_{max}*** (thin film/cm^{-1}) 2924, 1710, 1587, 1472, 1424, 1405, 1222, 1151, 1089, 990, 906, 869, 792, 725, 699.

2-(3-Bromophenyl)pyridine 6f

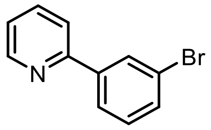

6f

Following GP5, 2-bromopyridine (61 µL, 0.63 mmol, 1 equiv.) at RT was added 1.3 M isopropylmagnesium(II) chloride lithium chloride in THF (0.58 mL, 0.76 mmol, 1.2 equiv.). The reaction was stirred for 3 hours at RT before use. In a separate flask, a solution of **2j-PXT-Cl** (72 mg, 0.20 mmol, 1.0 equiv.) in THF (2 mL) was cooled to -78 °C, and the preformed aryl Grignard reagent (0.4 mL, 0.40 mmol, 2.0 equiv.) was added. The product was purified by column chromatography on silica gel using 20:80 acetone:hexanes as eluent to give 2-(3-bromophenyl)pyridine **6f** (28 mg, 0.12 mmol, 60%) as a colorless oil; **^{1}H NMR** (500 MHz, $CDCl_3$) δ 8.62 (d, *J* = 3.2 Hz, 1H, ArCH), 8.10 (t, *J* = 1.9 Hz, 1H, ArCH), 7.83 (d, *J* = 7.7 Hz, 1H, ArCH), 7.69 (td, *J* = 7.7, 1.8 Hz, 1H, ArCH), 7.63 (d, *J* = 7.9 Hz, 1H, ArCH), 7.49 – 7.45 (m, 1H, ArCH), 7.27 (t, *J* = 7.9 Hz, 1H, ArCH), 7.21 – 7.16 (m, 1H, ArCH); **^{13}C NMR** (126 MHz, $CDCl_3$) δ 155.8 (ArC), 149.8 (ArCH), 141.4 (ArC), 136.9 (ArCH), 131.8 (ArCH), 130.2 (ArCH), 130.0 (ArCH), 125.4 (ArCH), 123.0 (ArC), 122.7 (ArCH), 120.6 (ArCH); **HRMS** (ESI) $C_{11}H_9BrN$ $[M+H]^+$: calculated 233.9918, found 233.9920; $\boldsymbol{\nu_{max}}$ (thin film/cm^{-1}) 3063, 1584, 1456, 1431, 1403, 1276, 1152, 1063, 911, 881, 784, 740, 671, 613. Data consistent with the literature[16]

3-(3-Bromophenyl)pyridine 6g

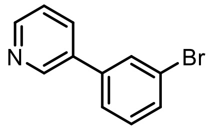

6g

Following GP5, to 3-bromopyridine (61 µL, 0.63 mmol, 1 equiv.) at RT was added 1.3 M isopropylmagnesium(II) chloride lithium chloride in THF (0.58 mL, 0.76 mmol, 1.2 equiv.). The reaction was stirred for 4 hours at RT before use. In a separate flask, a solution of **2j-PXT-Cl** (72 mg, 0.20 mmol, 1.0 equiv.) in THF (2 mL) was cooled to -78 °C, and the preformed aryl

Grignard reagent (0.4 mL, 0.40 mmol, 2.0 equiv.) was added. The product was purified by column chromatography on silica gel using 20:80 acetone:hexanes as eluent to give 3-(3-bromophenyl)pyridine **6g** (25.5 mg, 0.108 mmol, 54%) as a colorless oil; **^{1}H NMR** (400 MHz, $CDCl_3$) δ 8.83 (s, 1H, ArCH), 8.64 (d, *J* = 4.7 Hz, 1H, ArCH), 7.85 (dt, *J* = 7.9, 2.0 Hz, 1H, ArCH), 7.72 (t, *J* = 1.9 Hz, 1H, ArCH), 7.57 – 7.48 (m, 2H, ArCH), 7.43 – 7.31 (m, 2H, ArCH); **^{13}C NMR** (101 MHz, $CDCl_3$) δ 148.9 (ArCH), 148.1 (ArCH), 139.9 (ArC), 135.3 (ArC), 134.5 (ArCH), 131.1 (ArCH), 130.6 (ArCH), 130.2 (ArCH), 125.8 (ArCH), 123.6 (ArCH), 123.2 (ArC); **HRMS** (ESI) $C_{11}H_9BrN$ $[M+H]^+$: calculated 233.9918, found 233.9921; $\boldsymbol{v}_{\mathbf{max}}$ (thin film/cm^{-1}) 3031, 1573, 1465, 1426, 1388, 1188, 1075, 1012, 811, 775, 708, 689, 639. Data consistent with the literature[16].

12.5 Preliminary study- a Pd-catalysed cross coupling

3-(4-(Trifluoromethyl)phenyl)pyridine S6

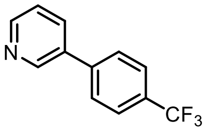

S6

Following G6, Aryl sulfonium salt (51 mg, 0.17 mmol, 1.0 equiv.), trifluoromethyl-phenylboronic acid (38 mg, 0.2 mmol, 1.0 equiv.), Cs_2CO_3 (130 mg, 0.4 mmol, 2 equiv.) and $Pd(OAc)_2$ (0.5 mol %) gave the crude product, which was purified by column chromatography on silica gel using hexane to 7:3 hexane:EtOAc as eluent to give 3-(4-(trifluoromethyl)phenyl)pyridine **S6** (15 mg, 0.067 mmol, 40% isolated yield at 68% conversion) as a white solid. ^{1}H NMR (400 MHz, $CDCl_3$) δ 8.89 (dd, *J* = 2.4, 0.9 Hz, 1H, ArCH), 8.68 (dd, *J* = 4.8, 1.6 Hz, 1H, ArCH), 7.92 (ddd, *J* = 7.9, 2.4, 1.6 Hz, 1H, ArCH), 7.81 – 7.75 (m, 2H, ArCH), 7.75 – 7.70 (m, 2H, ArCH), 7.44 (ddd, *J* = 7.9, 4.8, 0.9 Hz, 1H, ArCH). ^{13}C NMR (101 MHz, $CDCl_3$) δ 149.4 (ArCH), 148.3 (ArCH), 141.4 (ArC), 135.3 (ArC), 134.5 (ArCH), 130.2 (d, $J_{C\text{–}F}$ = 32.7 Hz, ArC), 127.5 (ArCH), 126.0 (q, *J* = 3.7 Hz, ArCH), 124.1 (q, $J_{C\text{–}F}$ = 272.1 Hz, CF_3), 123.7 (ArCH); **^{19}F NMR** (376

MHz, $CDCl_3$) δ -62.25; **HRMS** (ESI) $C_{12}H_8F_3N$ $[M+H]^+$: calculated 223.0609 found 223.0616;.

Data consistent with the literature[18]

13. Telescoped Approach to Ligand Coupling

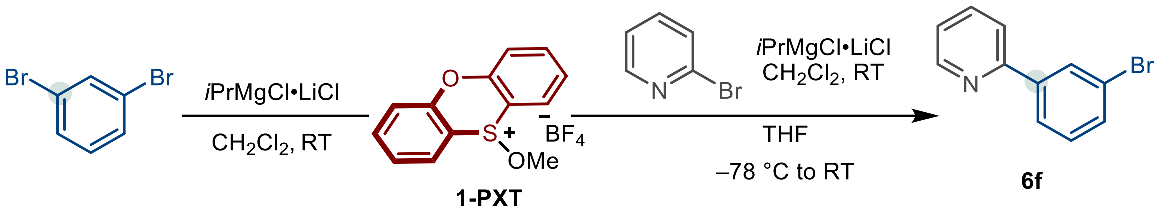

Preformed Grignard A: To an oven-dried vial containing 1,3-dibromobenzene (30 μL, 0.24 mmol, 1.2 equiv.) at RT was added 1.3 M isopropylmagnesium(II) chloride lithium chloride in THF (0.2 mL, 0.26 mmol, 1.3 equiv.). The reaction was stirred for 5 hours at RT before use.

Preformed Grignard B: To an oven-dried vial containing bromopyridine (61 μL, 0.63 mmol, 1 equiv.) at RT was added 1.3 M isopropylmagnesium(II) chloride lithium chloride in THF (0.58 mL, 0.76 mmol, 1.2 equiv.). The reaction was stirred for 3 hours at RT before use.

To an oven-dried vial, the preformed Grignard **A** was added to a solution of methoxysulfonium salt (65 mg, 0.2 mmol, 1 equiv.) in CH_2Cl_2 (0.1M) at -78 °C and stirred for 10 min. The solution was then warmed to RT and stirred for a further 2 h. The reaction was quenched with methanol (50-70 μL) and concentrated to afford the desired sulfonium salt. The vial was then sealed, evacuated and backfilled with nitrogen three times. Anhydrous THF (0.1 M) was added and the solution cooled to -78 °C. The preformed Grignard **B** prepared from 2-bromopyridine (0.4 mL, 0.4 mmol, 2 equiv.) was added to the reaction mixture and the resulted solution stirred for 10 min at -78 °C, then warmed to RT and stirred for a further 2 h. After this time, saturated aqueous NH_4Cl was added, and the aqueous layer was extracted with CH_2Cl_2 (10 mL). The combined organic layers were washed with brine (10 mL), dried using $MgSO_4$, filtered, and concentrated *in vacuo*, to give the crude product. Purification by column chromatography on silica gel using 20:80 acetone:hexanes as eluent gave 2-(3-bromophenyl)pyridine **6f** (26 mg, 0.11 mmol, 55%) as a colorless oil.

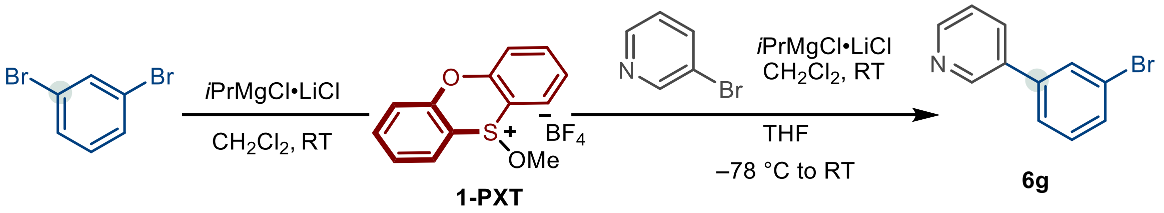

To an oven-dried vial, the preformed Grignard **A** was added to the solution of methoxysulfonium salt (65 mg, 0.2 mmol, 1 equiv.) in CH_2Cl_2 (0.1M) at -78 °C and stirred for 10 min. The solution was then warmed to RT and stirred for a further 2 h. The reaction was then quenched with methanol (50-70 μL) and concentrated to afford the desired sulfonium salt. The vial was then sealed, evacuated and backfilled with nitrogen three times. Anhydrous THF (0.1 M) was then added and the solution cooled to -78 °C. The preformed Grignard **B** prepared from 3-bromopyridine (0.4 mL, 0.4 mmol, 2 equiv.) was added and the resulted mixture stirred for 10 min at -78 °C, then warmed to RT and stirred for a further 2 h. After this time, saturated aqueous NH_4Cl was added, and the aqueous layer was extracted with CH_2Cl_2 (10 mL). The combined organic layers were washed with brine (10 mL), dried using $MgSO_4$, filtered, and concentrated *in vacuo*, to give the crude product. Purification by column chromatography on silica gel using 20:80 acetone:hexanes as eluent gave 3-(3-bromophenyl)pyridine **6g** (25 mg, 0.104 mmol, 52%) as a colorless oil.

14. X-ray Crystal Structures

All data collections, crystal structure determinations and refinements were carried out by the X-ray crystallography service (Dr George Whitehead, Dr Inigo J. Vitorica-Yrezabal, and Avantika Hasija) at The University of Manchester.

Data collection

X-ray data was collected on a Rigaku FR-X DW rotating anode 1.54184 Å) with an AFC-11 RINC goniometer and a Rigaku Hypix 6000 HE photon counting detector. The diffractometer was equipped with an Oxford Cryosystems Cryostream 800 plus nitrogen flow gas system.

Crystal structure determinations and refinements

X-ray data were processed and reduced using CrysAlisPro suite of programs by. The crystal structures were solved and refined against all F2 values using the SHELX and Olex 2 suite of programs. All the non-hydrogen atoms were refined anisotropically. Hydrogen atoms were placed in a calculated position refined using idealised geometries (riding model) and assigned fixed isotropic displacement parameters. Some carbon atoms were found disordered and modelled over two positions were possible. In such cases, C–C bond distances were restrained using FIX and SADI commands. The atomic displacement parameters (adp) of the disordered atoms have been restrained using RIGU command.

5-Methoxy-5H-dibenzo[b,d]thiophen-5-ium tetrafluoroborate 1-DBT

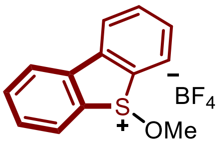

1-DBT

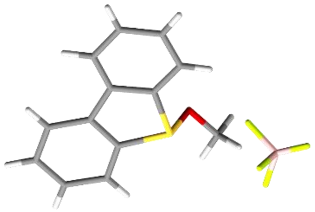

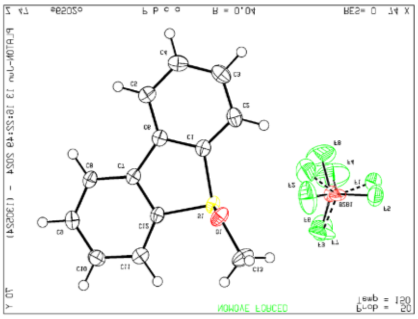

Crystal data structure and refinement

Identification code – 5-methoxy-5H-dibenzo[b,d]thiophen-5-ium tetrafluoroborate

Empirical formula – $C_{13}H_{11}BF_4OS$

Molecular weight – 302.09

Temperature (K) – 150.00(10)

Crystal system – Orthorhombic

Space group – P b c a

a (Å) – 8.2921(3)

b (Å) – 16.6460(8)

c (Å) – 18.6132(9)

α (°) – 90

β (°) – 90

γ (°) – 90

Volume ($Å^3$) – 2569.2(2)

Z – 8

P_{calc} (g cm^{-3}) – 1.562

μ (mm^{-1}) – 0.291

F(100) – 1232.0

Radiation – MoKα (λ = 0.71073)

Reflections collected – 14293

Independent reflections – 3143

2Θ range for data collection (°) – 5.91 to 58.946

Index ranges – $-11 \leq h \leq 8$, $-21 \leq k \leq 20$, $-23 \leq l \leq 19$

Goodness-of-fit on F^2 – 1.047

Final R Indexes [I>=2σ (I)] – R_1 = 0.0384 wR_2 = 0.0938

Final R Indexes [all data] – R_1 = 0.0515 wR_2 = 0.1023

5-(Pyridin-3-yl)-5*H*-dibenzo[*b,d*]thiophen-5-ium chloride 2a-DBT-Cl

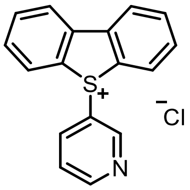

2a-DBT-Cl

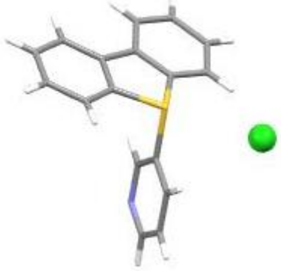

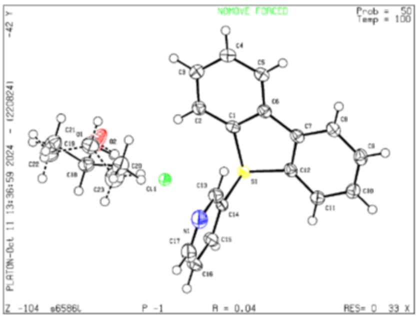

Crystal data structure and refinement

Identification code – 5-(pyridin-3-yl)-5*H*-dibenzo[*b,d*]thiophen-5-ium chloride

Empirical formula – $C_{20}H_{20}ClNOS$

Molecular weight – 357.88

Temperature (K) – 100.00(11)

Crystal system – triclinic

Space group – P -1

a (Å) – 8.2212(2)

b (Å) – 9.3432(3)

c (Å) – 12.4765(4)

α (°) – 108.879(3)

β (°) – 104.772(2)

γ (°) – 94.071(2)

Volume ($Å^3$) – 864.39(5)

Z – 2

P_{calc} (g cm^{-3}) – 1.375

μ (mm^{-1}) – 3.123

F(100) – 376

Radiation – CuKα (λ = 1.54184)

Reflections collected – 8723

Independent reflections – 3132

2Θ range for data collection (°) – 10.15 to 136.496

Index ranges – -9 ≤ h ≤ 9, -11 ≤ k ≤ 11, -14 ≤ l ≤ 13

Goodness-of-fit on F2 – 1.068

Final R Indexes [I>=2σ (I)] – R1 = 0.0390 wR2 = 0.1059

Final R Indexes [all data] – R1 = 0.0400 wR2 = 0.1067

5-(3,5-Dimethoxyphenyl)-5*H*-dibenzo[*b,d*]thiophen-5-ium chloride 2z-DBT-Cl

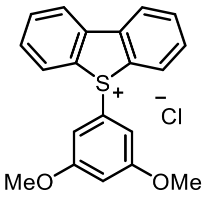

2z-DBT-Cl

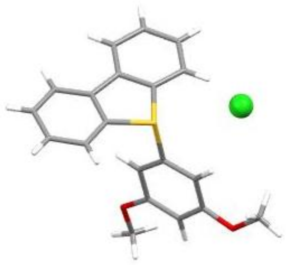

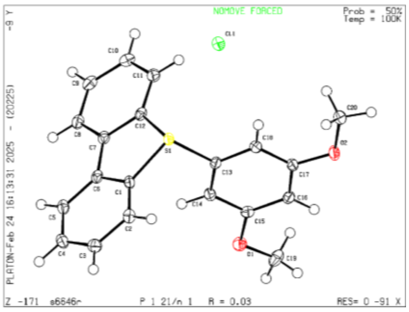

Crystal data structure and refinement

Identification code – 5-(3,5-dimethoxyphenyl)-5*H*-dibenzo[*b,d*]thiophen-5-ium chloride

Empirical formula – $C_{20}H_{17}ClO_2S$

Molecular weight – 356.85

Temperature (K) – 99.99(10)

Crystal system – monoclinic

Space group – P 1 21/n 1

a (Å) – 11.23630(9)

b (Å) – 11.97461(12)

c (Å) – 12.27987(11)

α (°) – 90

β (°) – 91.0649(8)

γ (°) – 90

Volume (Å^3) – 1651.97(3)

Z – 4

P_{calc} (g cm^{-3}) – 1.435

μ (mm^{-1}) – 3.300

F(100) – 744

Radiation – CuKα (λ = 1.54184)

Reflections collected – 24863

Independent reflections – 3486

2Θ range for data collection (°) – 10.32 to 155.958

Index ranges – -14 ≤ h ≤ 10, -15 ≤ k ≤ 15, -15 ≤ l ≤ 15

Goodness-of-fit on F2 – 1.058

Final R Indexes [I>=2σ (I)] – R1 = 0.0297 wR2 = 0.0801

Final R Indexes [all data] – R1 = 0.0316 wR2 = 0.0813

15. NMR Spectra

^{1}H NMR (400 MHz $CDCl_3$) of **S1**

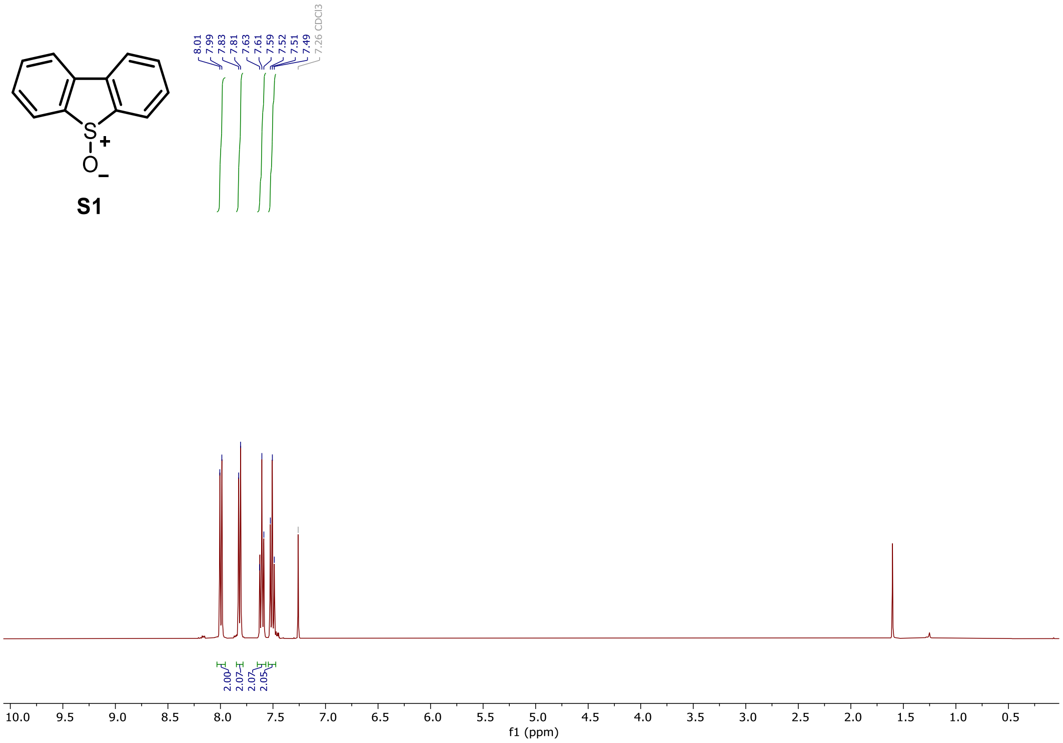

^{13}C NMR (101 MHz $CDCl_3$) of **S1**

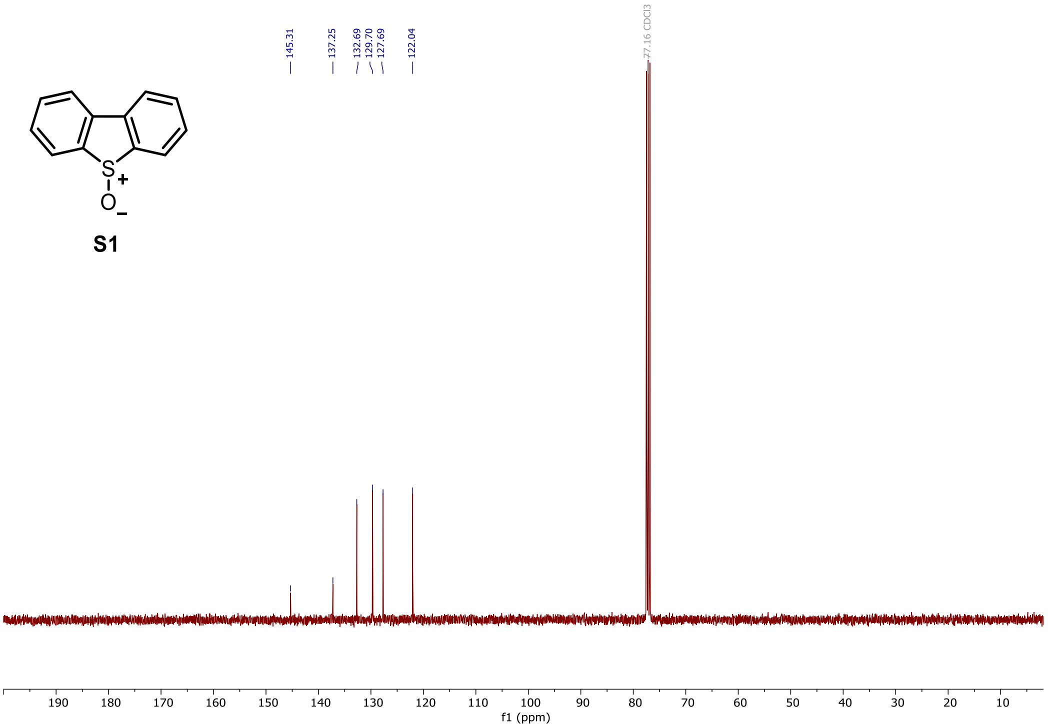

^{1}H NMR (400 MHz $CDCl_3$) of **S2**

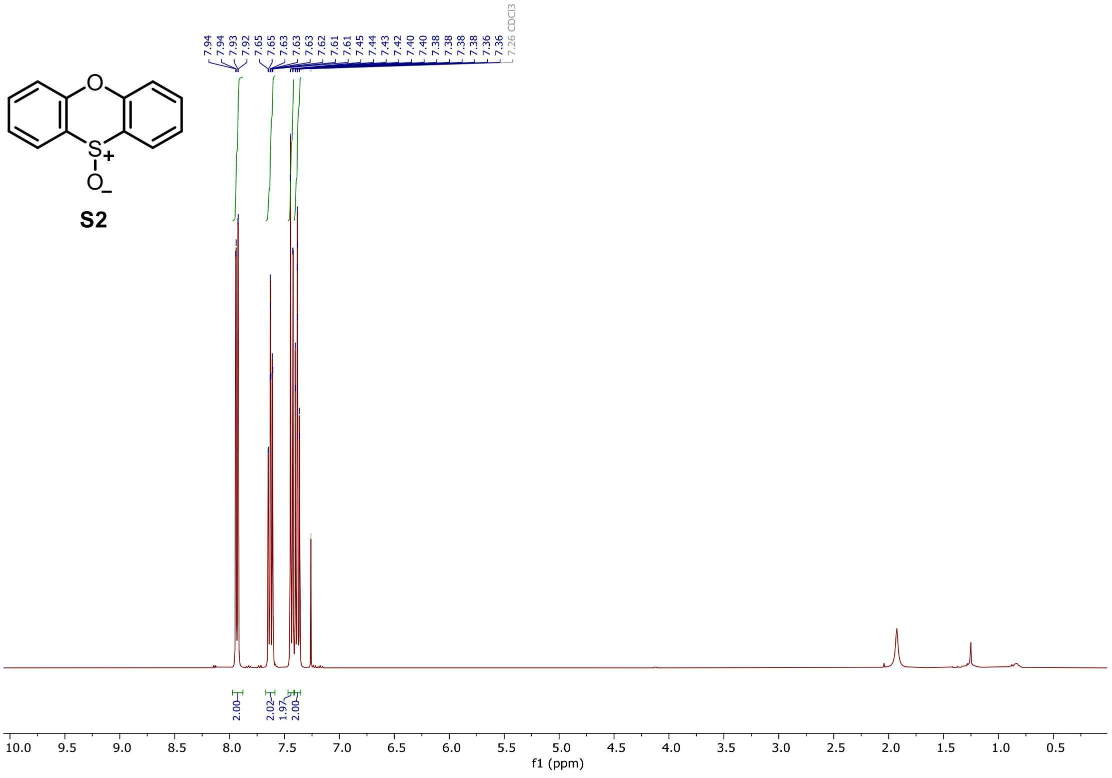

^{13}C NMR (101 MHz $CDCl_3$) of **S2**

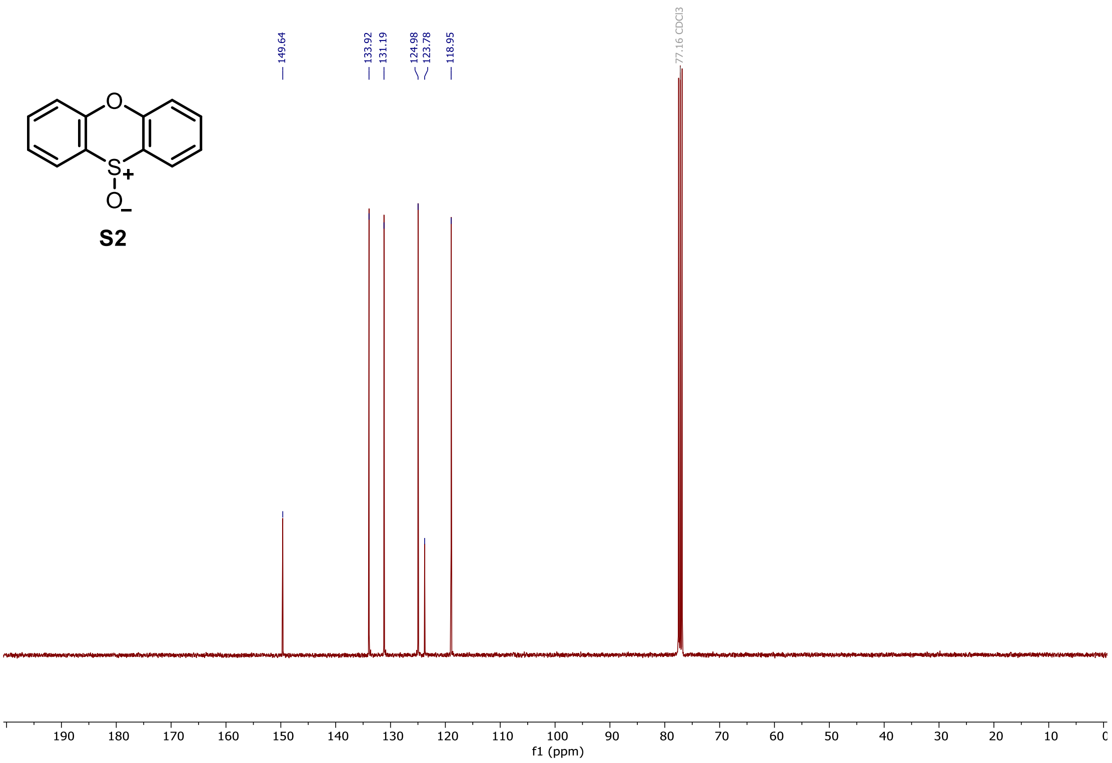

^{1}H NMR (400 MHz, $CDCl_3$) of **S3**

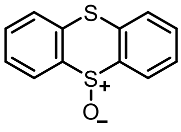

S3

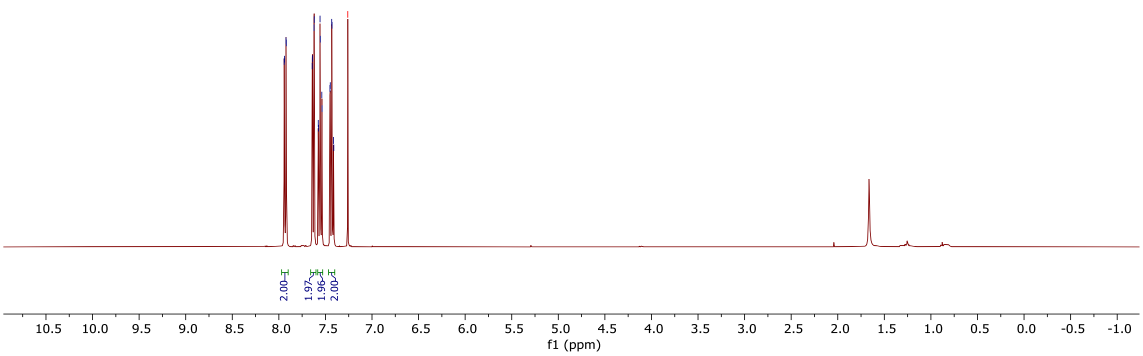

^{13}C NMR (126 MHz, $CDCl_3$) of **S3**

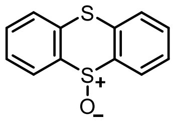

S3

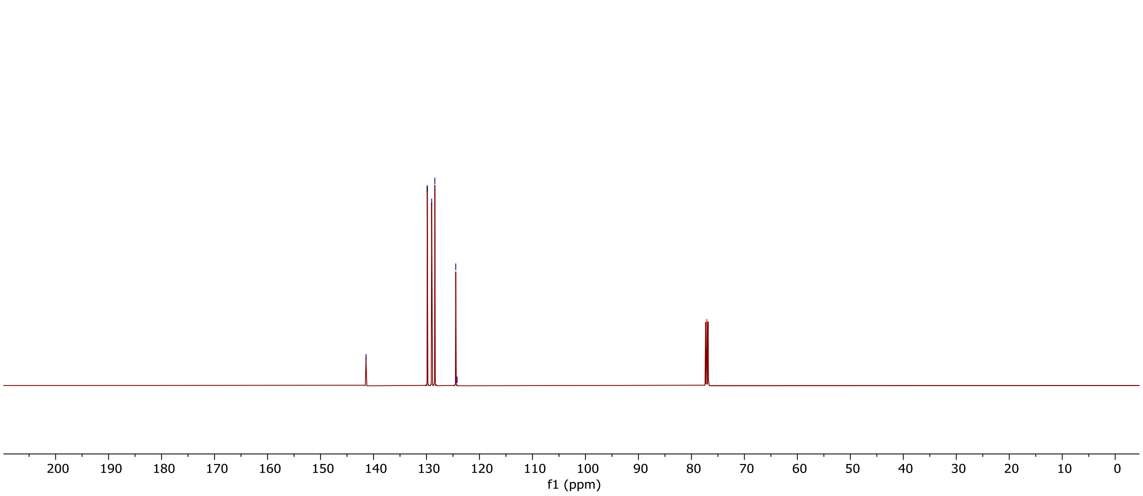

^{1}H NMR (500 MHz CD_3CN) of **1-DBT**

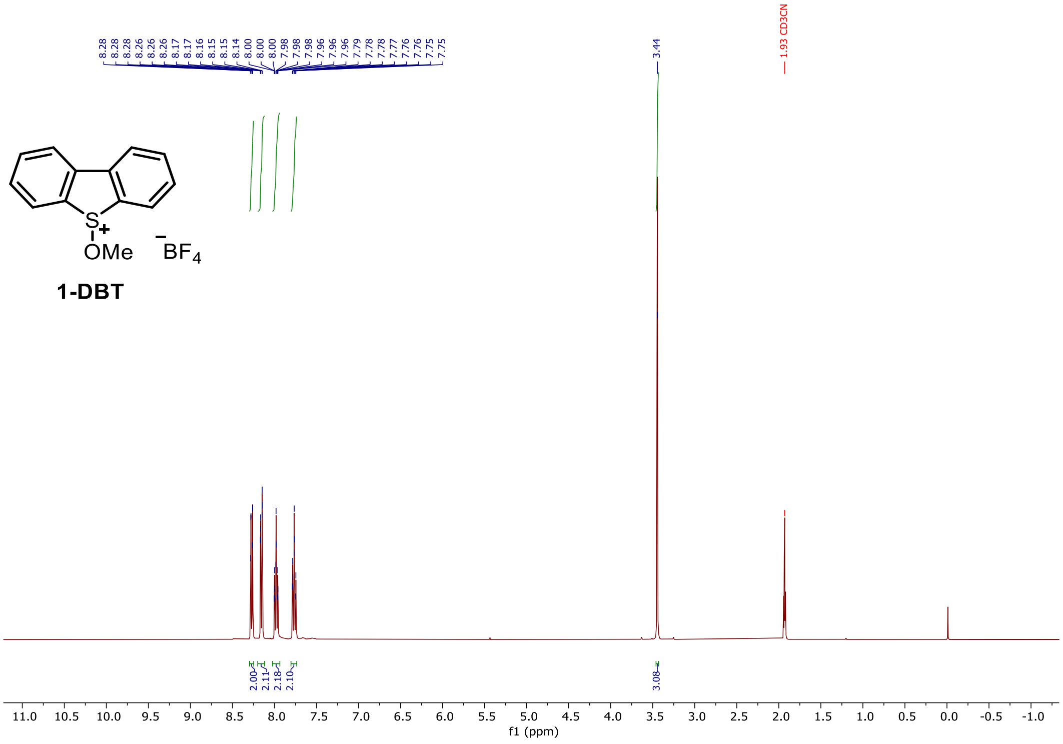

^{13}C NMR (126 MHz CD_3CN) of **1-DBT**

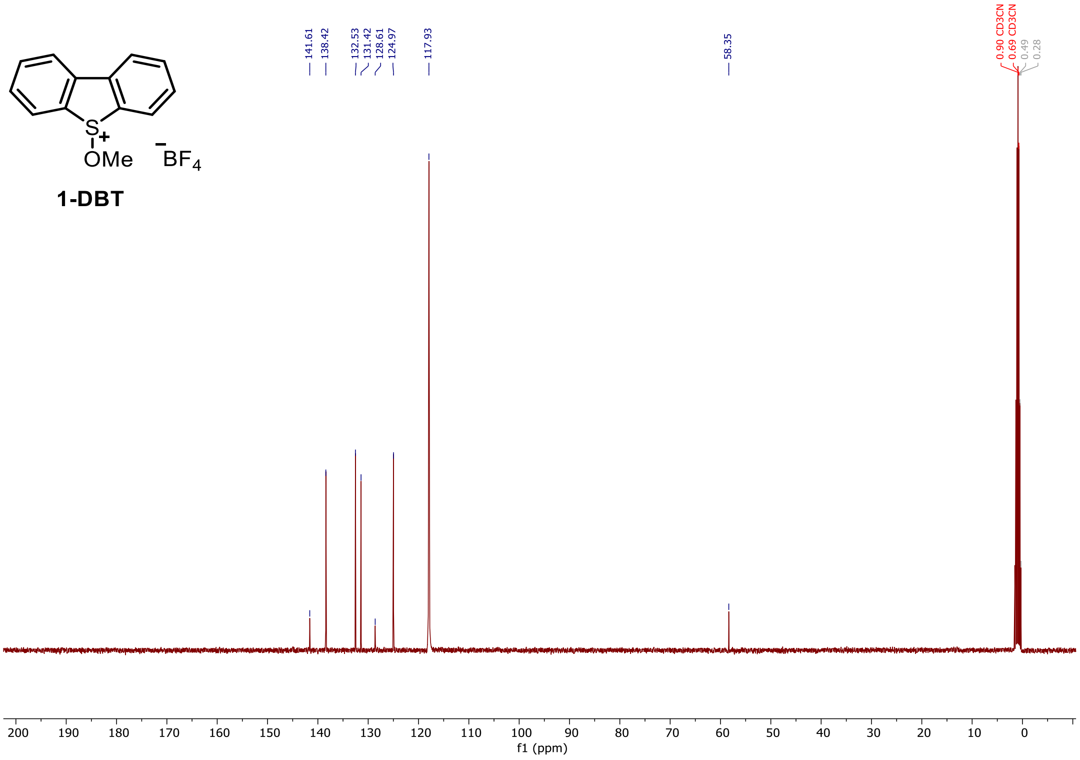

^{19}F NMR (376 MHz, CD_3CN) of **1-DBT**

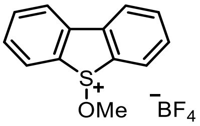

1-DBT

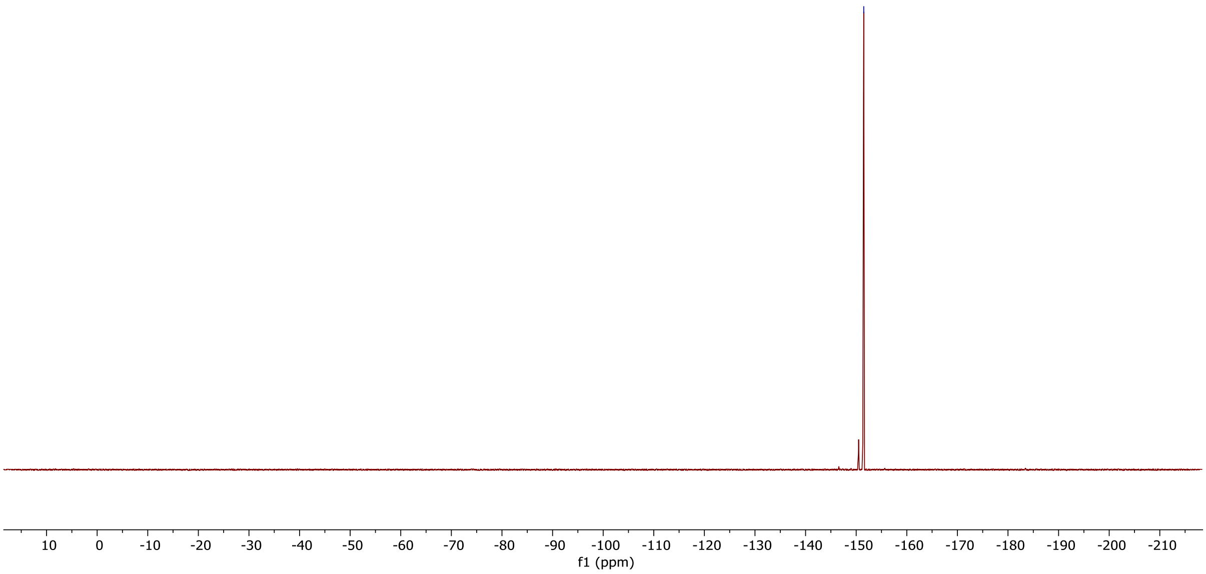

^{1}H NMR (400 MHz CD_3CN) of **1-PXT**

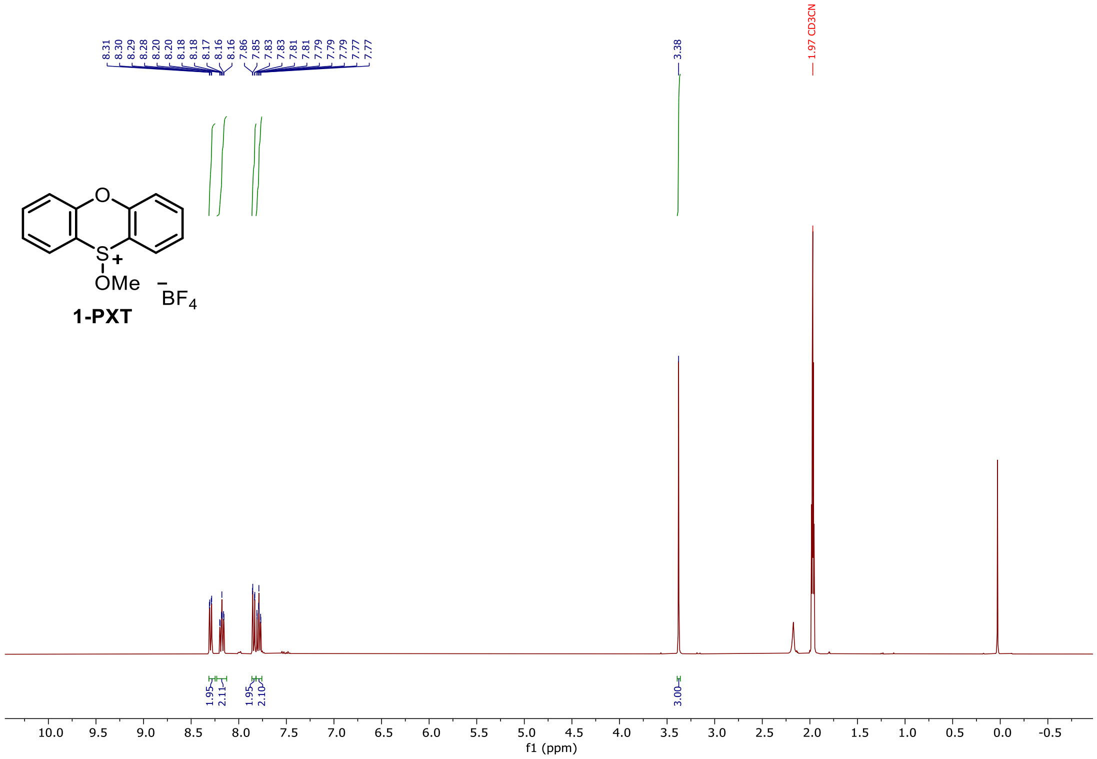

^{13}C NMR (126 MHz CD_3CN) of **1-PXT**

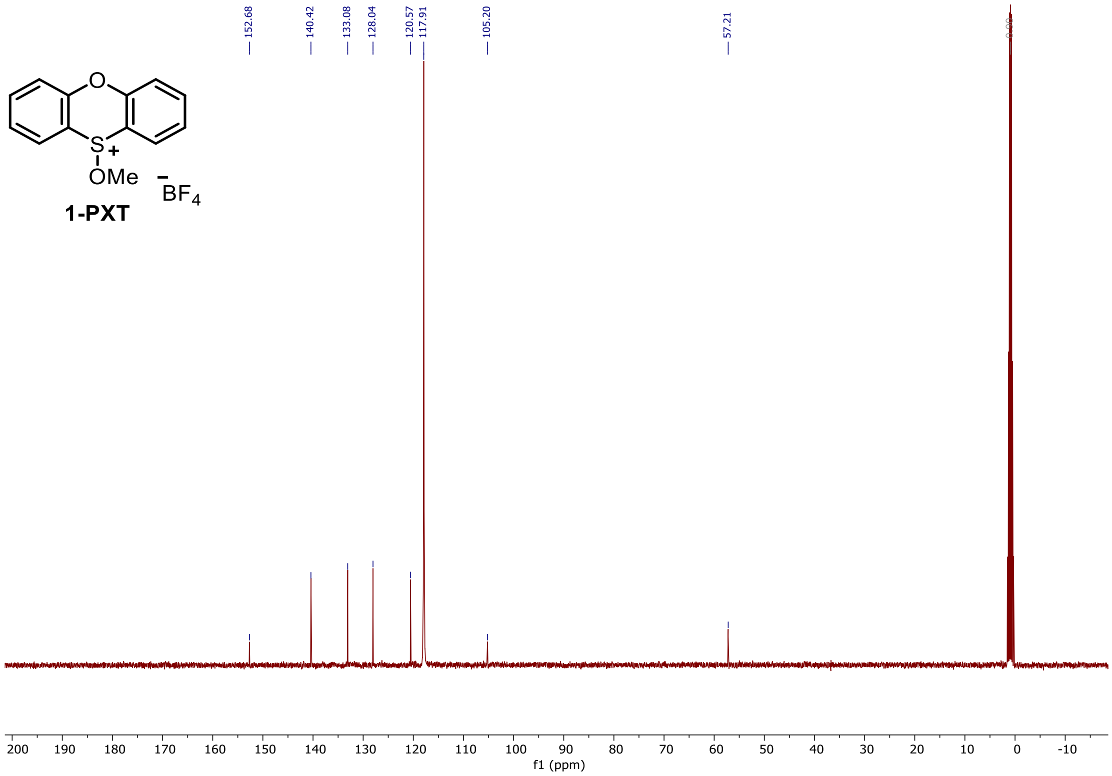

^{19}F NMR (376 MHz, CD_3CN) of **1-PXT**

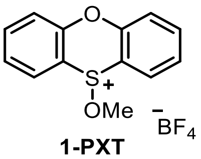

1-PXT

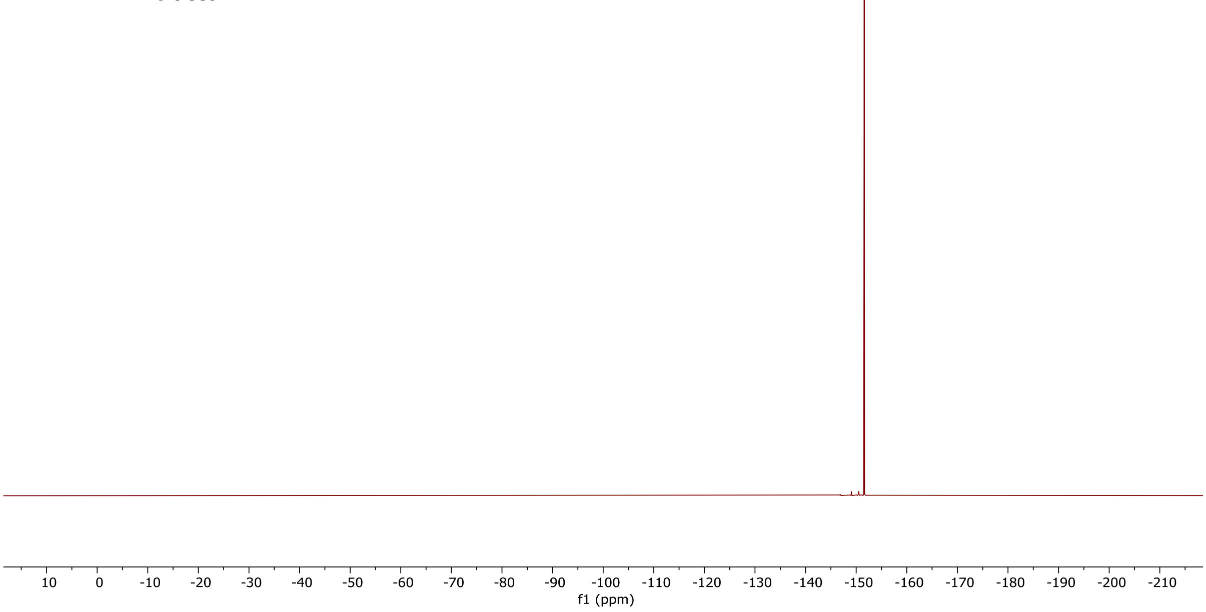

^{1}H NMR (400 MHz, CD_3CN) of **1-TT**

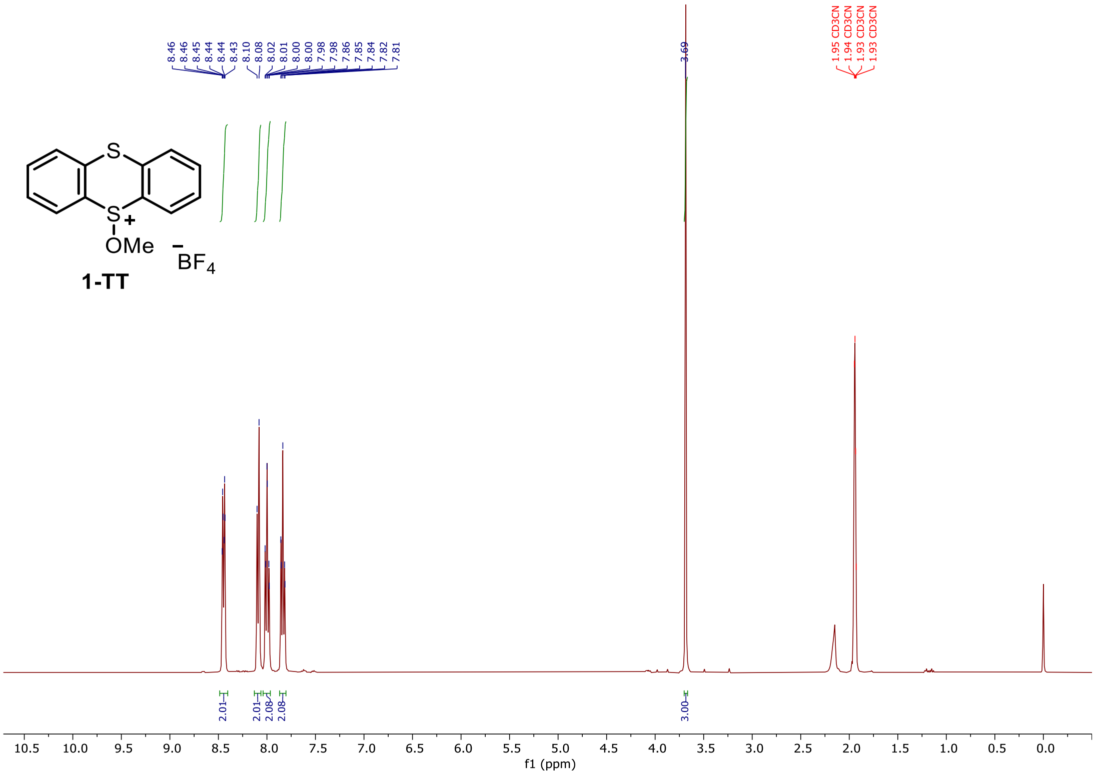

^{13}C NMR (101 MHz, CD_3CN) of **1-TT**

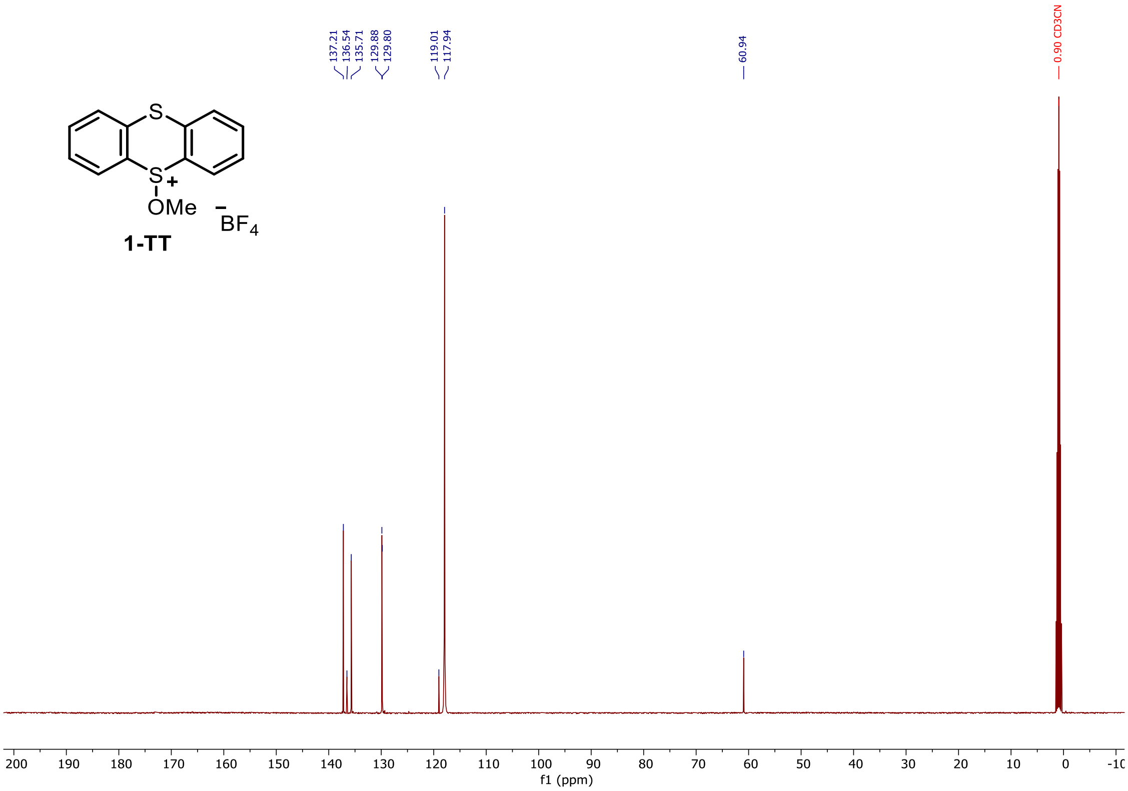

^{19}F NMR (471 MHz, CD_3CN) of **1-TT**

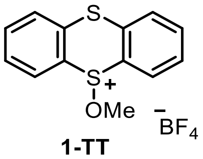

1-TT

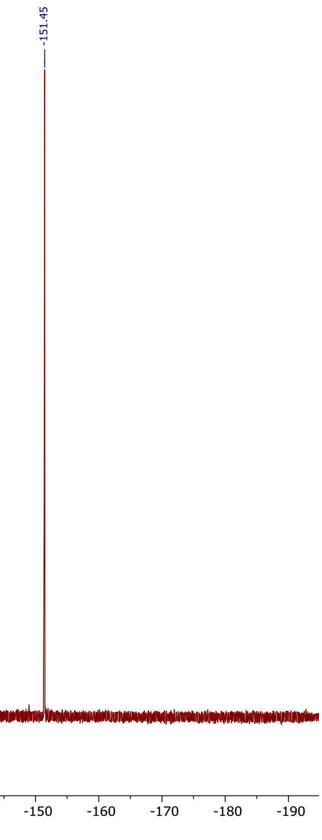

^{1}H NMR (500 MHz DMSO-d_6) of **2a-DBT-Cl**

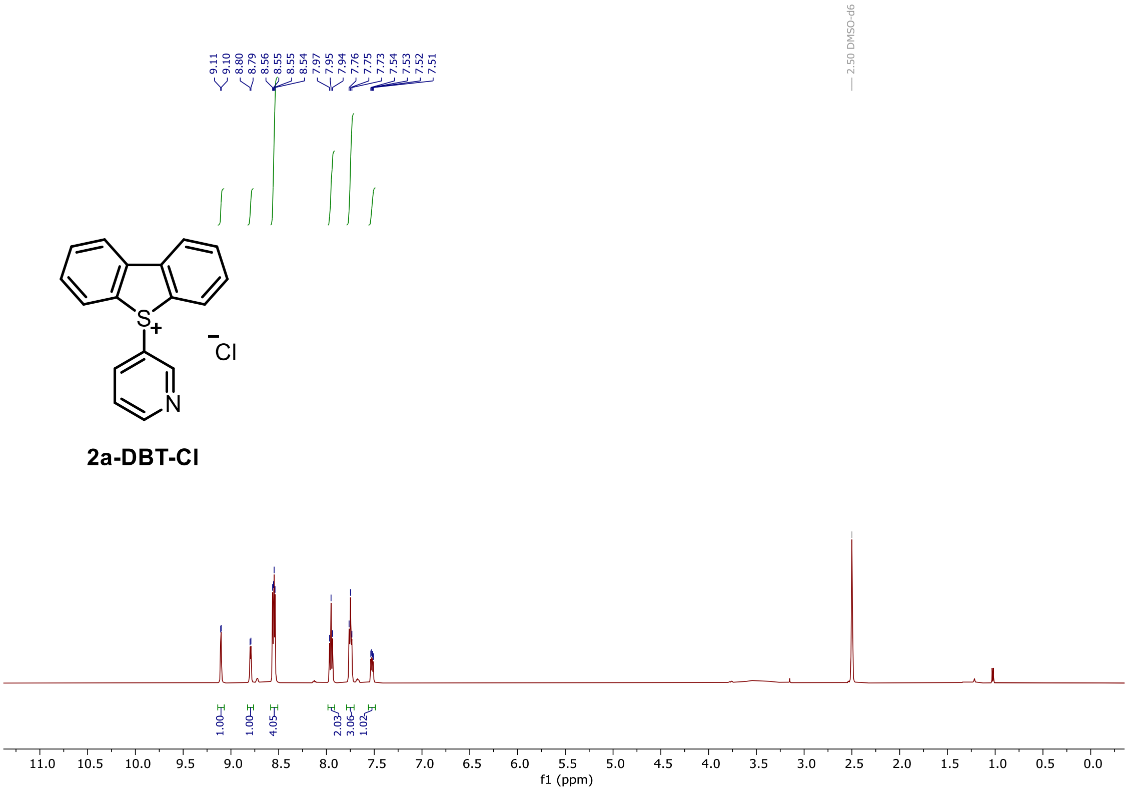

^{13}C NMR (126 MHz DMSO-d_6) of **2a-DBT-Cl**

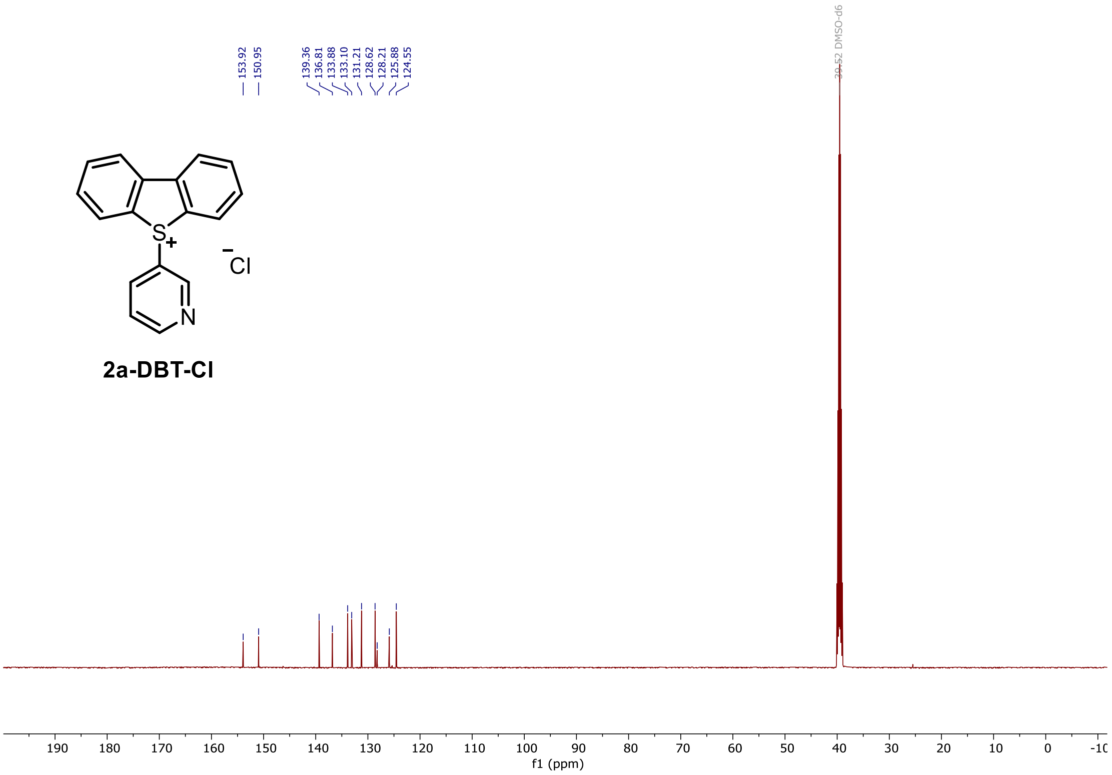

^{1}H NMR (400 MHz DMSO-d$_6$) of **2a-DBT-BF$_4$**

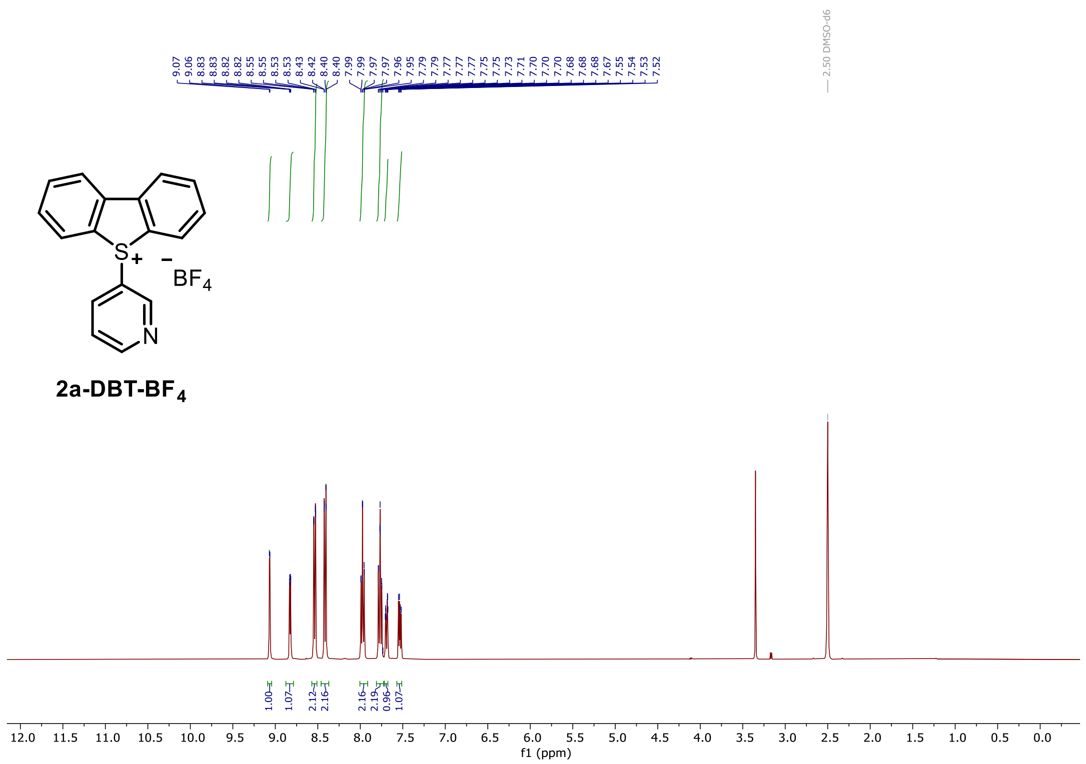

^{13}C NMR (101 MHz DMSO-d$_6$) **of 2a-DBT-BF$_4$**

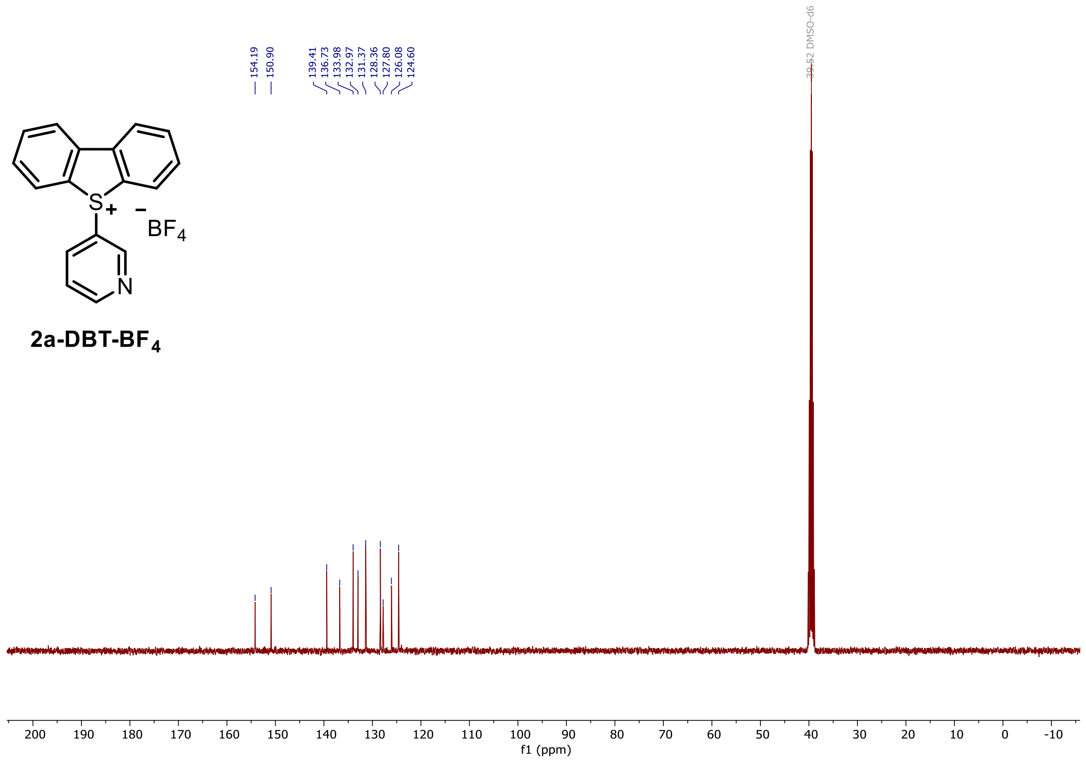

^{1}H NMR (400 MHz, DMSO-d$_6$) of **2a-PXT-Cl**

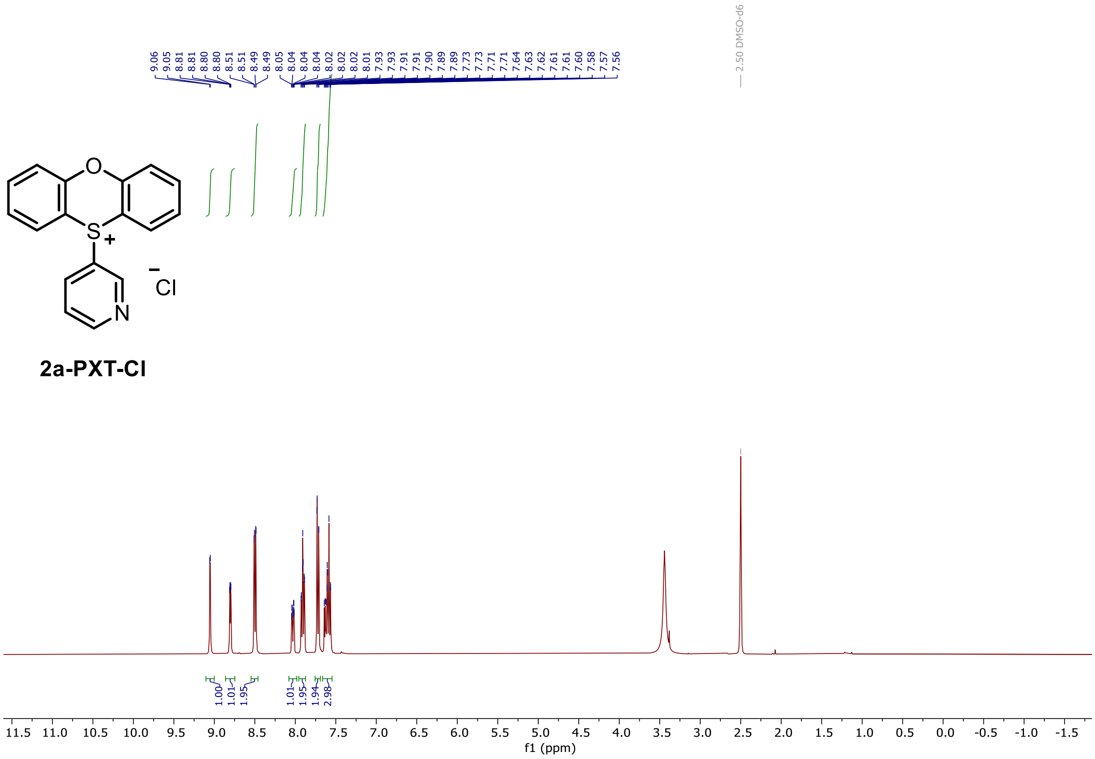

^{13}C NMR (101 MHz, DMSO-d$_6$) of **2a-PXT-Cl**

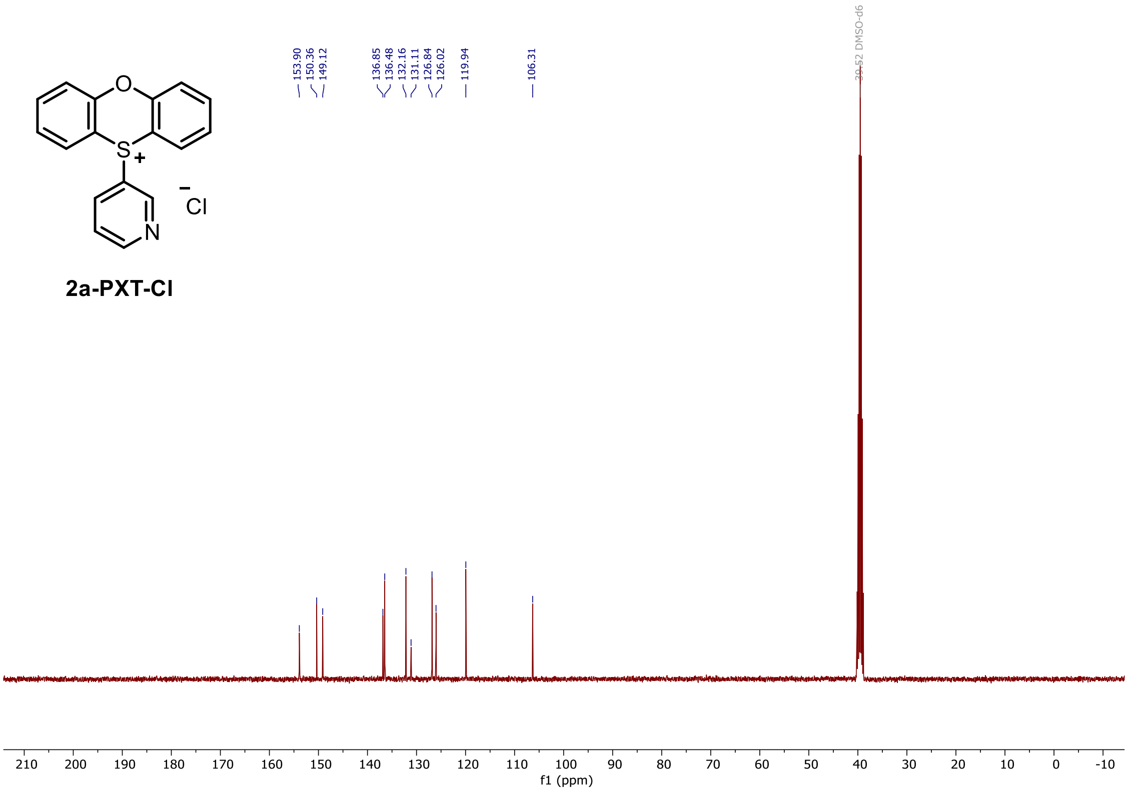

^1H NMR (400 MHz, DMSO-d$_6$) of **2a-PXT-BF$_4$**

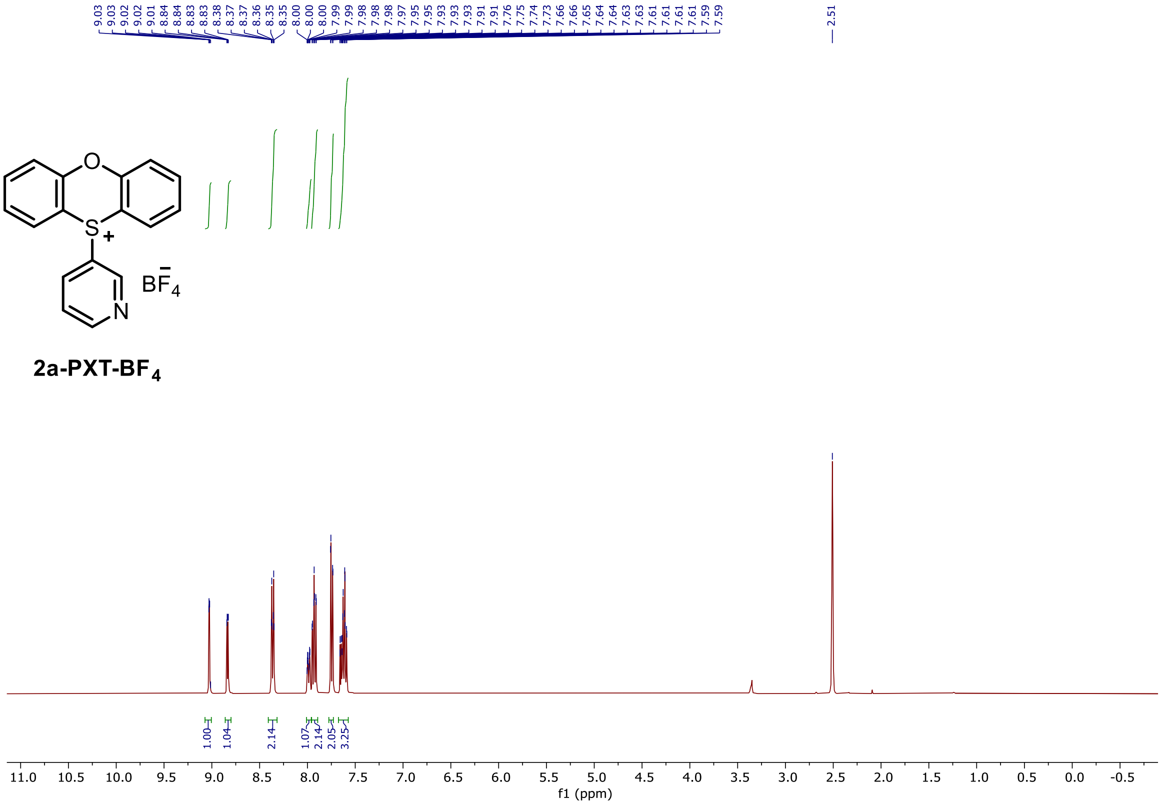

^{13}C NMR (101 MHz, DMSO-d$_6$) of **2a-PXT- BF$_4$**

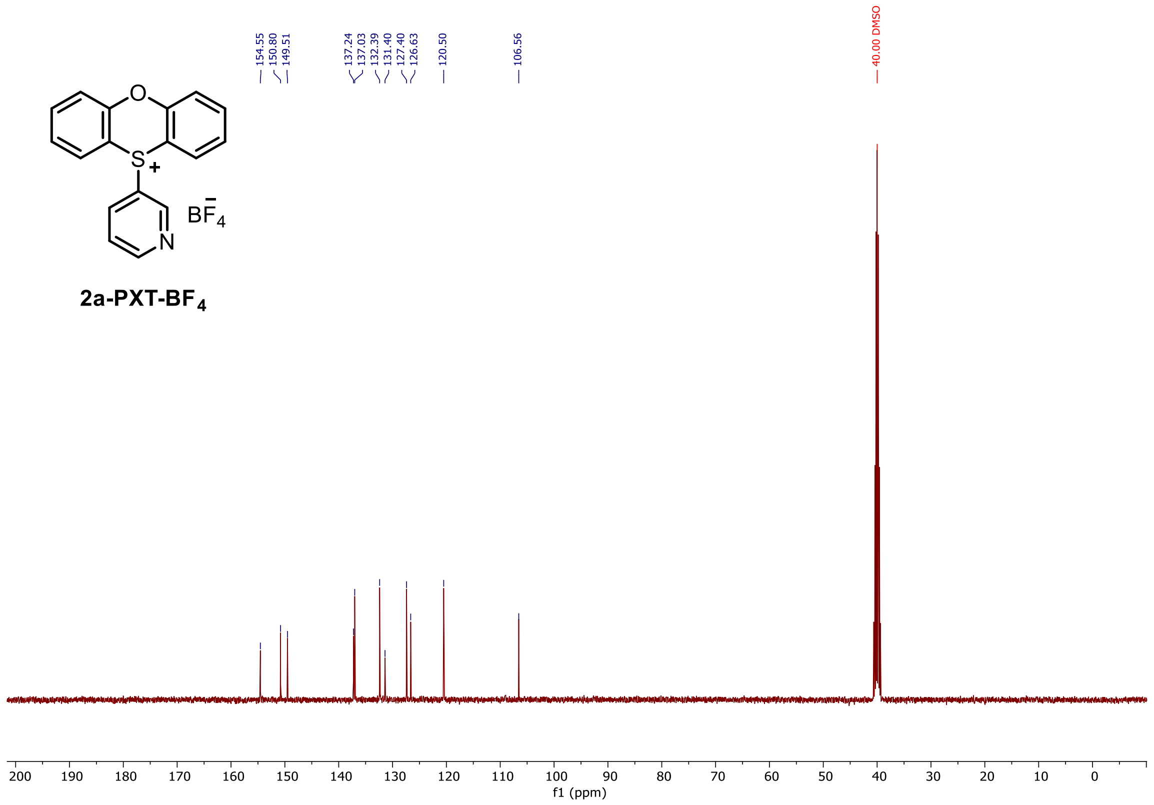

^{19}F NMR (376 MHz, DMSO-d$_6$) of **2a-PXT-BF$_4$**

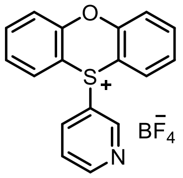

2a-PXT-BF$_4$

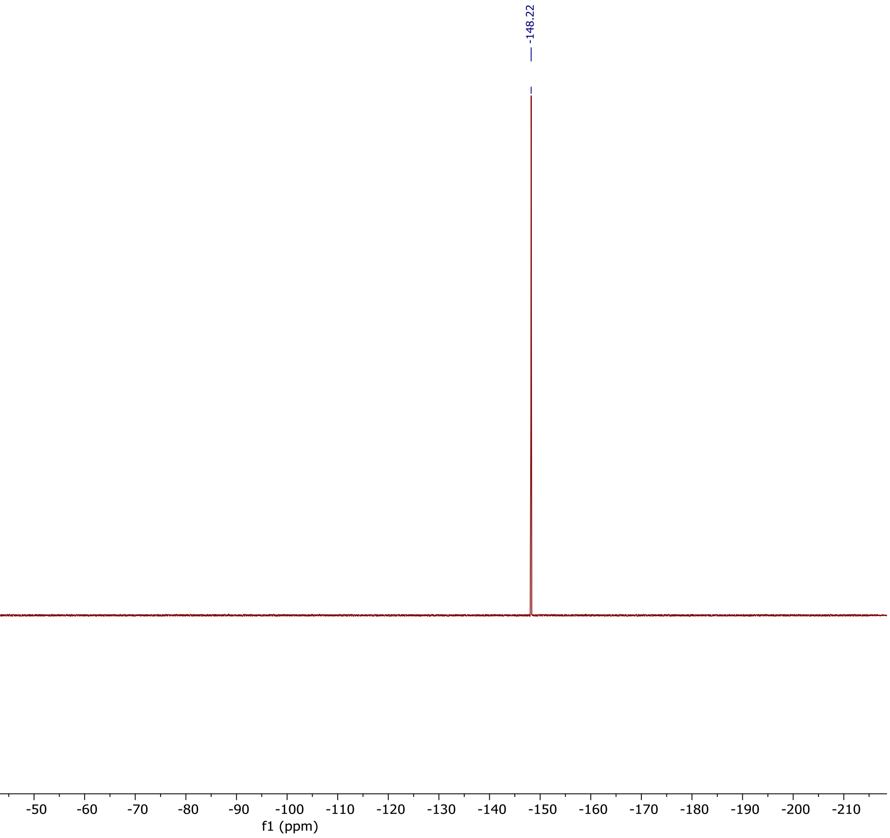

^{1}H NMR (400 MHz, DMSO-d6) of **2a-TT-Cl**

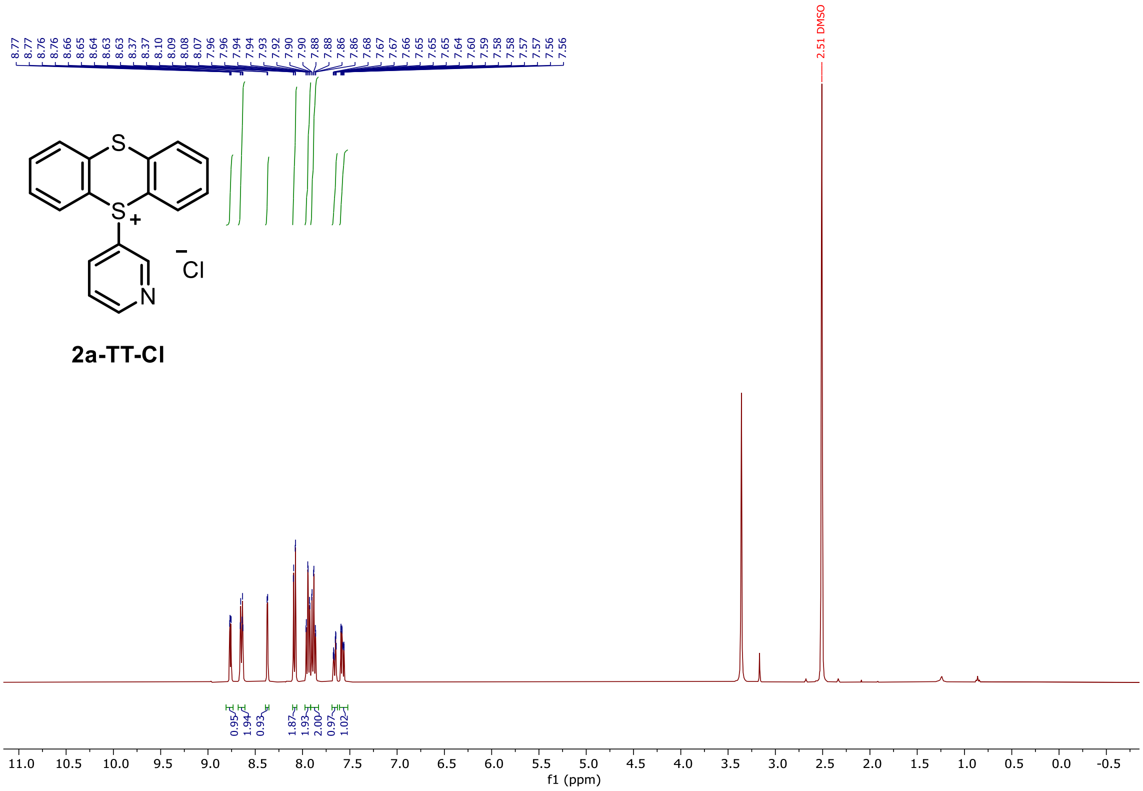

^{13}C NMR (101 MHz, DMSO-d6) of **2a-TT-Cl**

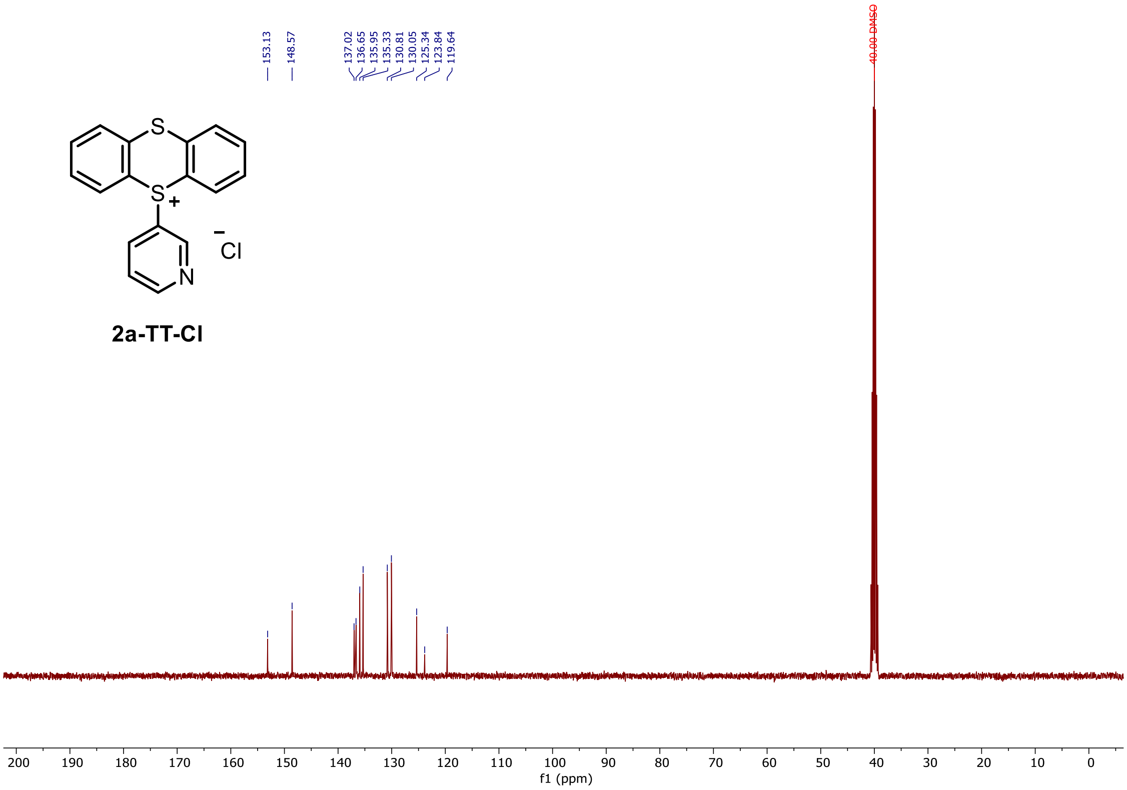

^{1}H NMR (400 MHz, DMSO-d_6) of **2a-TT-BF$_4$**

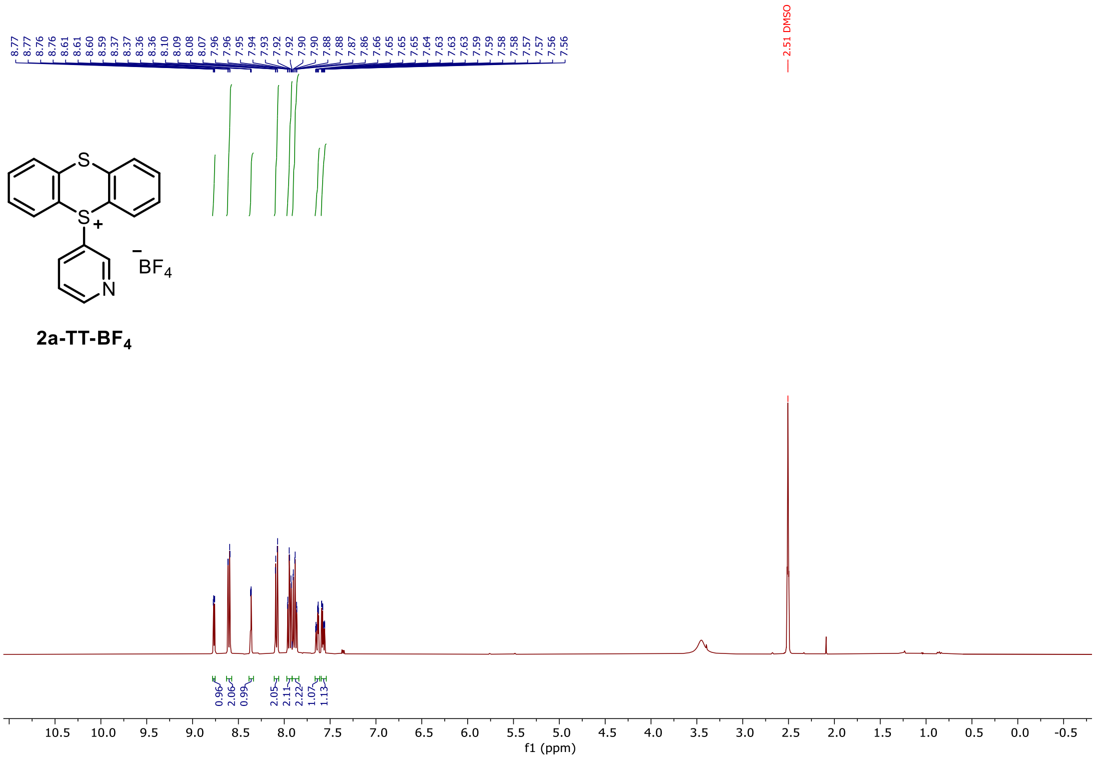

^{13}C NMR (101 MHz, DMSO-d_6) of **2a-TT-BF$_4$**

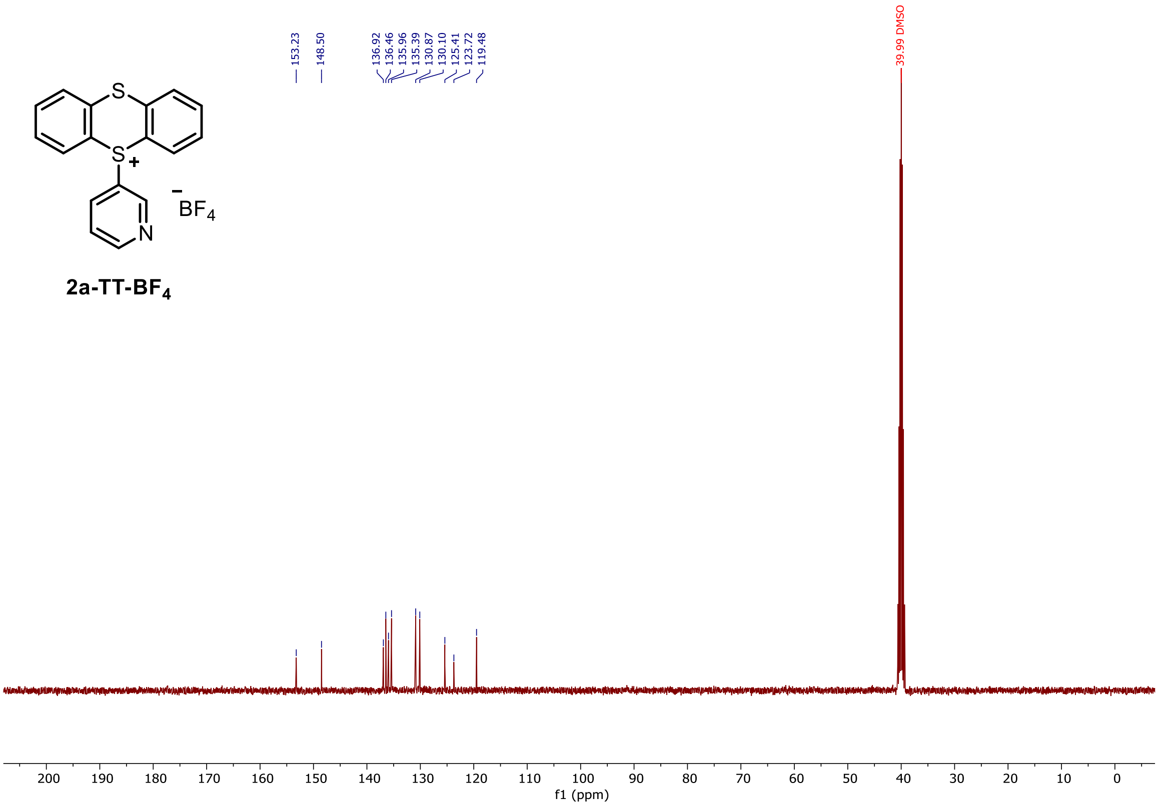

^{19}F NMR (376 MHz, DMSO) of **2a-TT-BF$_4$**

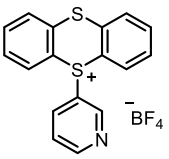

2a-TT-BF$_4$

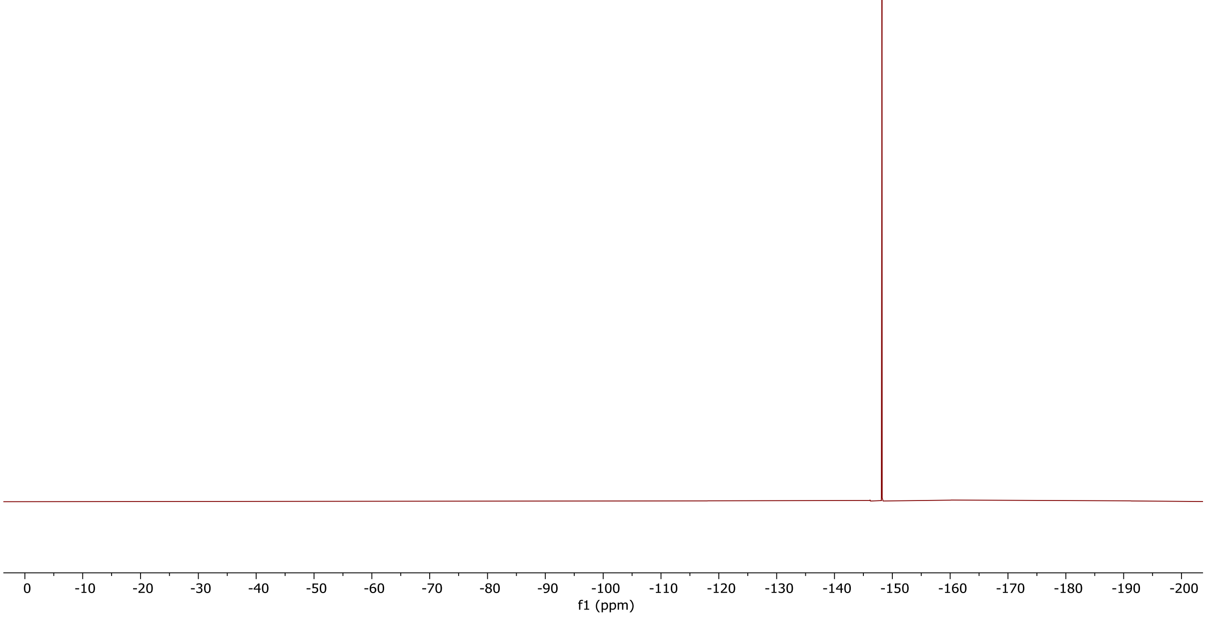

^{1}H NMR (400 MHz, DMSO-d_6) of **2b-DBT-Cl**

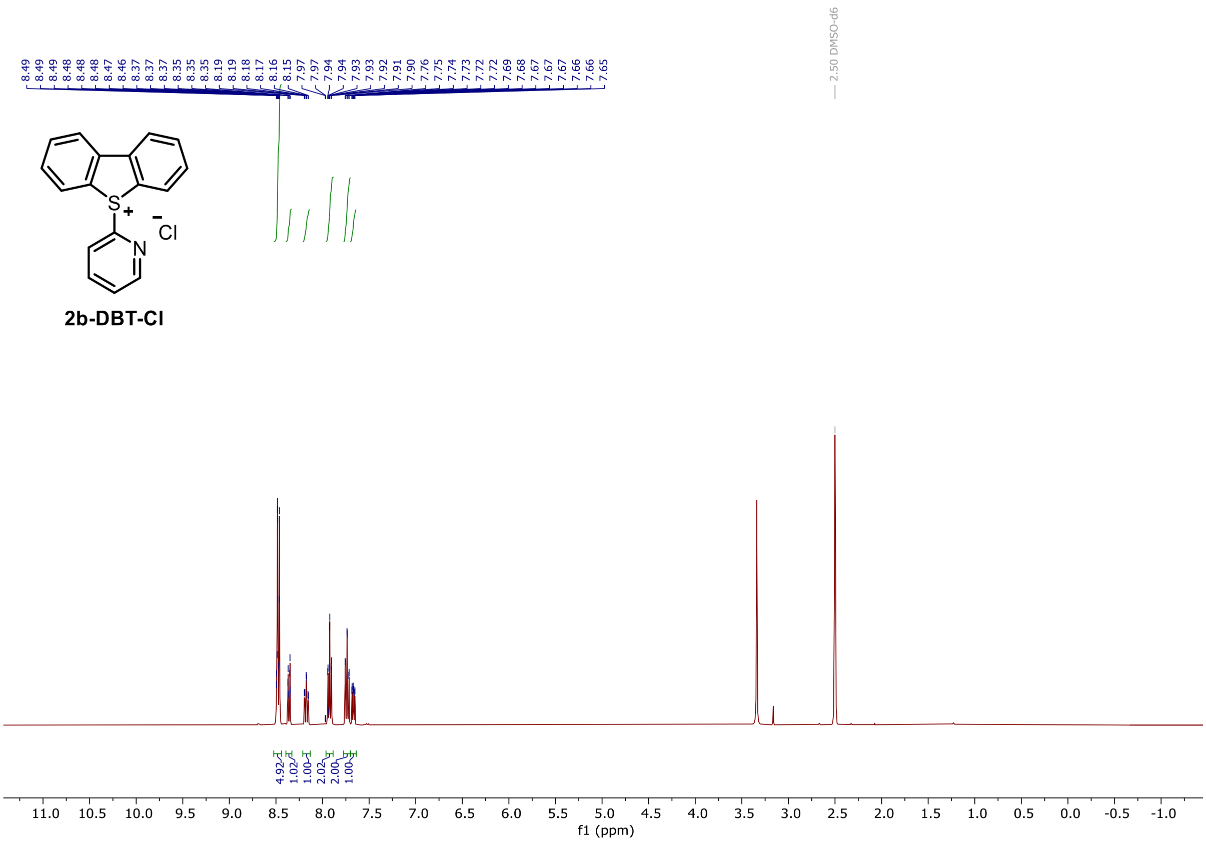

^{13}C NMR (101 MHz, DMSO-d_6) of **2b-DBT-Cl**

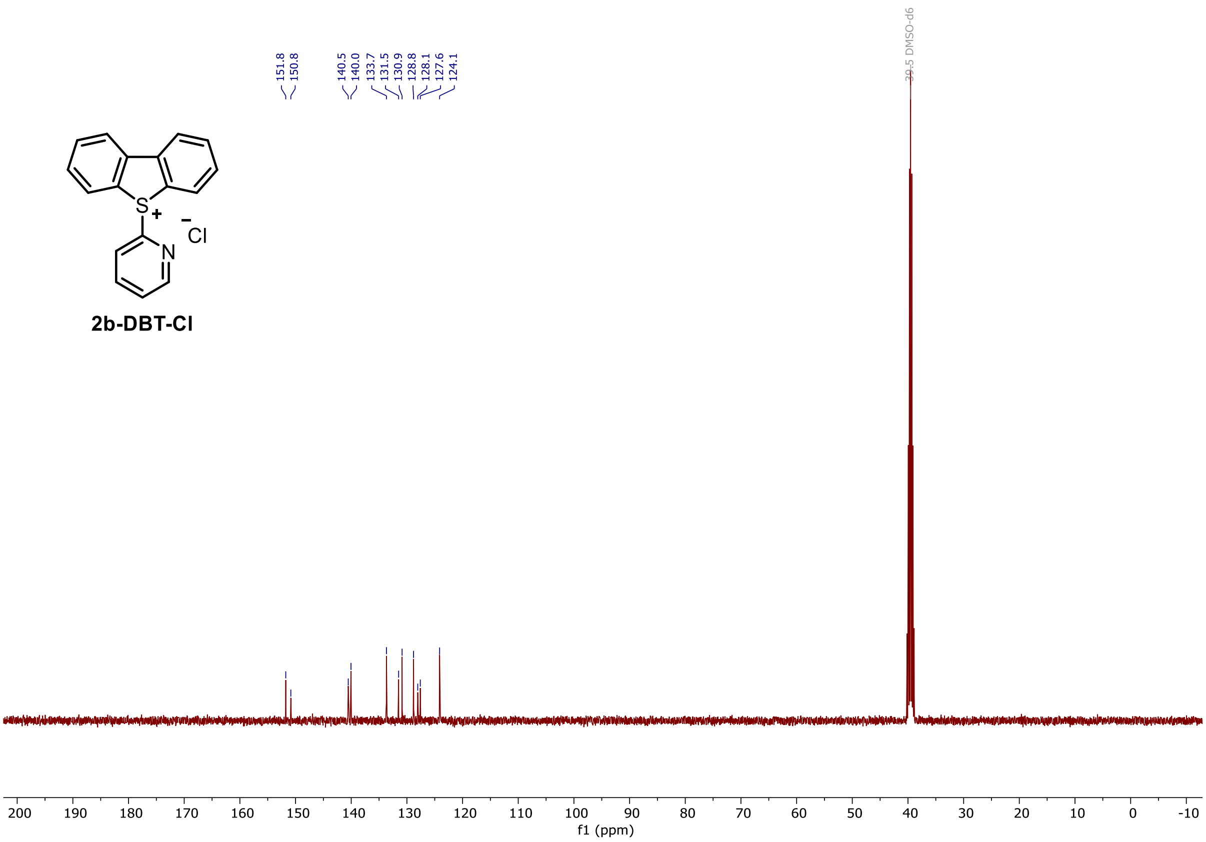

^{1}H NMR (400 MHz, DMSO-d$_6$) of **2b-PXT-Cl**

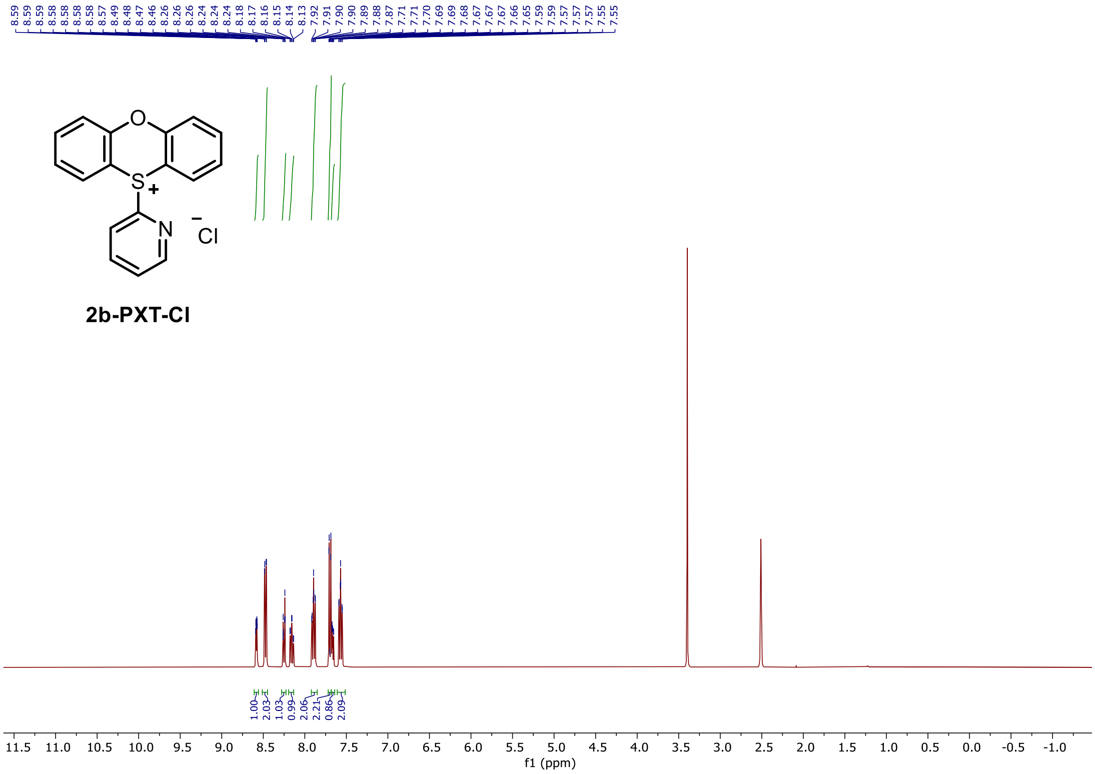

^{13}C NMR (101 MHz, DMSO-d$_6$) of **2b-PXT-Cl**

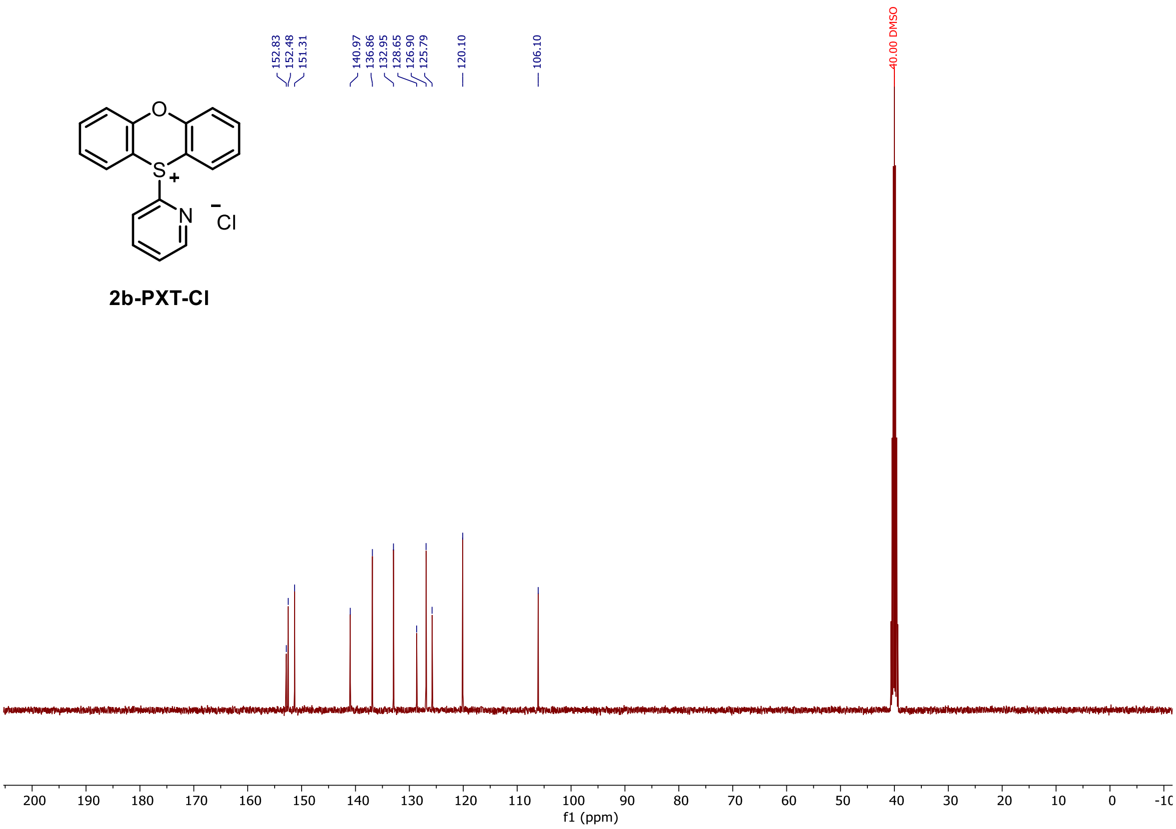

^{1}H NMR (400 MHz, DMSO-d$_6$) of **2c-DBT-Cl**

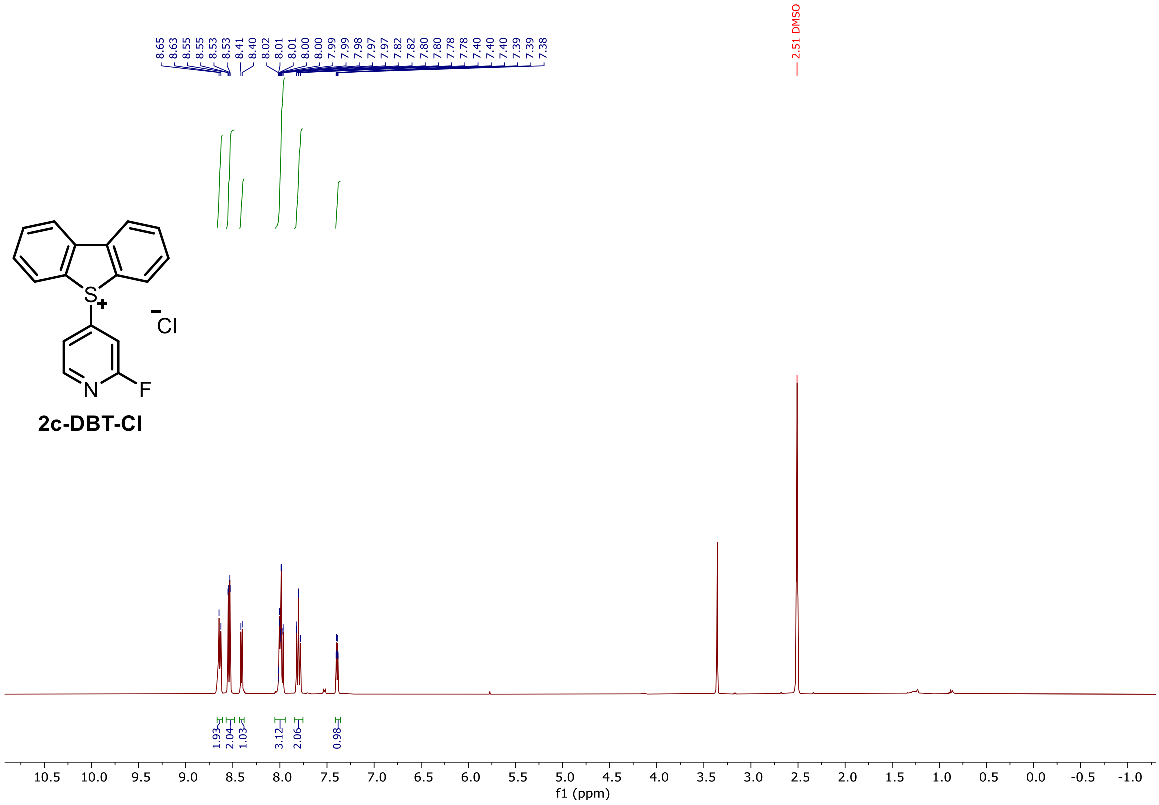

^{13}C NMR (101 MHz, DMSO-d$_6$) of **2c-DBT-Cl**

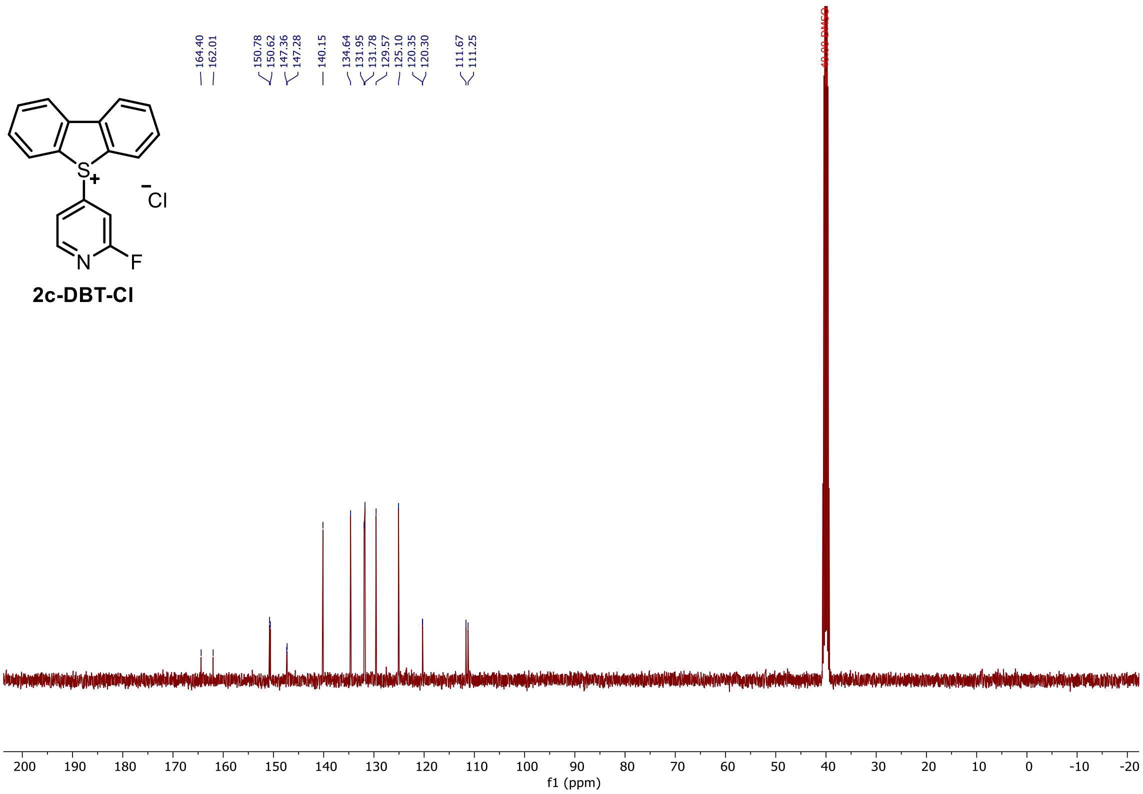

^{19}F NMR (376 MHz, DMSO-d$_6$) of **2c-DBT-Cl**

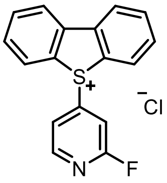

2c-DBT-Cl

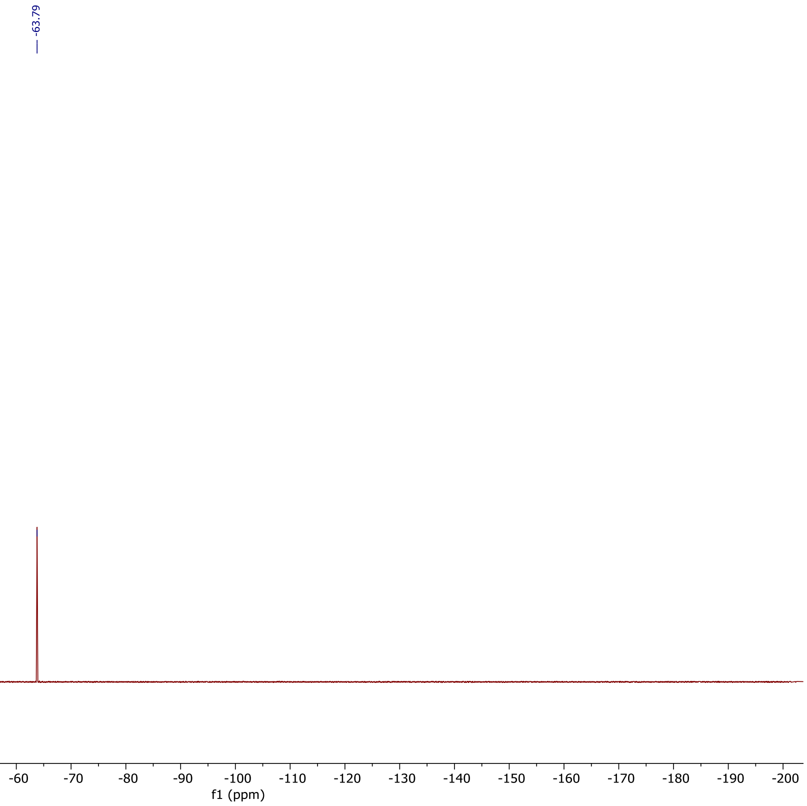

^{1}H NMR (400 MHz, DMSO-d$_6$) of **2d-DBT-Cl**

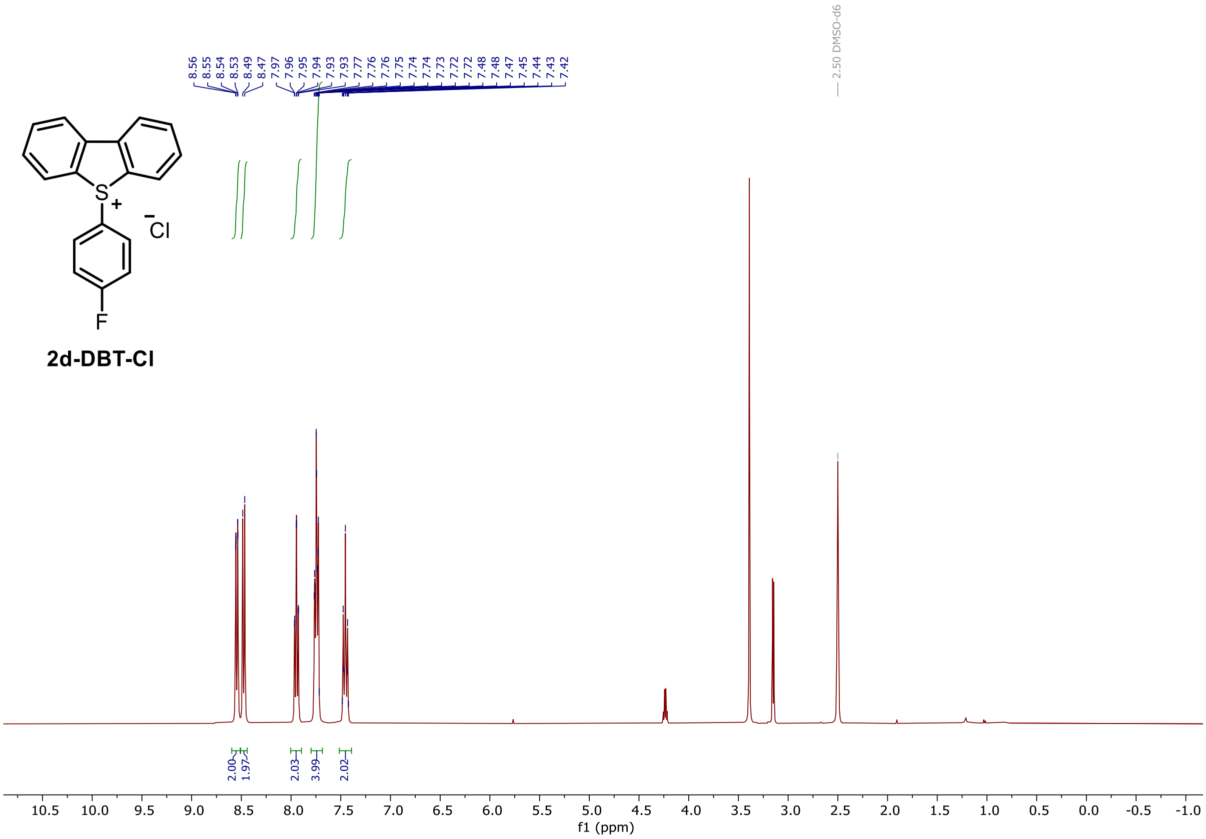

^{13}C NMR (101 MHz, DMSO-d$_6$) of **2d-DBT-Cl**

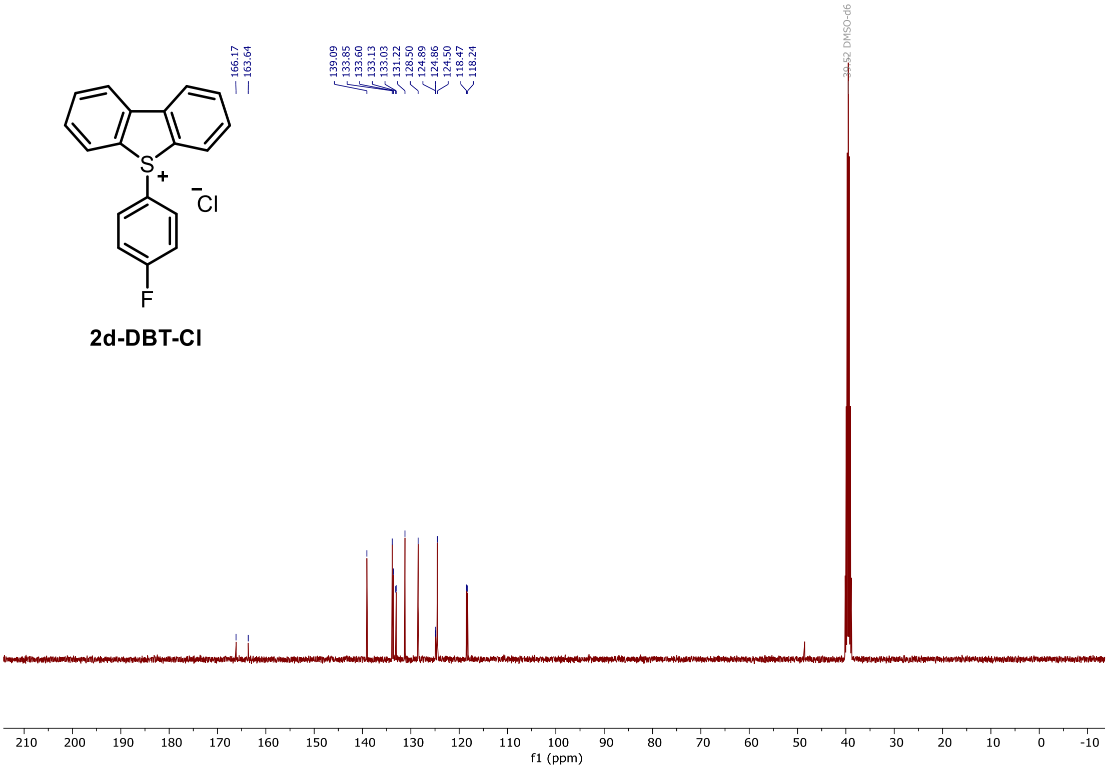

^{19}F NMR (376 MHz, DMSO-d$_6$) of **2d-DBT-Cl**

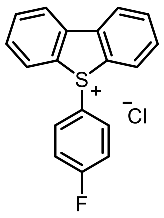

2d-DBT-Cl

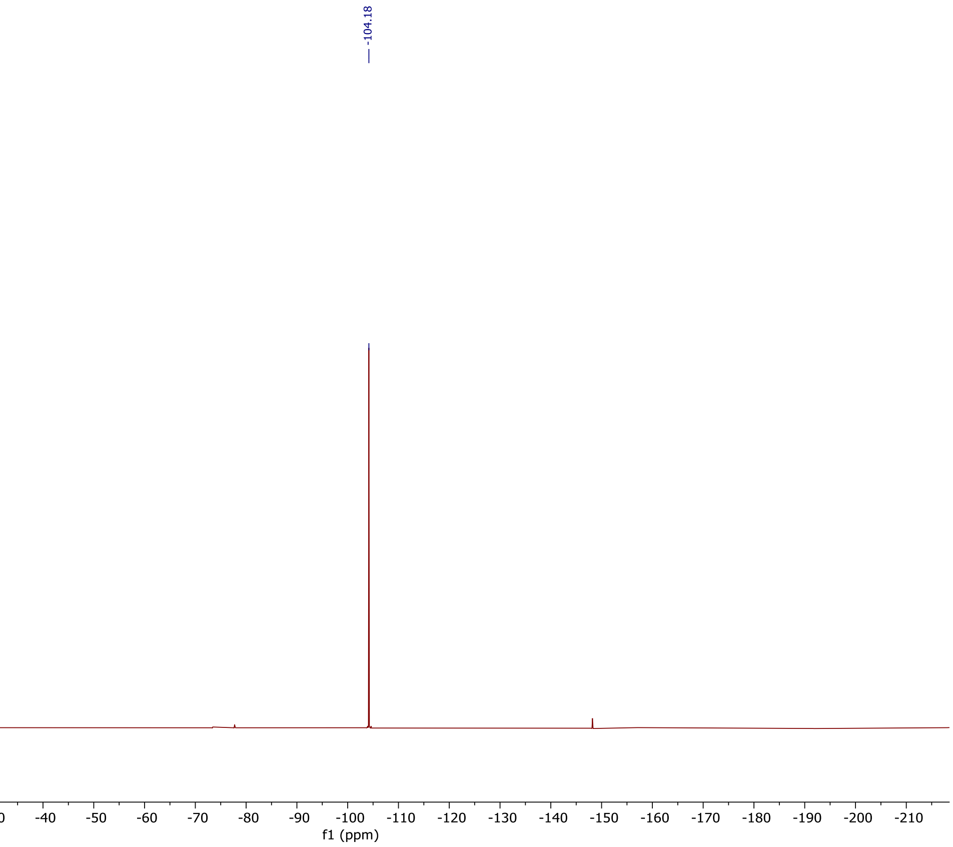

^{1}H NMR (400 MHz, DMSO-d6) of **2e-DBT-Cl**

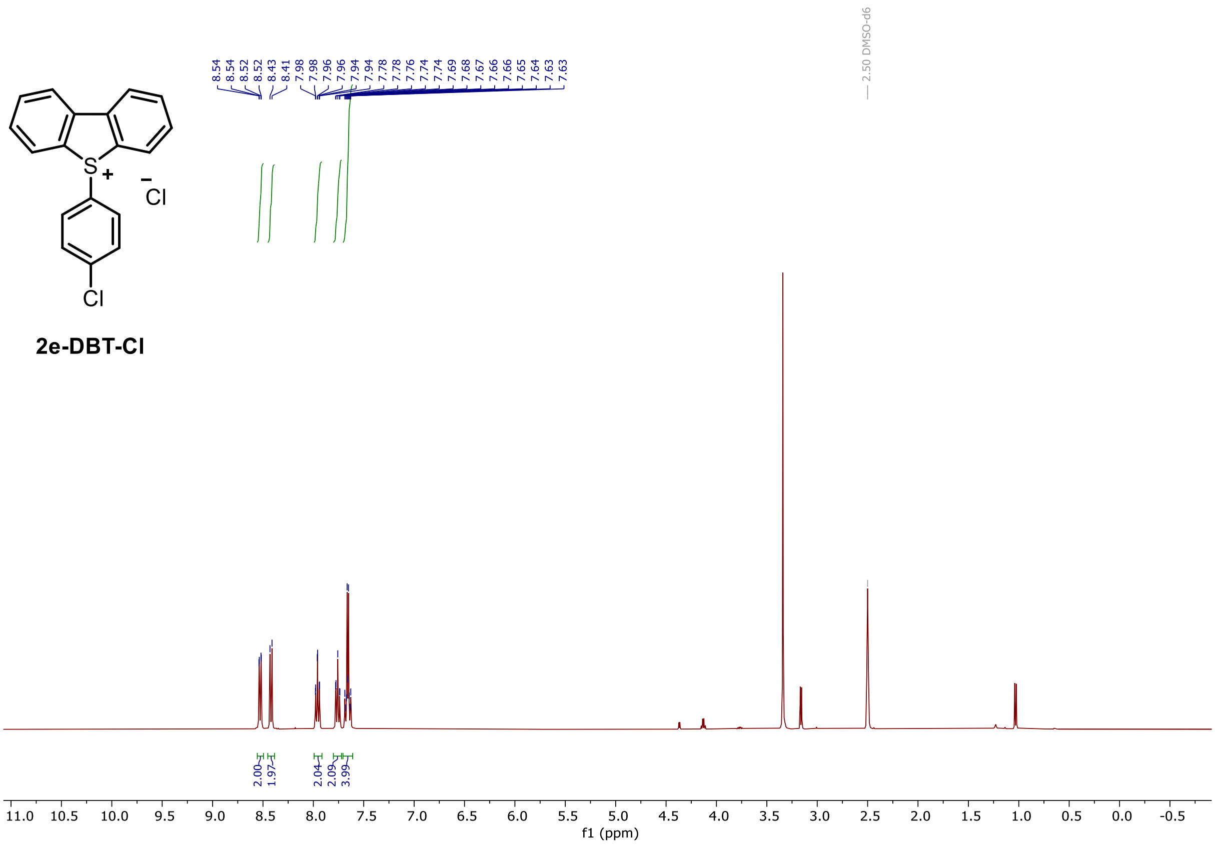

^{13}C NMR (101 MHz, DMSO-d6) of **2e-DBT-Cl**

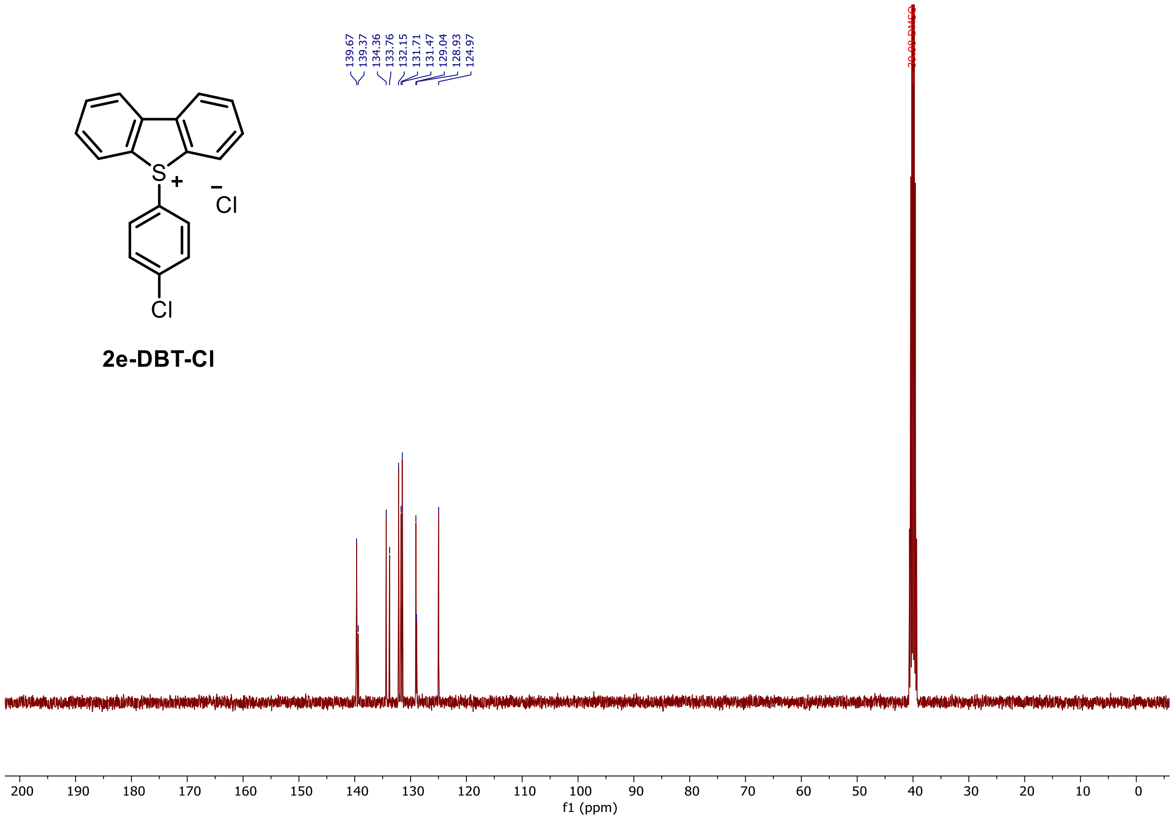

^{1}H NMR (500 MHz, DMSO-d_6) of **2f-DBT-Cl**

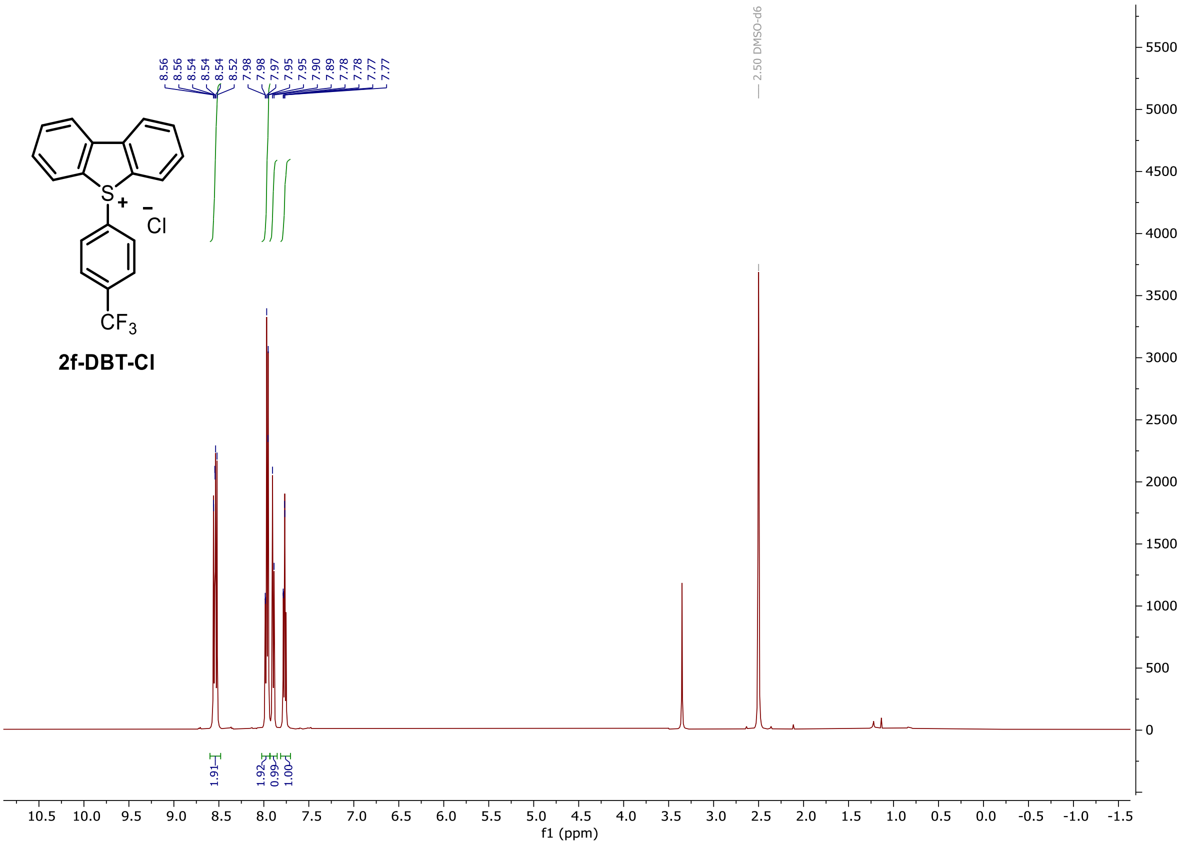

^{13}C NMR (126 MHz, DMSO-d_6) of **2f-DBT-Cl**

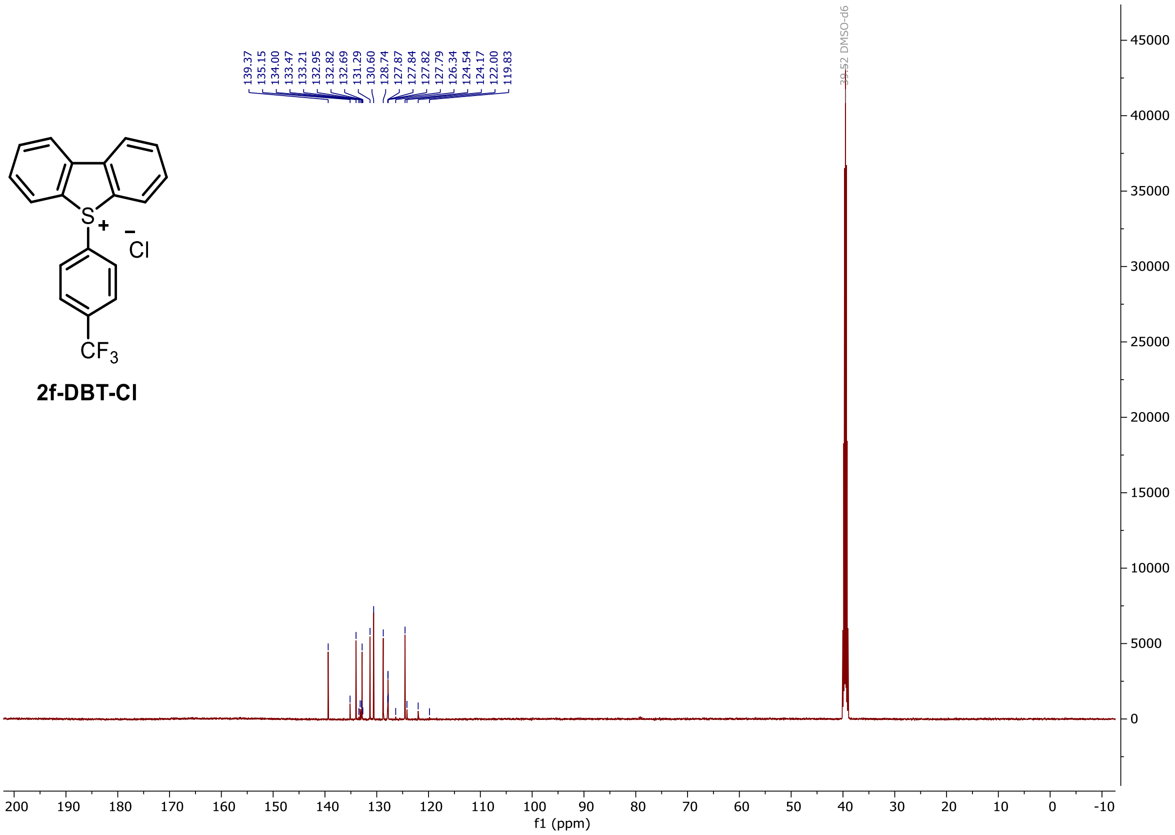

^{19}F NMR (471 MHz, DMSO-d_6) of **2f-DBT-Cl**

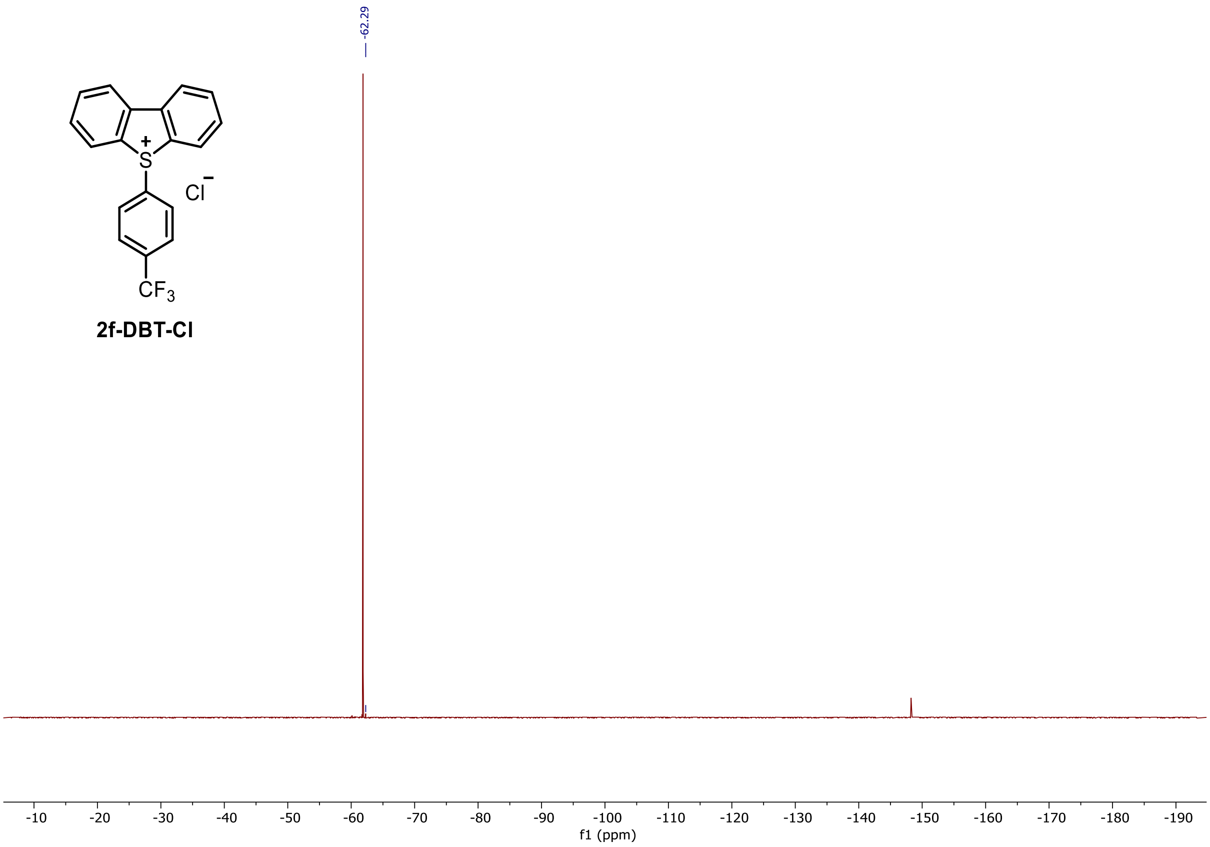

^{1}H NMR (400 MHz, DMSO-d$_6$) of **2g-DBT-Cl**

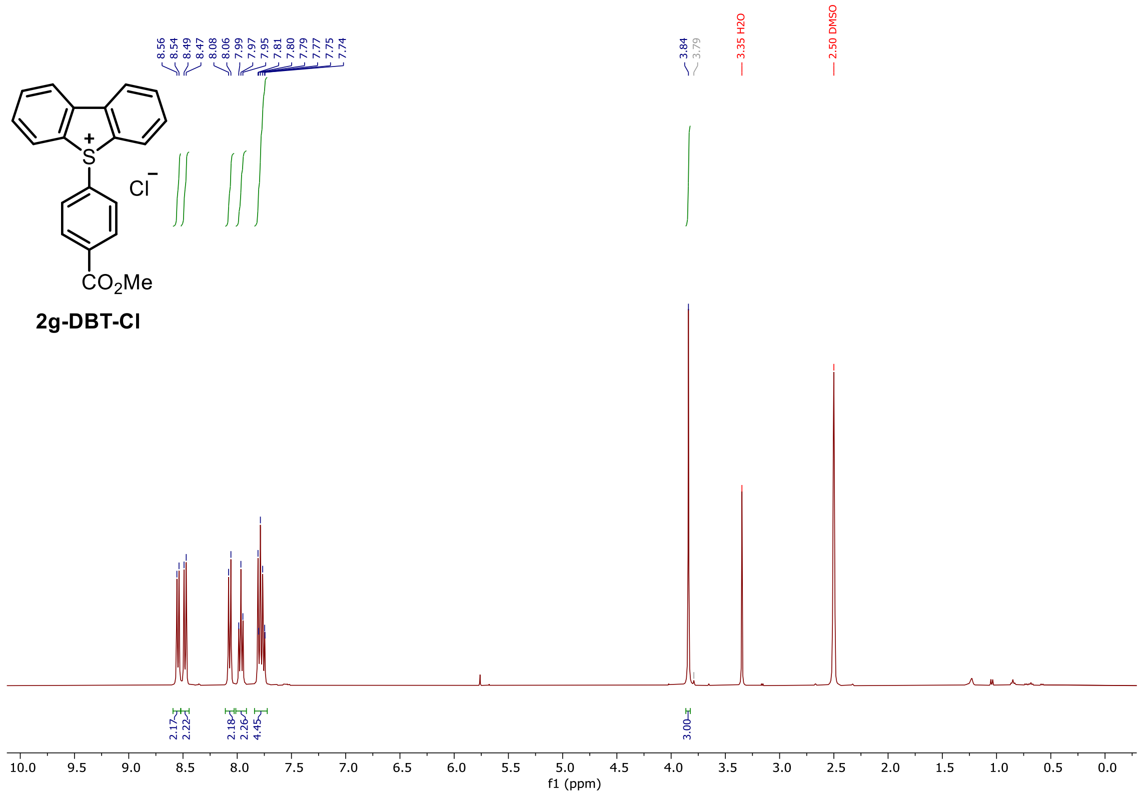

^{13}C NMR (101 MHz, DMSO-d$_6$) of **2g-DBT-Cl**

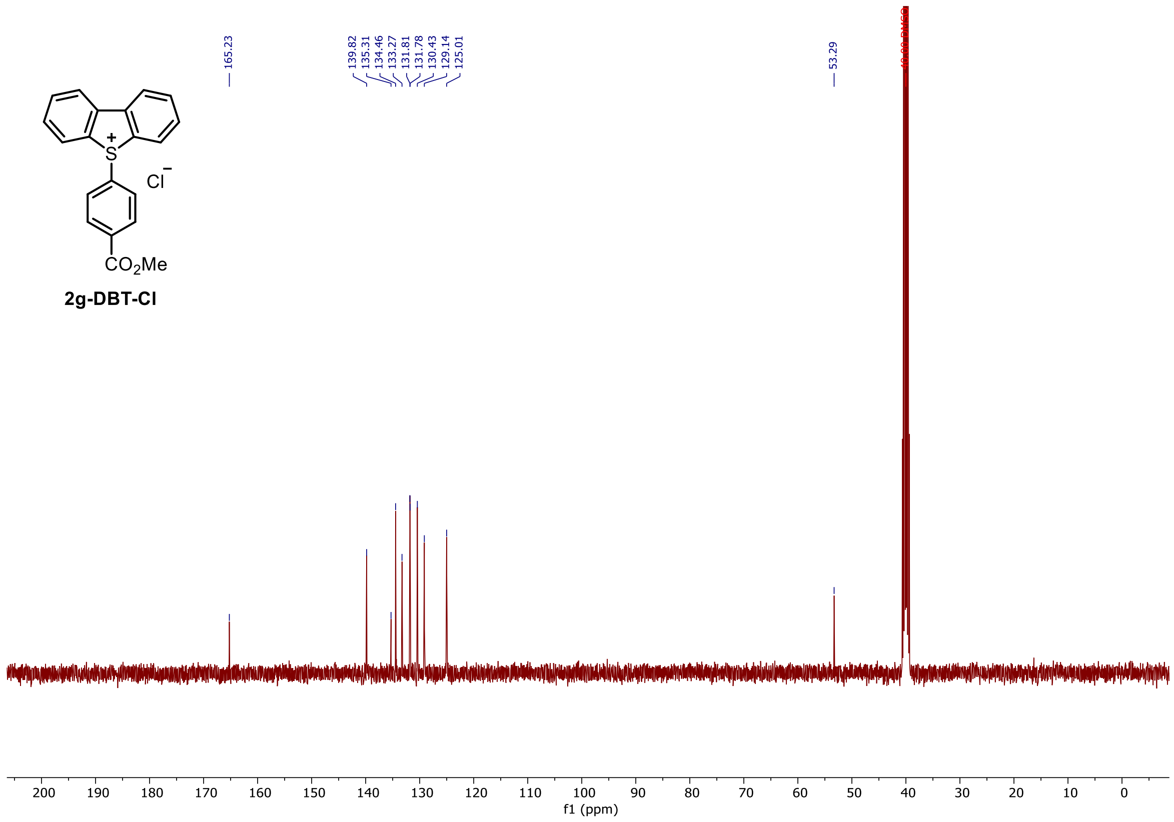

^{1}H NMR (500 MHz, DMSO-d6) of **2h-DBT-Cl**

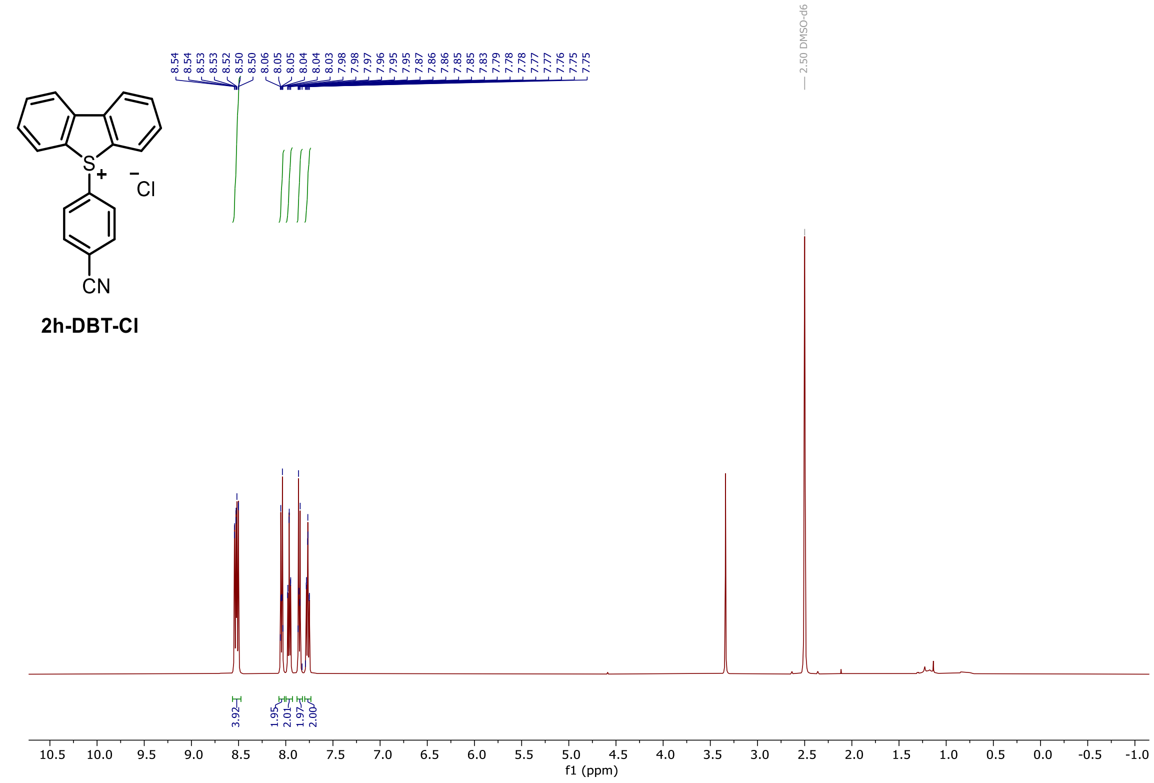

^{13}C NMR (126 MHz, DMSO-d6) of **2h-DBT-Cl**

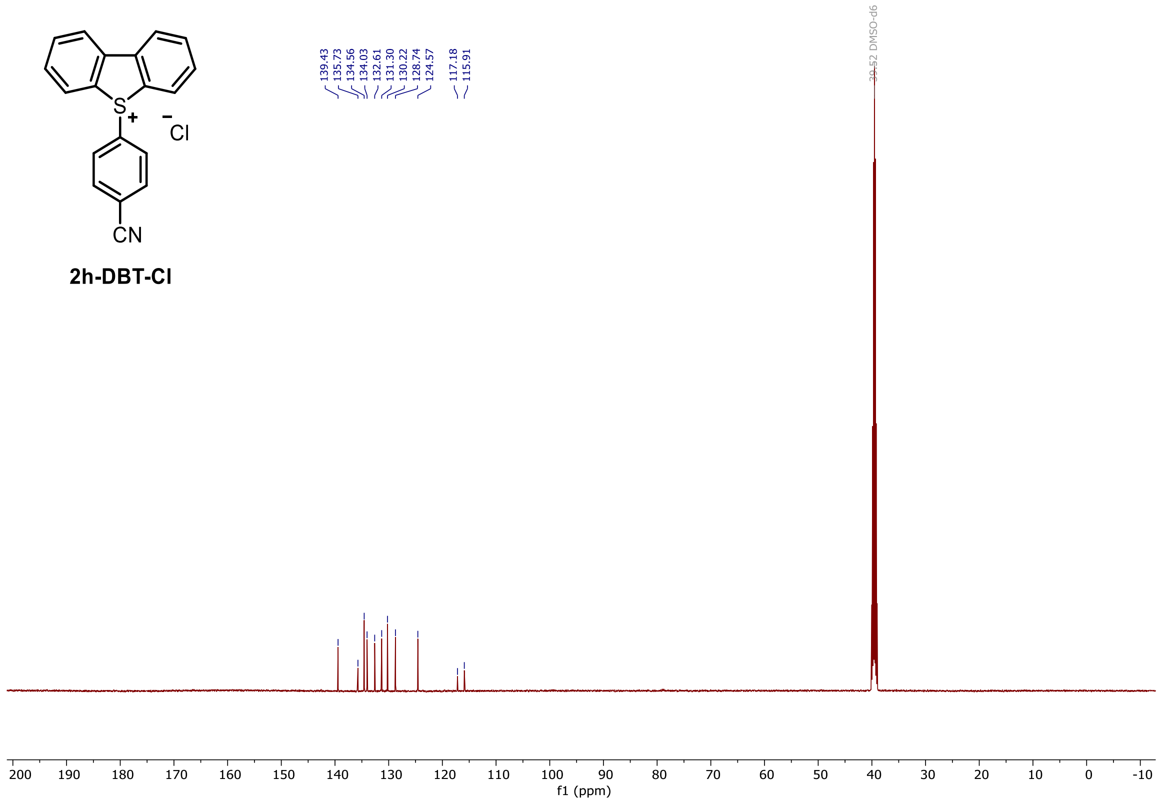

^{1}H NMR (400 MHz, DMSO-d$_6$) of **2i-DBT-Cl**

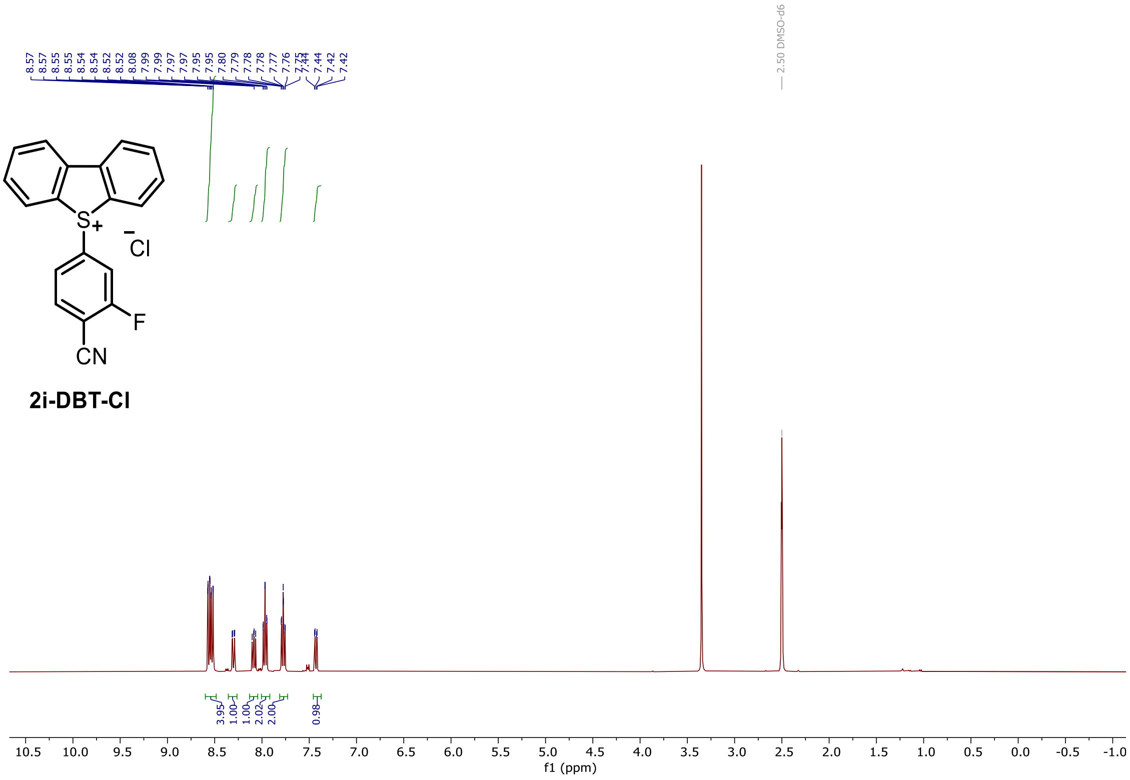

^{13}C NMR (101 MHz, DMSO-d$_6$) of **2i-DBT-Cl**

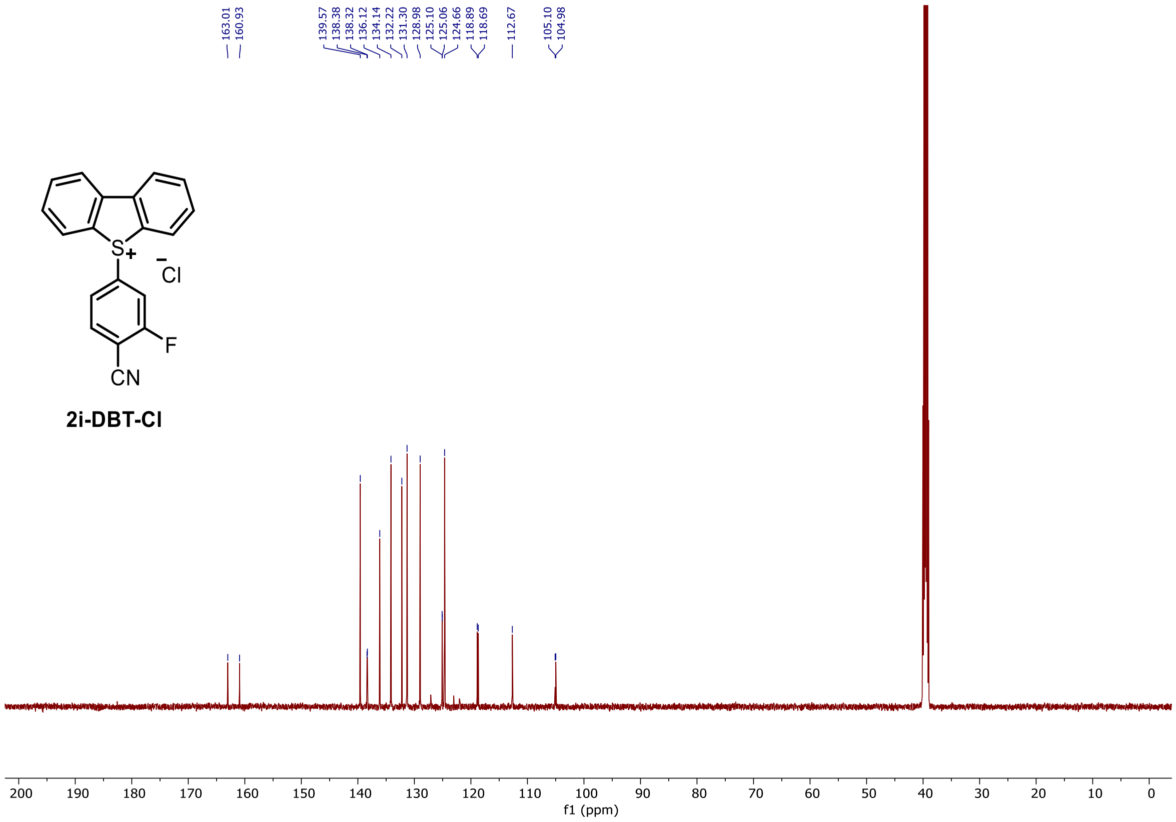

^{19}F NMR (376 MHz, DMSO-d$_6$) of **2i-DBT-Cl**

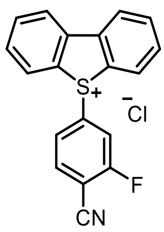

2i-DBT-Cl

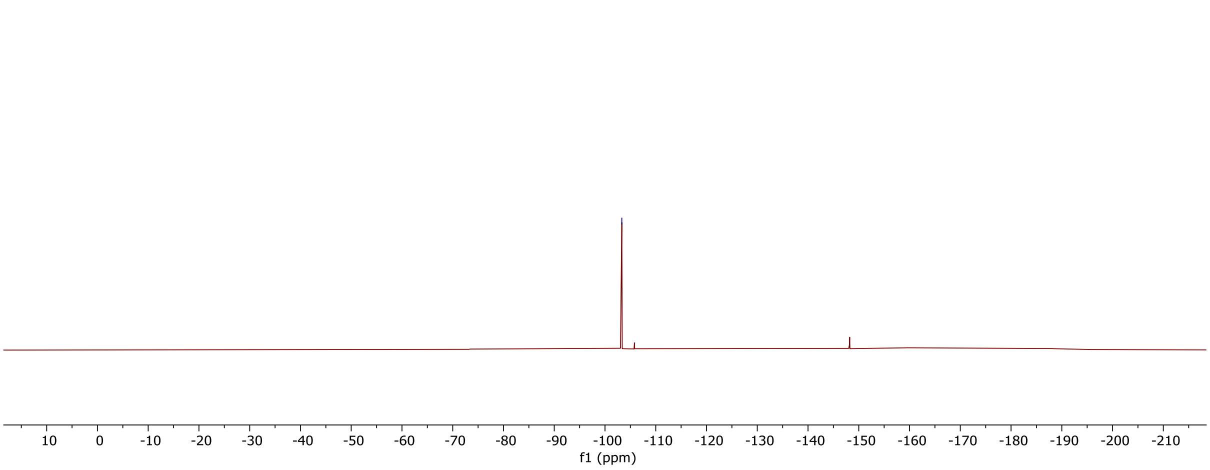

^{1}H NMR (400 MHz, DMSO-d$_6$) of **2j-DBT-Cl**

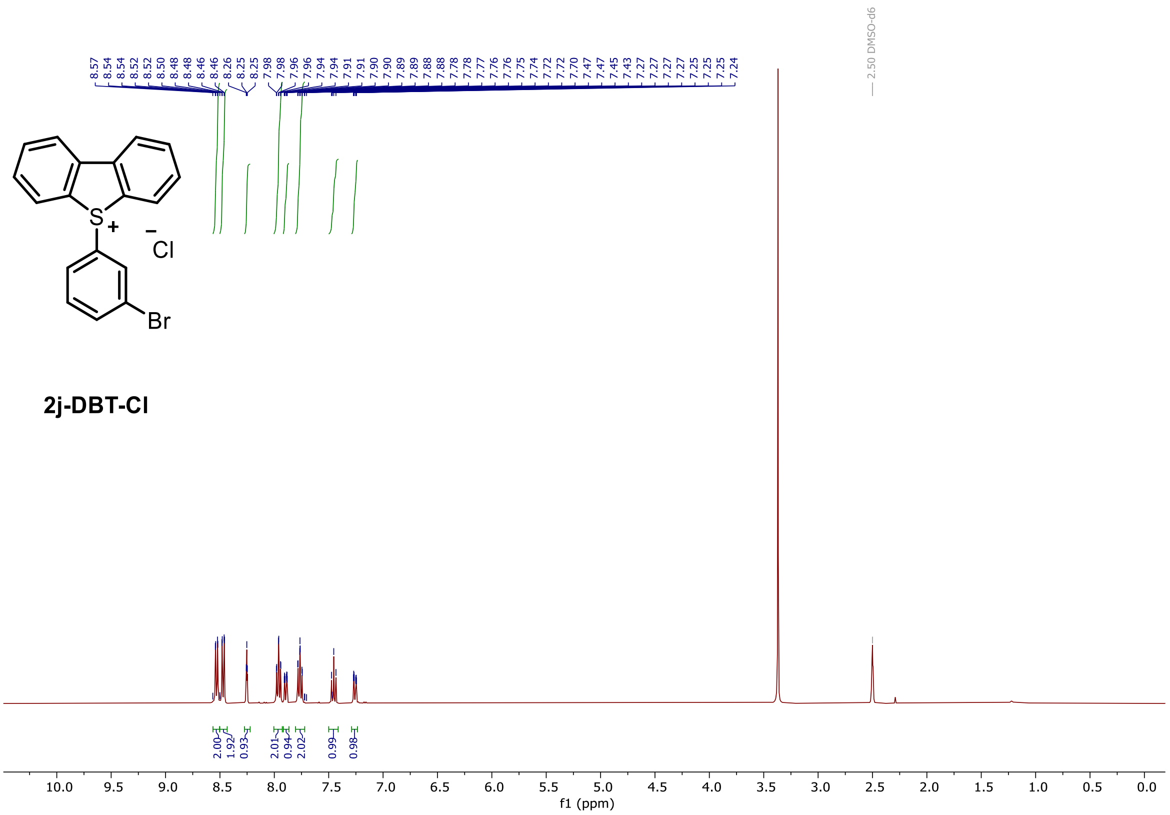

^{13}C NMR (101 MHz, DMSO-d$_6$) of **2j-DBT-Cl**

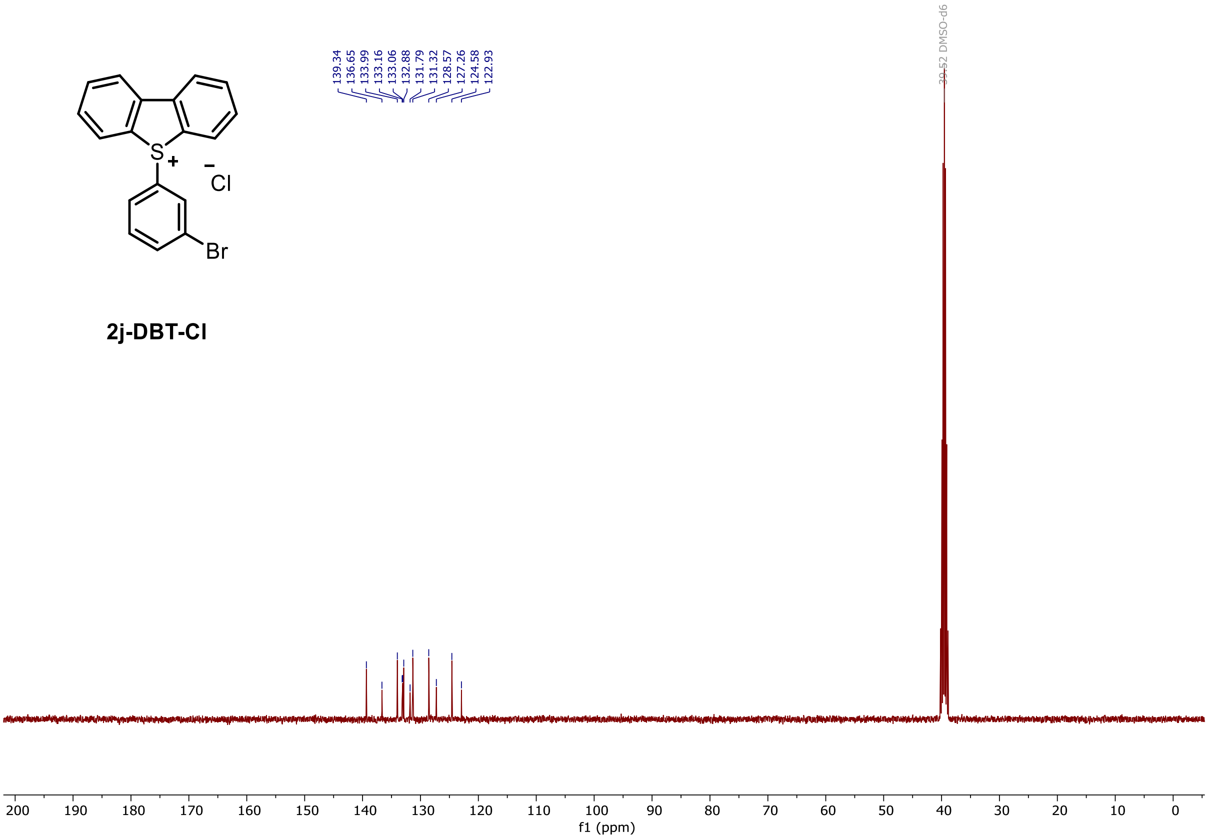

^{1}H NMR (400 MHz, DMSO-d$_6$) of **2j-PXT-Cl**

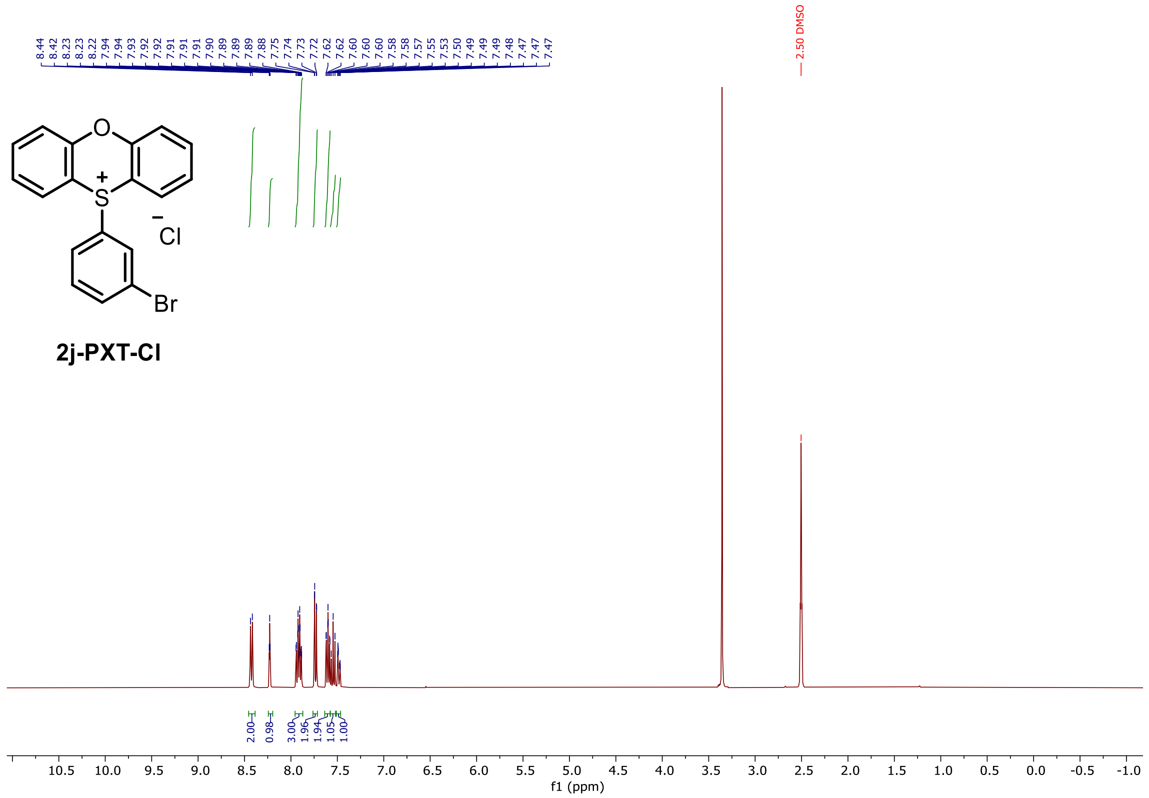

^{13}C NMR (101 MHz, DMSO-d$_6$) of **2j-PXT-Cl**

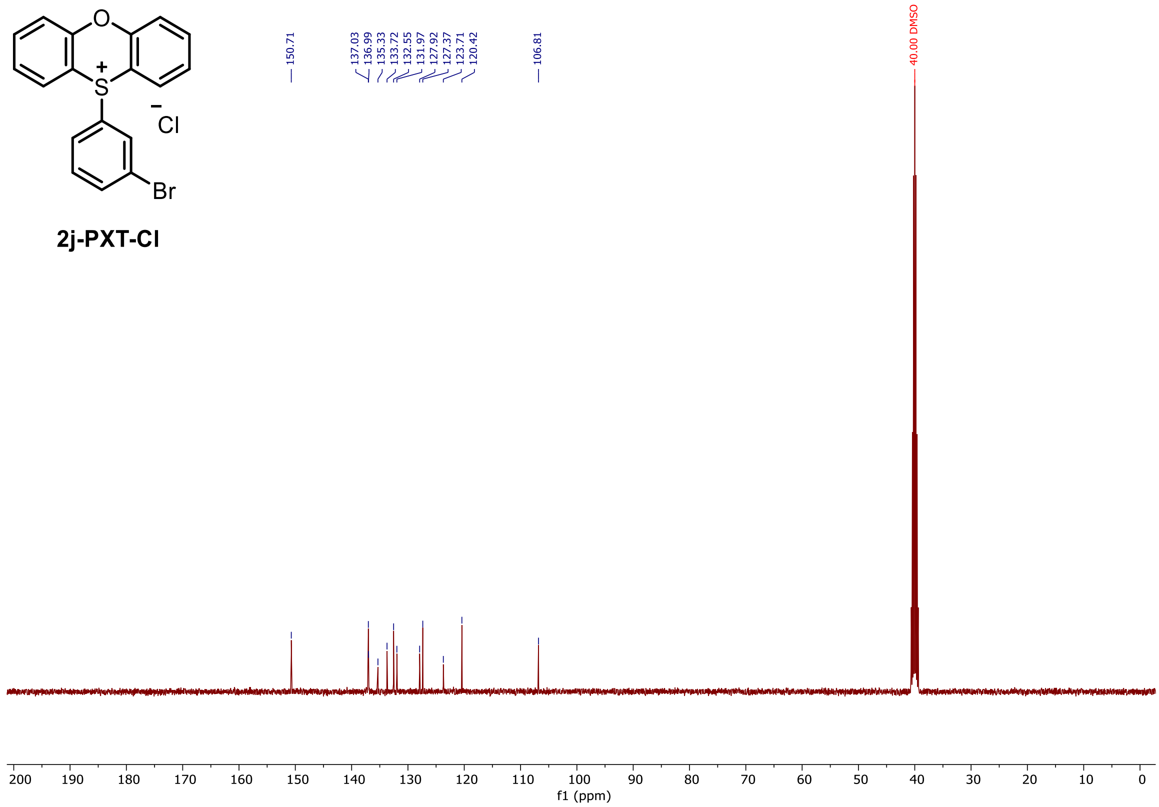

^1H NMR (500 MHz, DMSO-d_6) of **2k-DBT-Cl**

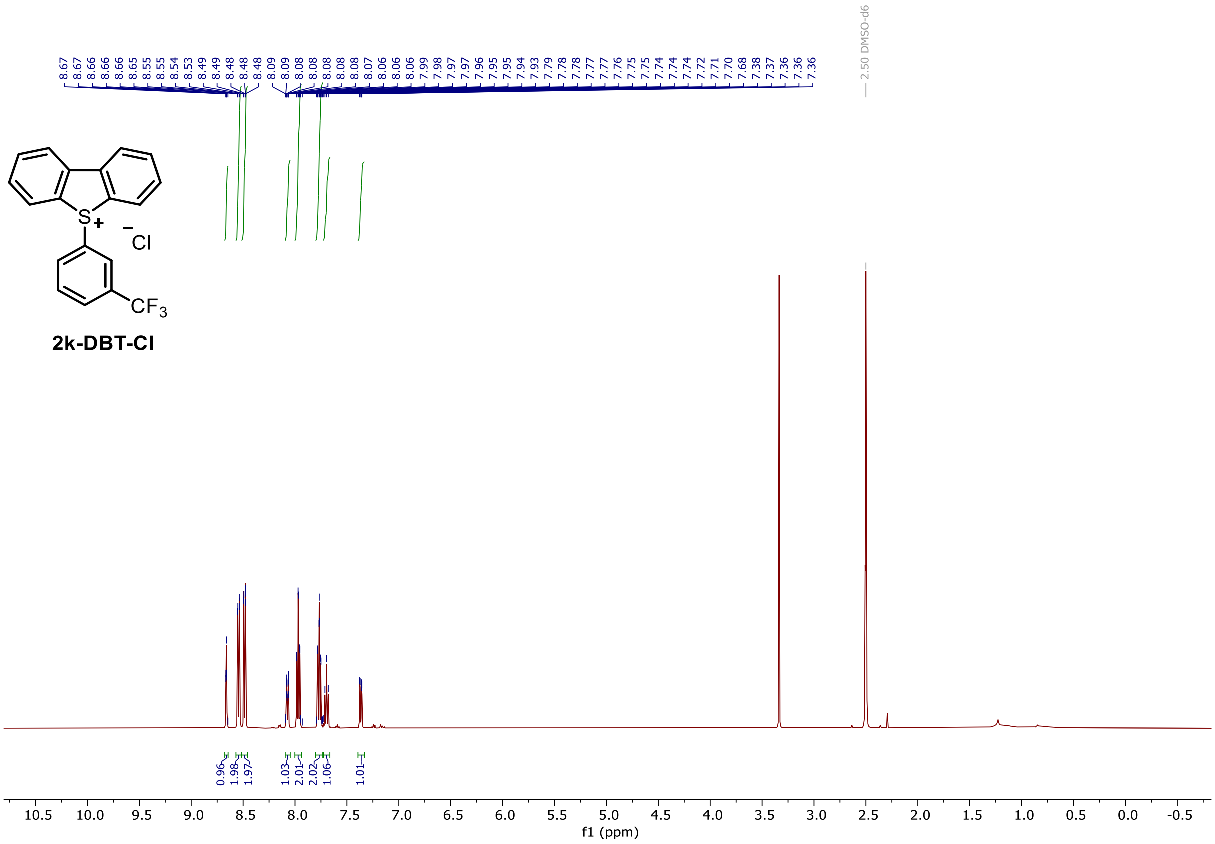

^{13}C NMR (126 MHz, DMSO-d_6) of **2k-DBT-Cl**

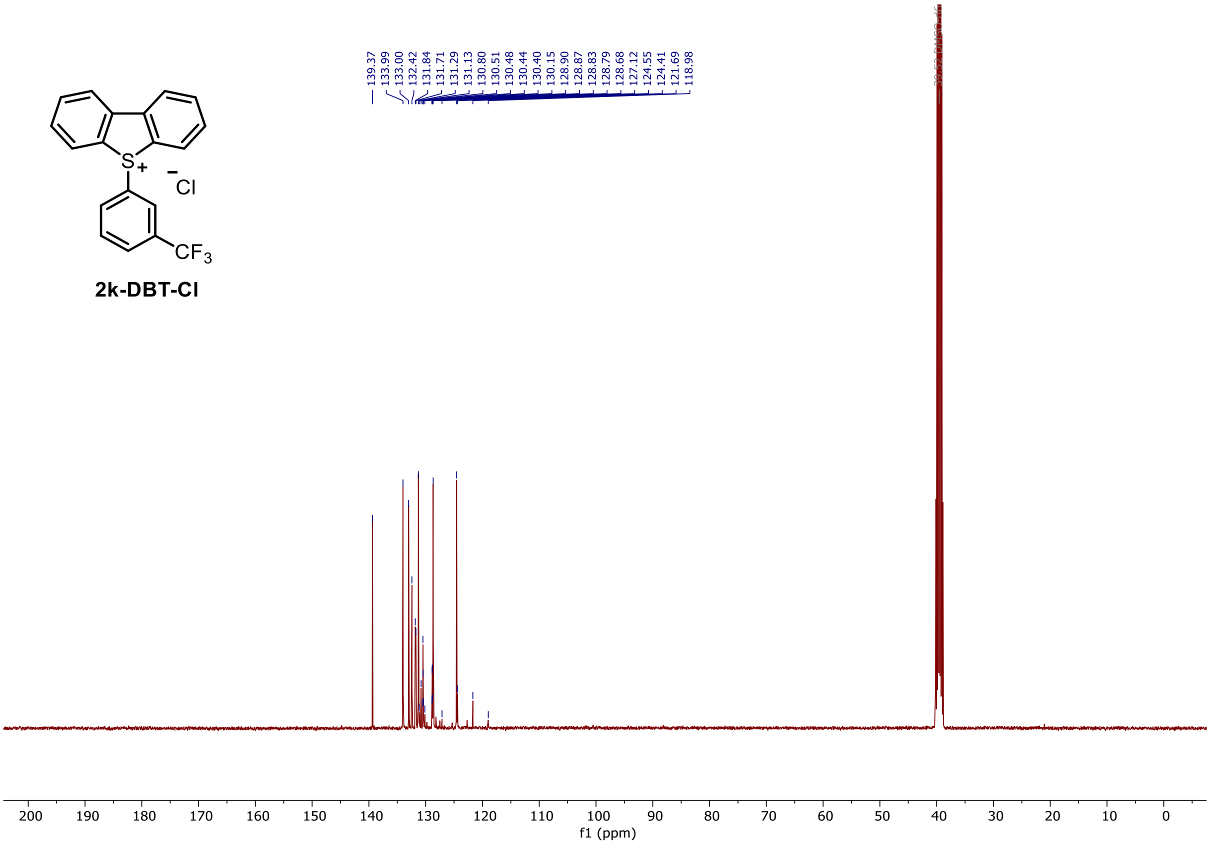

^{19}F (376 MHz, DMSO-d_6) of **2k-DBT-Cl**

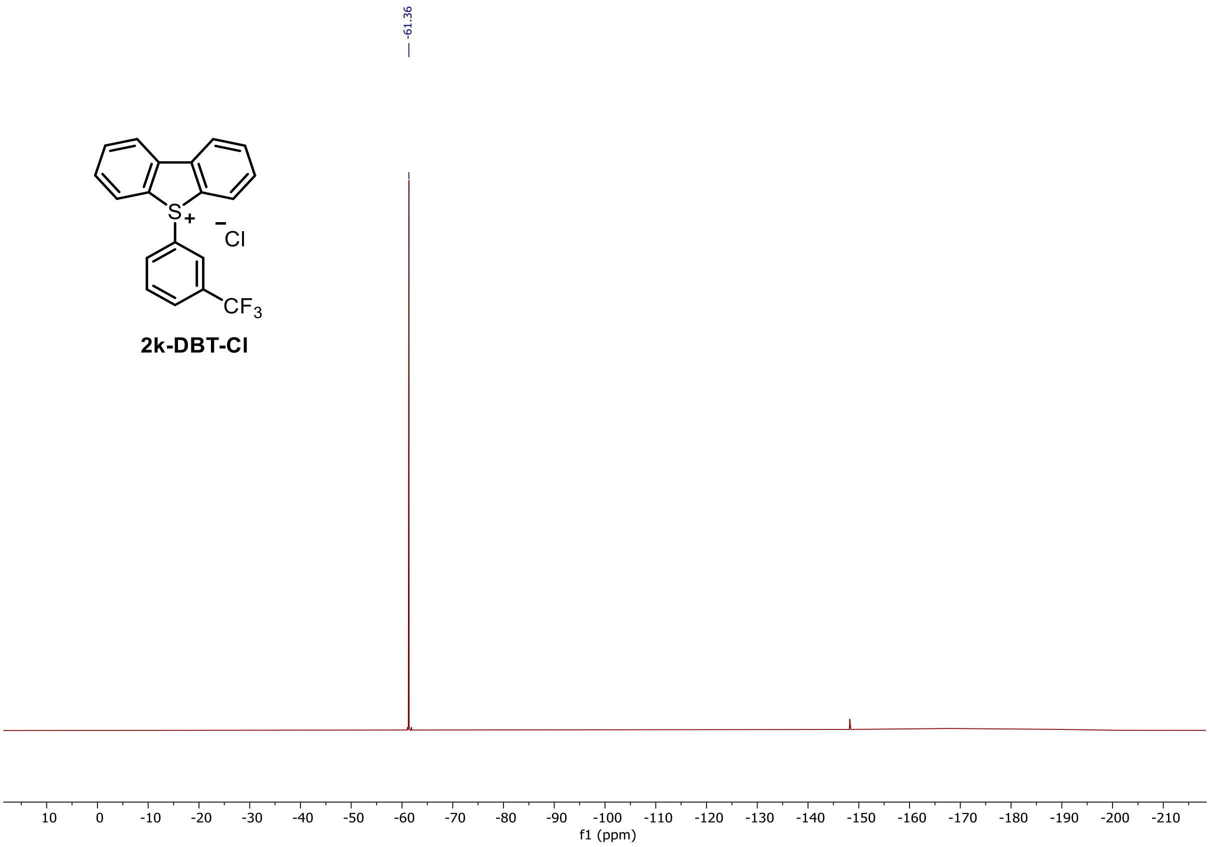

^{1}H NMR (400 MHz, DMSO-d$_6$) of **2l-DBT-Cl**

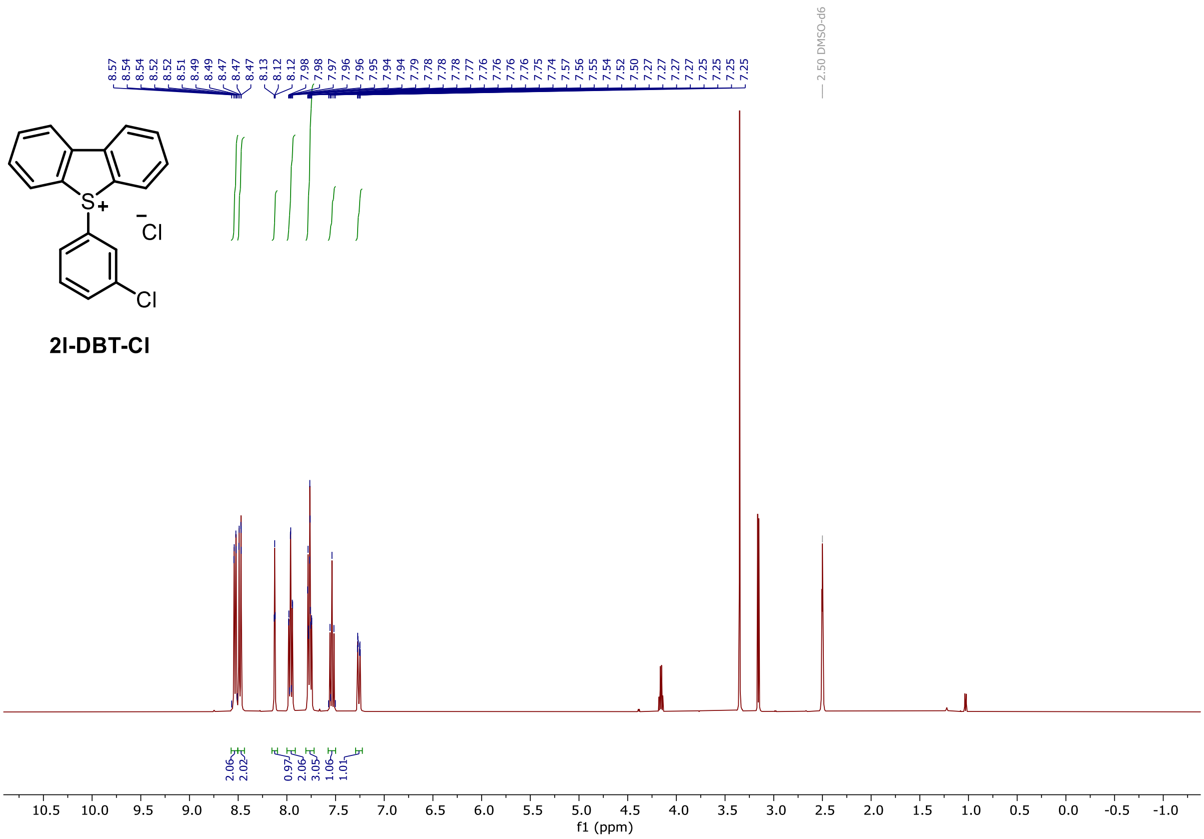

^{13}C NMR (101 MHz, DMSO-d$_6$) of **2l-DBT-Cl**

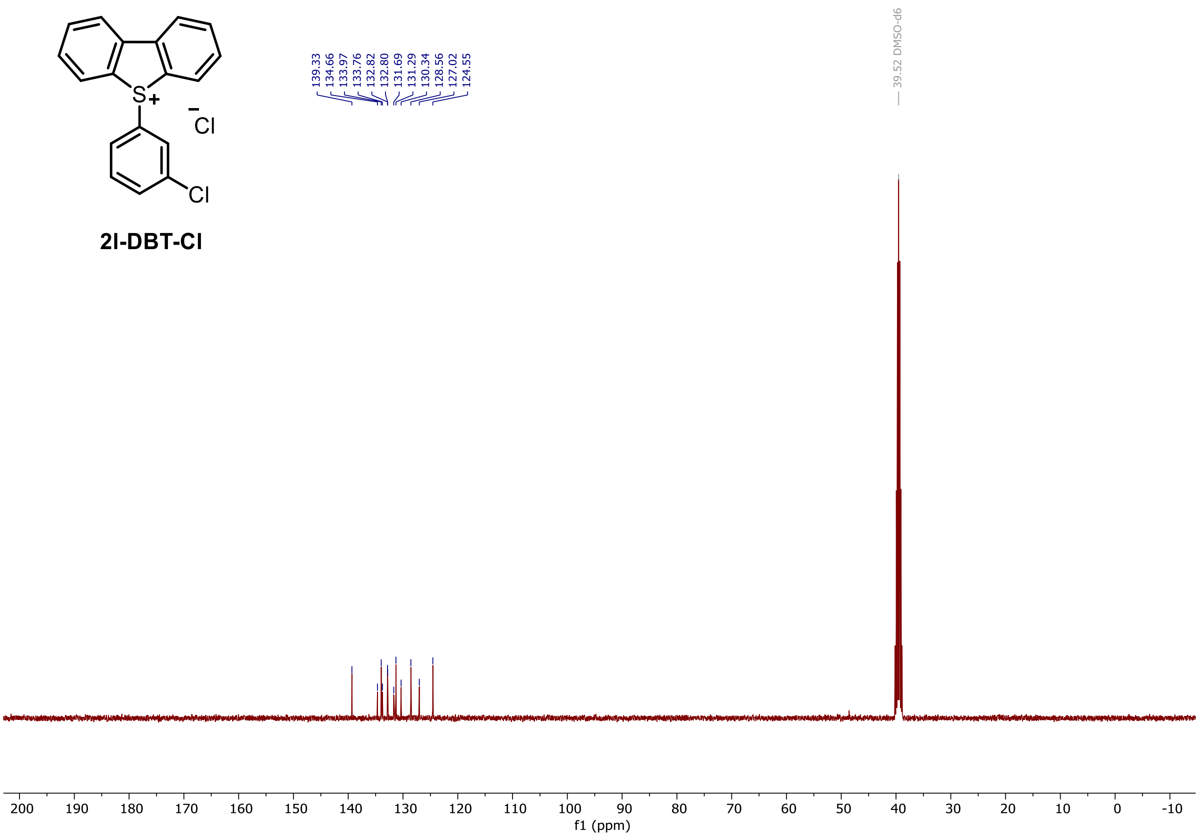

^{1}H NMR (500 MHz, DMSO-d$_6$) of **2m-DBT-Cl**

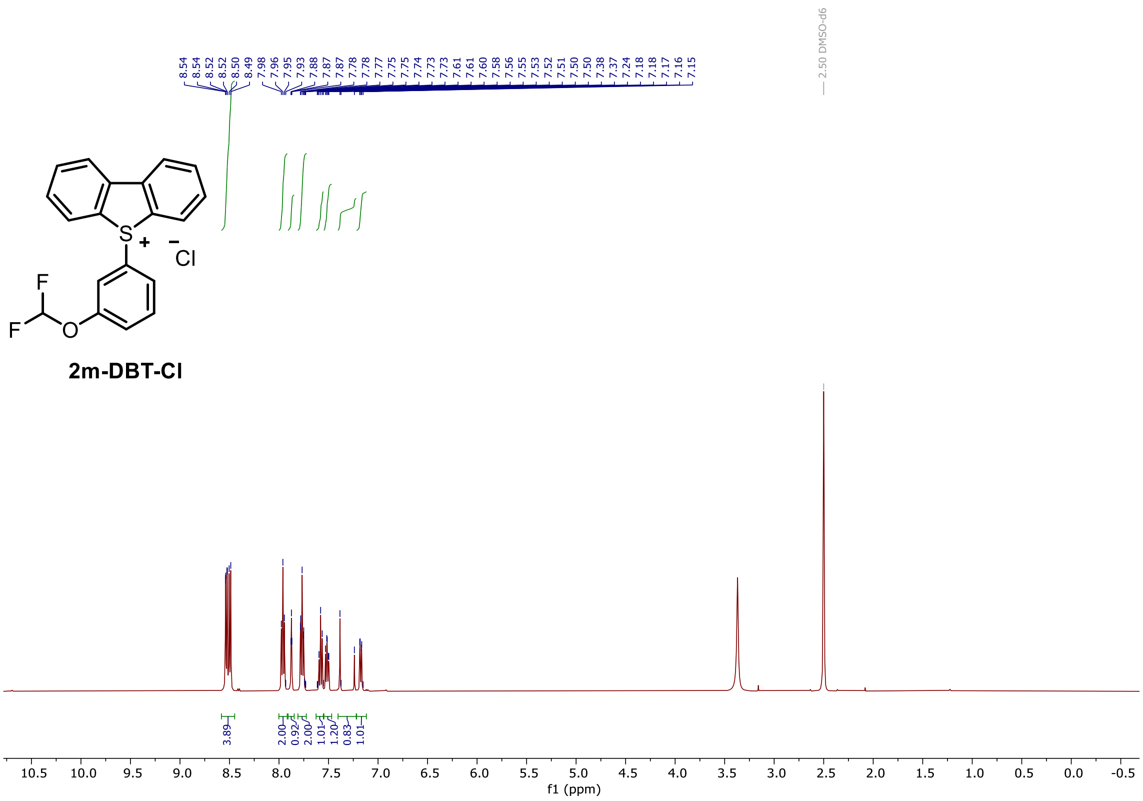

^{13}C NMR (126 MHz, DMSO-d$_6$) of **2m-DBT-Cl**

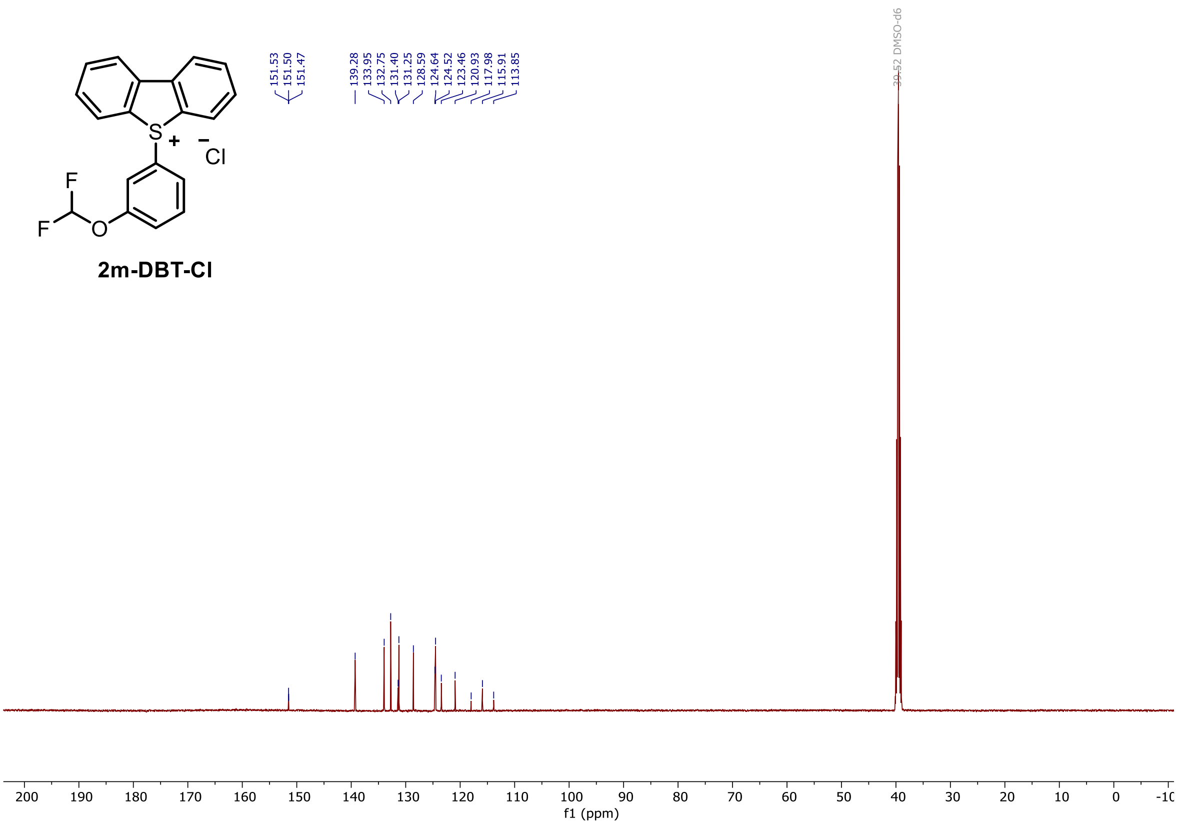

^{19}F NMR (376 MHz, DMSO-d_6) of **2m-DBT-Cl**

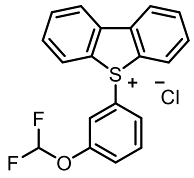

2m-DBT-Cl

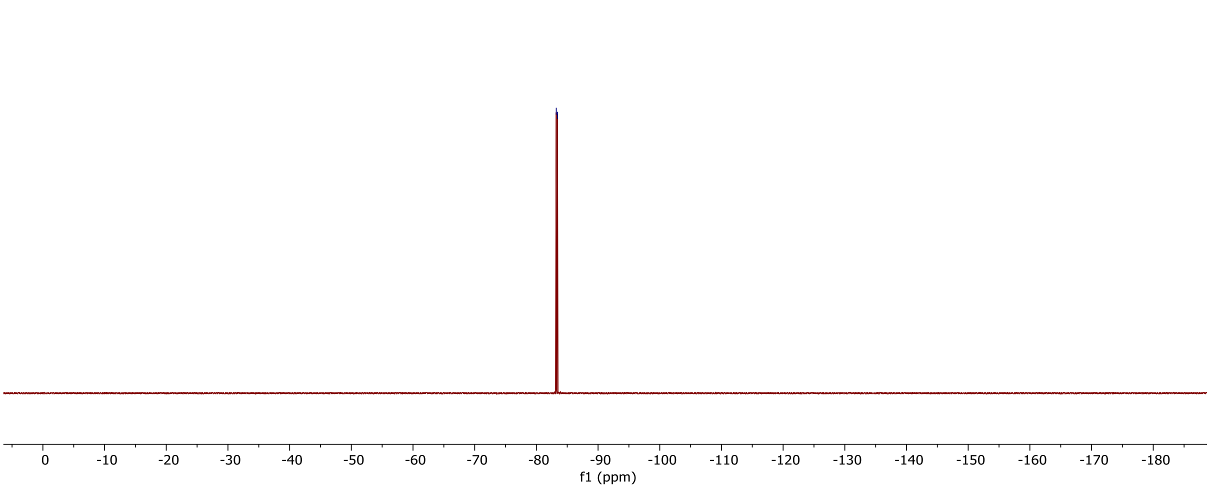

^{1}H NMR (400 MHz, DMSO-d$_6$) of **2n-DBT-Cl**

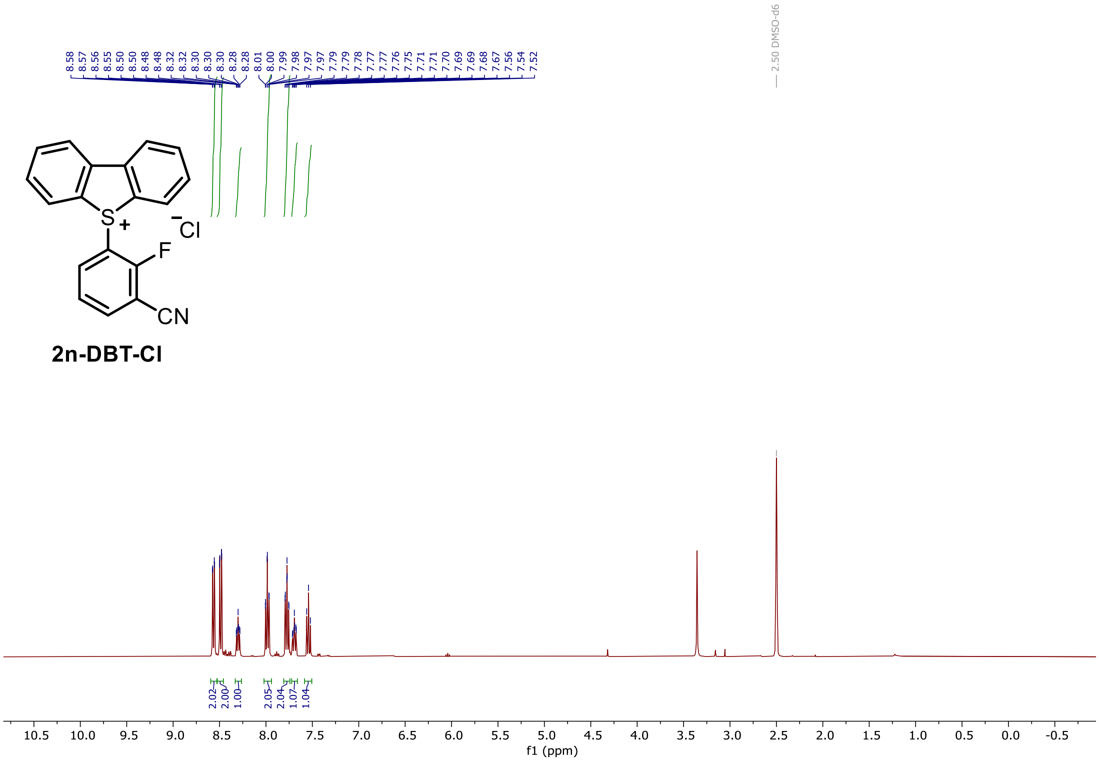

^{13}C NMR (101 MHz, DMSO-d$_6$) of **2n-DBT-Cl**

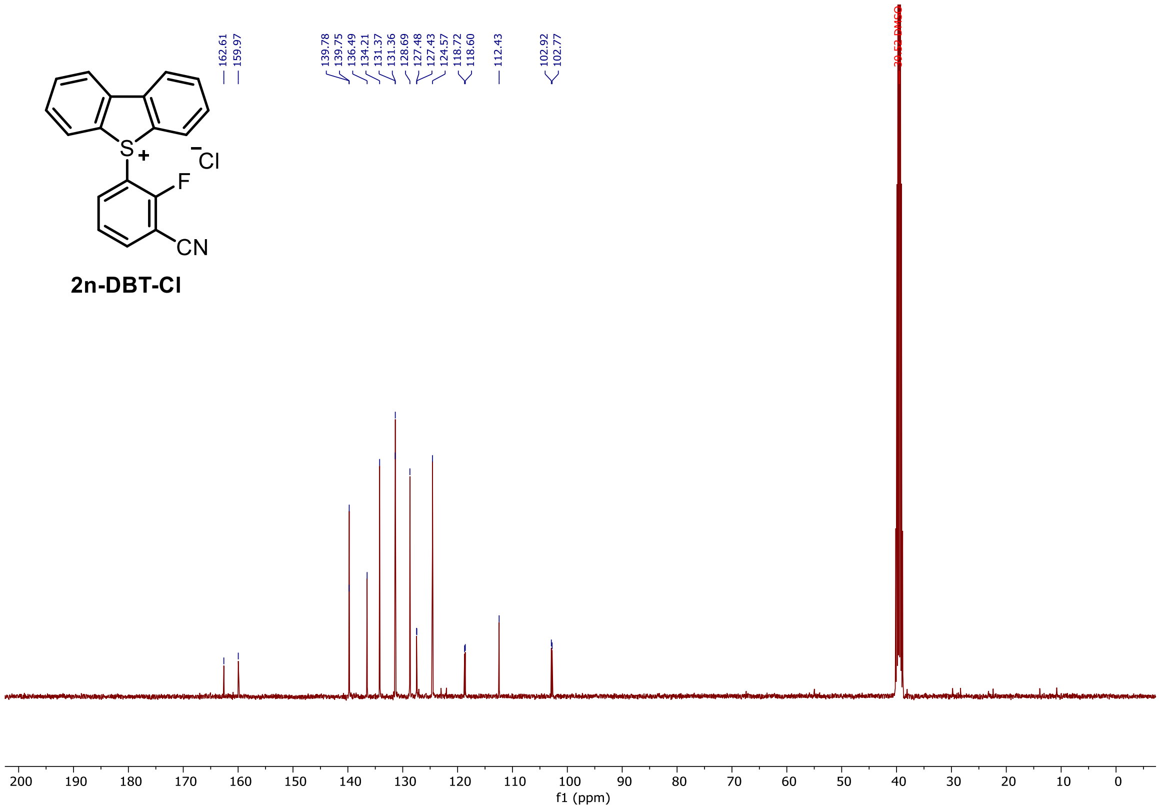

^{19}F NMR (471 MHz, DMSO-d$_6$) of **2n-DBT-Cl**

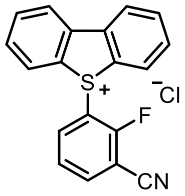

2n-DBT-Cl

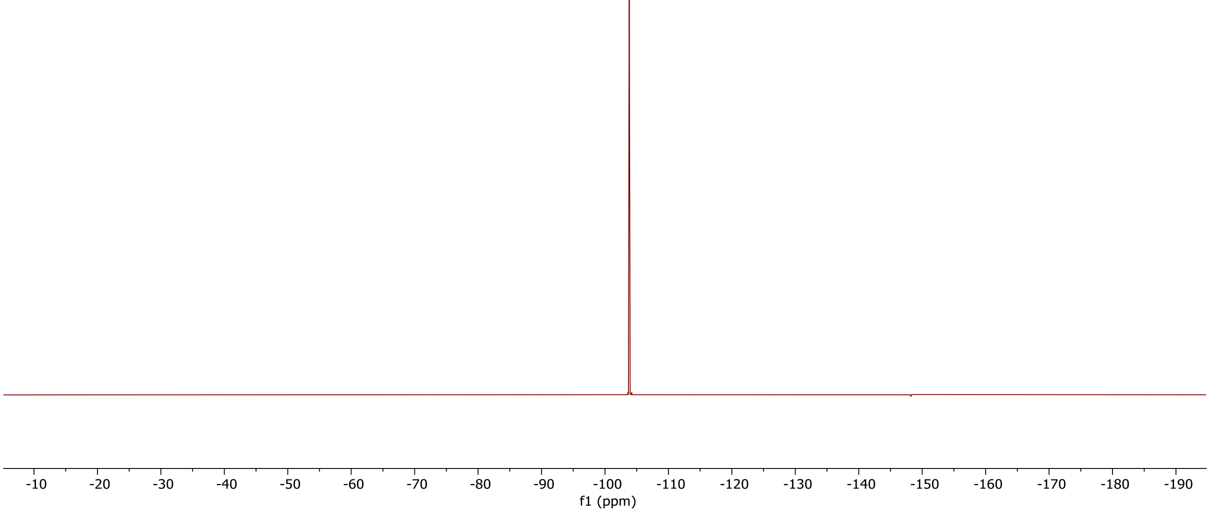

^{1}H NMR (400 MHz, DMSO-d$_6$) of **2o-DBT-Cl**

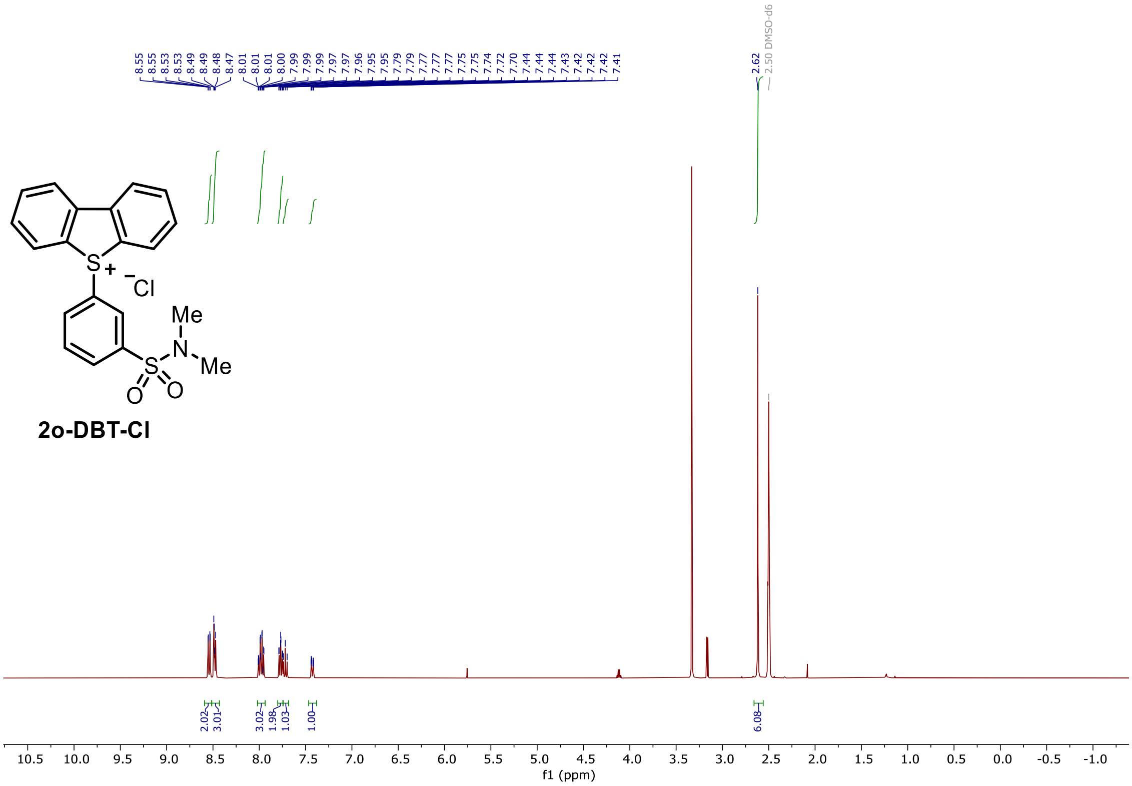

^{13}C NMR (101 MHz, DMSO-d$_6$) of **2o-DBT-Cl**

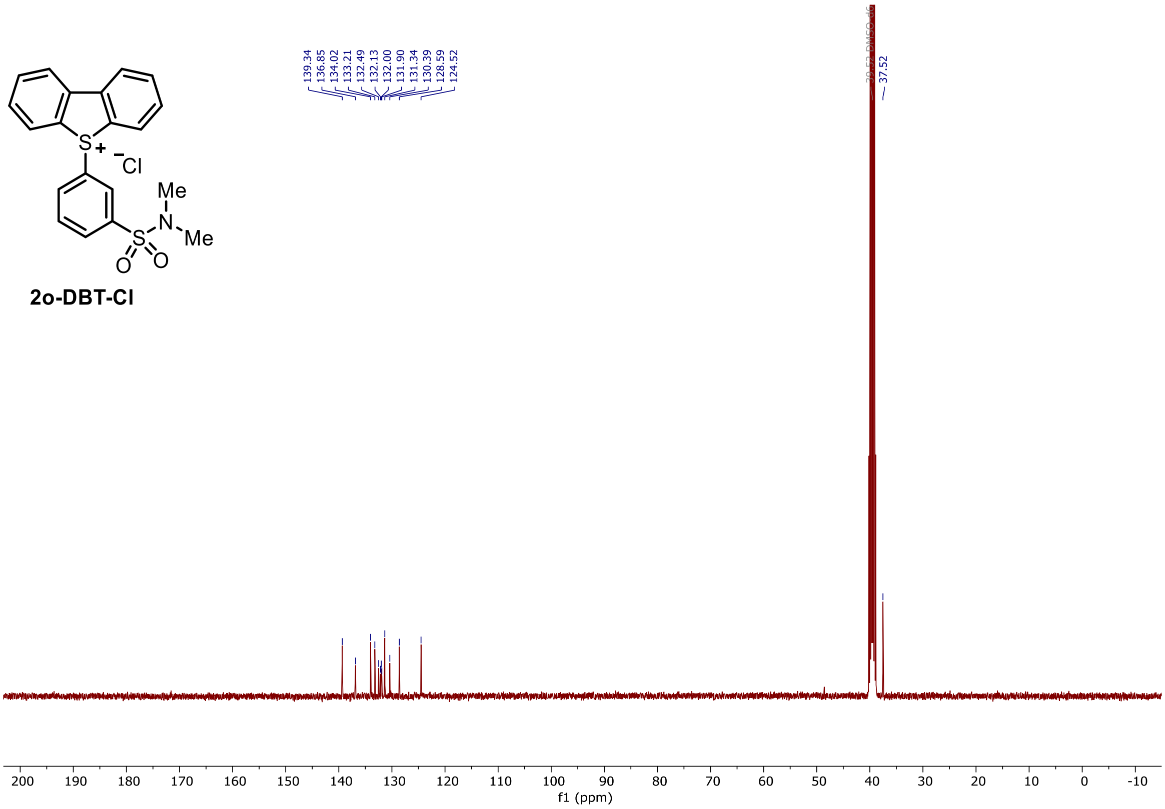

^{1}H NMR (500 MHz, DMSO-d$_6$) of **2p-DBT-Cl**

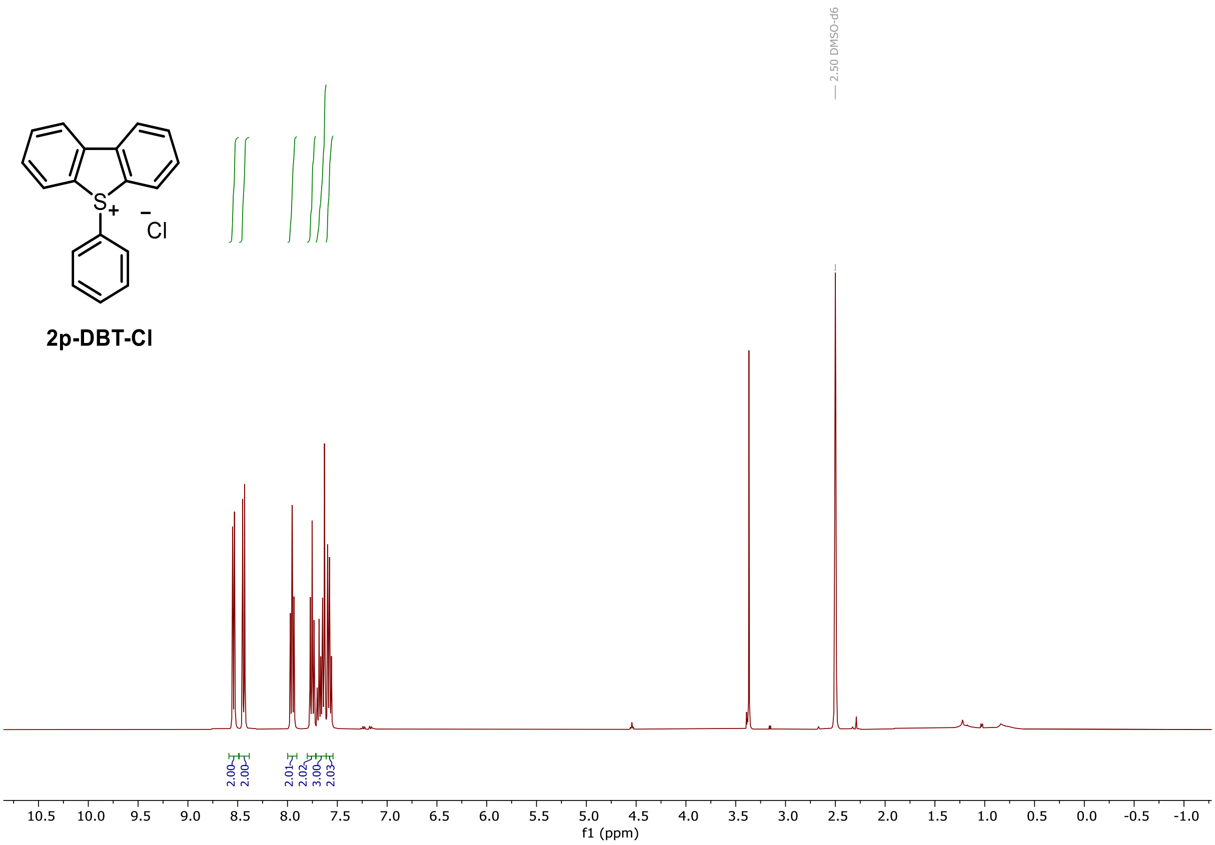

^{13}C NMR (126 MHz, DMSO-d$_6$) of **2p-DBT-Cl**

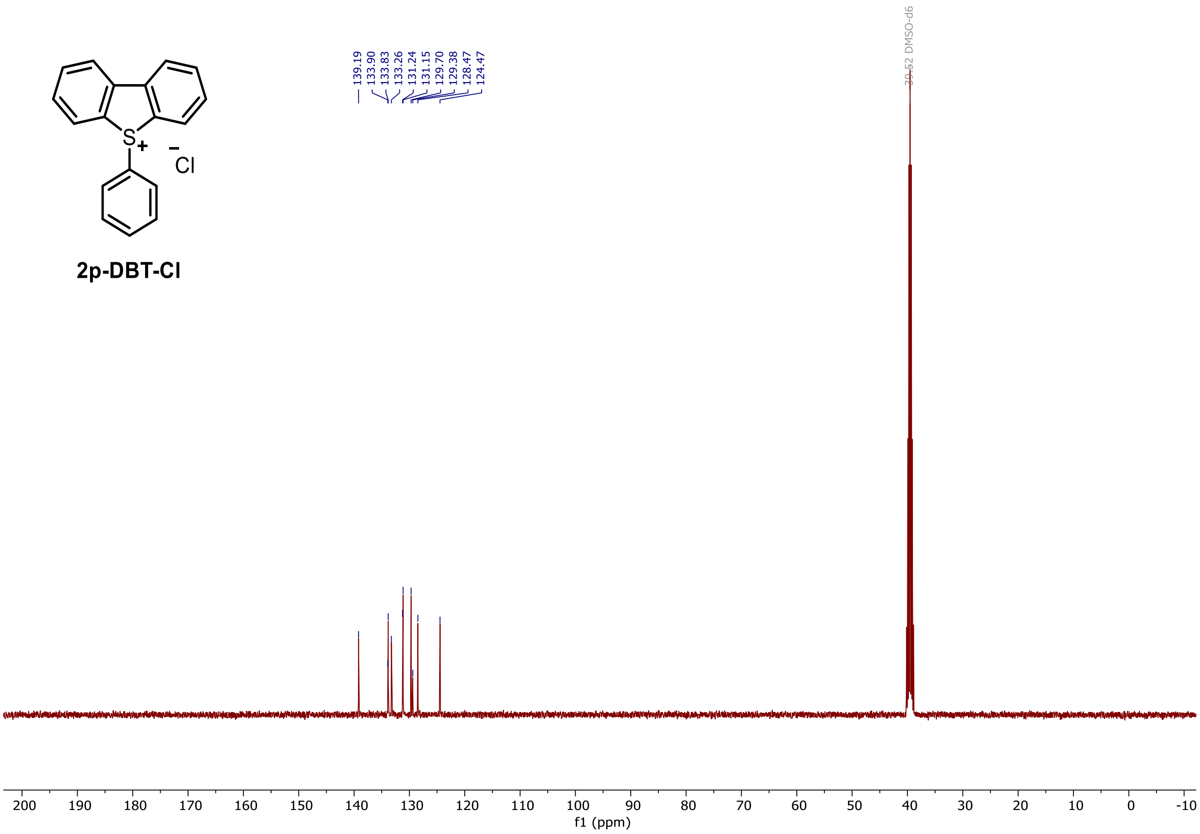

^{1}H NMR (500 MHz, DMSO-d$_6$) of **2q-DBT-Cl**

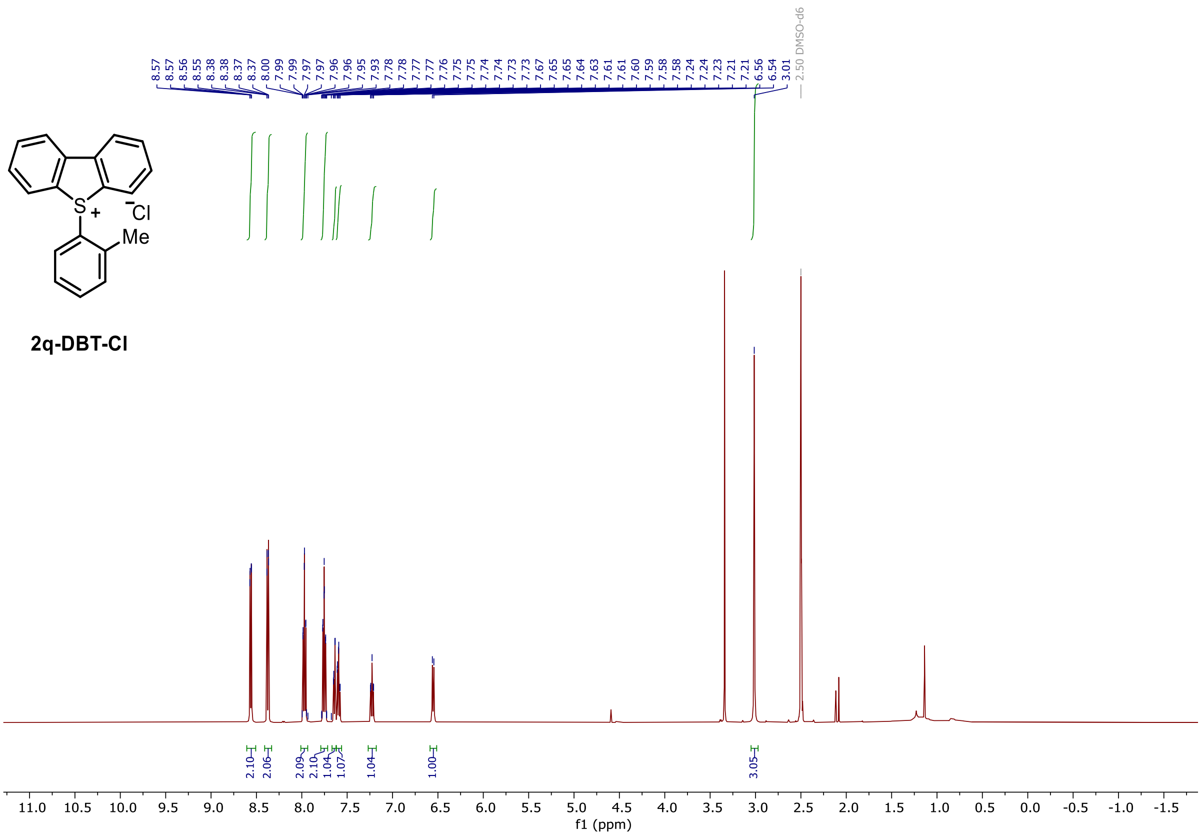

^{13}C NMR (126 MHz, DMSO-d$_6$) **2q-DBT-Cl**

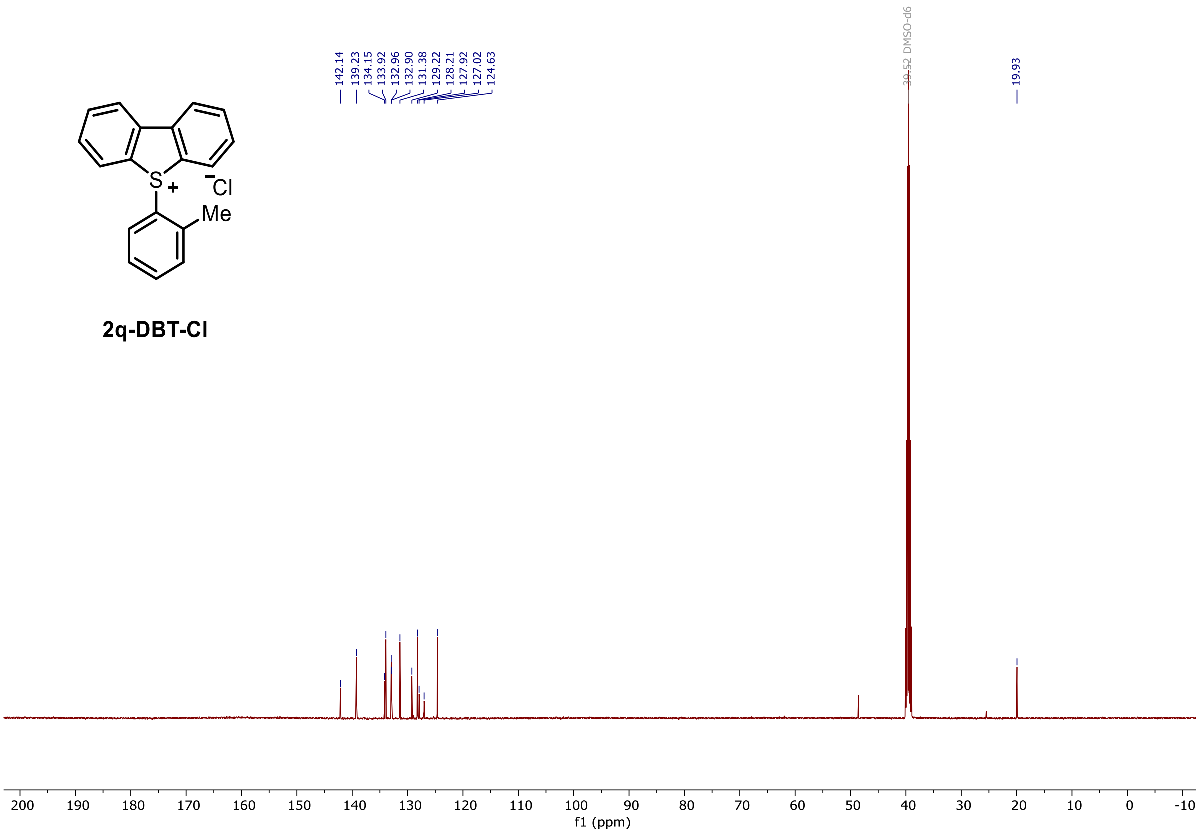

^{1}H NMR (400 MHz, DMSO-d$_6$) of **2r-DBT-Cl**

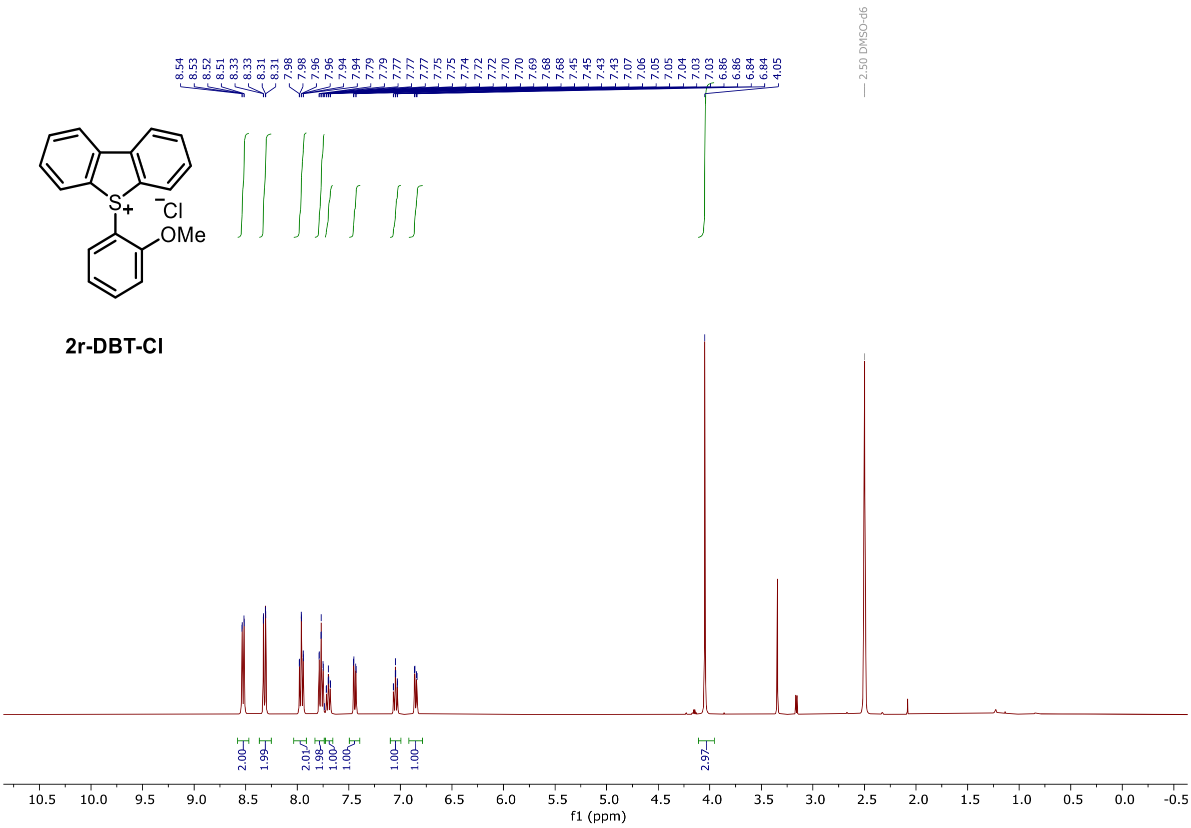

^{13}C NMR (101 MHz, DMSO-d$_6$) of **2r-DBT-Cl**

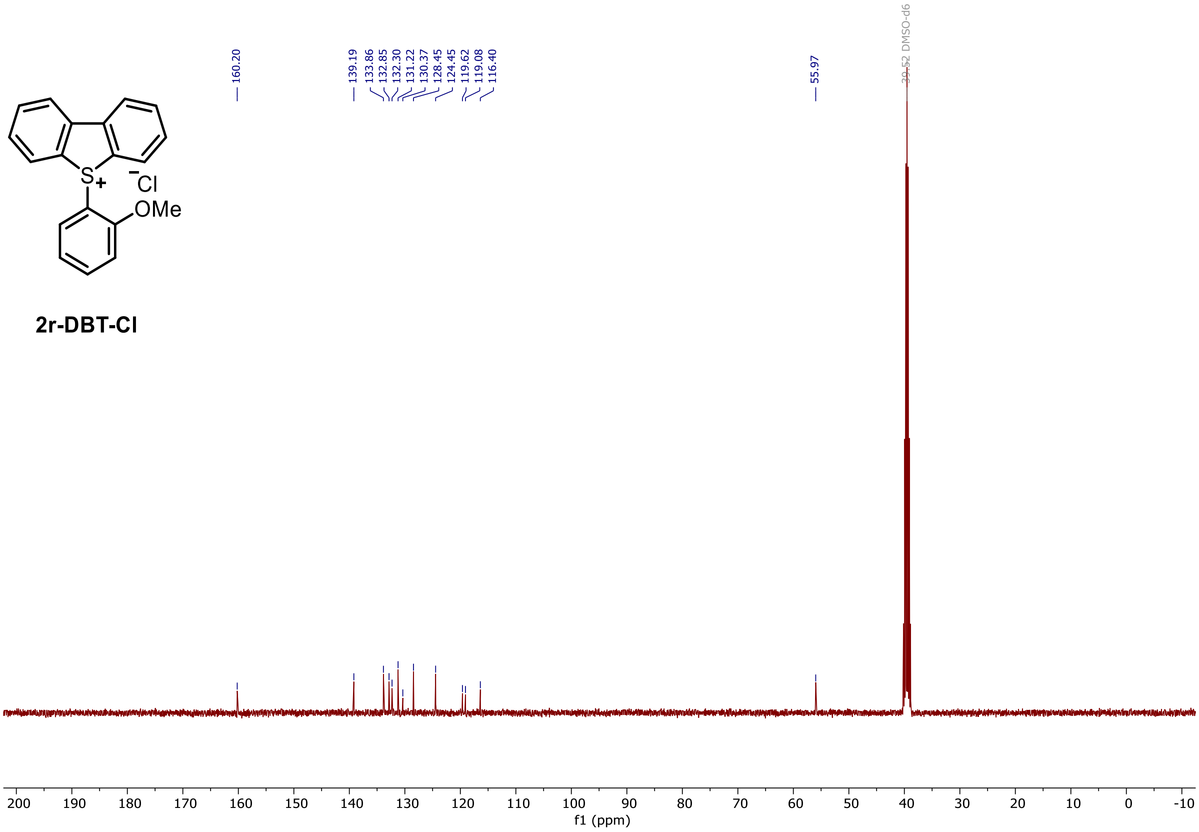

^1H NMR (400 MHz, DMSO-d$_6$) of **2s-DBT-Cl**

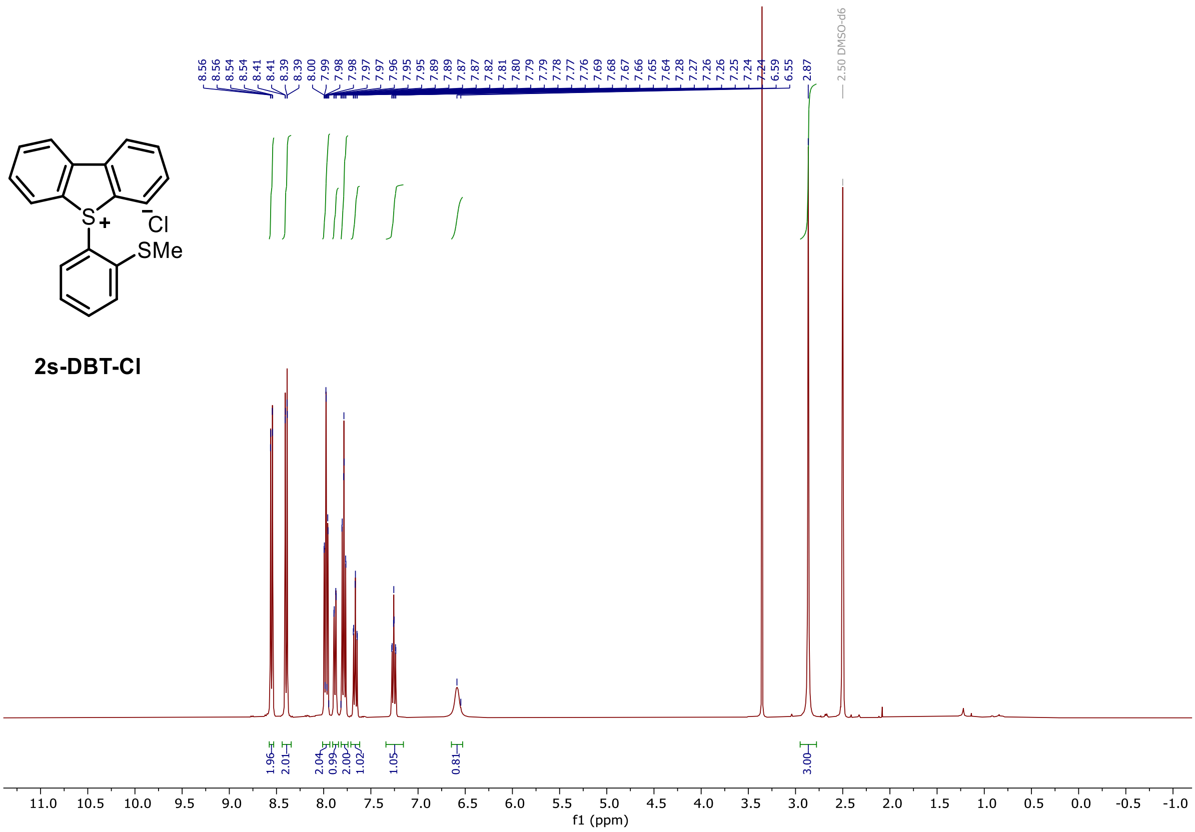

^{13}C NMR (101 MHz, DMSO-d$_6$) of **2s-DBT-Cl**

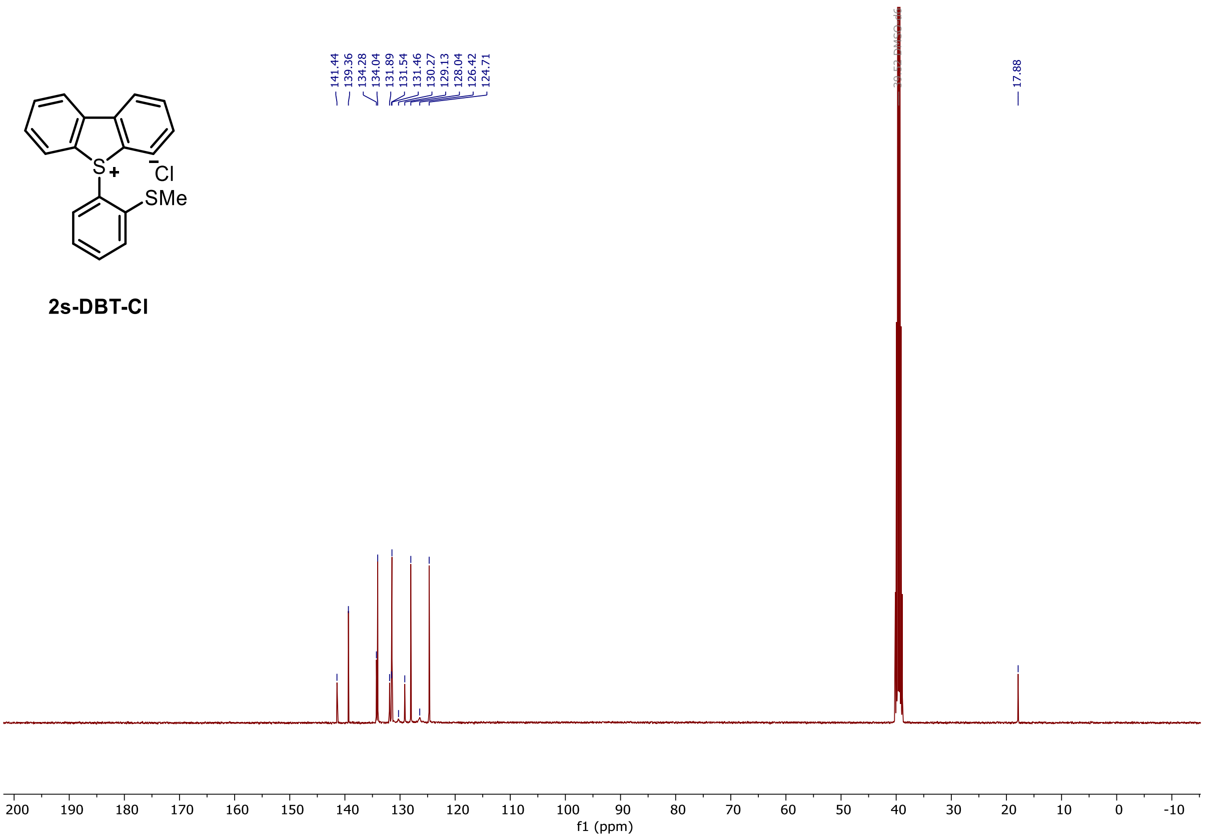

^{1}H NMR (500 MHz, DMSO-d$_6$) of **2t-DBT-Cl**

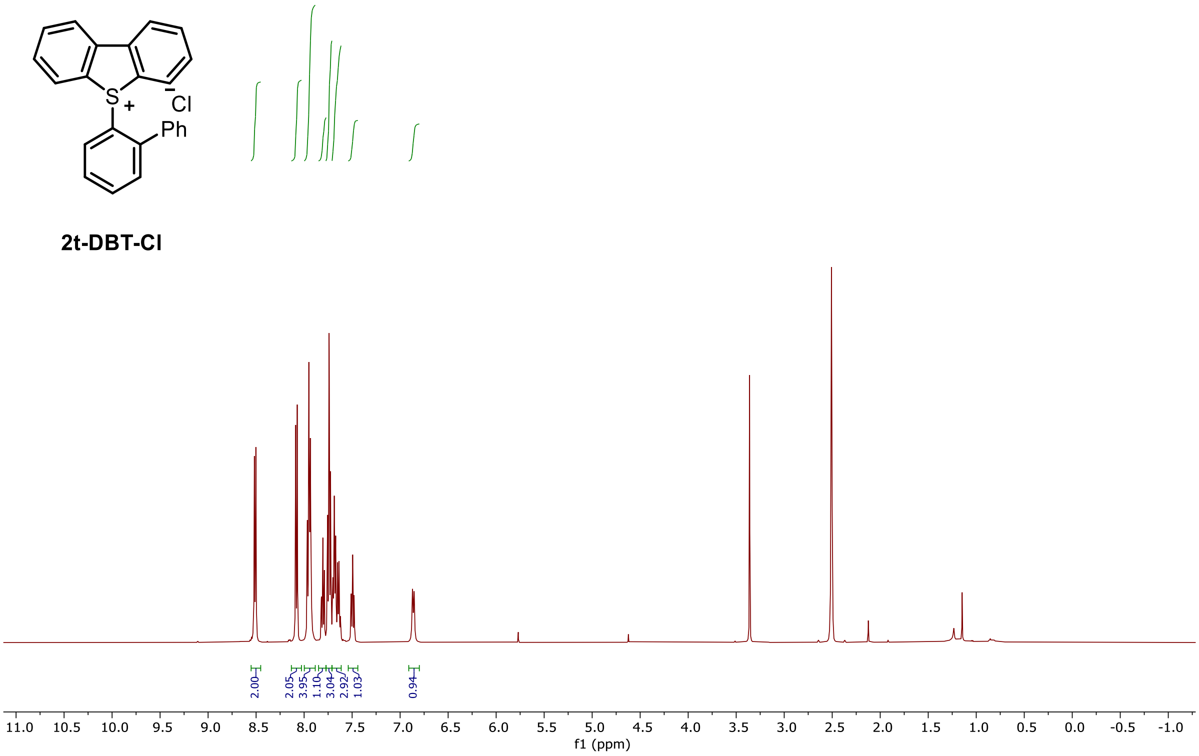

^{13}C NMR (126 MHz, DMSO-d$_6$) of **2t-DBT-Cl**

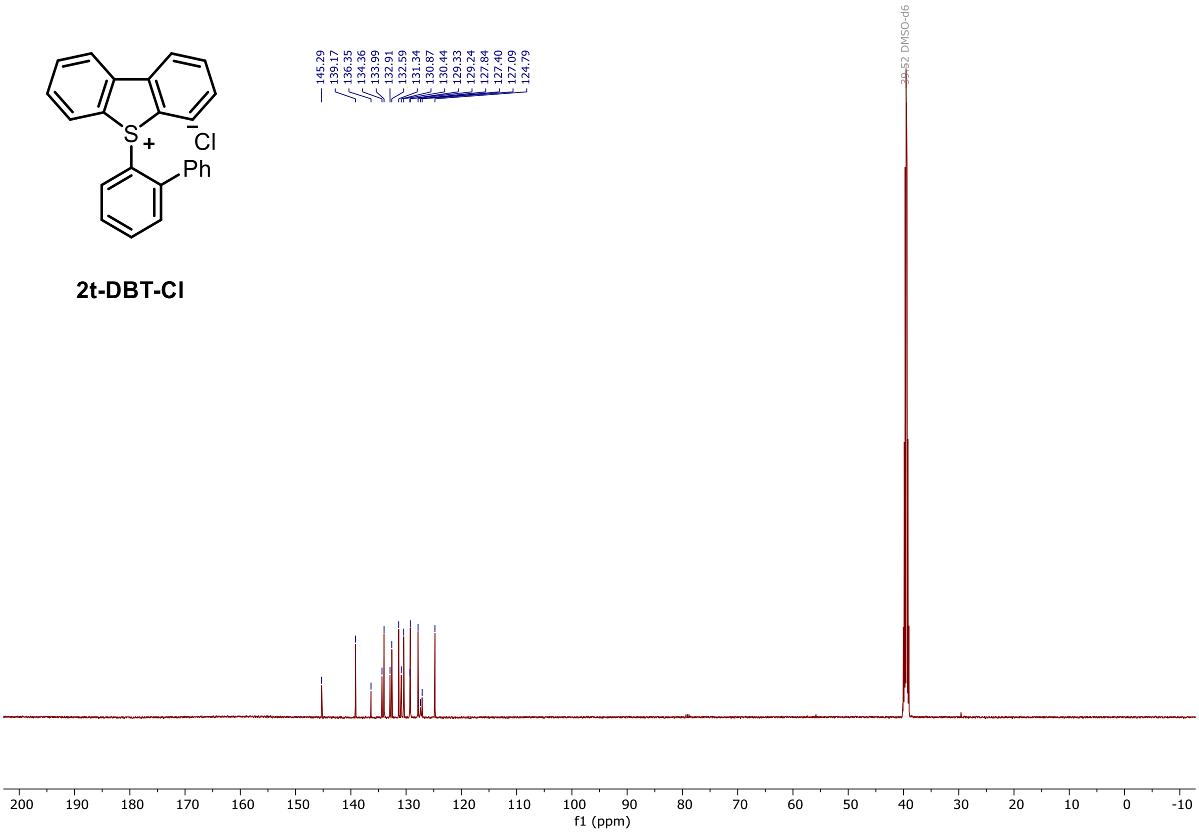

^{1}H NMR (500 MHz, DMSO-d$_6$) of **2u-DBT-Cl**

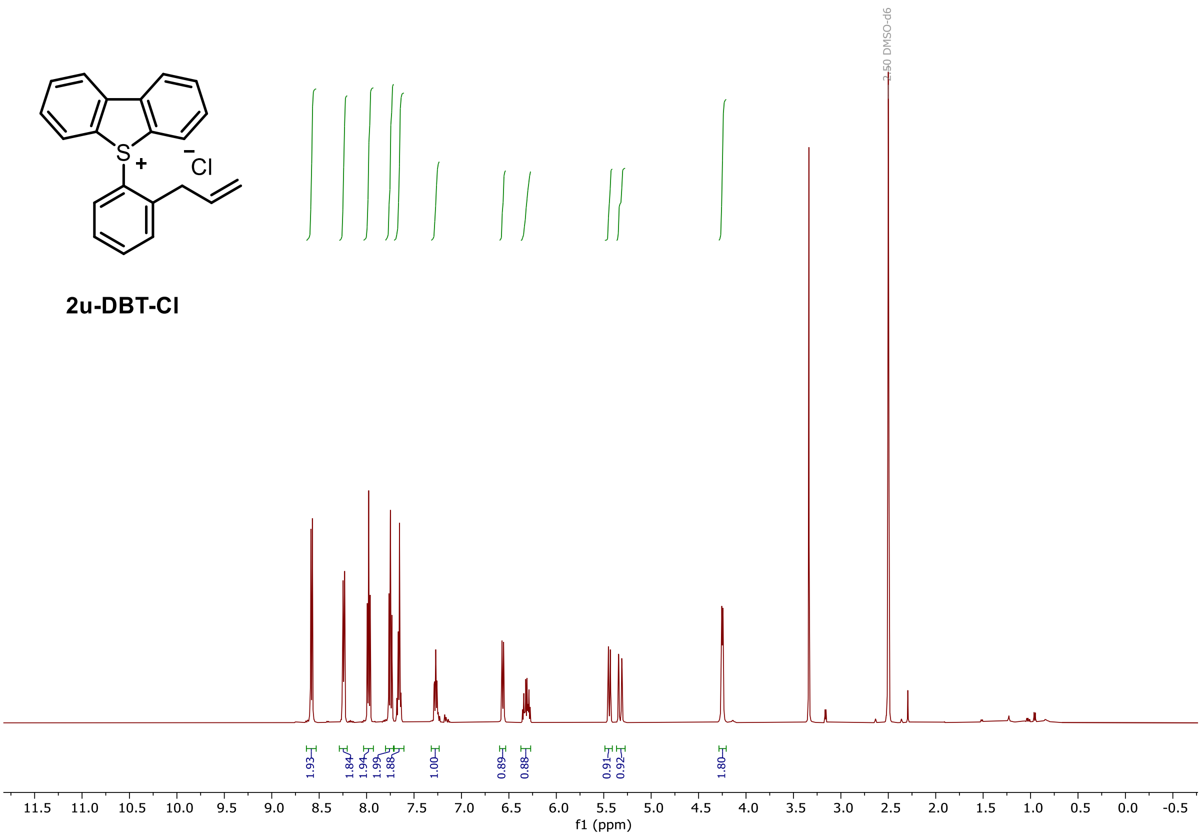

^{13}C NMR (126 MHz, DMSO-d$_6$) of **2u-DBT-Cl**

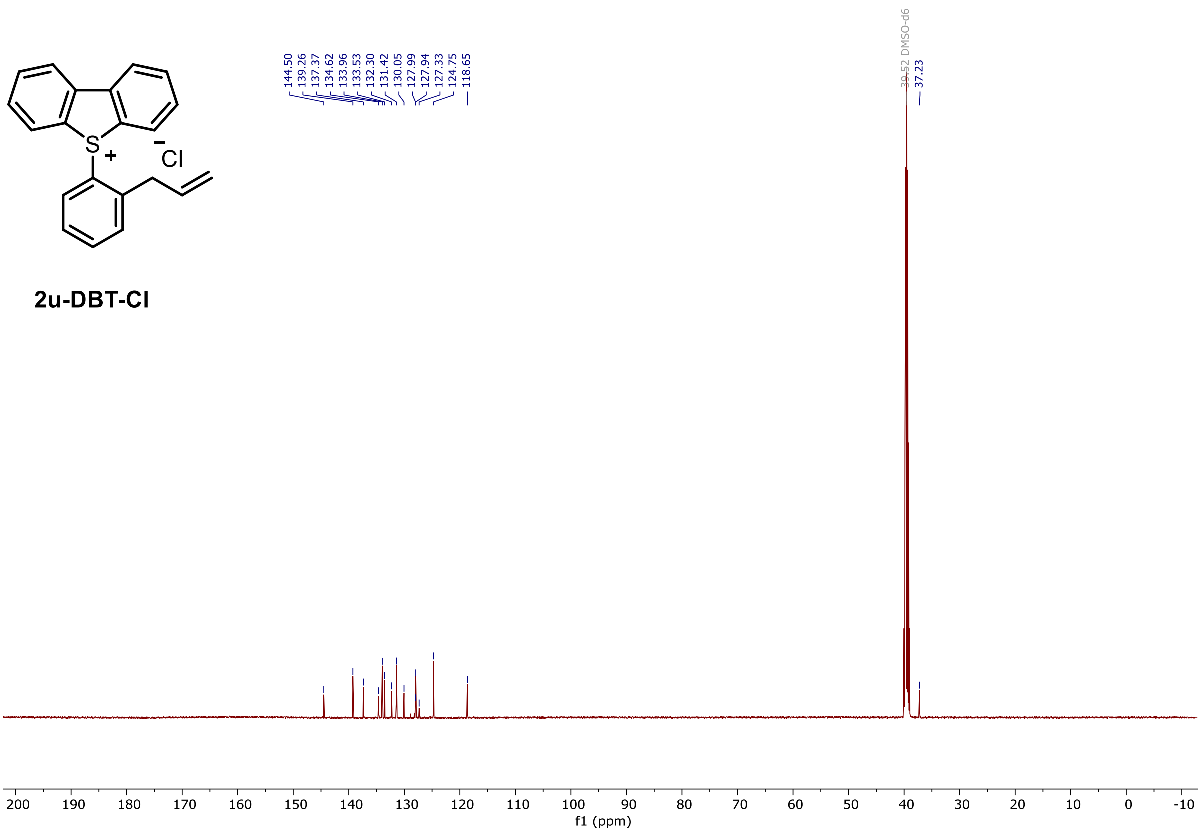

^{1}H NMR (400 MHz, DMSO-d$_6$) of **2v-DBT-Cl**

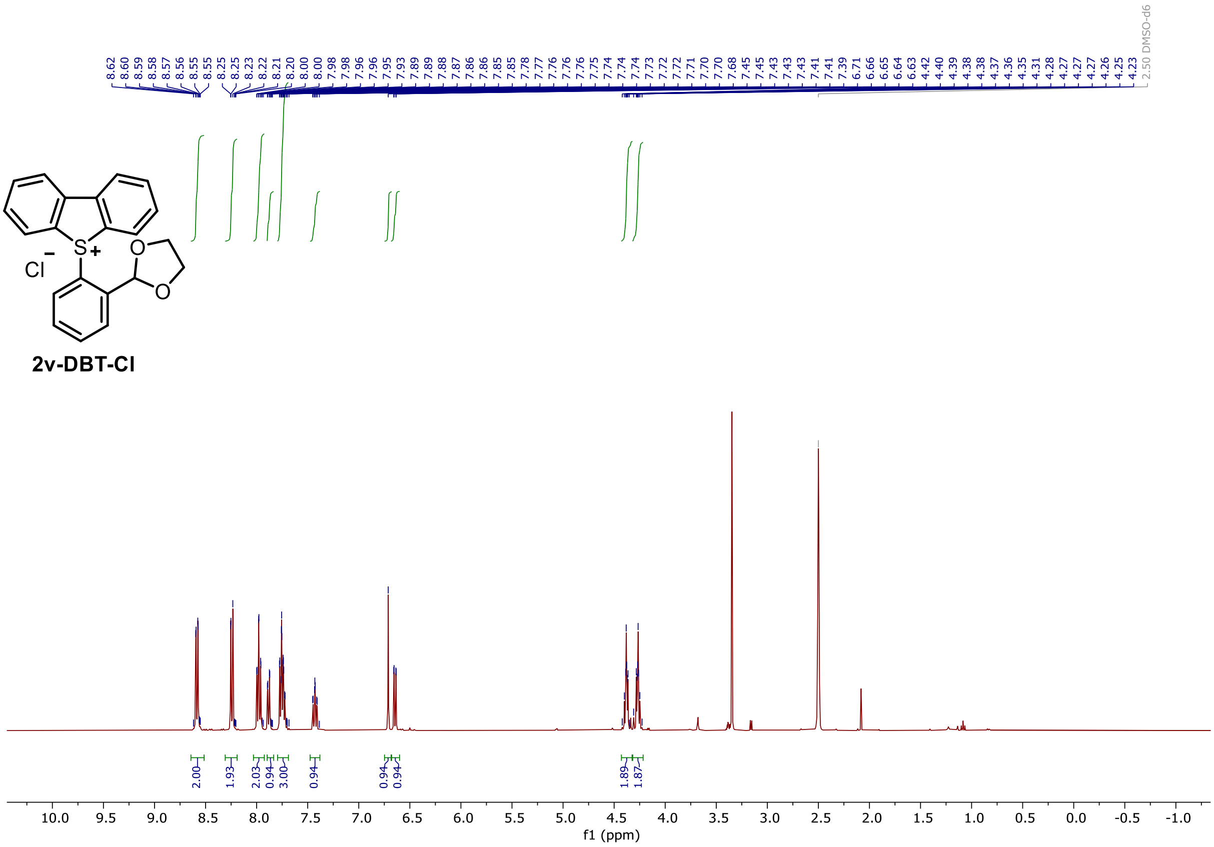

^{13}C NMR (101 MHz, DMSO-d$_6$) of **2v-DBT-Cl**

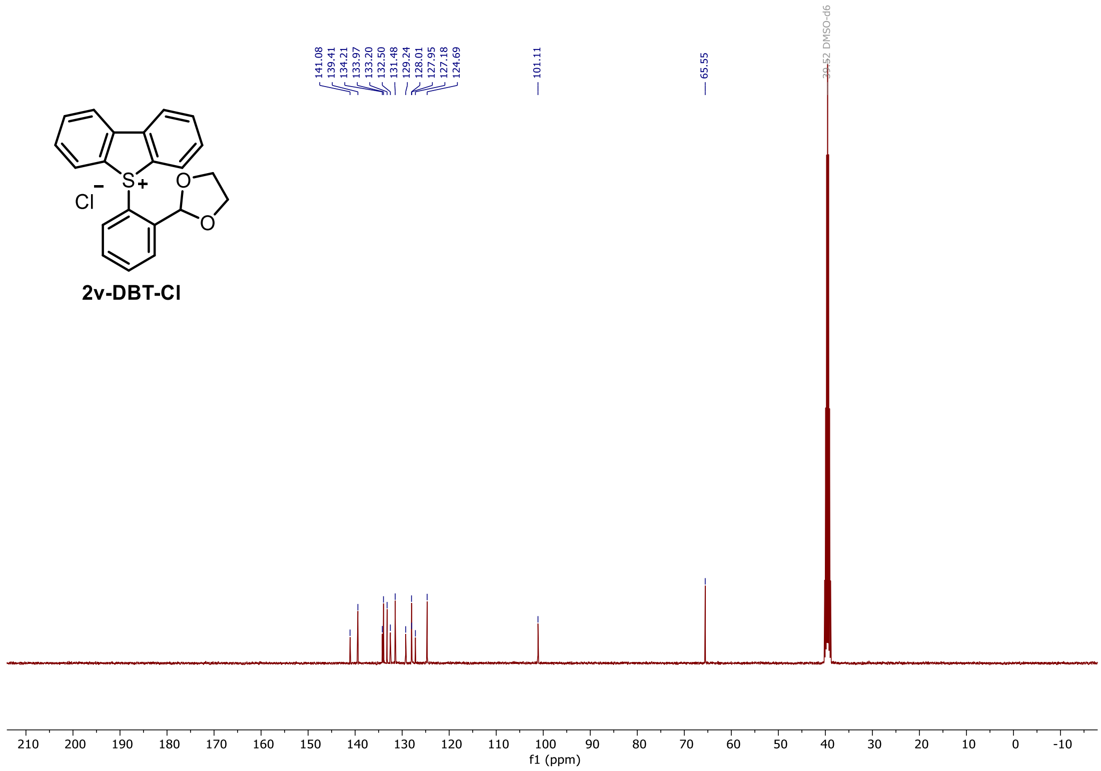

^{1}H NMR (400 MHz, DMSO-d$_6$) of **2w-DBT-Cl**

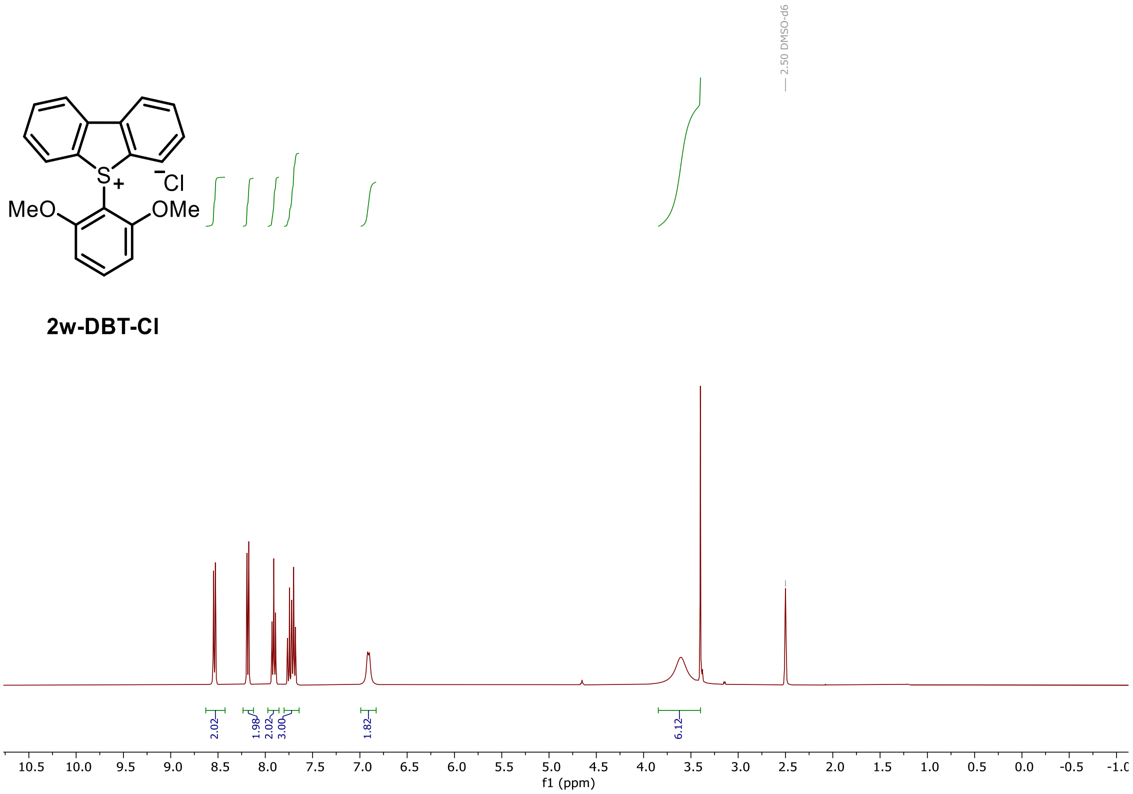

^{13}C NMR (101 MHz, DMSO-d$_6$) of **2w-DBT-Cl**

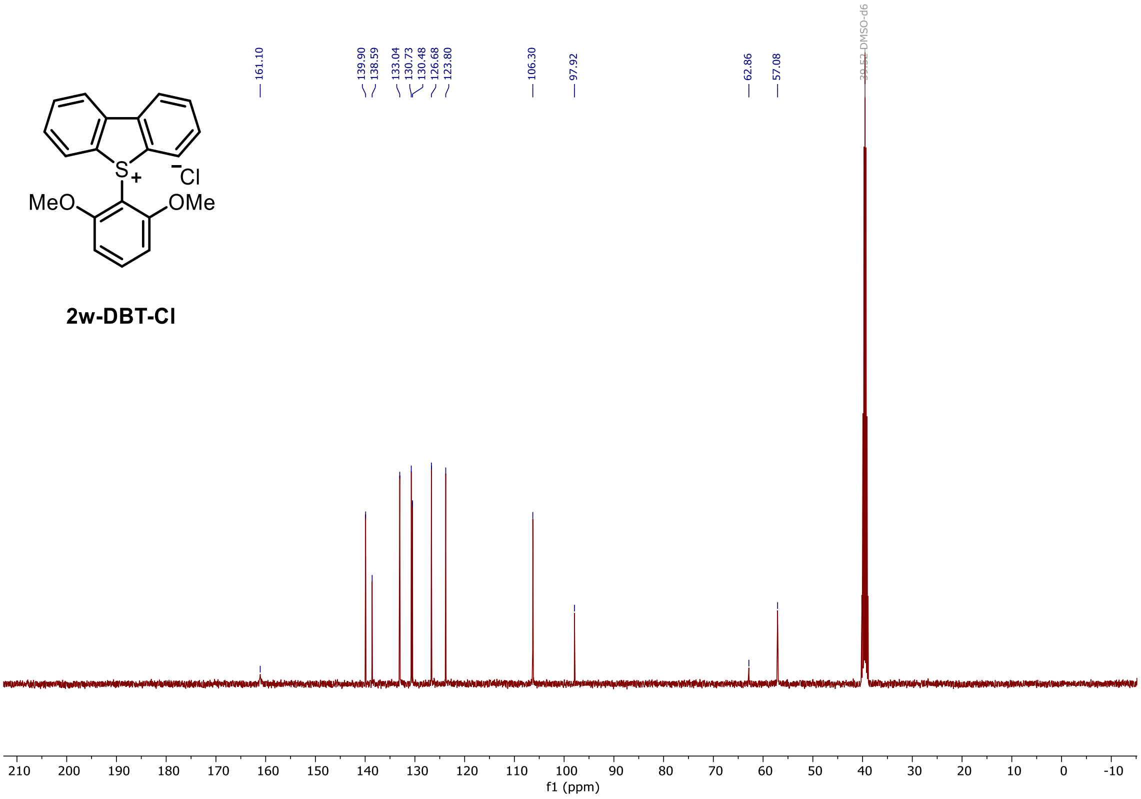

^{1}H NMR (500 MHz, DMSO-d$_6$) of **2x-DBT-Cl**

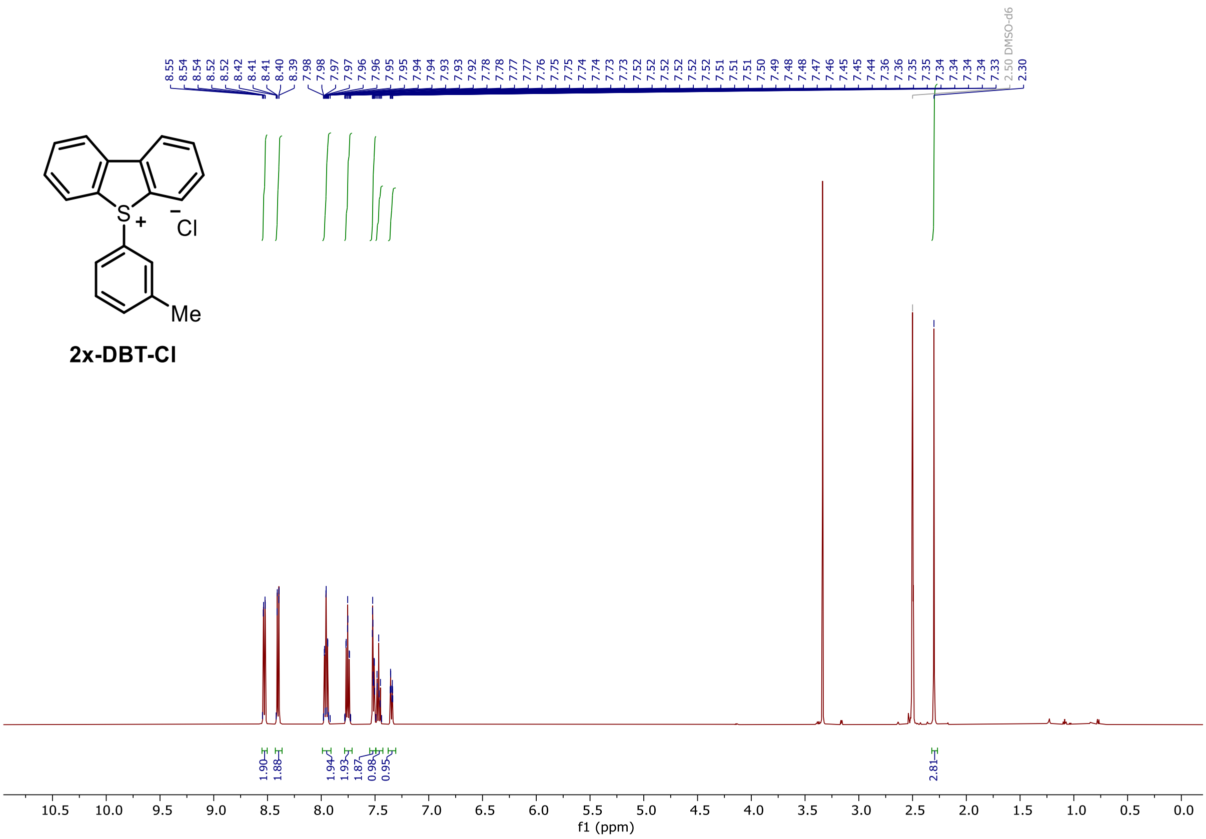

^{13}C NMR (126 MHz, DMSO-d$_6$) of **2x-DBT-Cl**

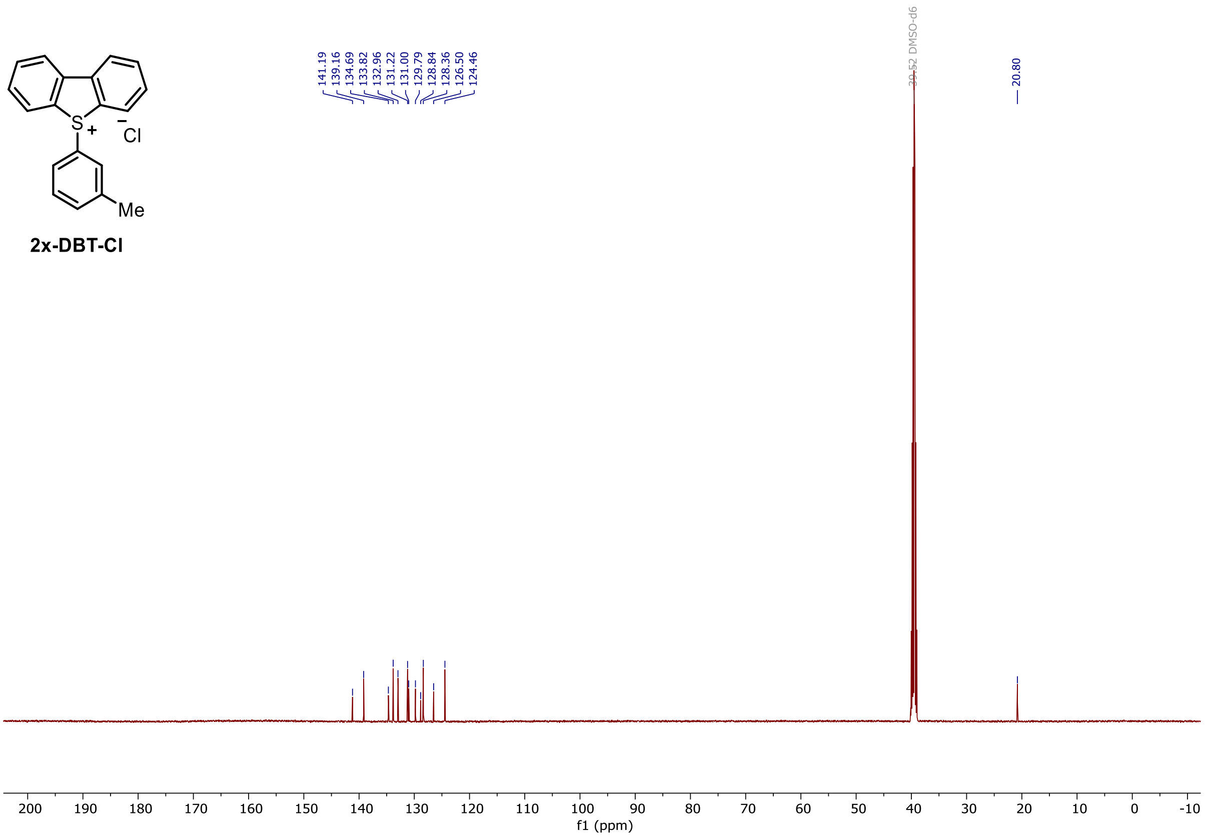

^{1}H NMR (500 MHz, DMSO-d$_6$) of **2y-DBT-Cl**

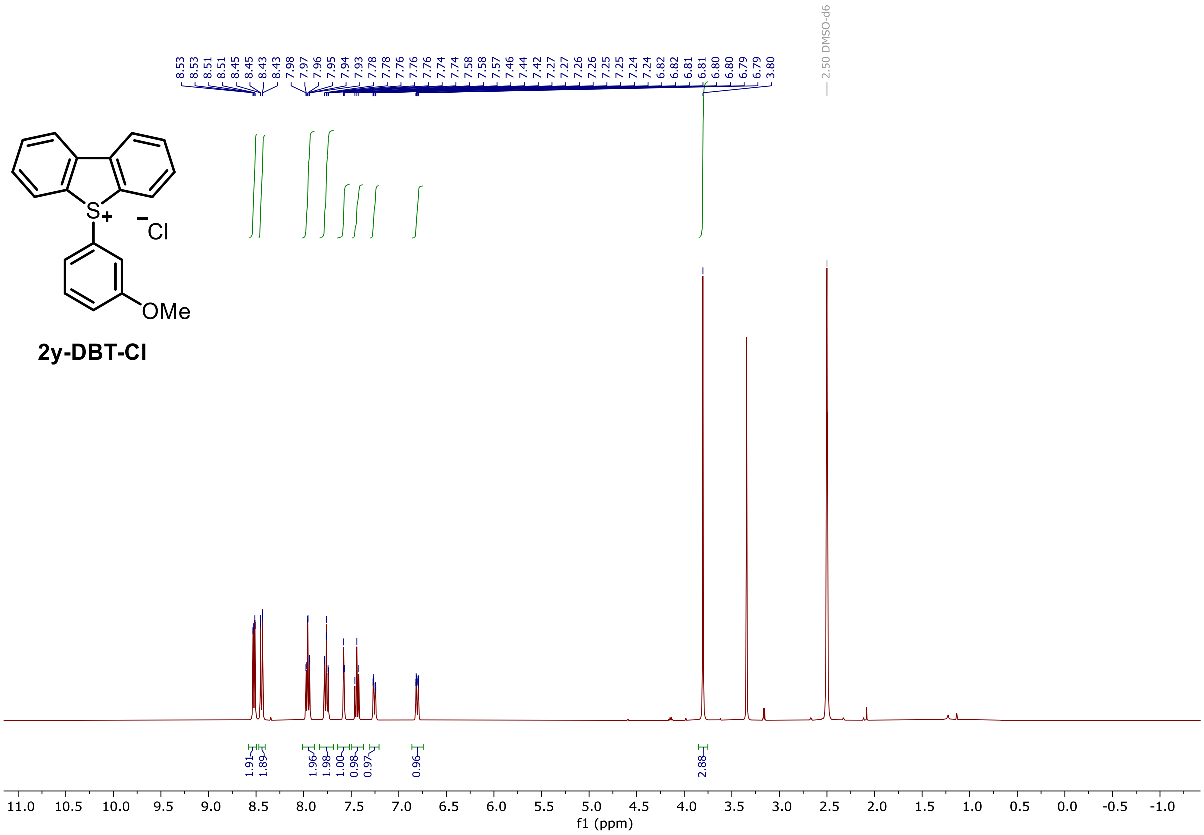

^{13}C NMR (126 MHz, DMSO-d$_6$) of **2y-DBT-Cl**

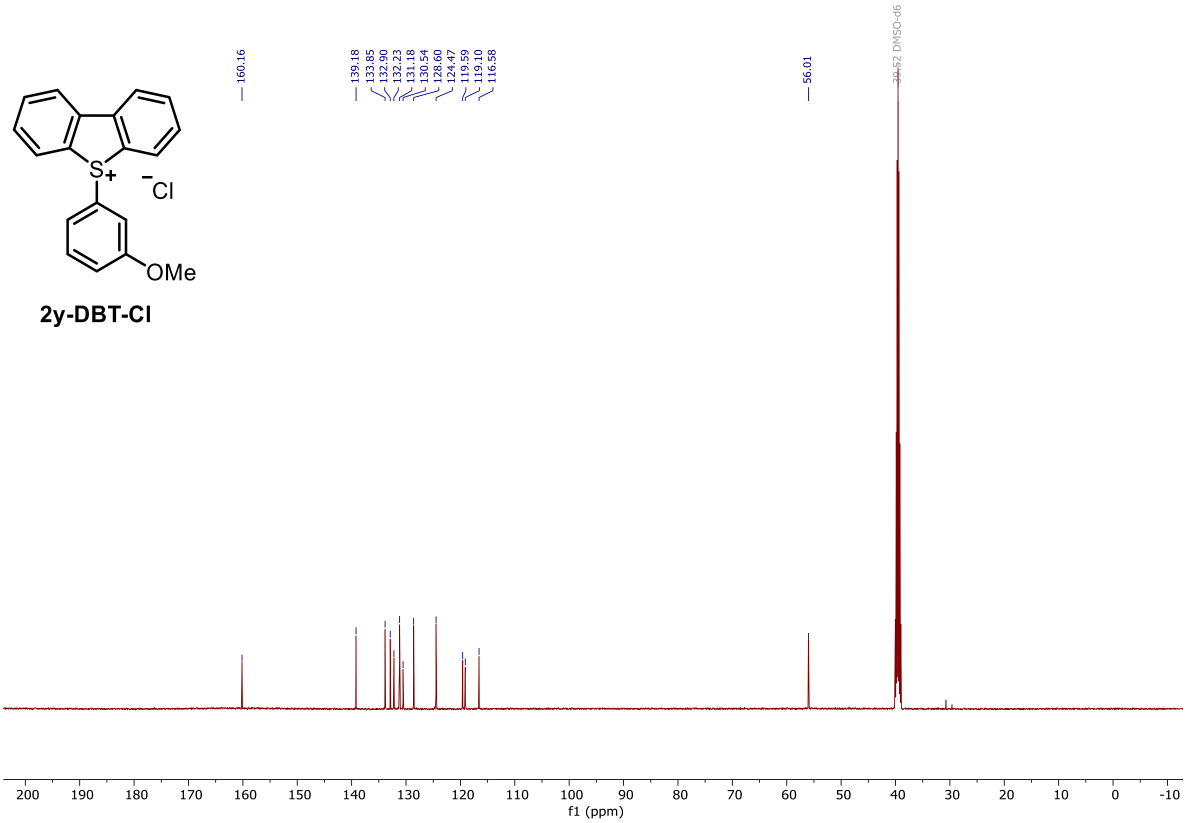

^1H NMR (500 MHz, DMSO-d$_6$) of **2z-DBT-Cl**

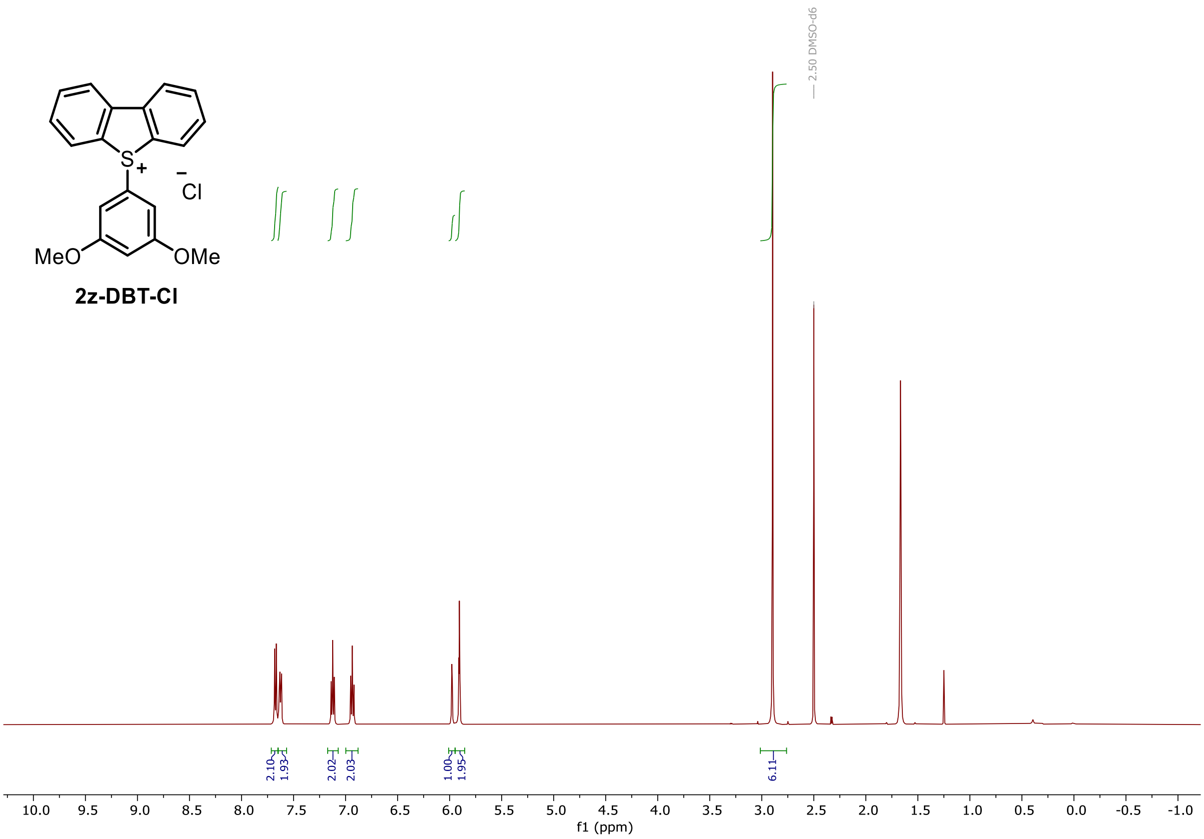

^{13}C NMR (126 MHz, DMSO-d$_6$) of **2z-DBT-Cl**

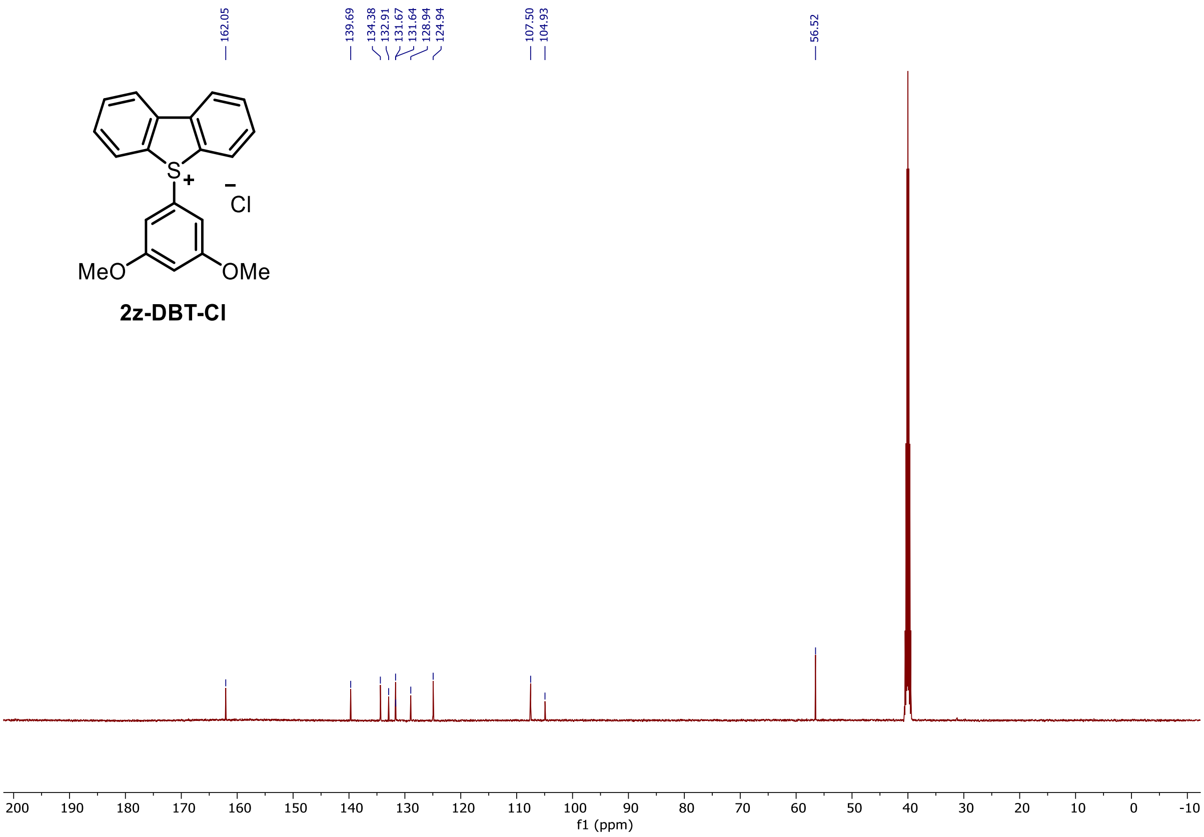

^{1}H NMR (400 MHz, DMSO-d$_6$) of **2aa-DBT-Cl**

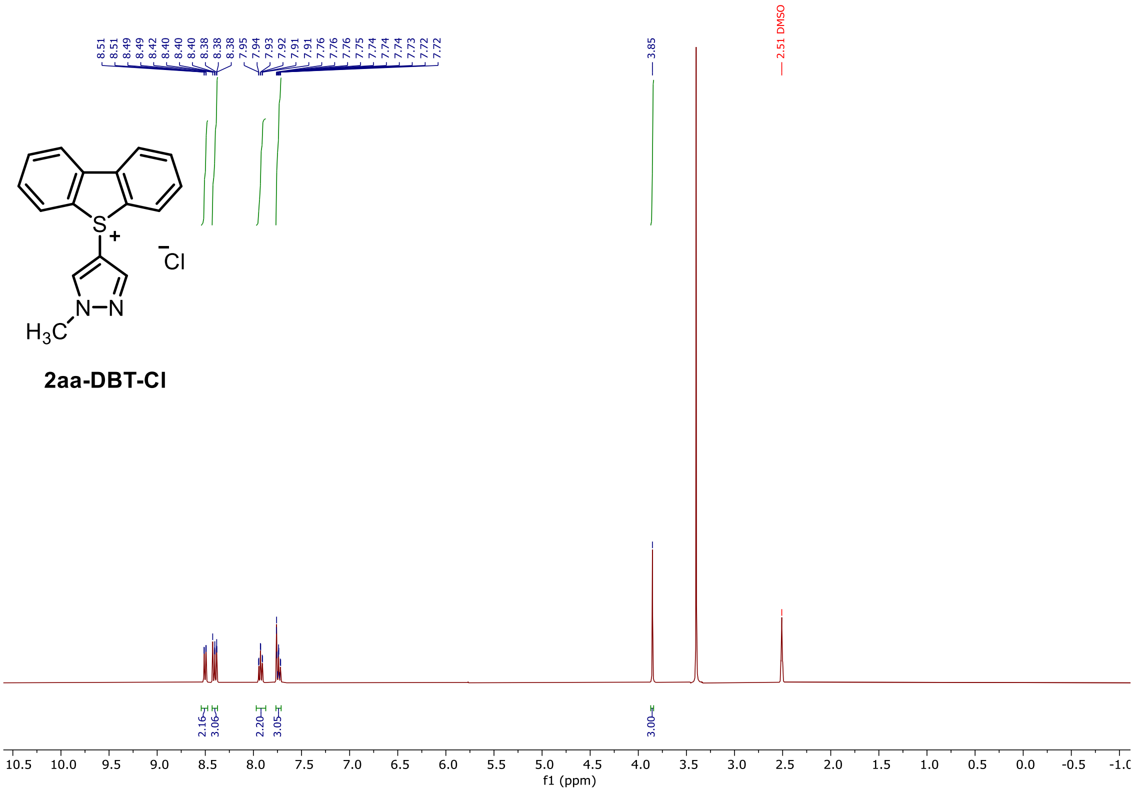

^{13}C NMR (101 MHz, DMSO-d$_6$) of **2aa-DBT-Cl**

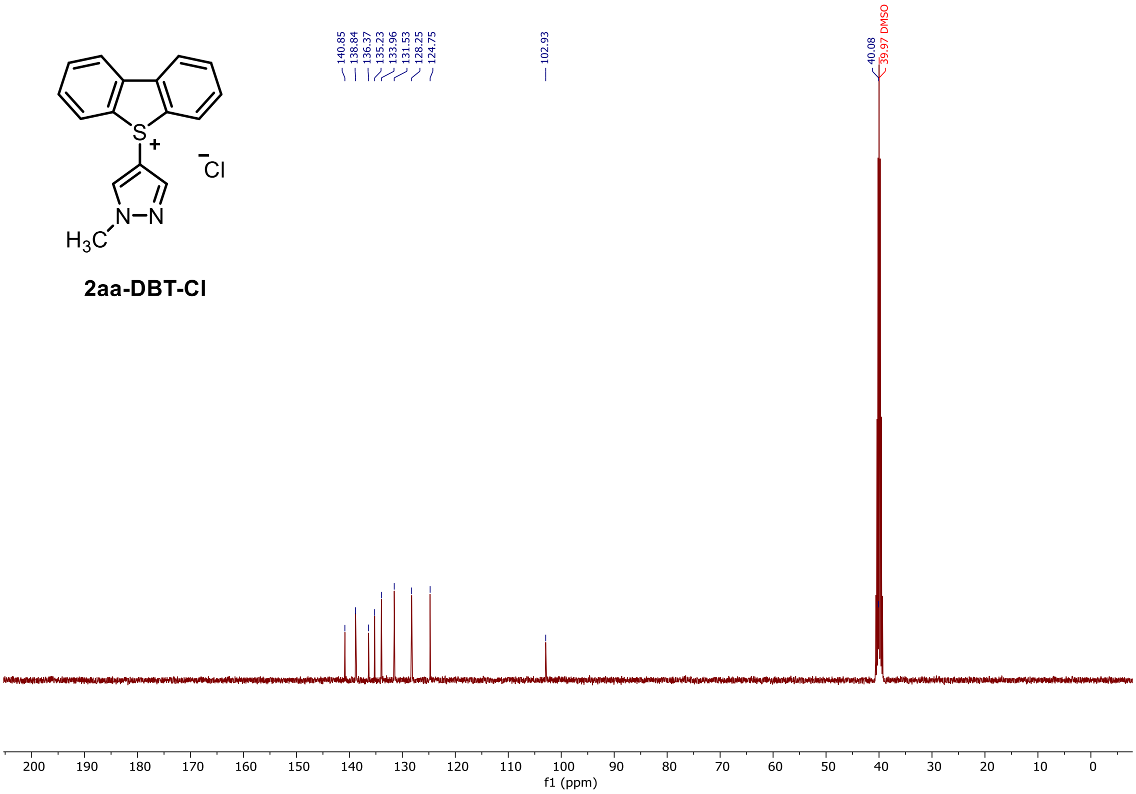

^1H NMR (400 MHz, DMSO-d$_6$) **2ab-DBT-Cl**

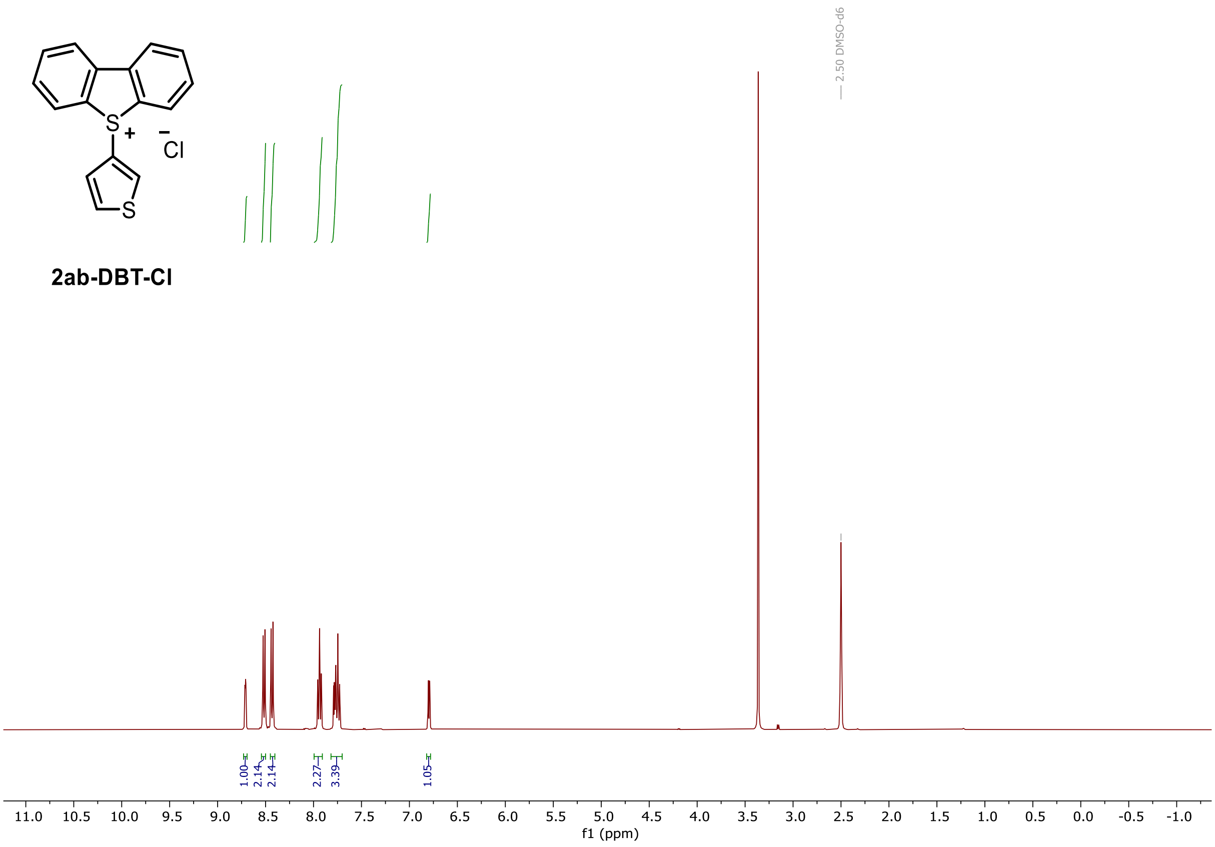

^{13}C NMR (101 MHz, DMSO-d$_6$) **2ab-DBT-Cl**

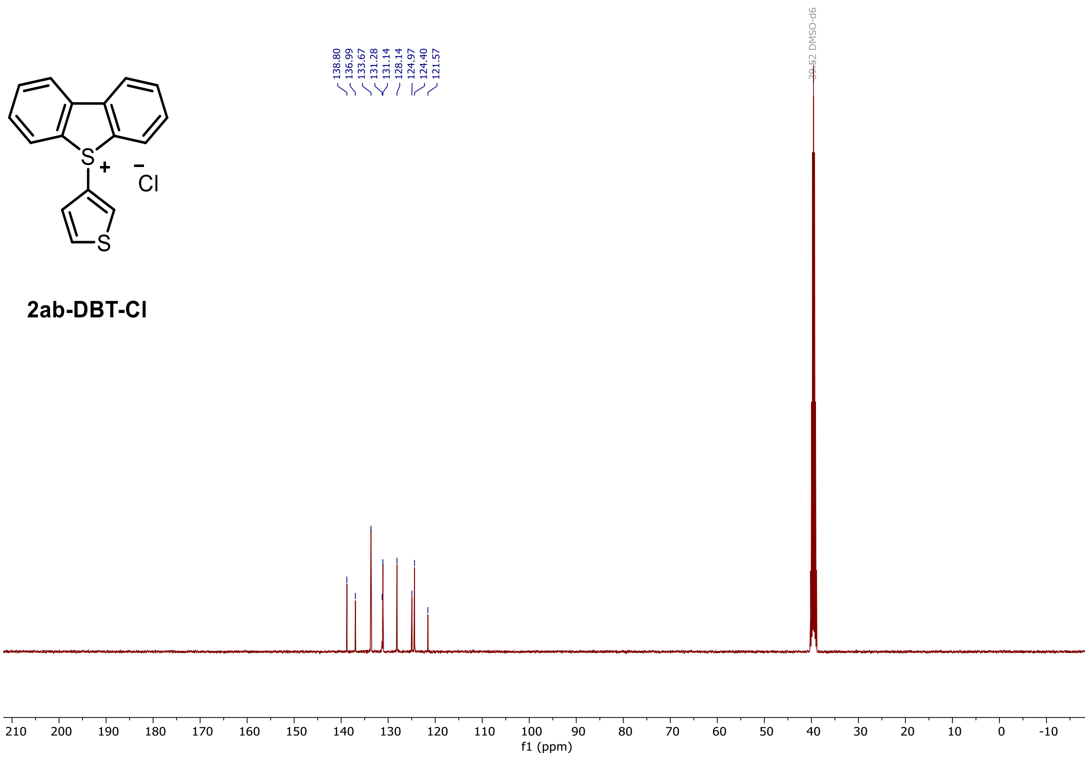

^{1}H NMR (400 MHz, DMSO-d_6) **2ac-DBT-Cl**

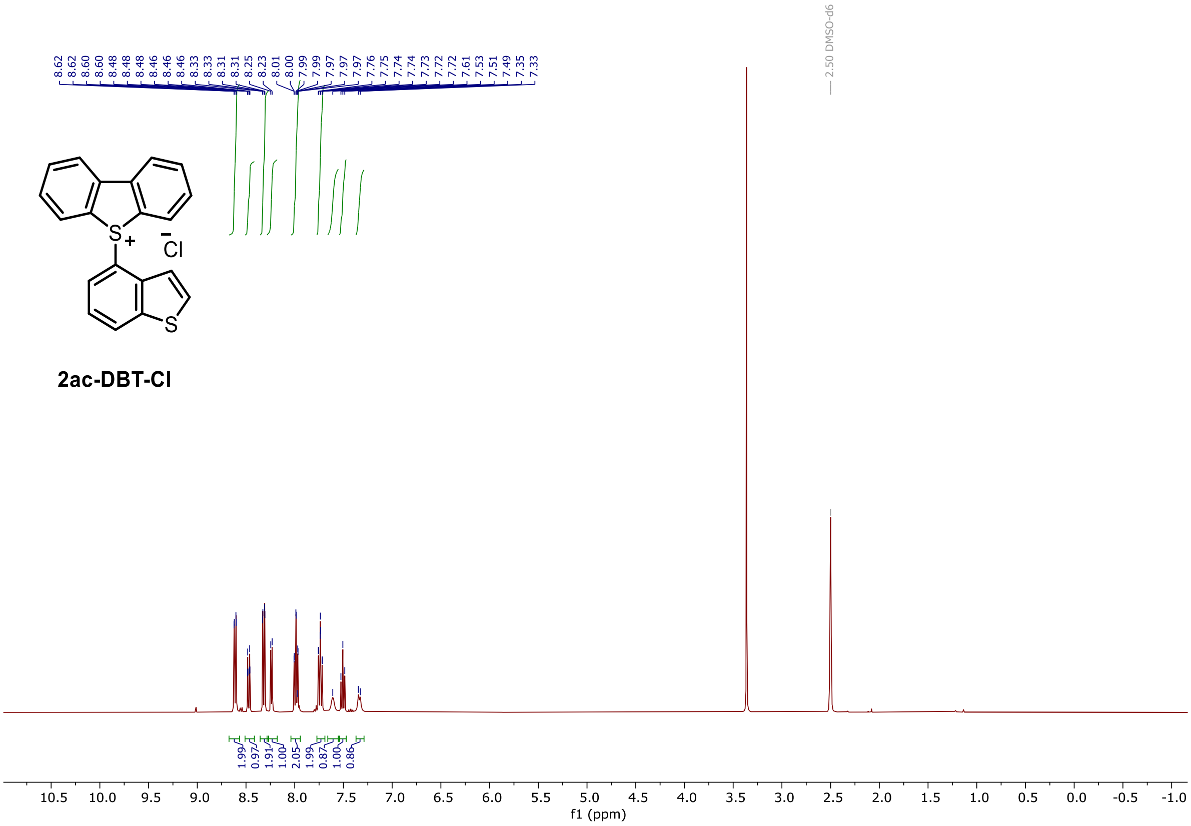

^{13}C NMR (101 MHz, DMSO-d_6) **2ac-DBT-Cl**

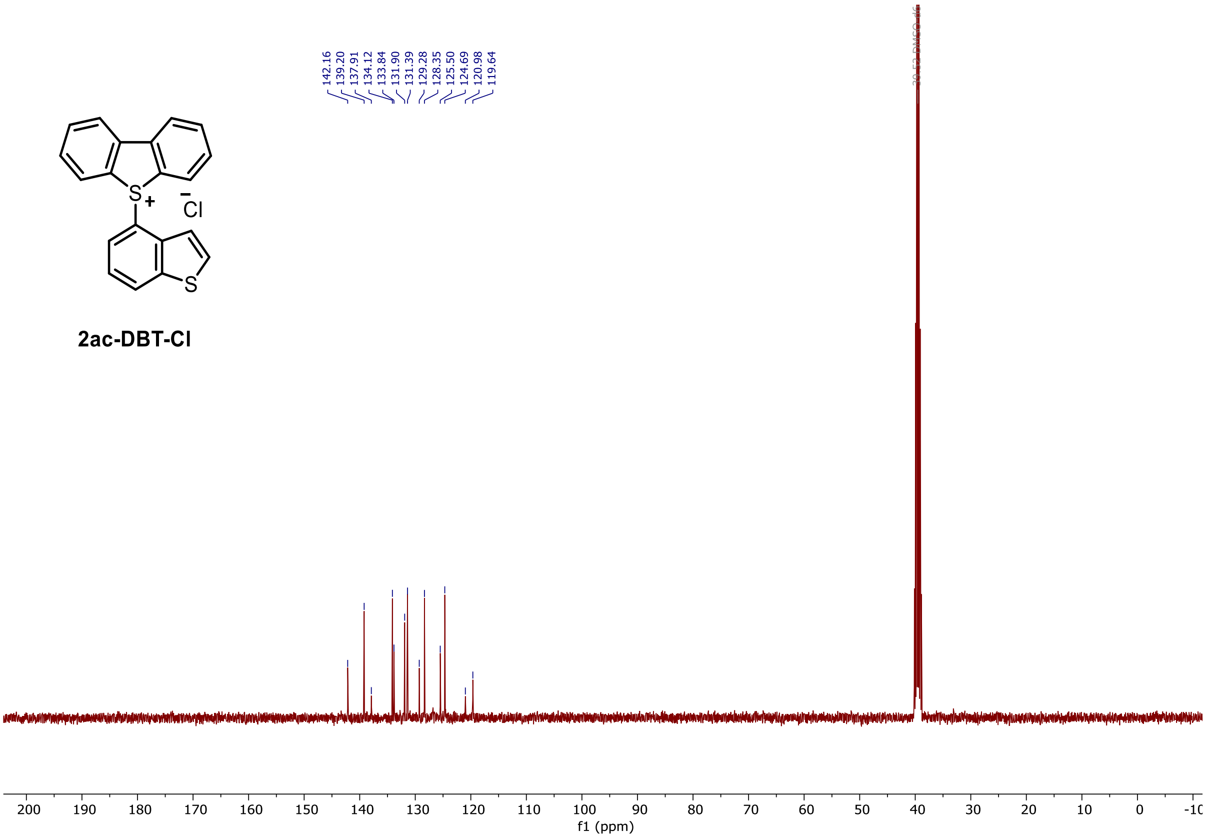

^{1}H NMR (500 MHz, DMSO-d$_6$) **2ac-PXT-Cl**

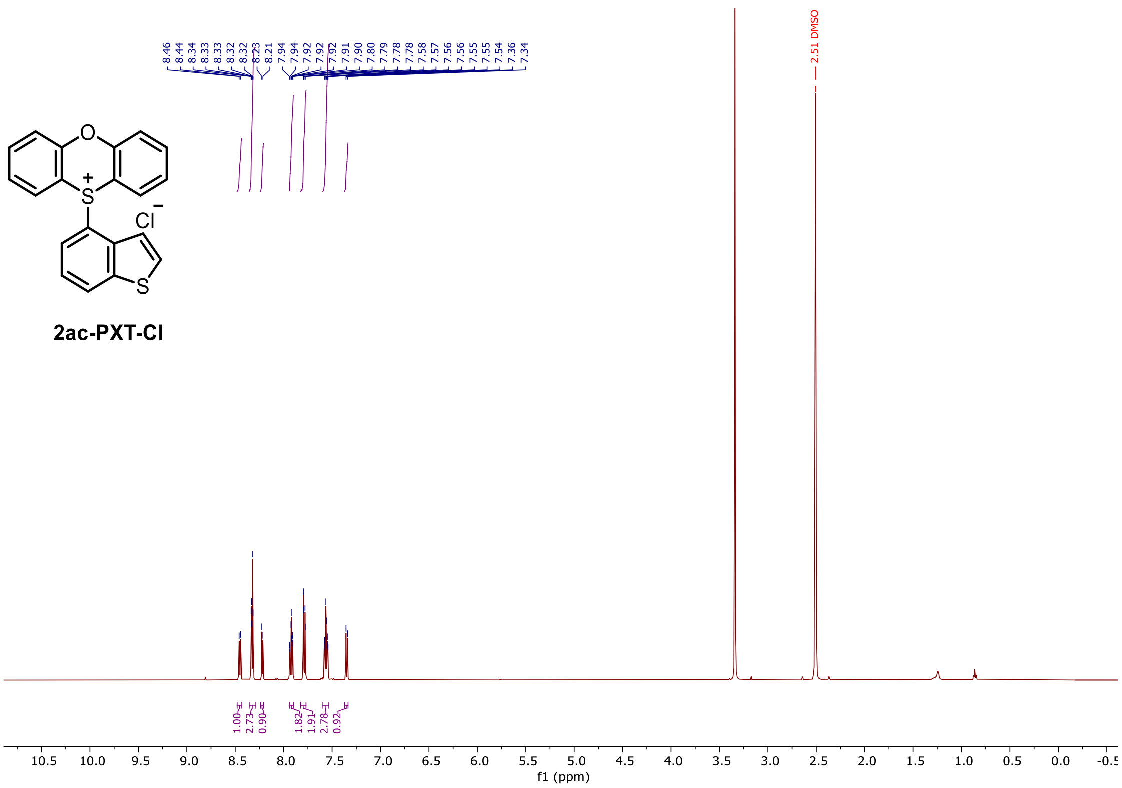

^{13}**C NMR** (101 MHz, DMSO-d$_6$) **2ac-PXT-Cl**

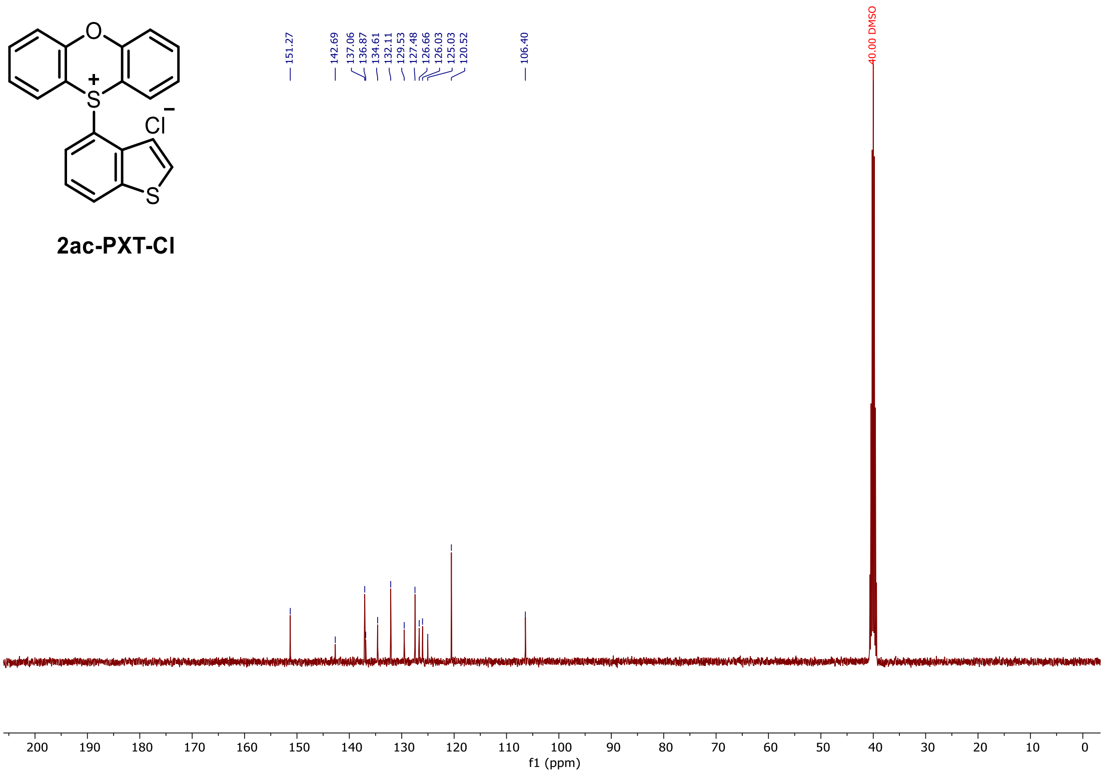

^{1}H NMR (400 MHz, DMSO-d$_6$) **2ad-DBT-Cl**

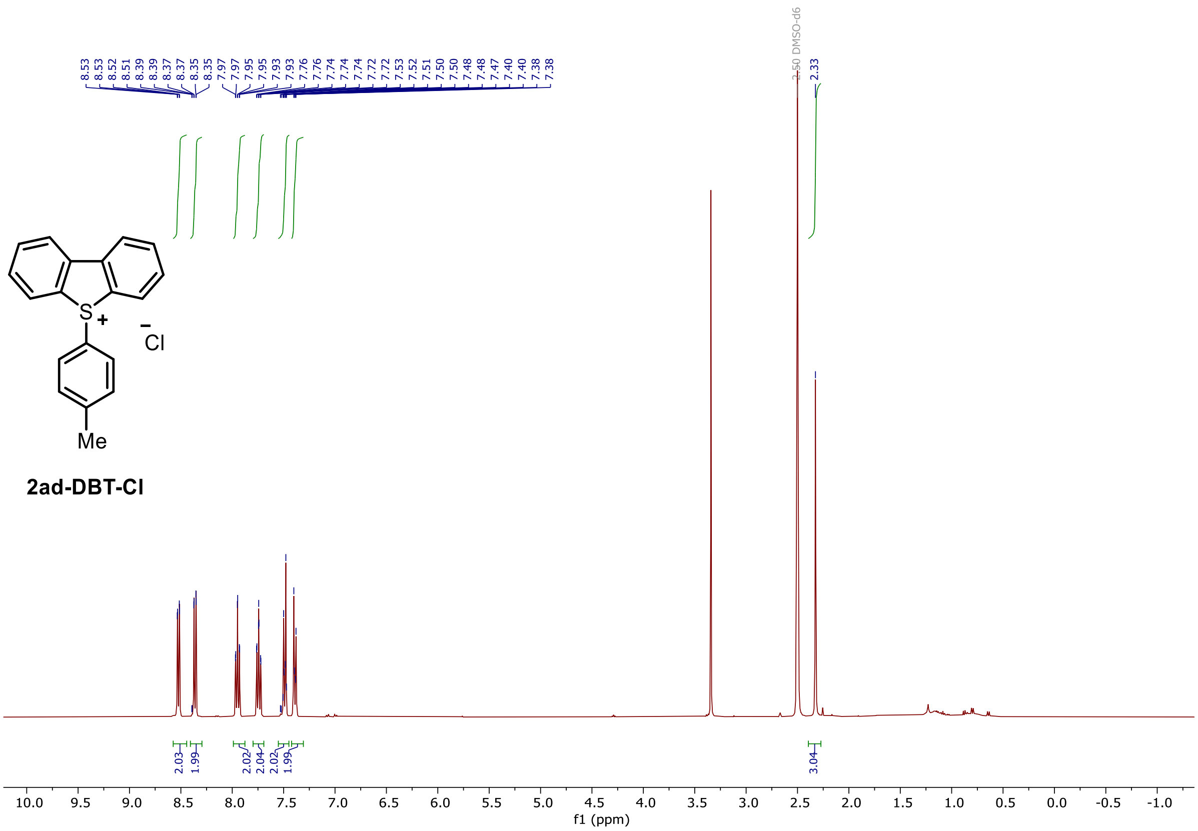

^{13}C NMR (101 MHz, DMSO-d$_6$) **2ad-DBT-Cl**

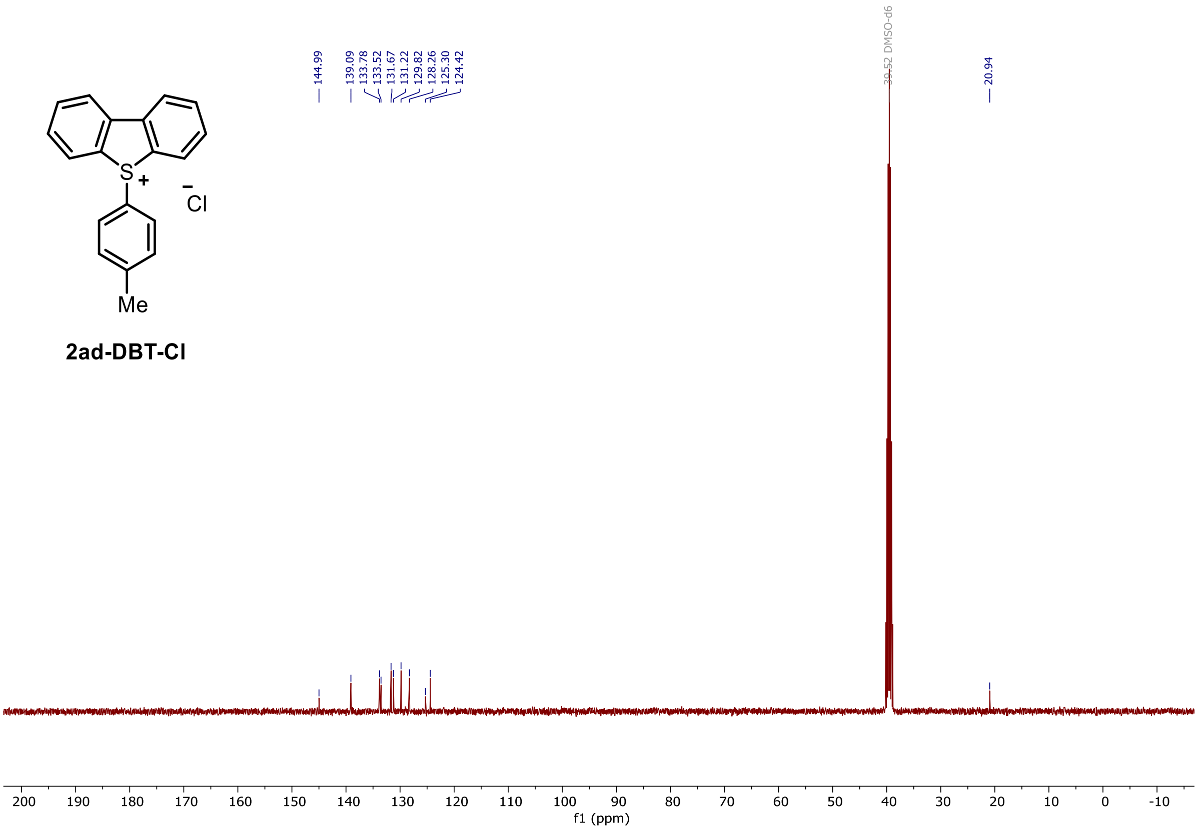

^{1}H NMR (500 MHz, DMSO-d$_6$) **2ae-DBT-Cl**

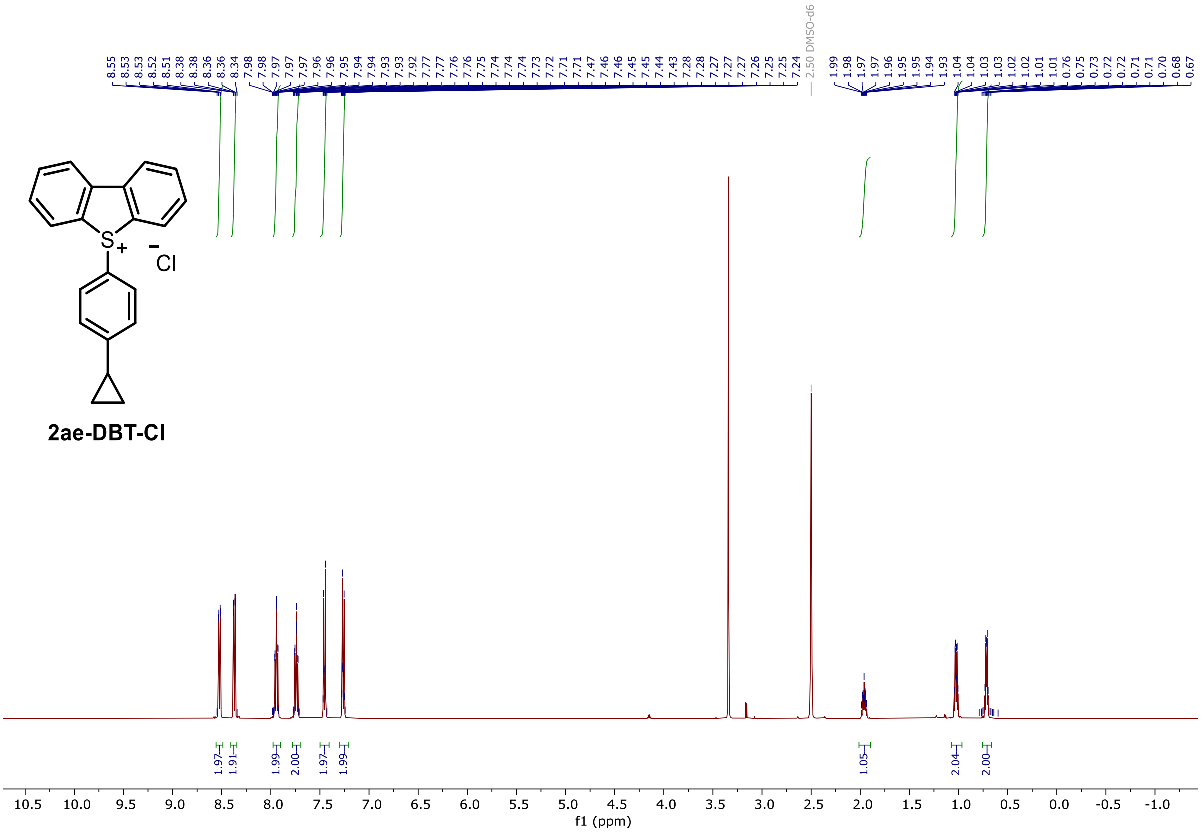

^{13}C NMR (126 MHz, DMSO-d$_6$) of **2ae-DBT-Cl**

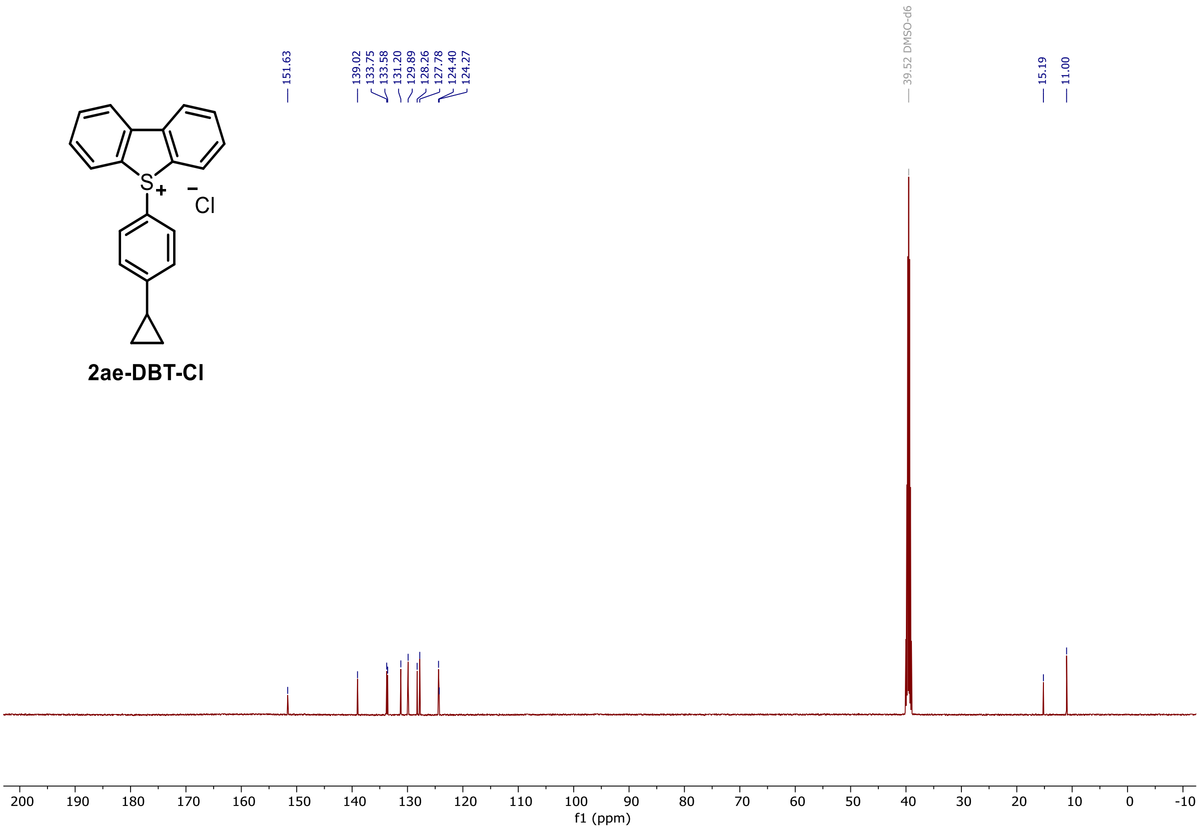

^{1}H NMR (400 MHz CDCl$_3$) of **3a**

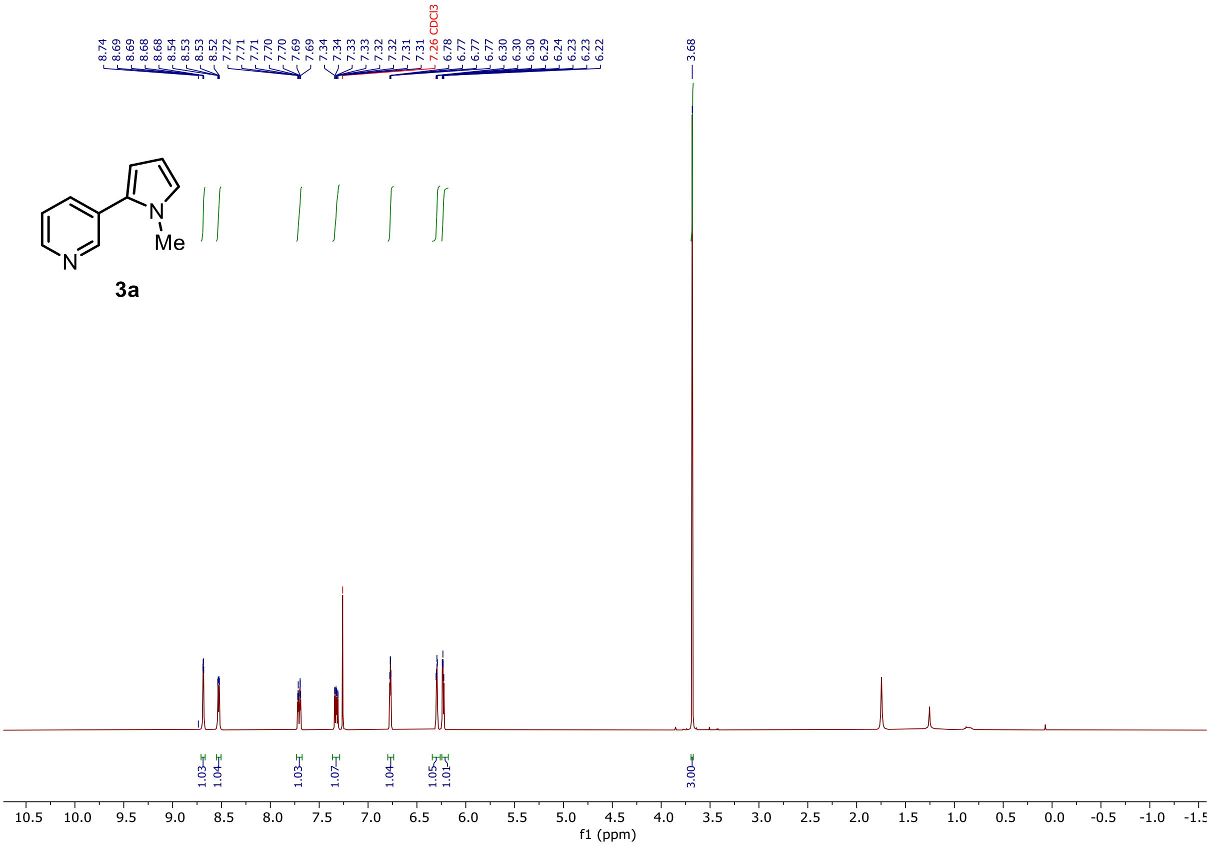

^{13}C NMR (101 MHz CDCl$_3$) of **3a**

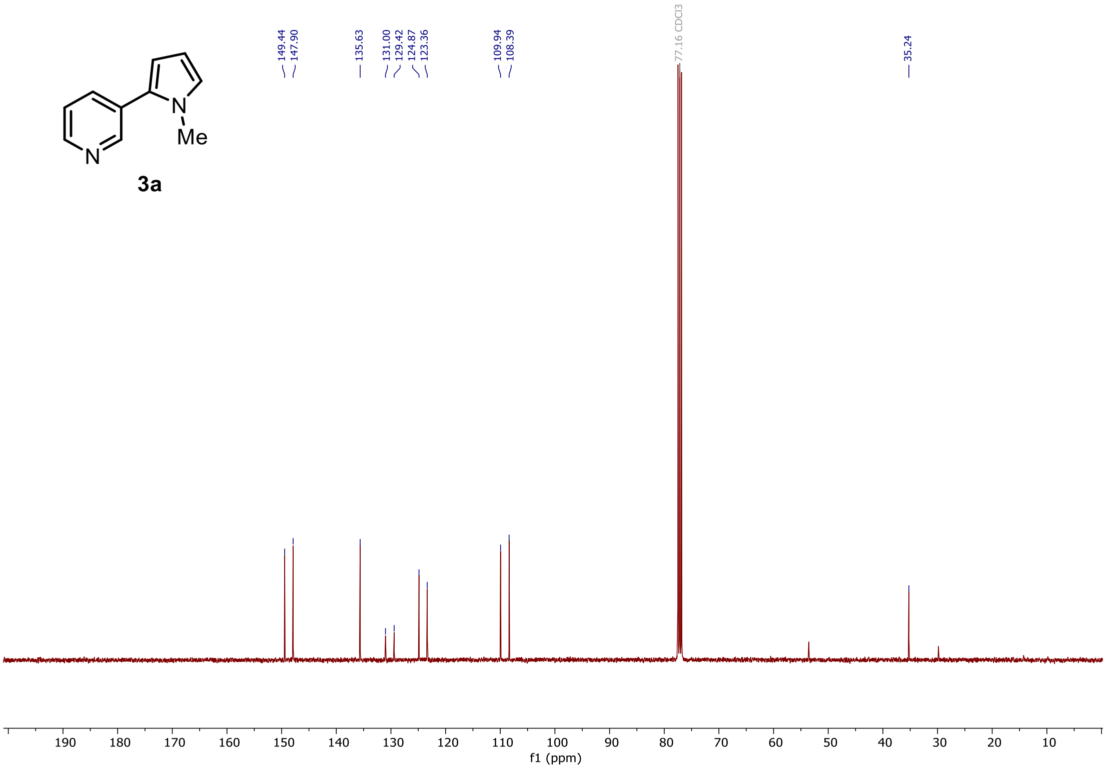

^{1}H NMR (400 MHz, $CDCl_3$) of **3b**

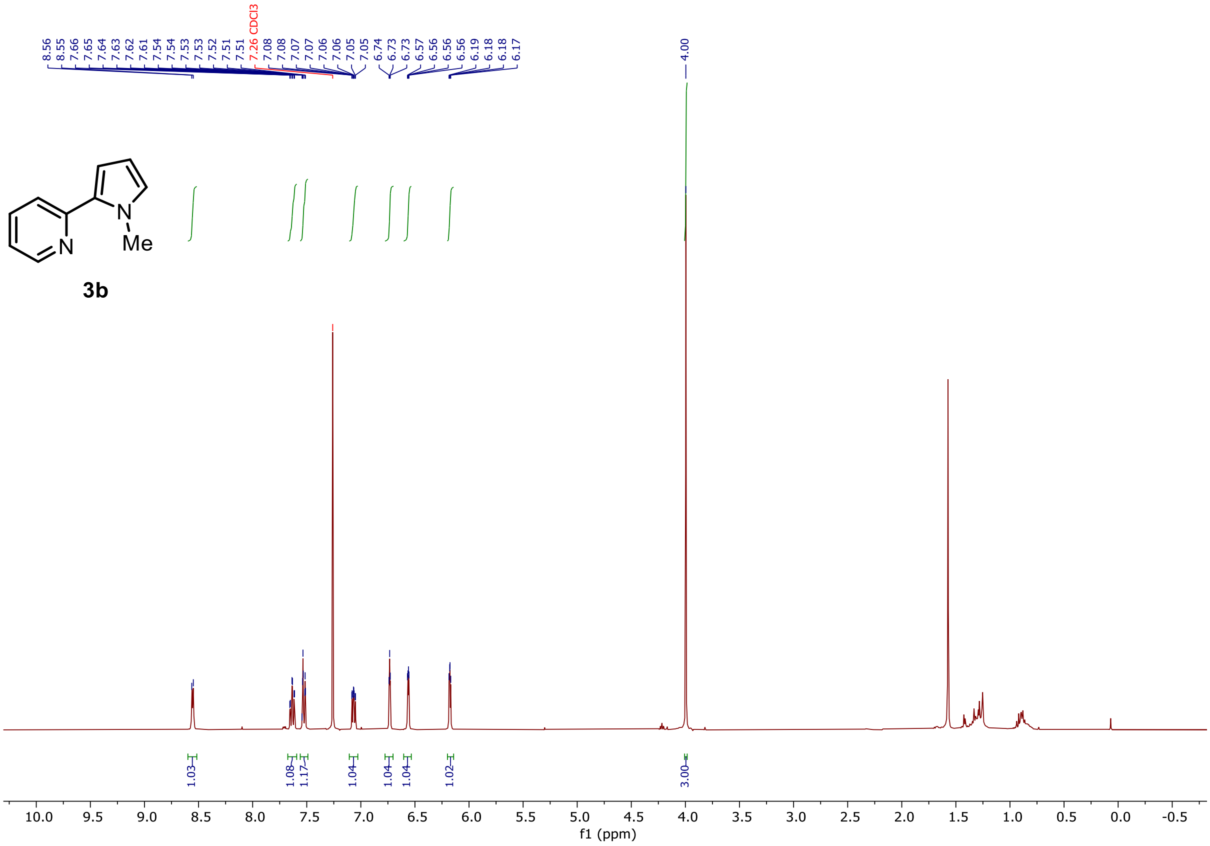

^{13}C NMR (101 MHz, $CDCl_3$) of **3b**

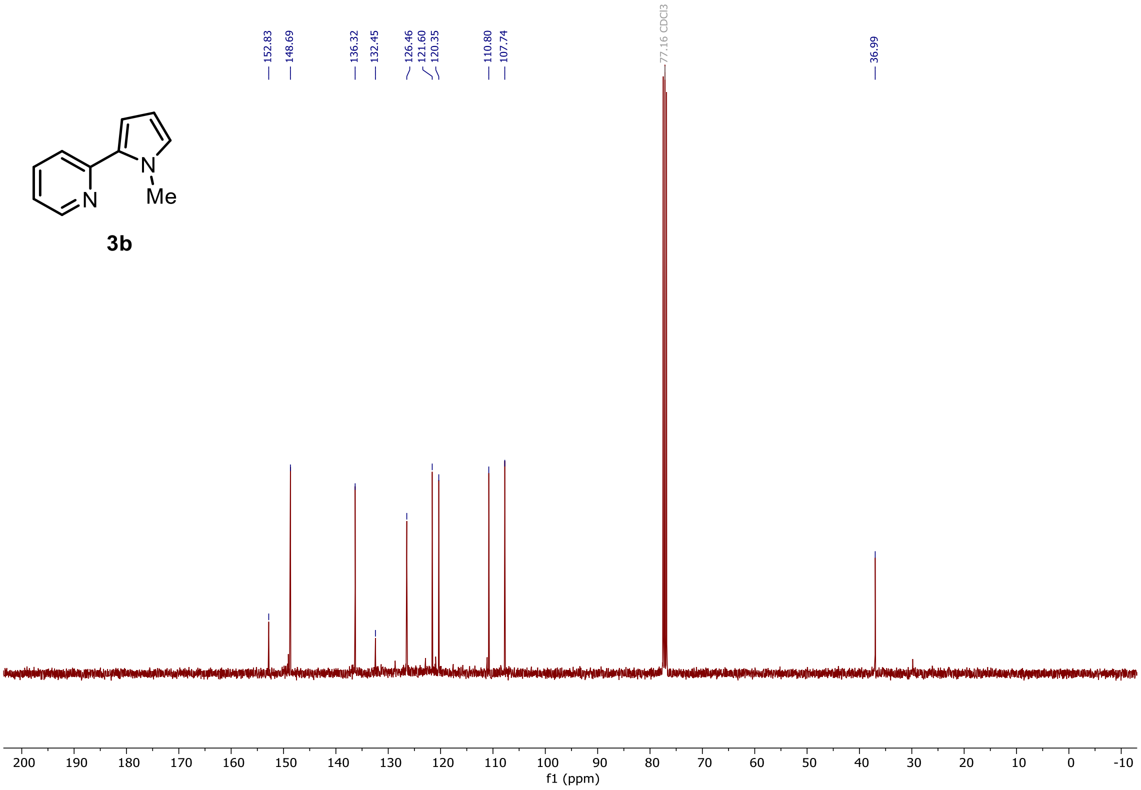

^{1}H NMR (400 MHz, $CDCl_3$) of **3c**

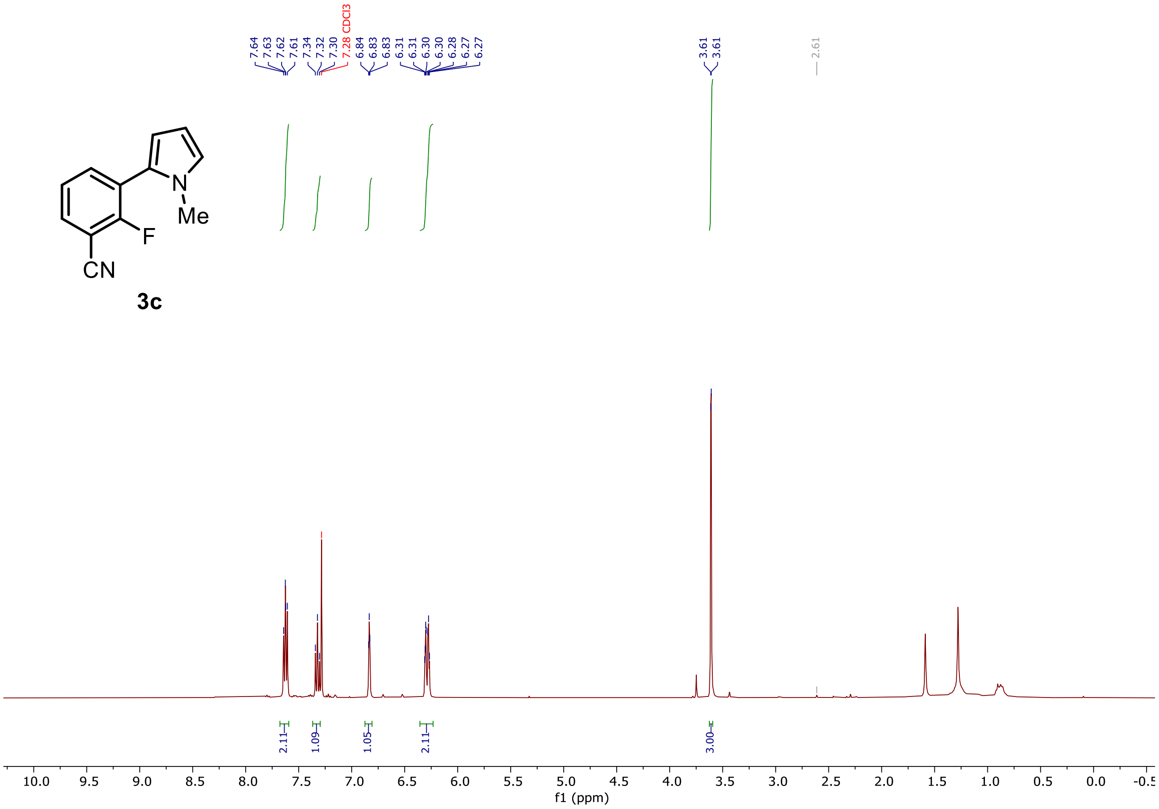

^{13}C NMR (101 MHz, $CDCl_3$) of **3c**

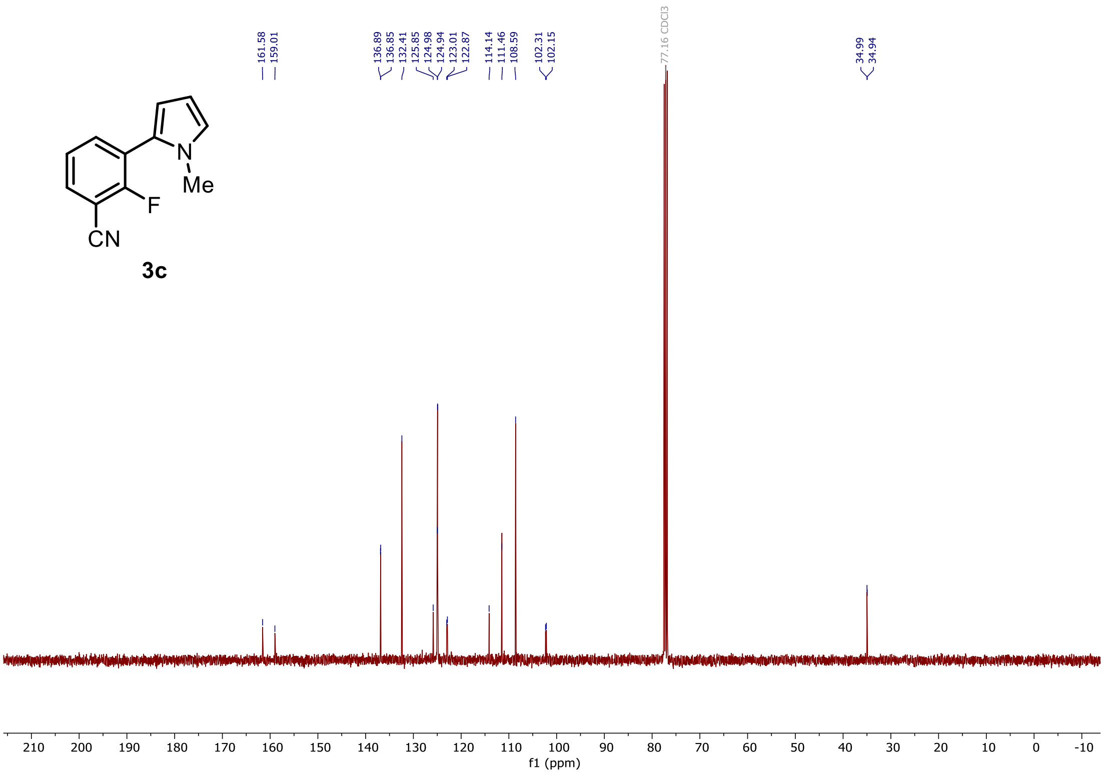

^{19}F NMR (376 MHz, DMSO-d$_6$) of **3c**

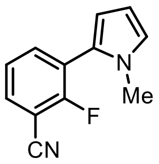

3c

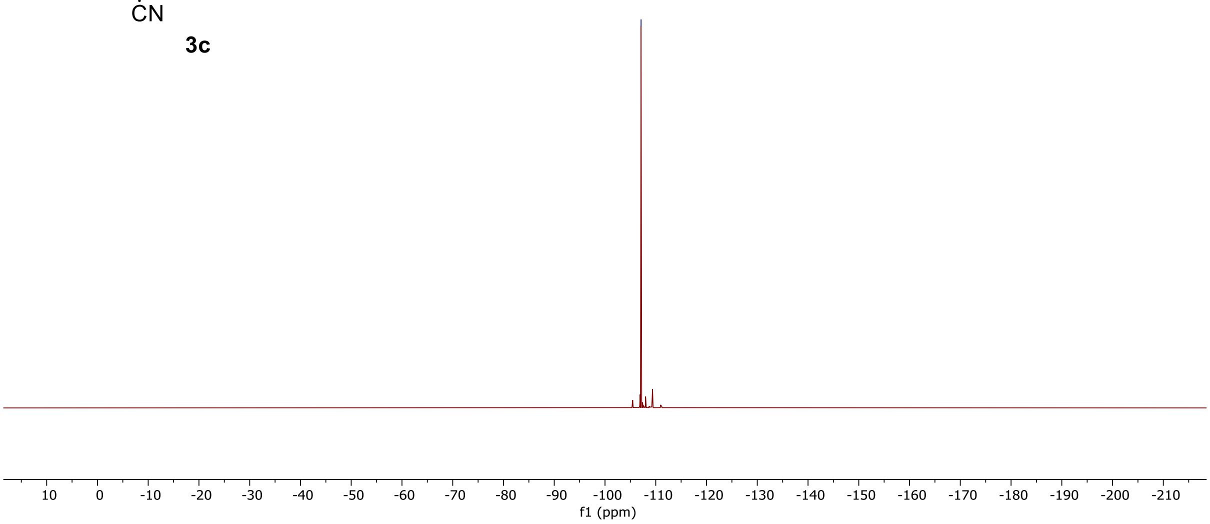

^{1}H NMR (400 MHz, $CDCl_3$) of **3d**

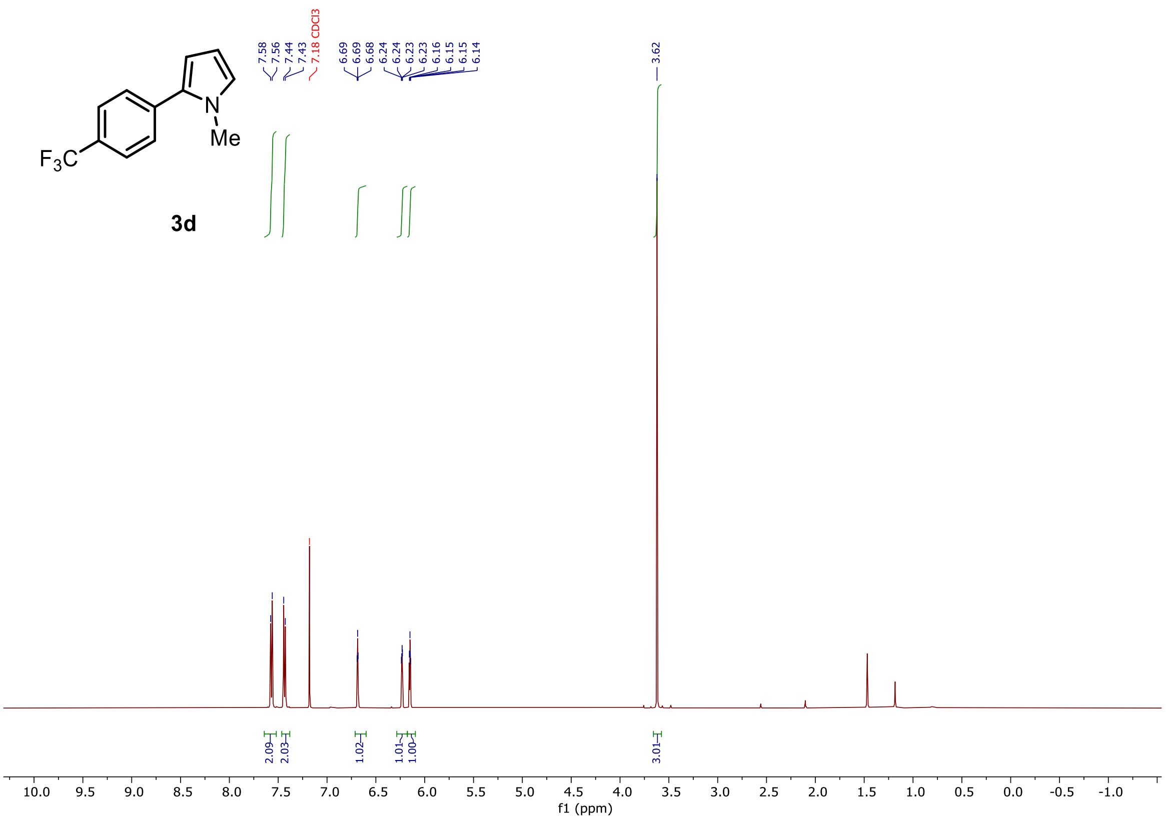

^{13}C NMR (126 MHz, $CDCl_3$) of **3d**

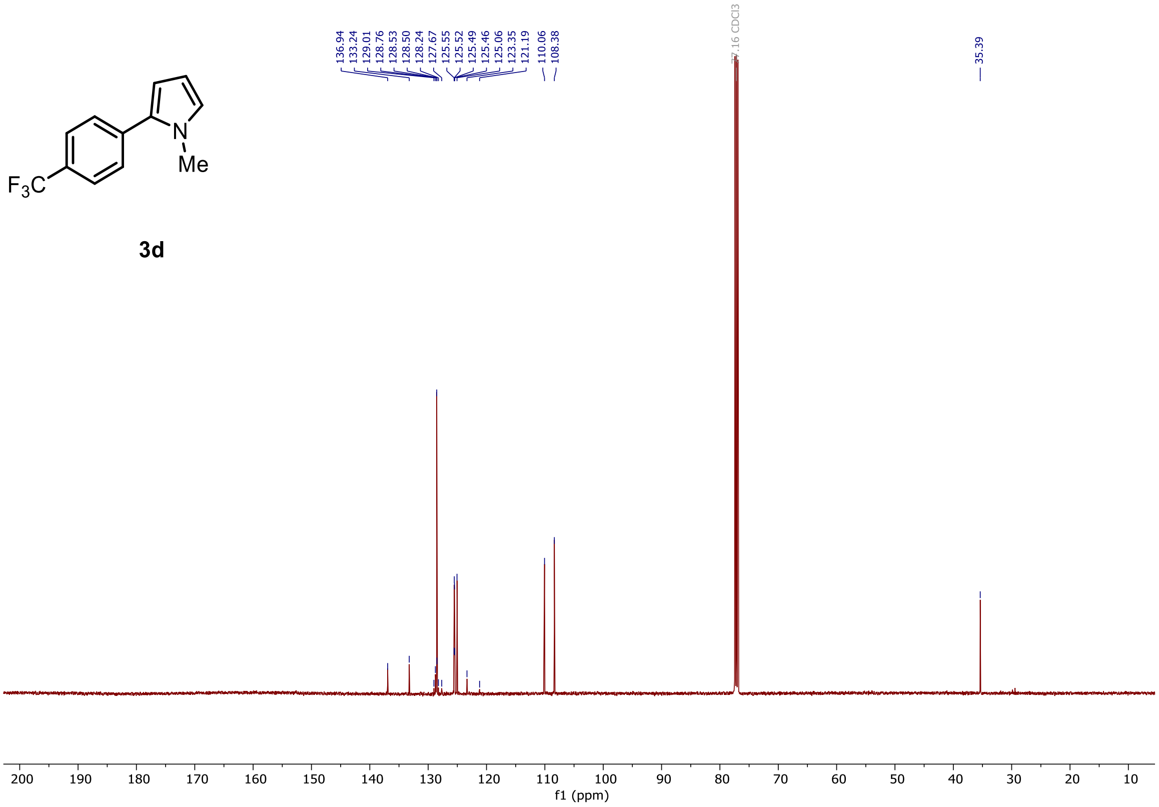

^{19}F NMR (376 MHz, DMSO-d$_6$) of **3d**

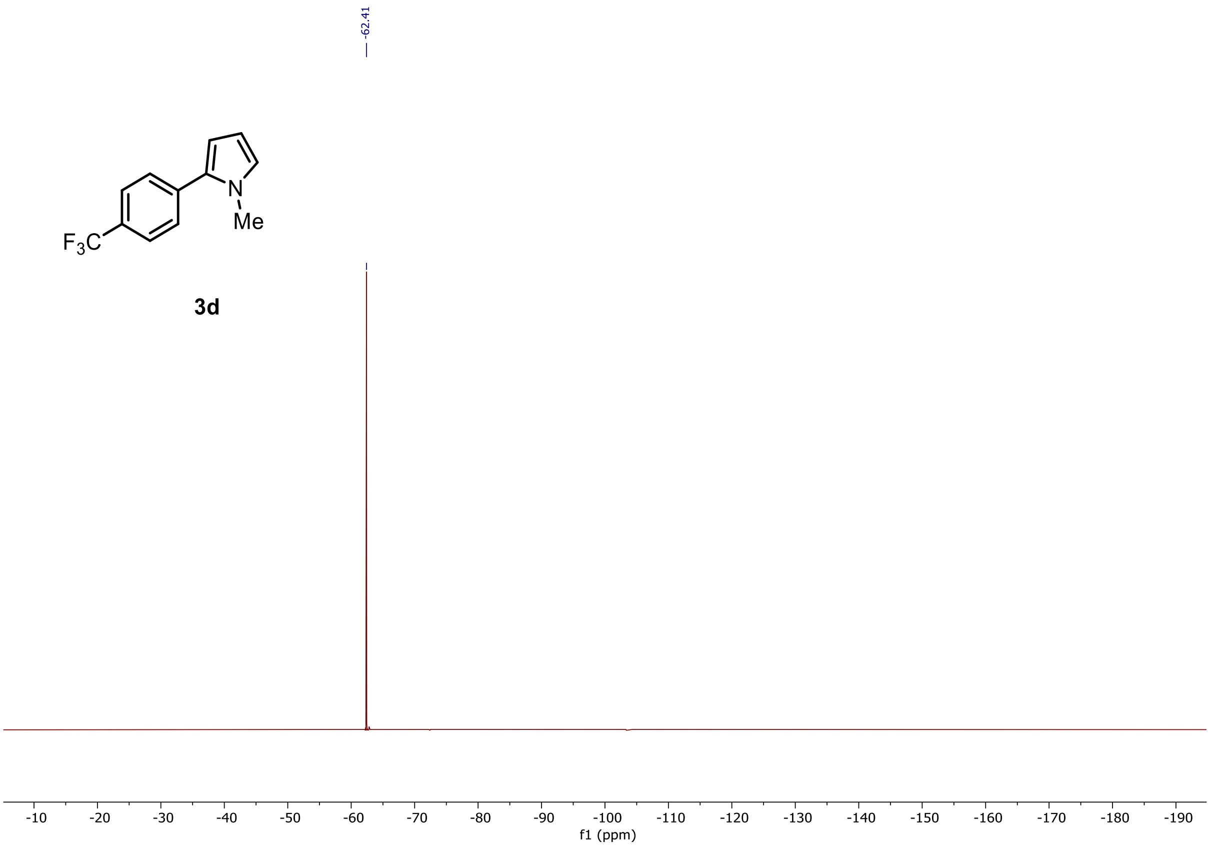

^{1}H NMR (500 MHz, $CDCl_3$) of **3e**

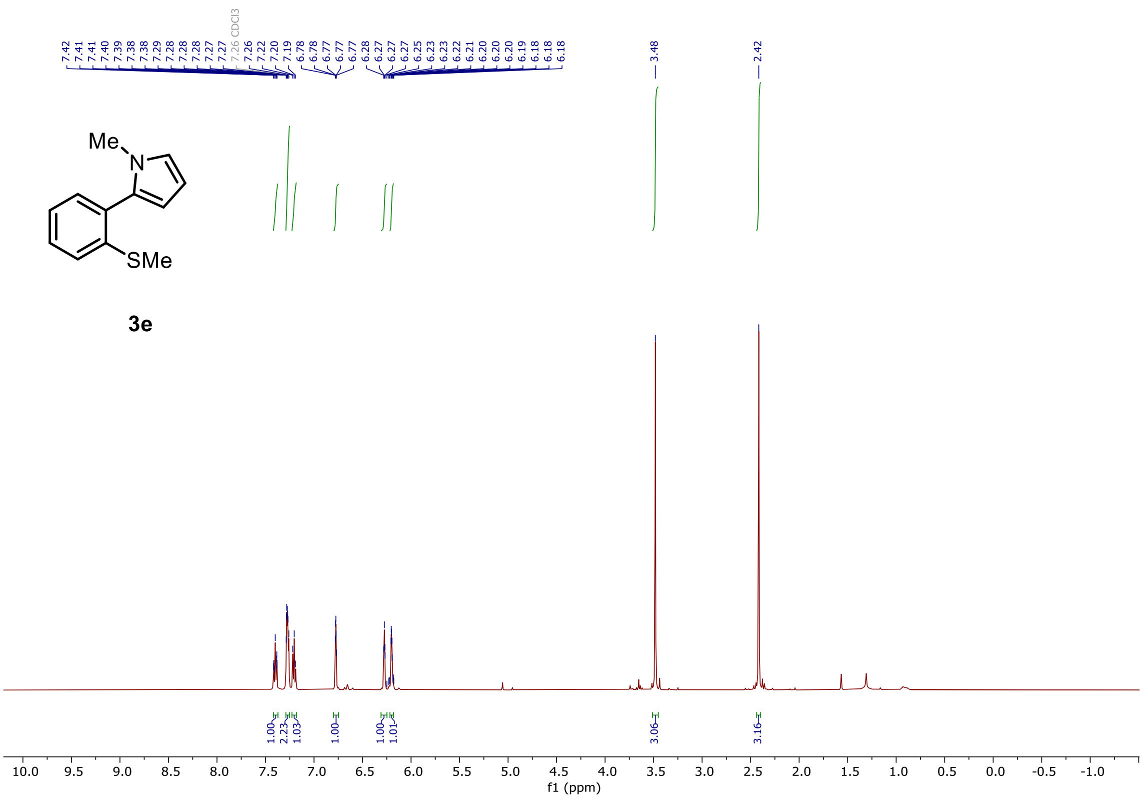

^{13}C NMR (126 MHz, $CDCl_3$) of **3e**

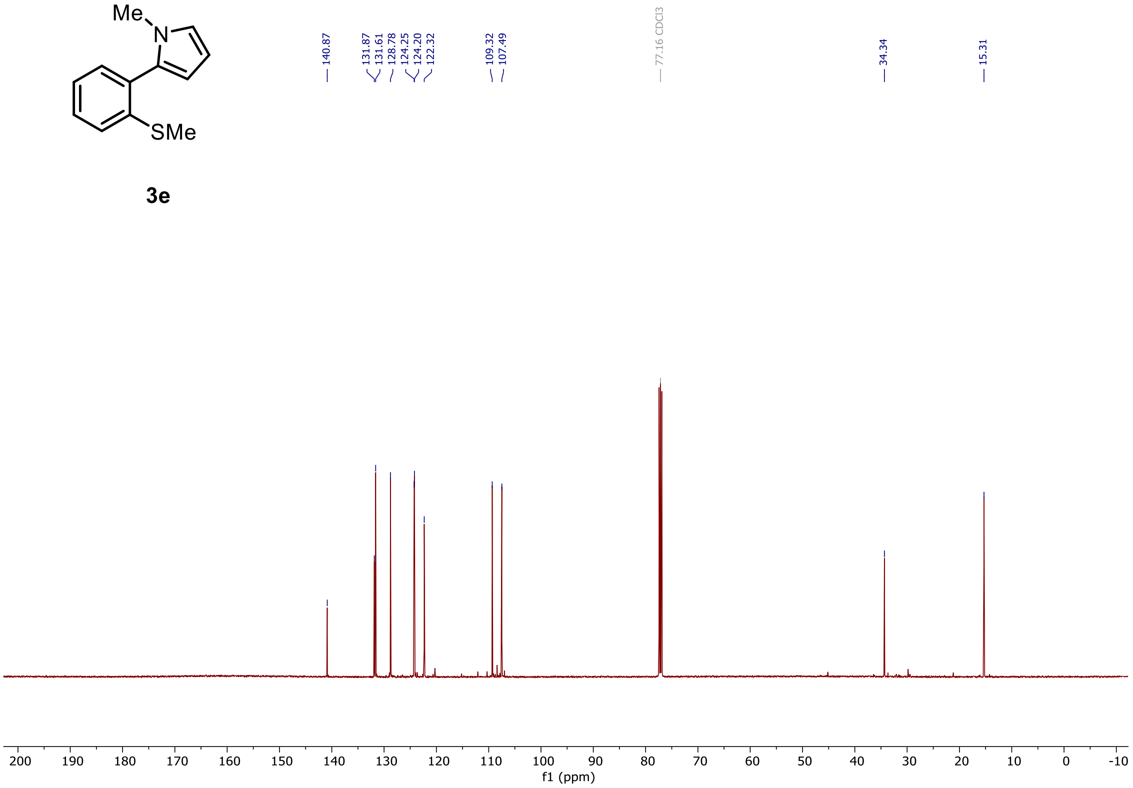

^{1}H NMR (400 MHz, $CDCl_3$) of **3f**

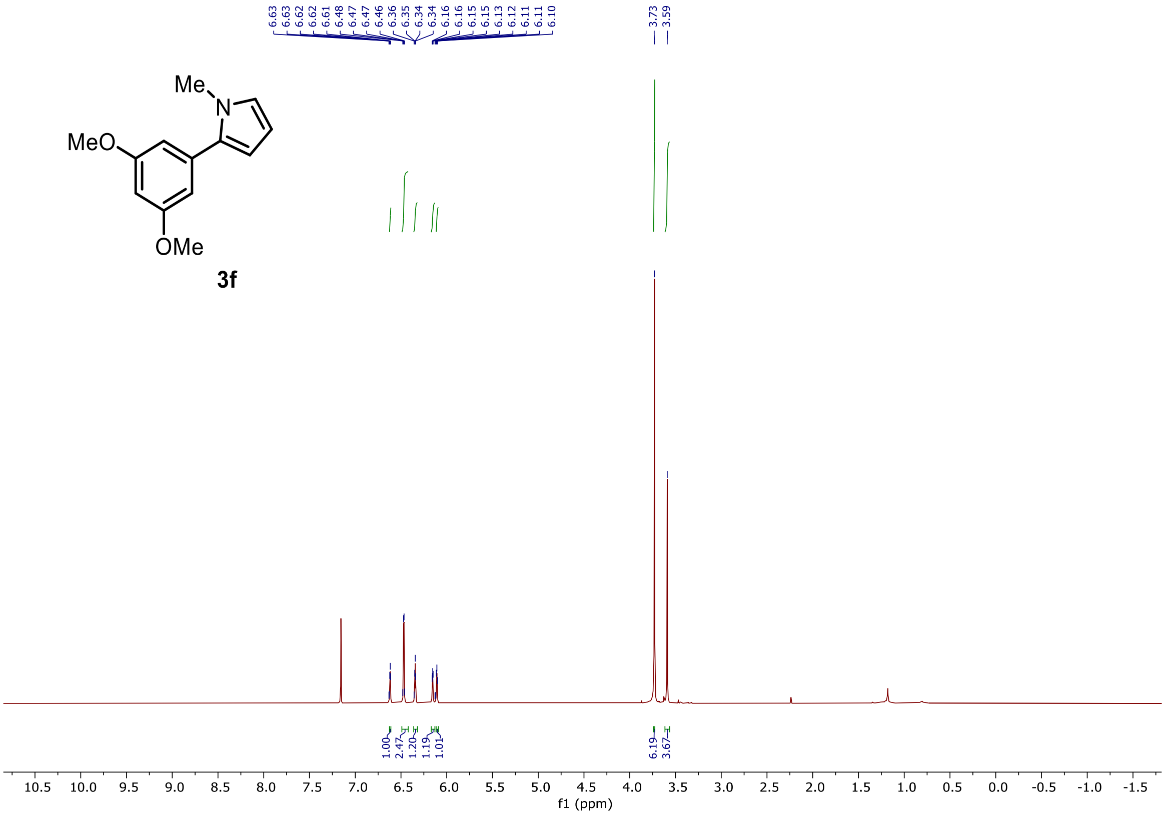

^{13}C NMR (101 MHz, $CDCl_3$) of **3f**

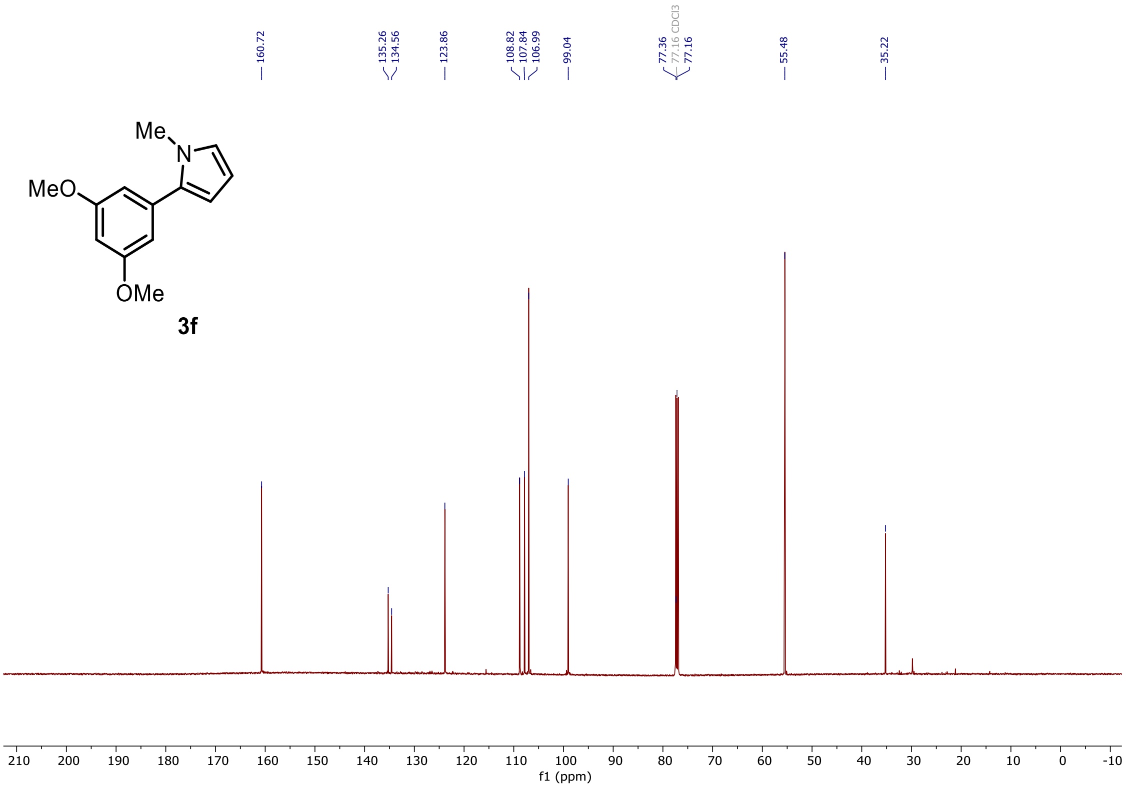

^{1}H NMR (400 MHz, $CDCl_3$) of **4a**

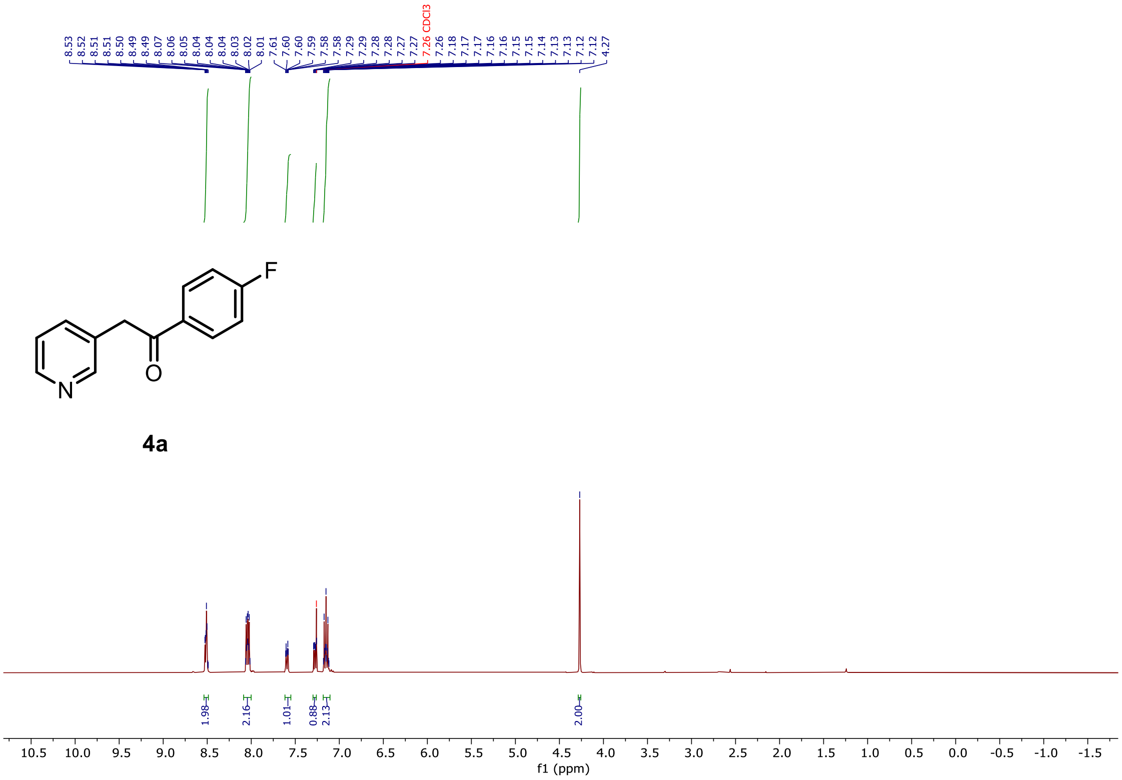

^{13}C NMR (101 MHz, $CDCl_3$) of **4a**

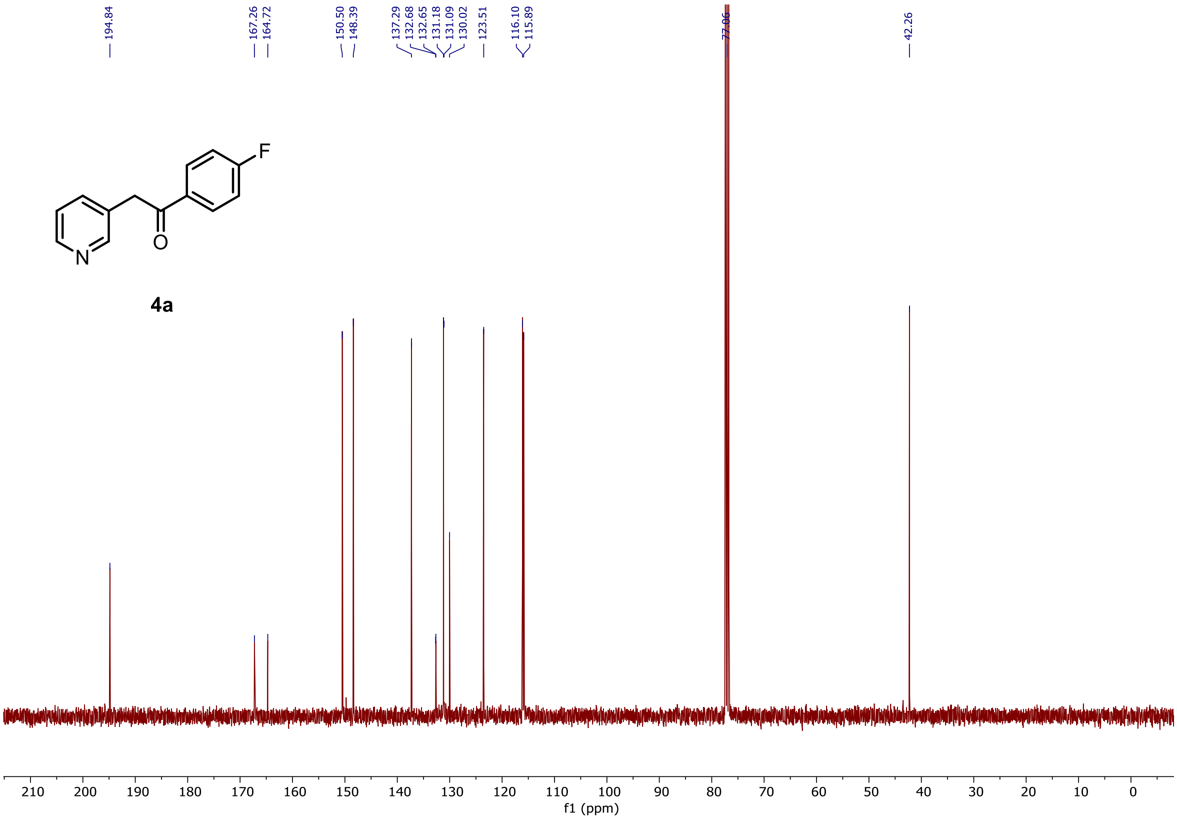

^{19}F NMR (376 MHz, $CDCl_3$) of **4a**

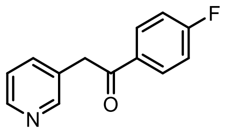

4a

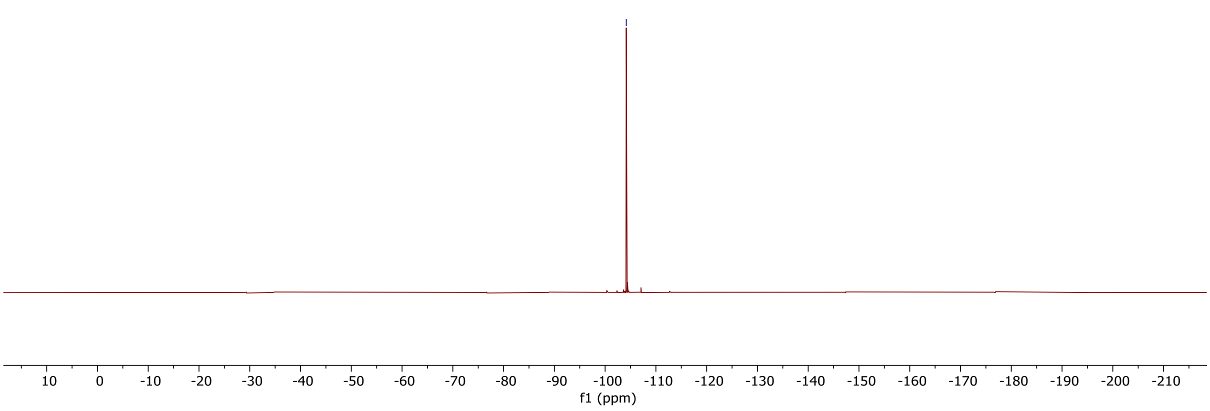

^{1}H NMR (400 MHz, $CDCl_3$) of **4b**

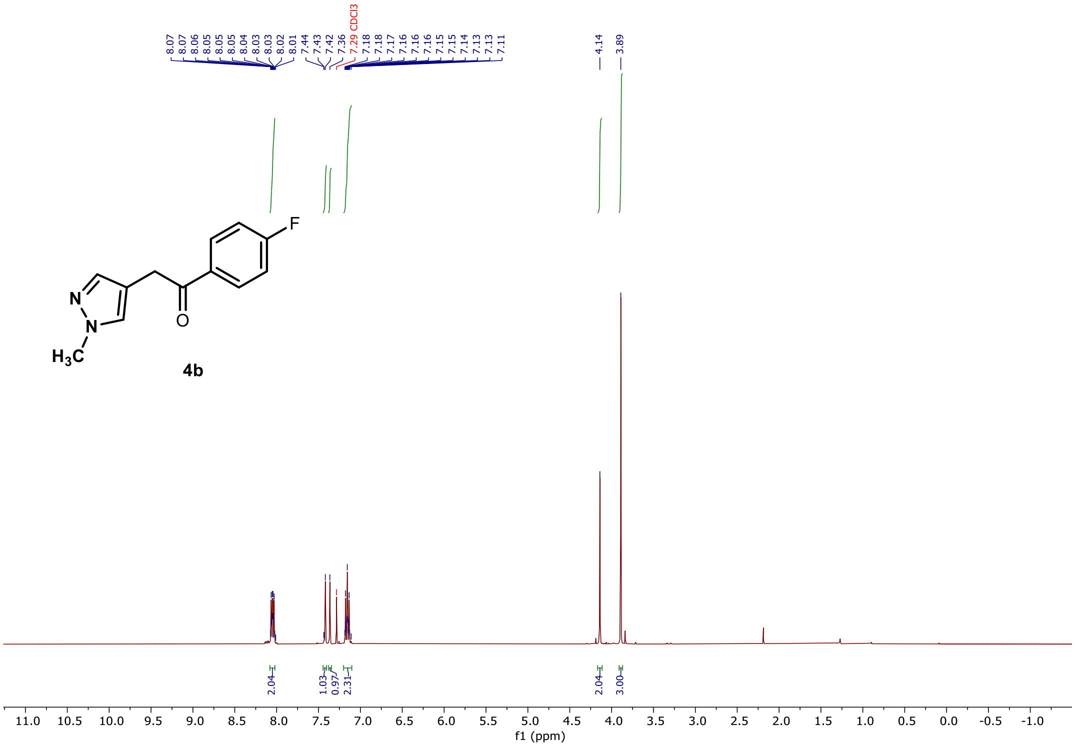

^{13}C NMR (101 MHz, $CDCl_3$) of **4b**

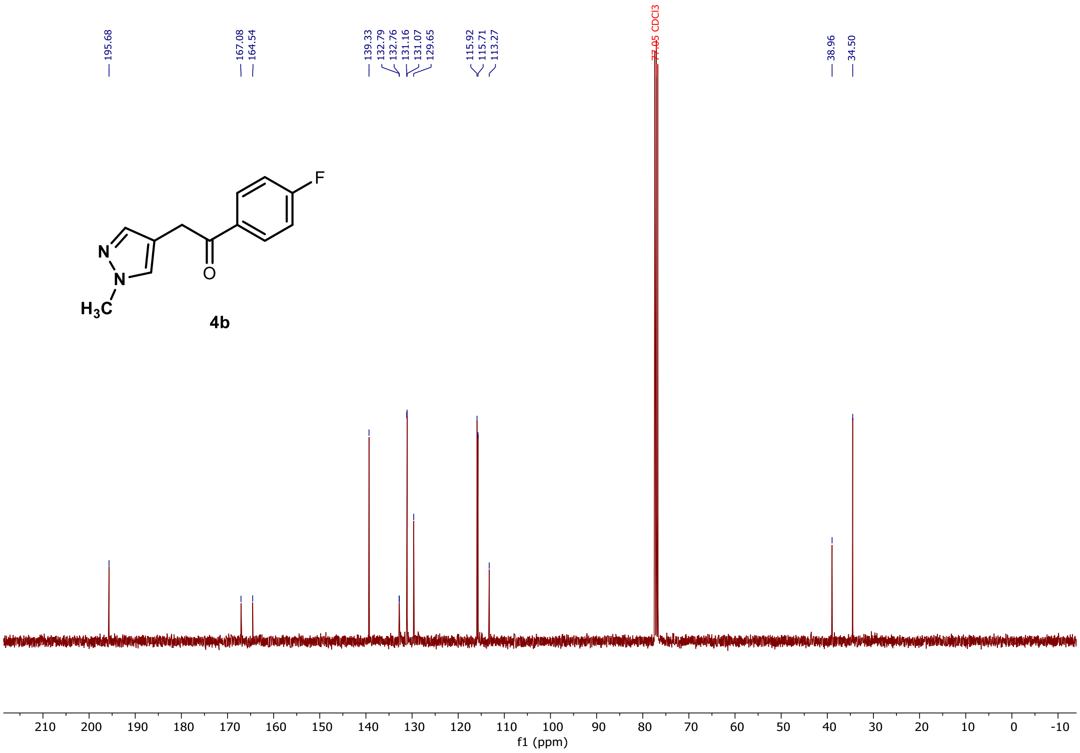

^{19}F NMR (471 MHz, $CDCl_3$) of **4b**

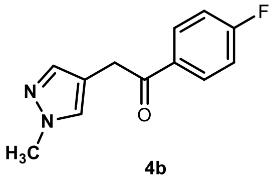

4b

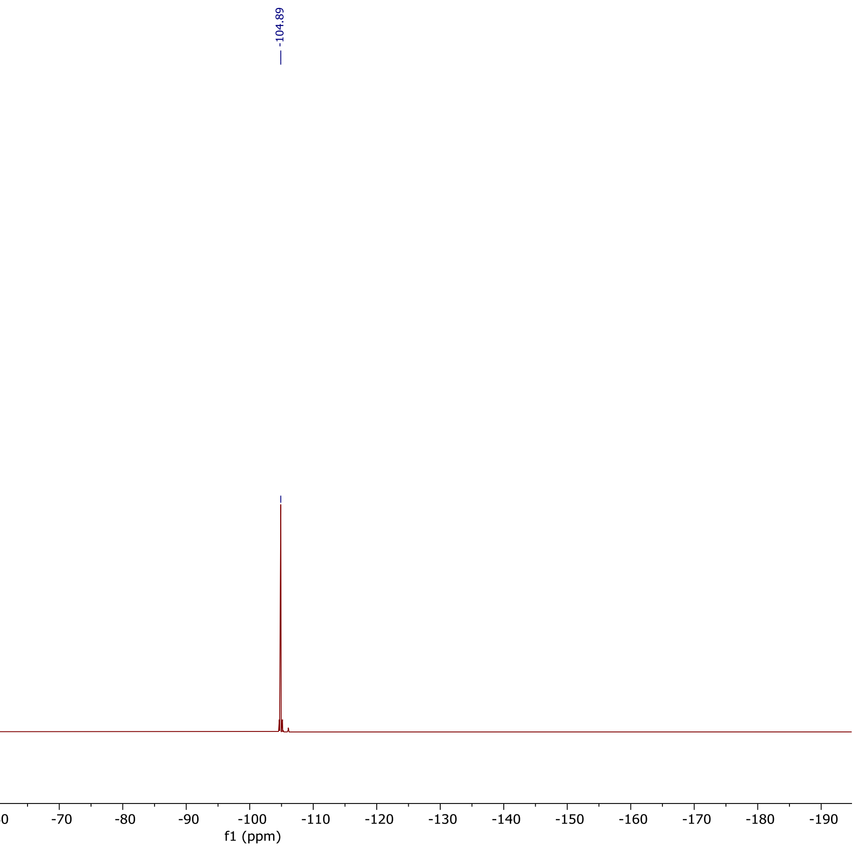

^{1}H NMR (400 MHz, $CDCl_3$) of **4c**

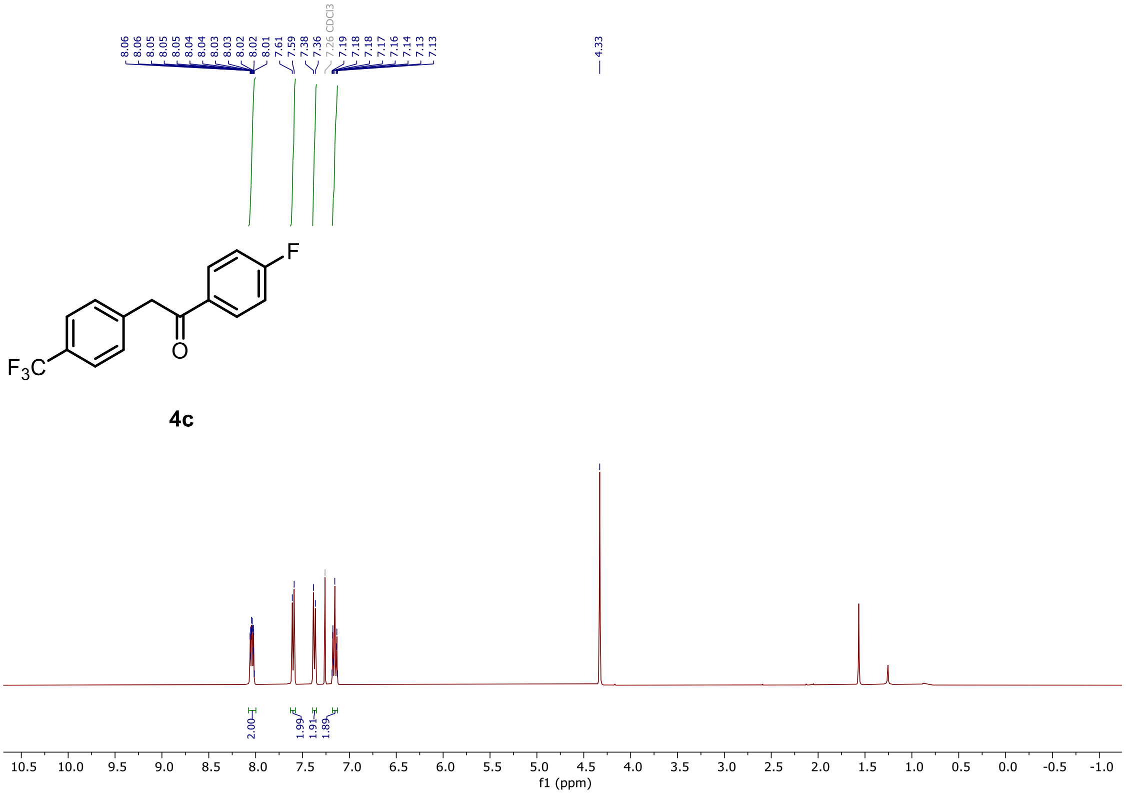

^{13}C NMR (101 MHz, $CDCl_3$) of **4c**

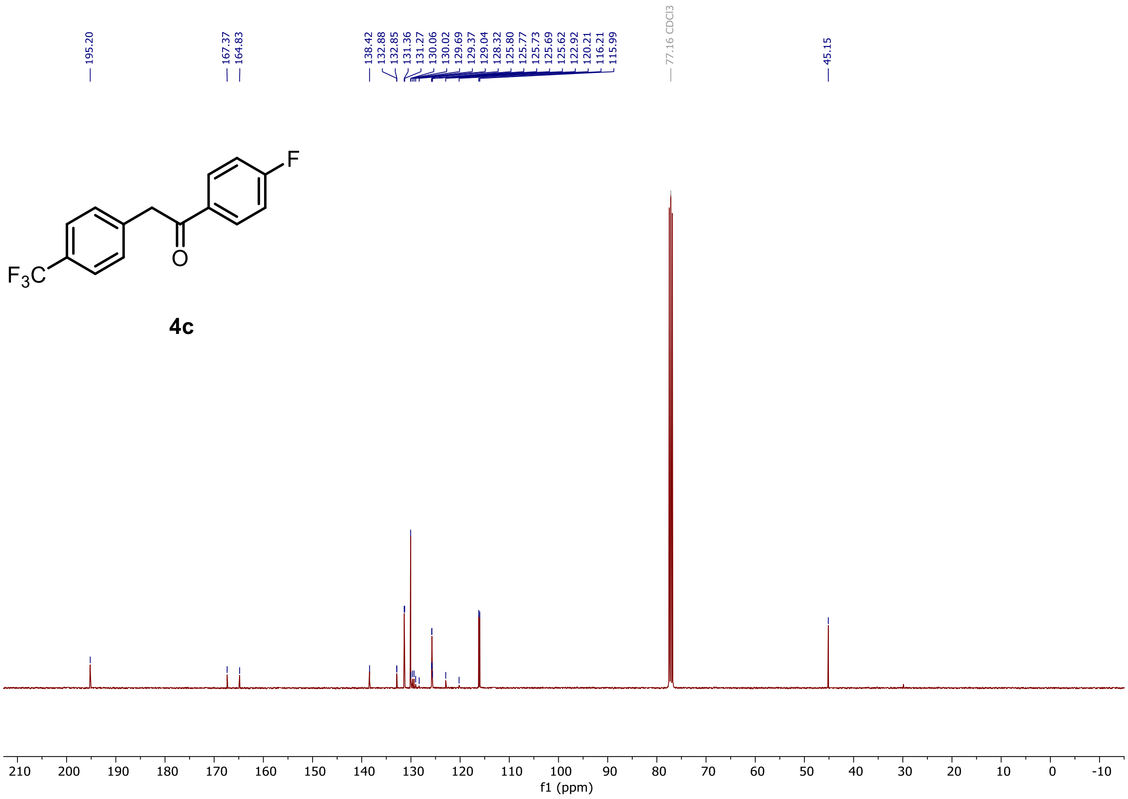

^{19}F NMR (376 MHz, $CDCl_3$) of **4c**

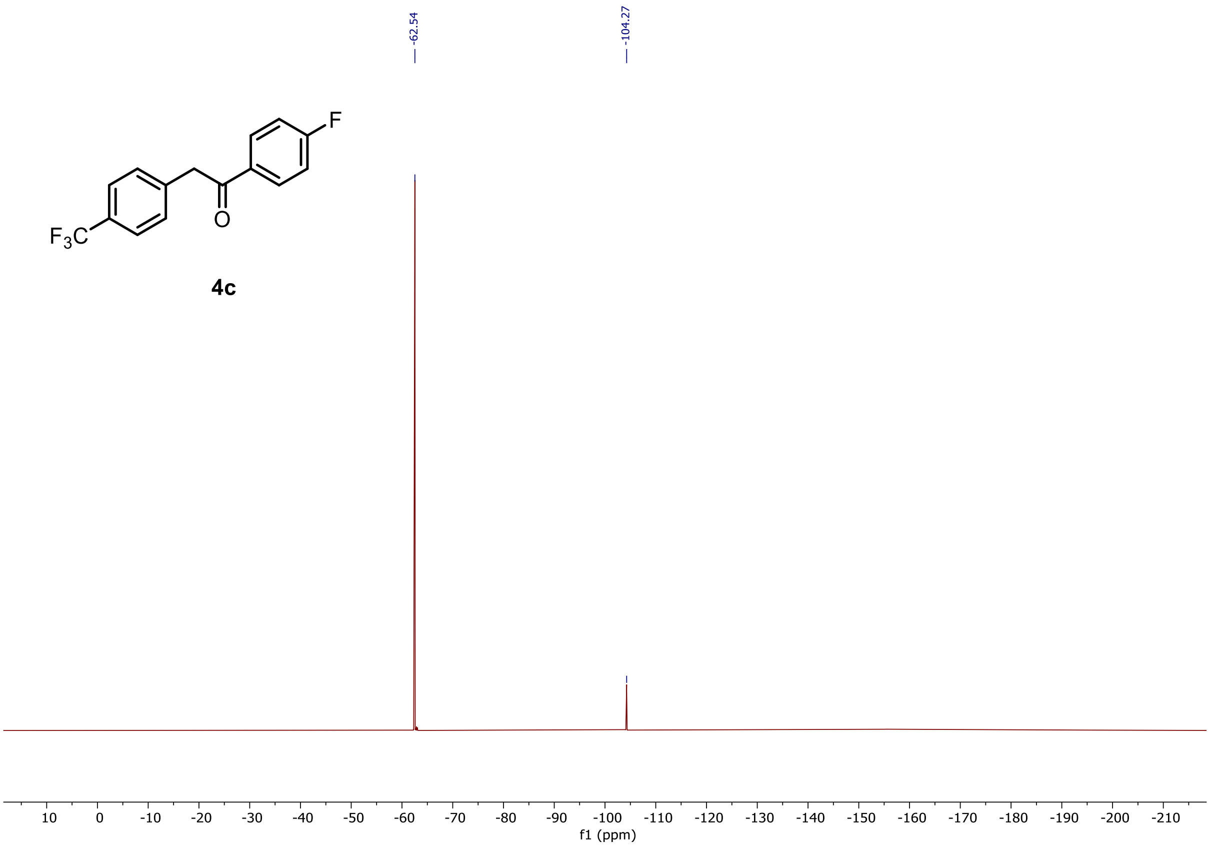

^{1}H NMR (400 MHz, $CDCl_3$) of **4d**

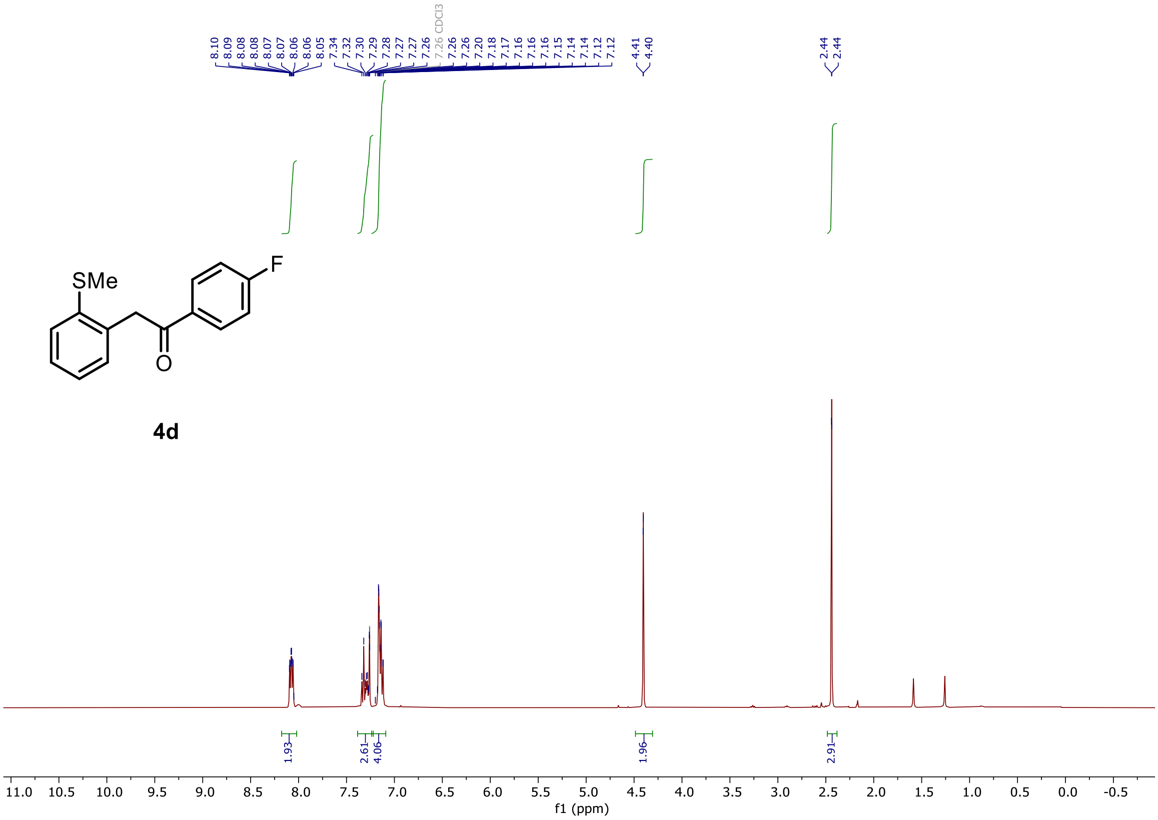

^{13}C NMR (101 MHz, $CDCl_3$) of **4d**

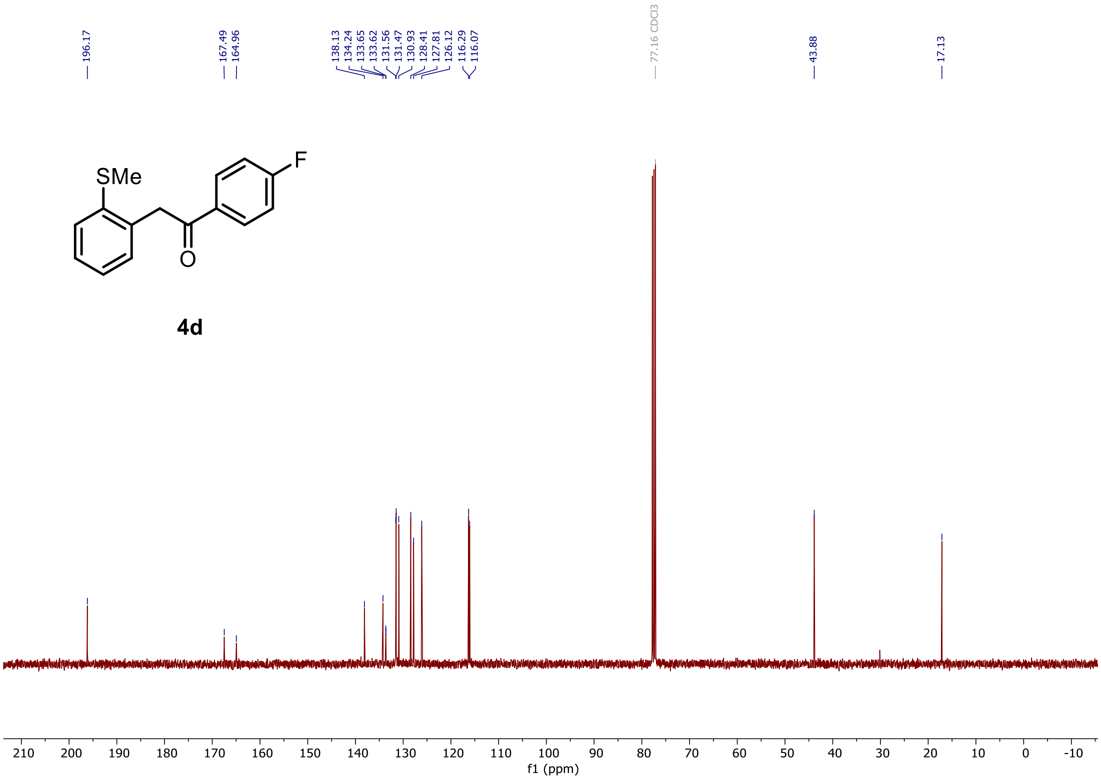

^{19}F NMR (376 MHz, $CDCl_3$) of **4d**

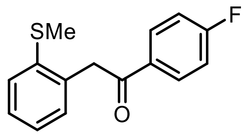

4d

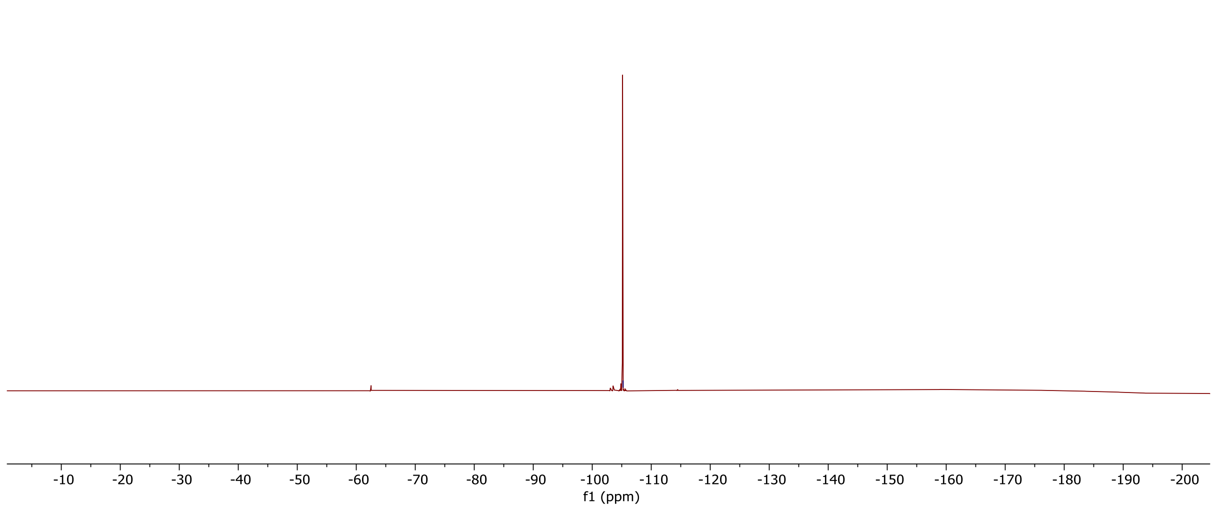

^{1}H NMR (400 MHz, $CDCl_3$) of **4e**

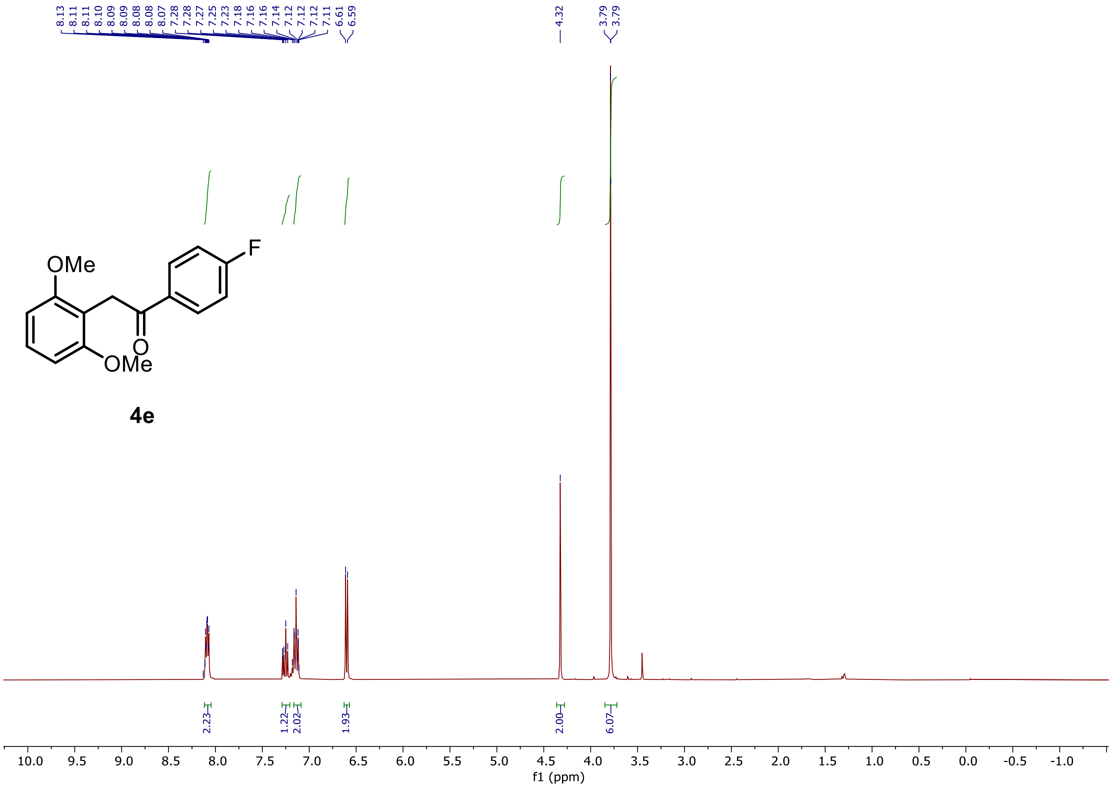

^{13}C NMR (101 MHz, $CDCl_3$) of **4e**

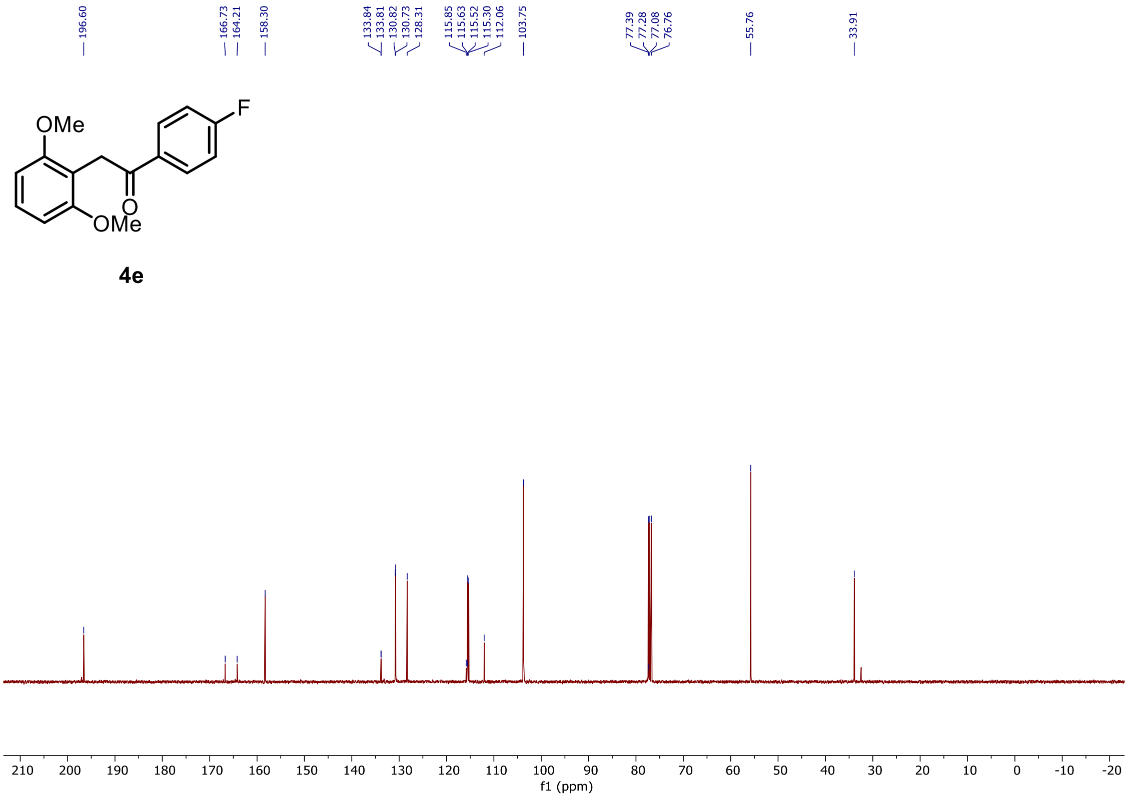

^{19}F NMR (376 MHz, $CDCl_3$) of **4e**

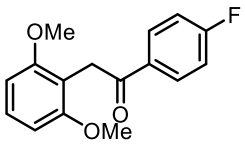

4e

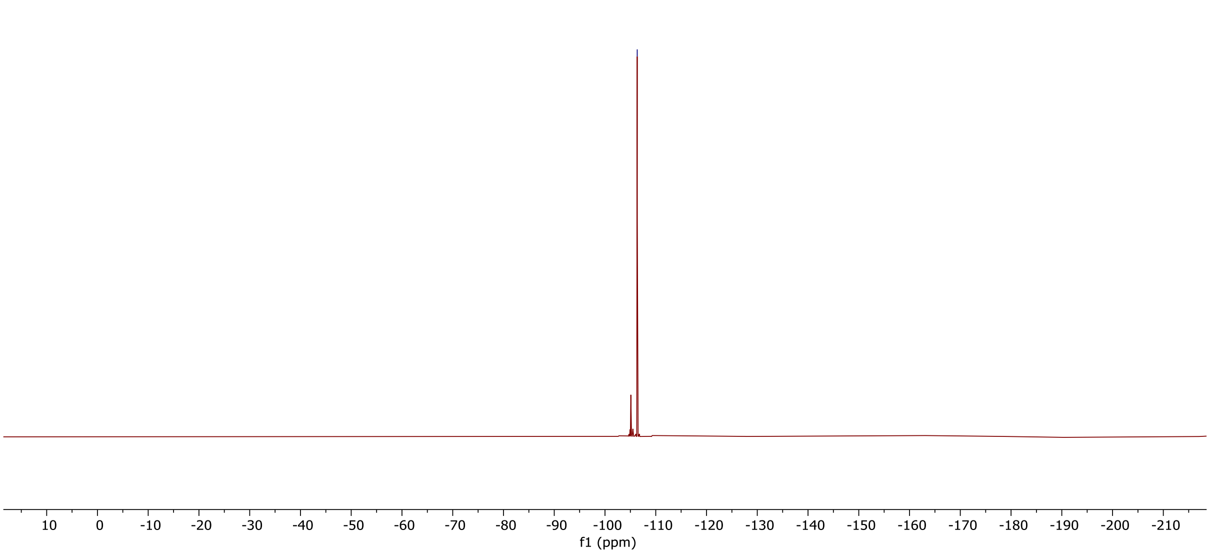

^{1}H NMR (500 MHz, $CDCl_3$) of **5a**

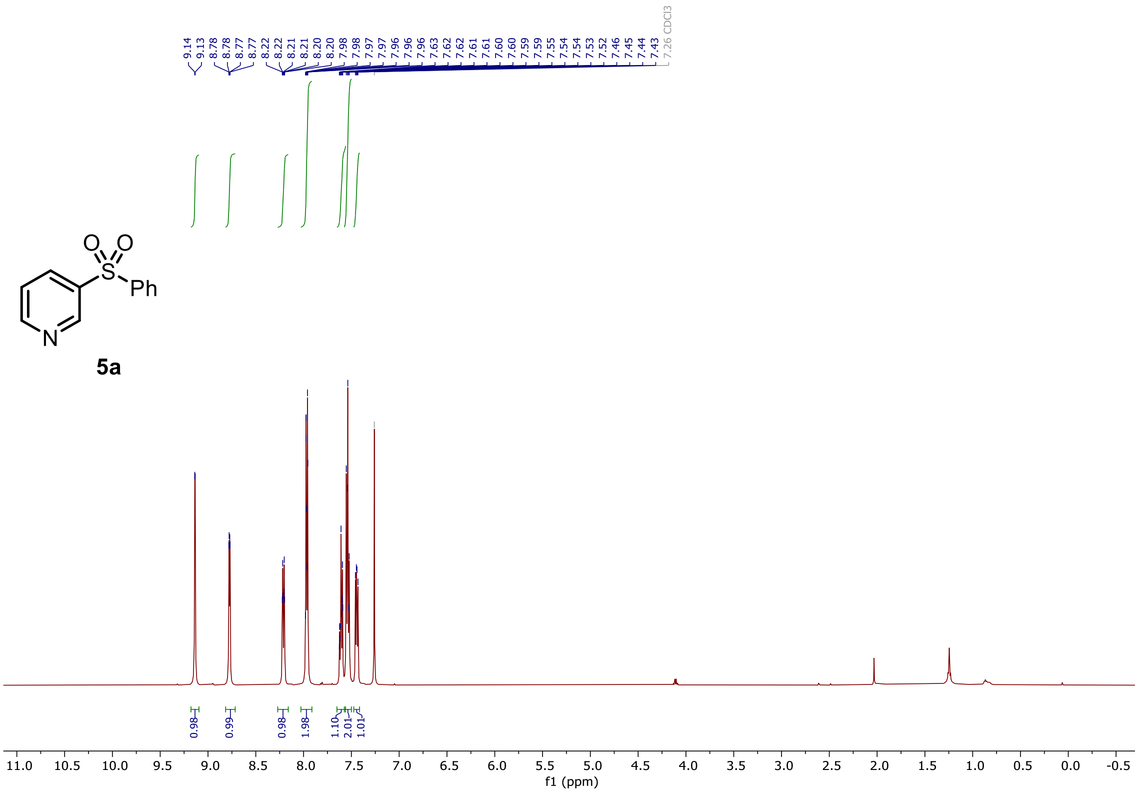

^{13}C NMR (126 MHz, $CDCl_3$) of **5a**

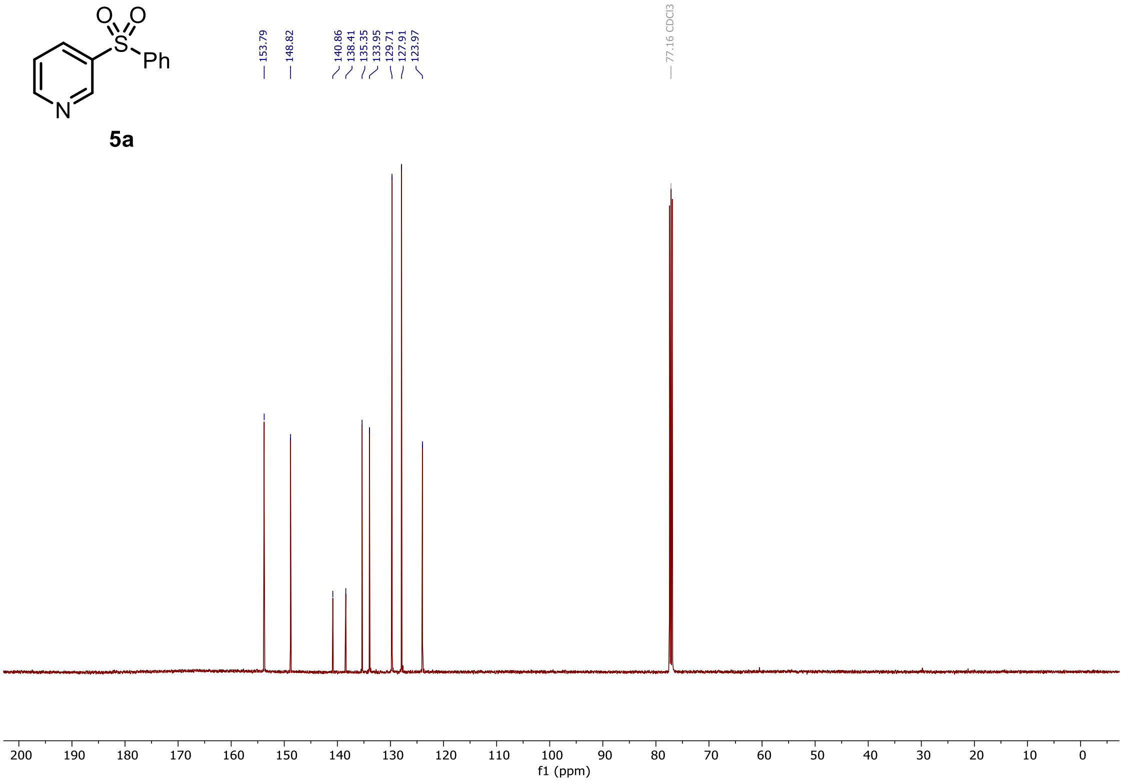

^{1}H NMR (500 MHz, $CDCl_3$) of **5b**

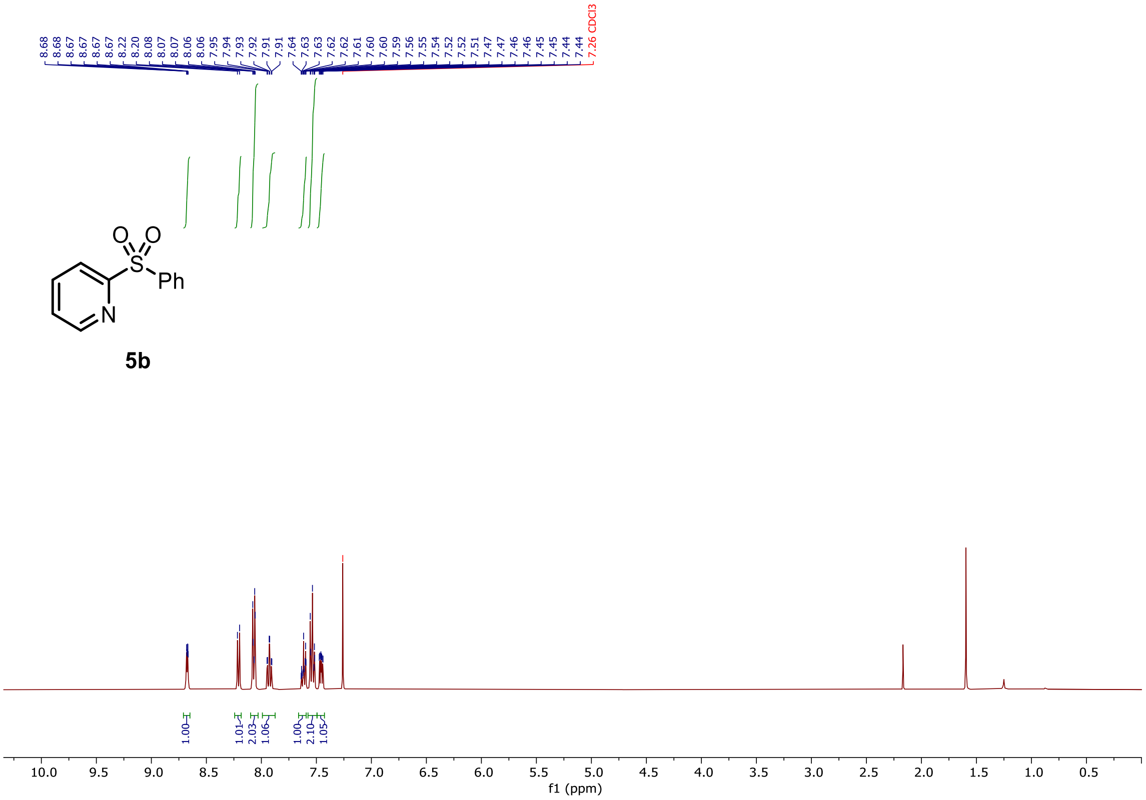

^{13}C NMR (126 MHz, $CDCl_3$) of **5b**

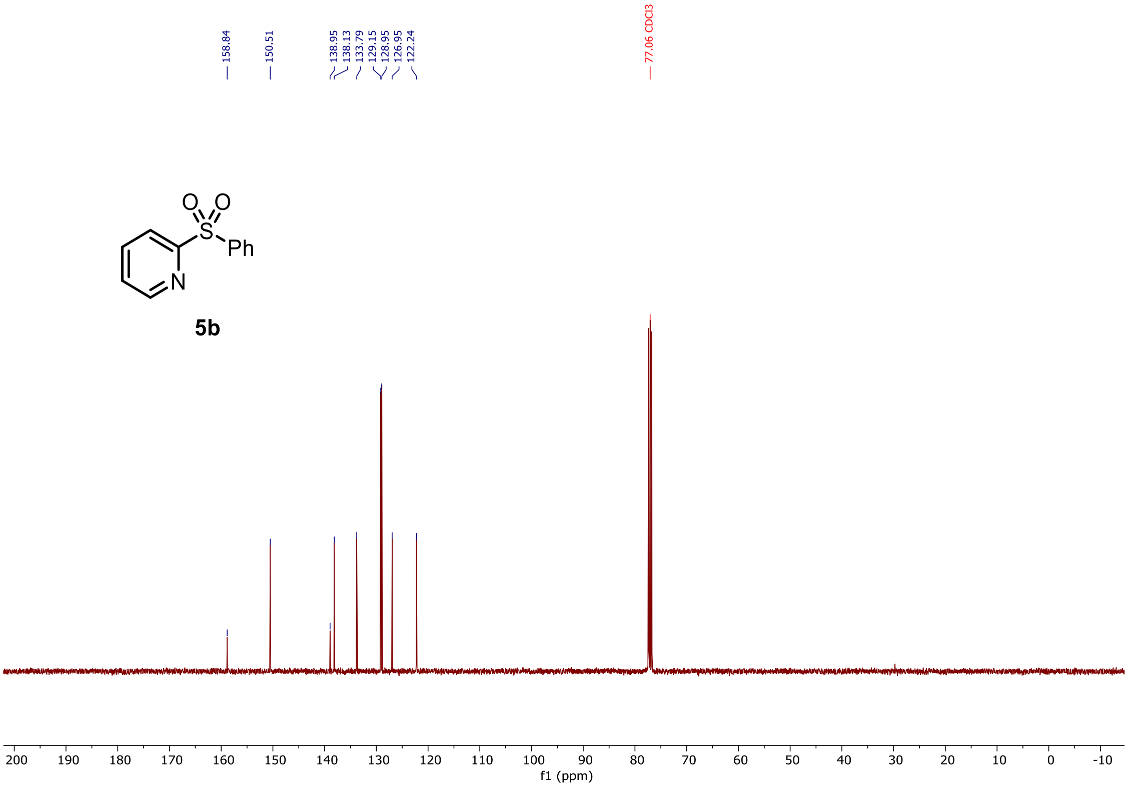

^{1}H NMR (500 MHz, $CDCl_3$) of **5c**

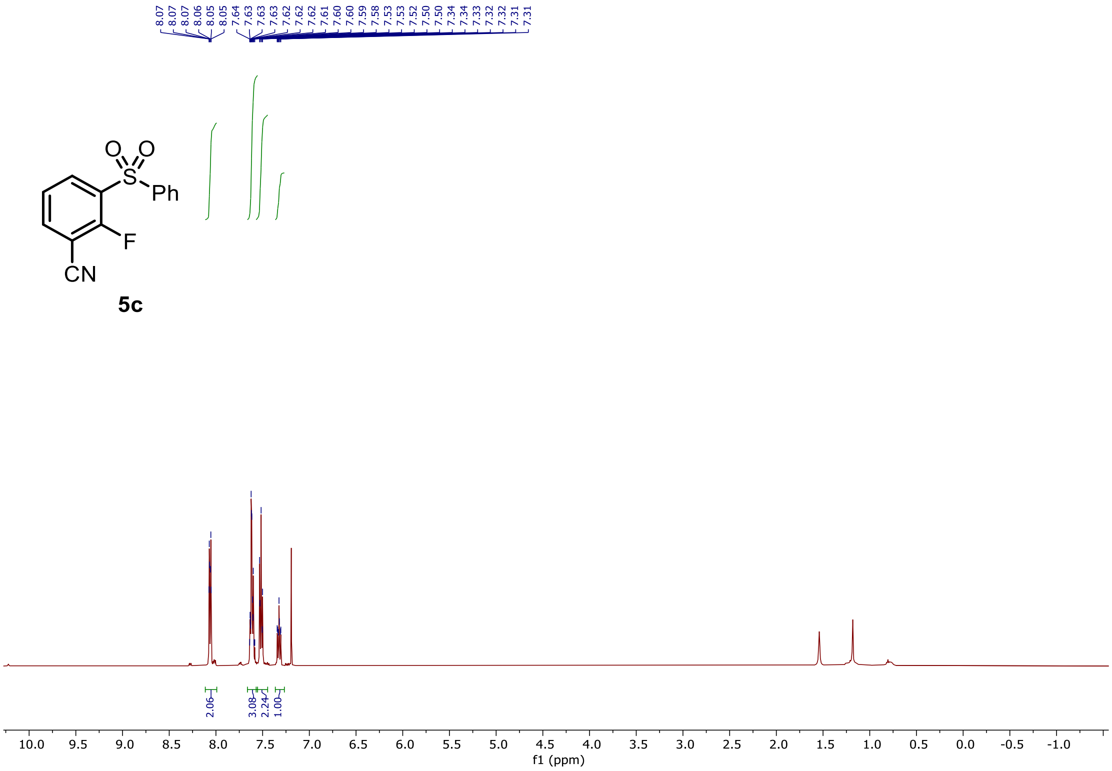

^{13}C NMR (126 MHz, $CDCl_3$) of **5c**

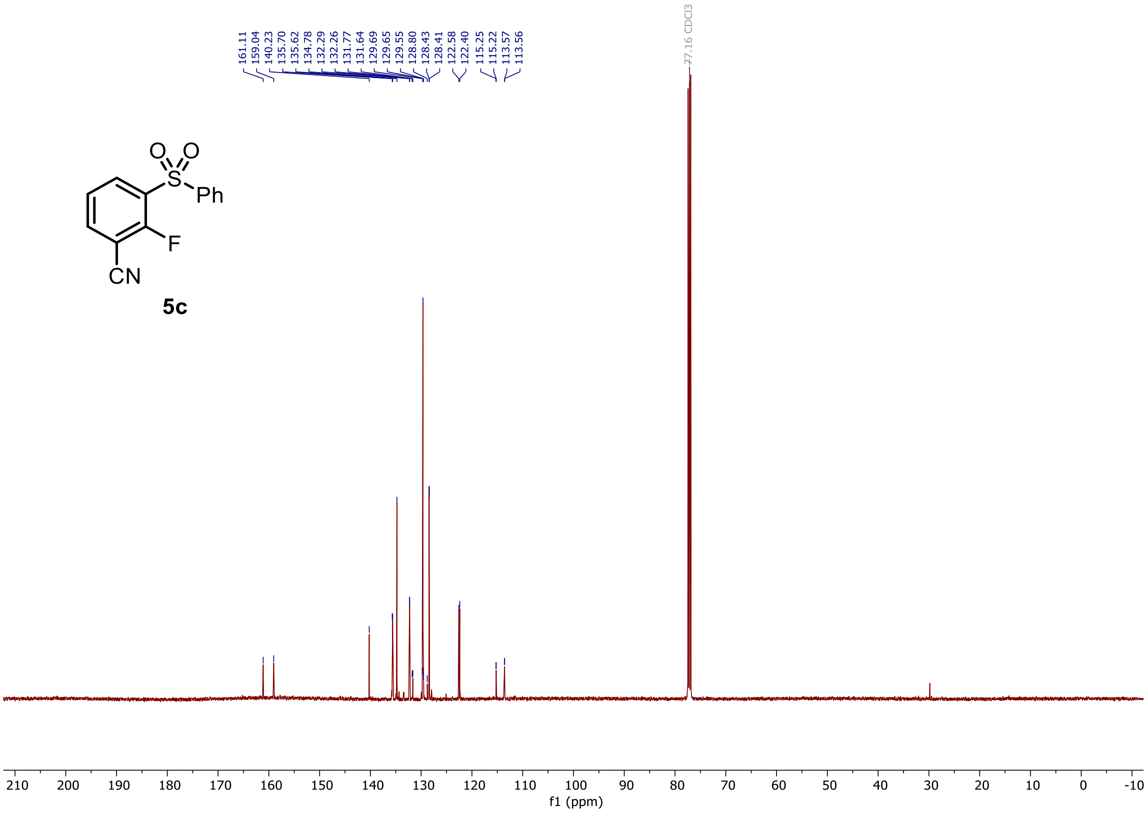

^{19}F NMR (376 MHz, $CDCl_3$) of **5c**

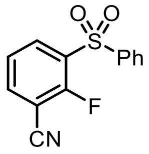

5c

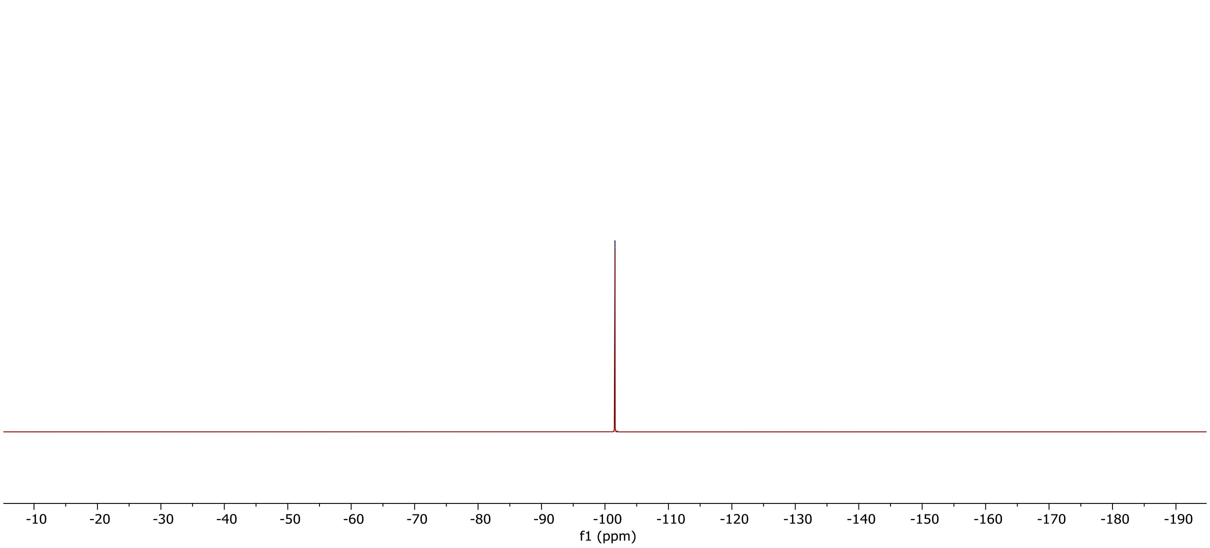

^1H NMR (400 MHz, $CDCl_3$) of **5d**

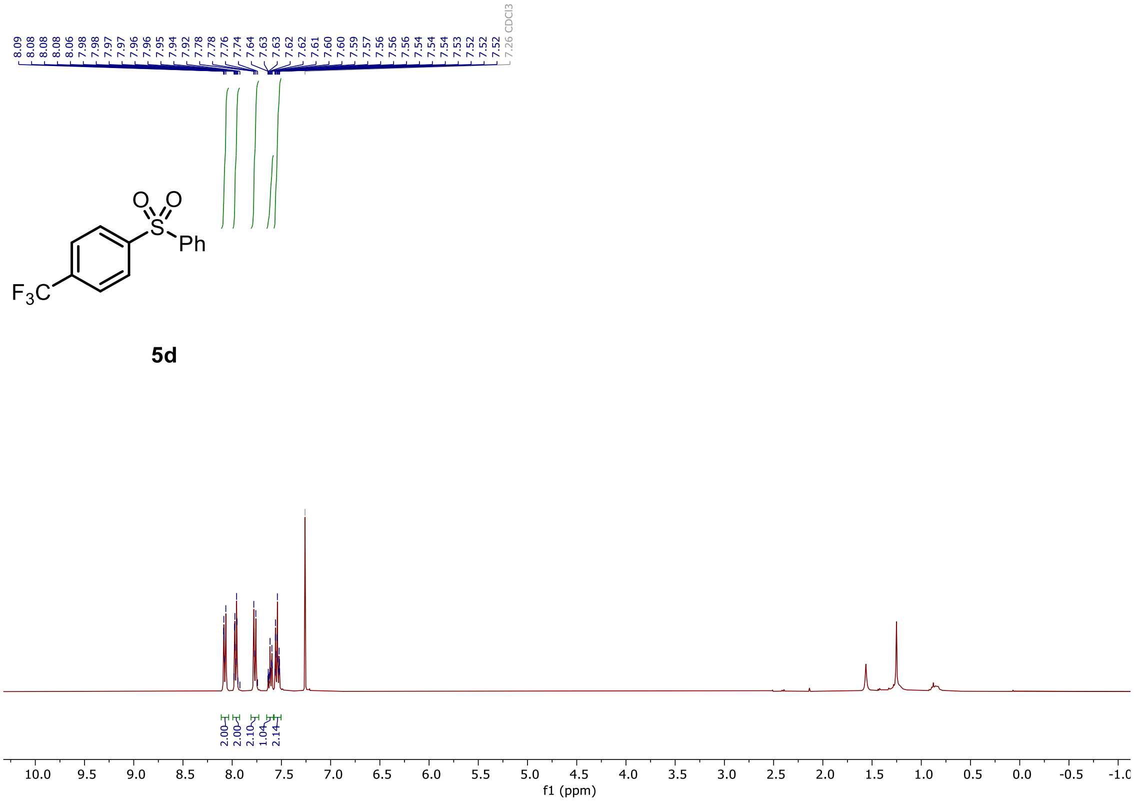

^{13}C NMR (101 MHz, $CDCl_3$) of **5d**

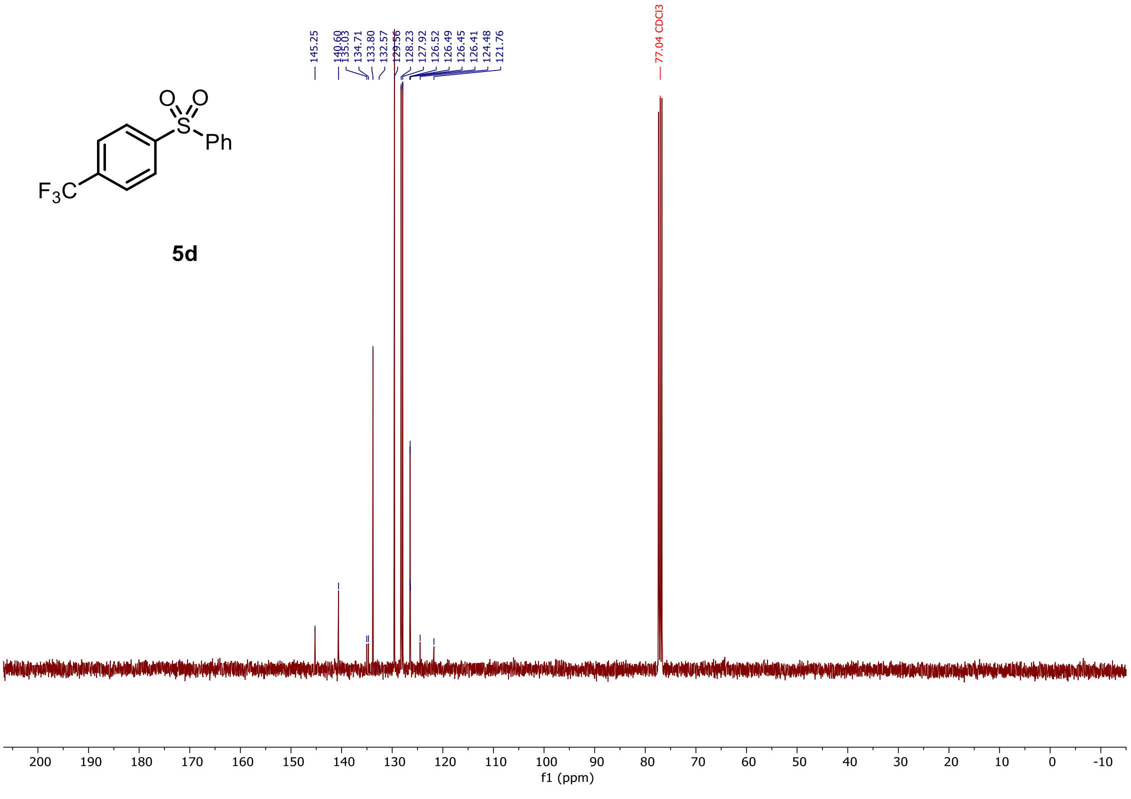

^{19}F NMR (376 MHz, $CDCl_3$) of **5d**

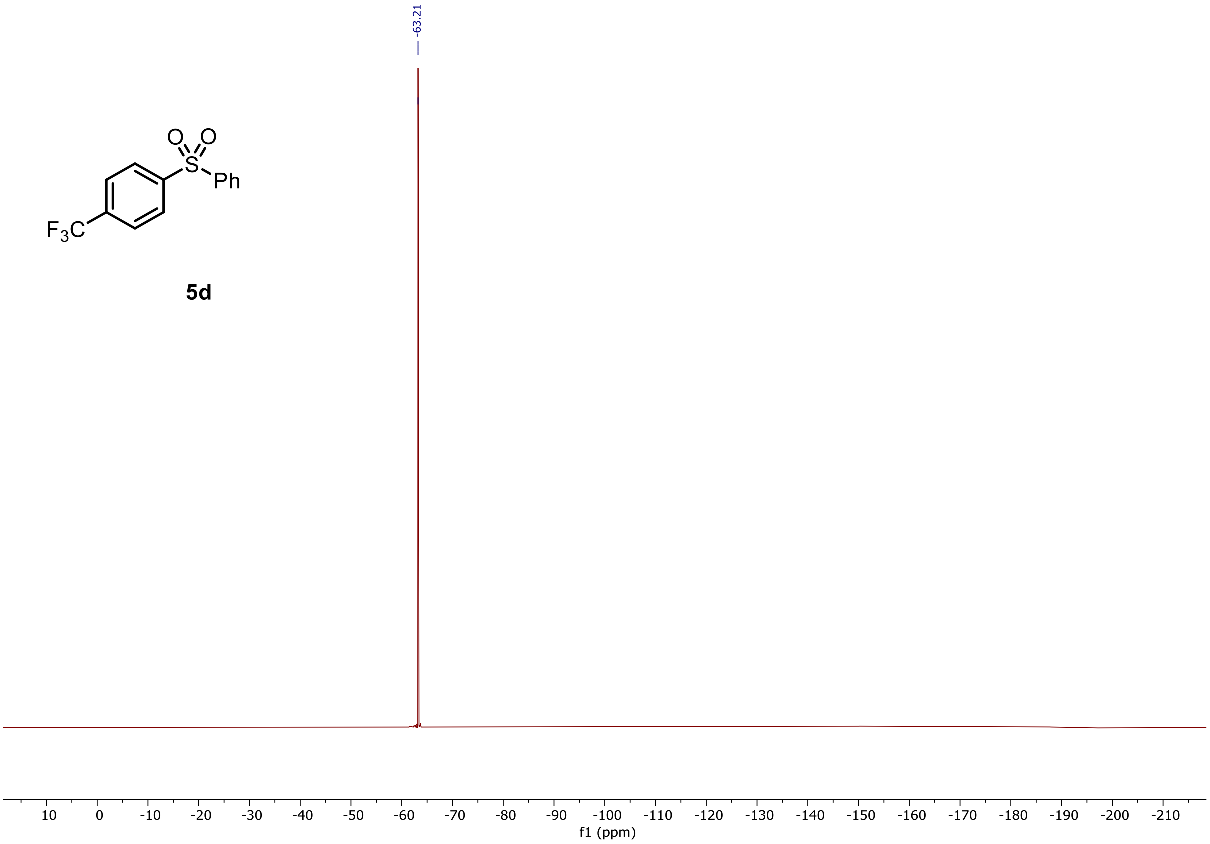

^{1}H NMR (500 MHz, CDCl$_3$) of **5e**

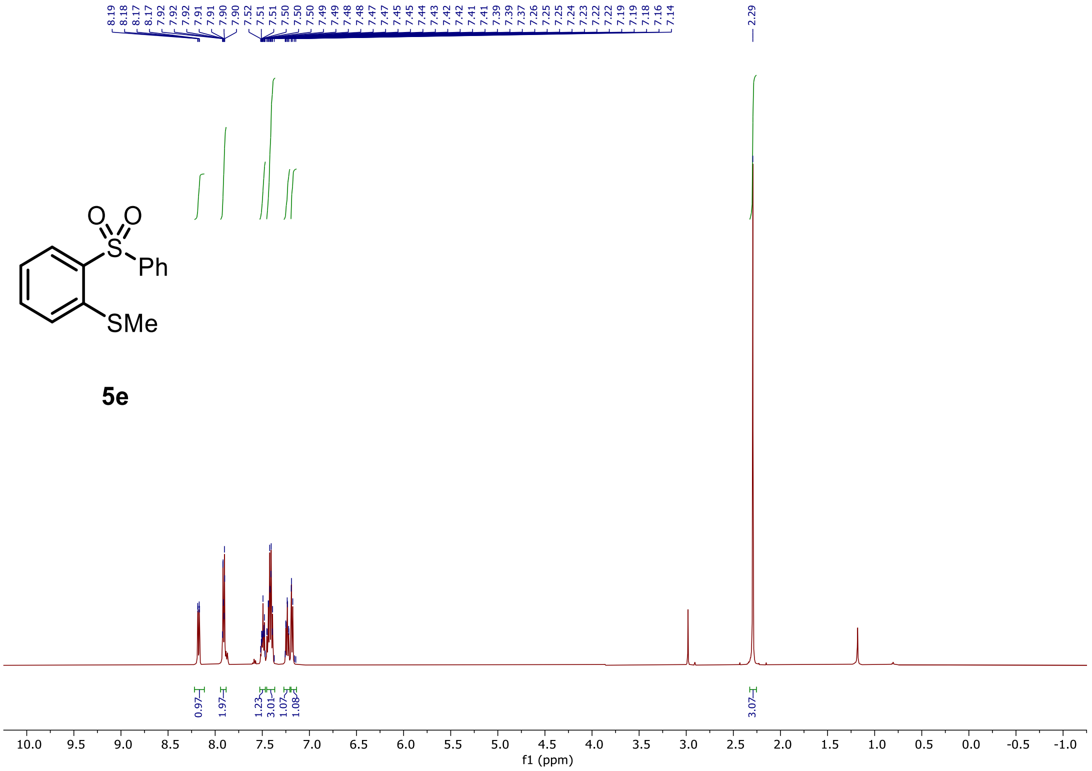

^{13}C NMR (126 MHz, CDCl$_3$) of **5e**

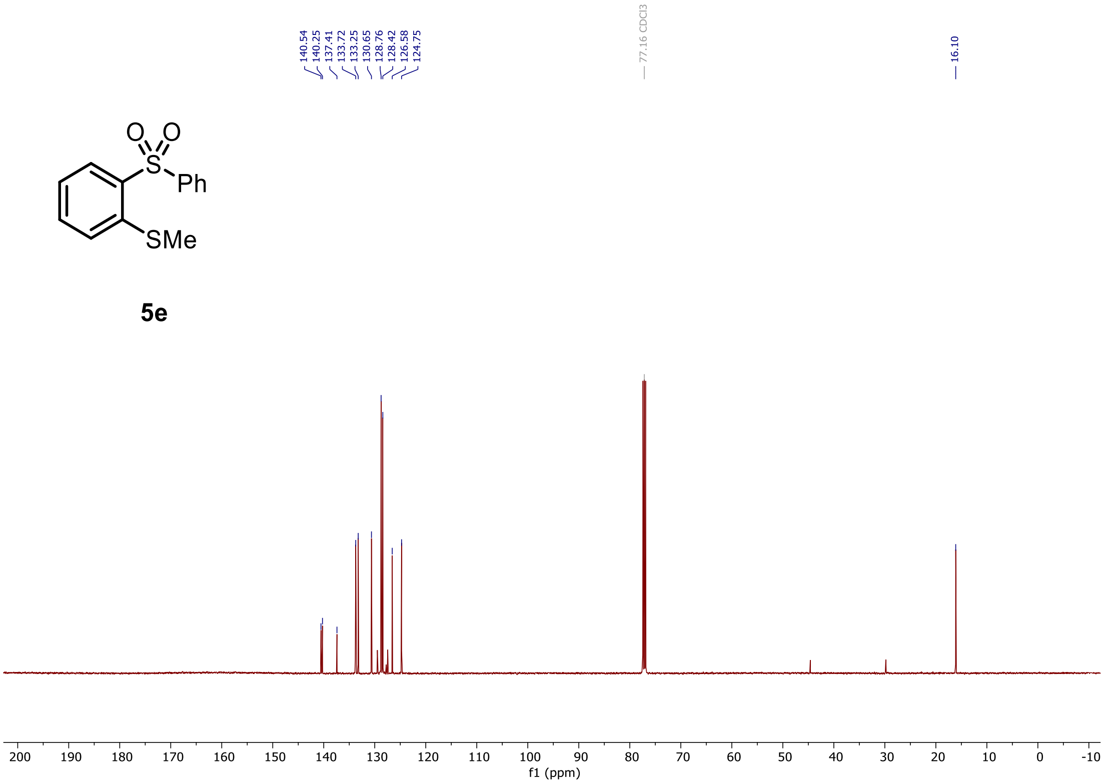

^{1}H NMR (400 MHz, $CDCl_3$) of **5f**

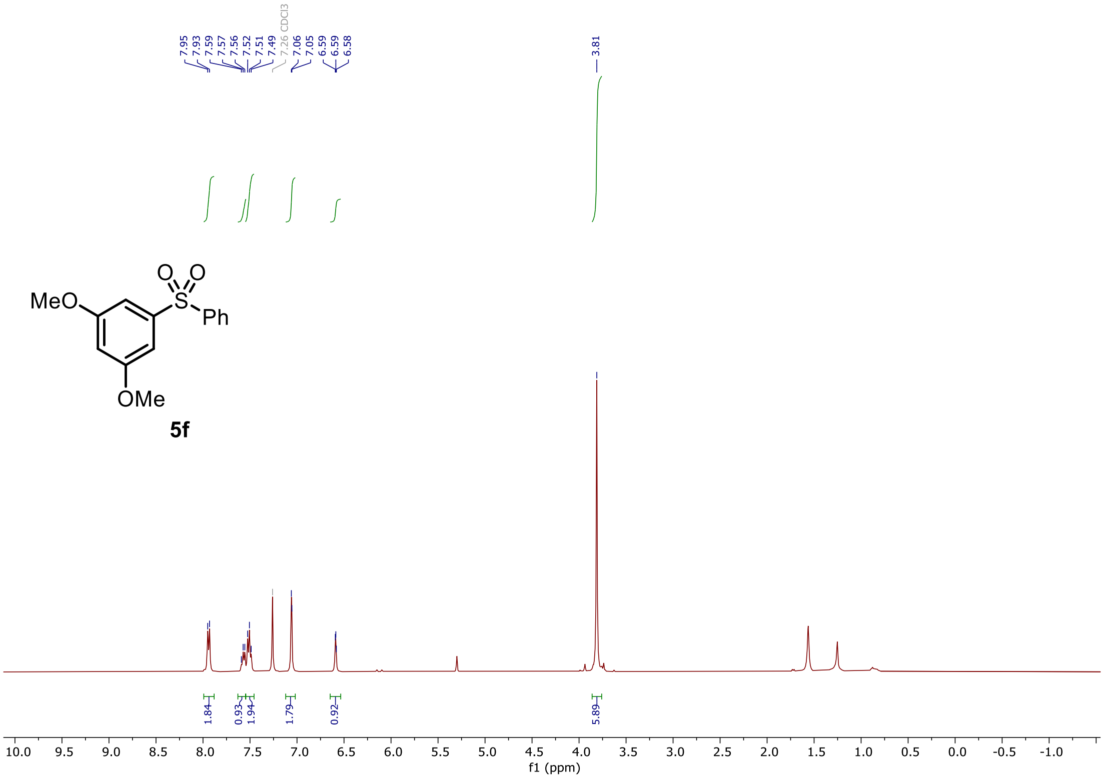

^{13}C NMR (126 MHz, $CDCl_3$) of **5f**

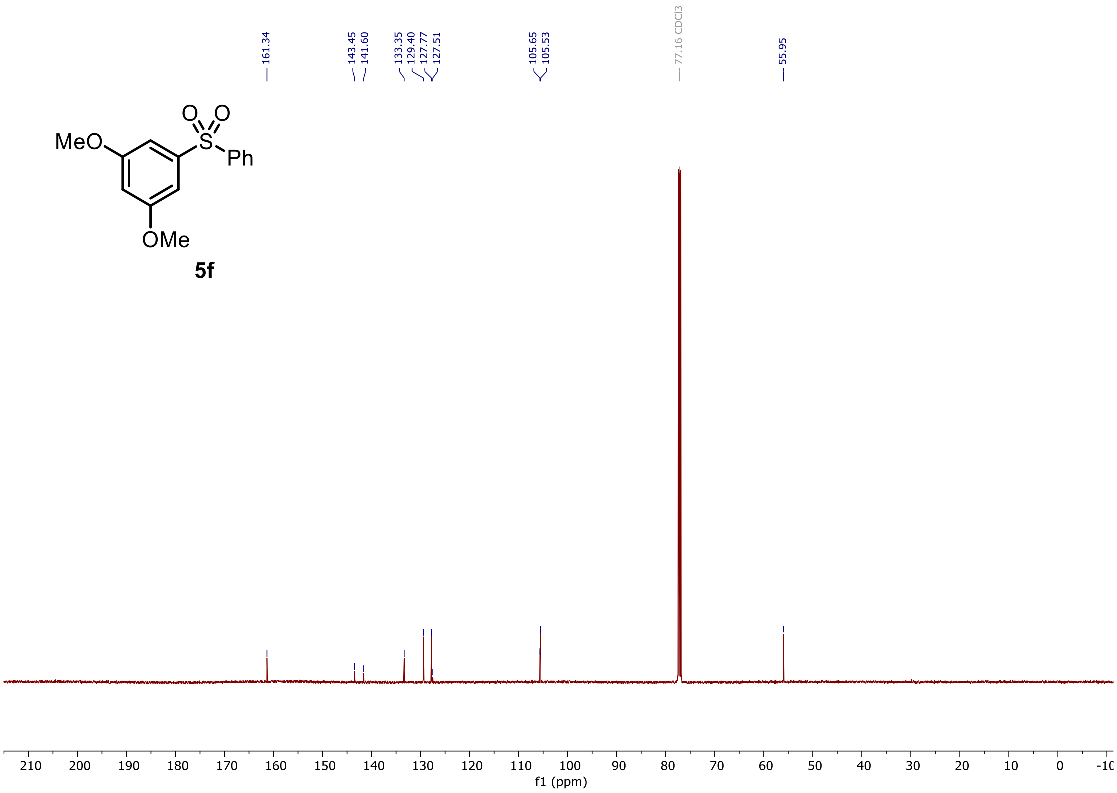

^{1}H NMR (400 MHz, $CDCl_3$) of **6a**

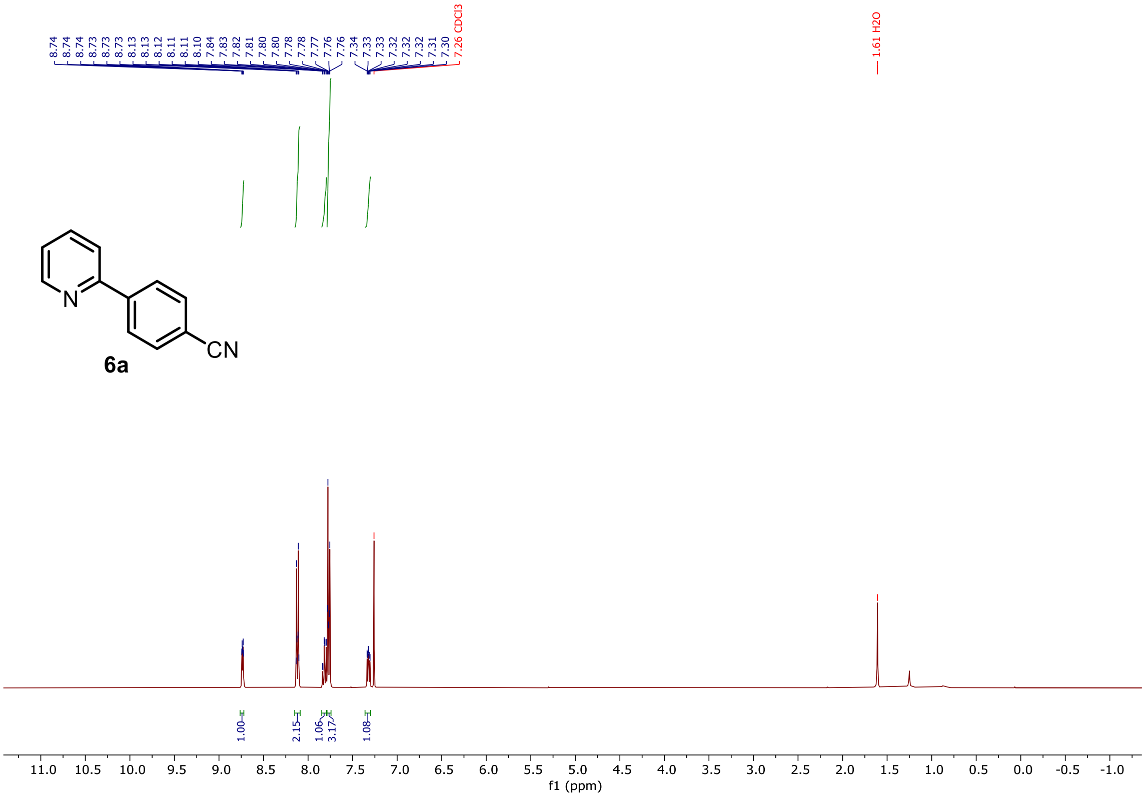

^{13}C NMR (126 MHz, $CDCl_3$) of **6a**

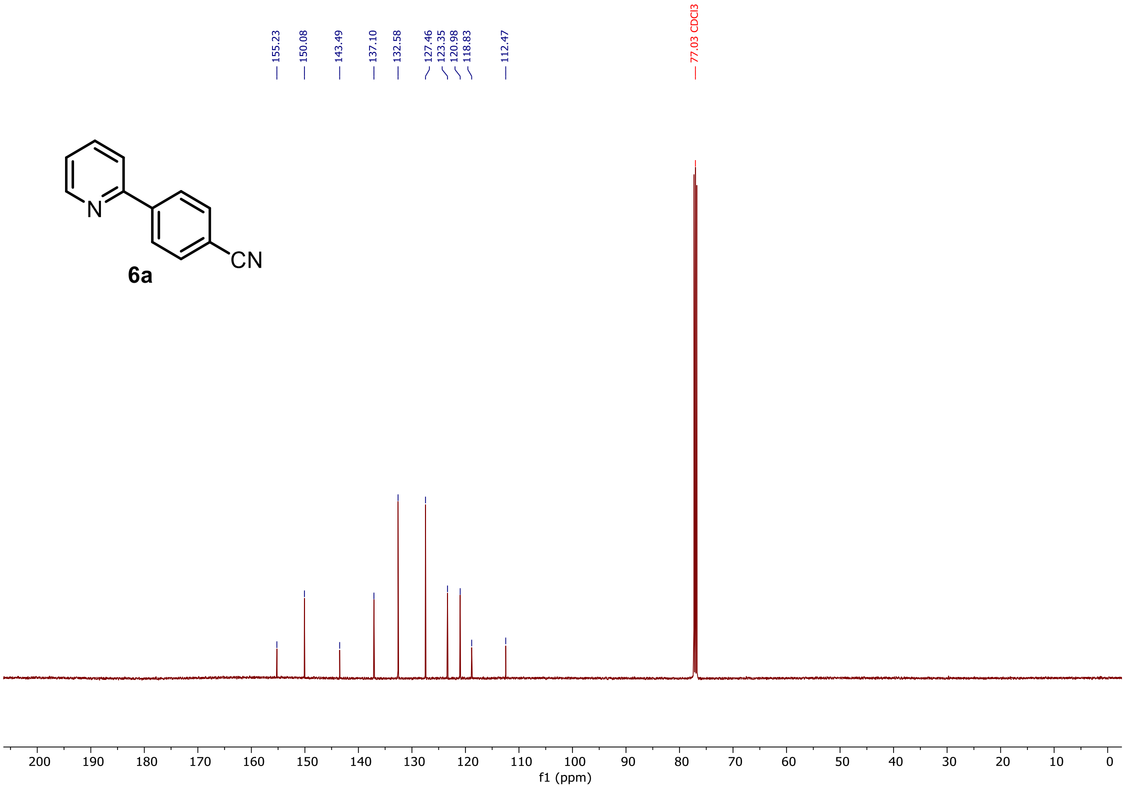

^1H NMR (500 MHz, $CDCl_3$) of **6b**

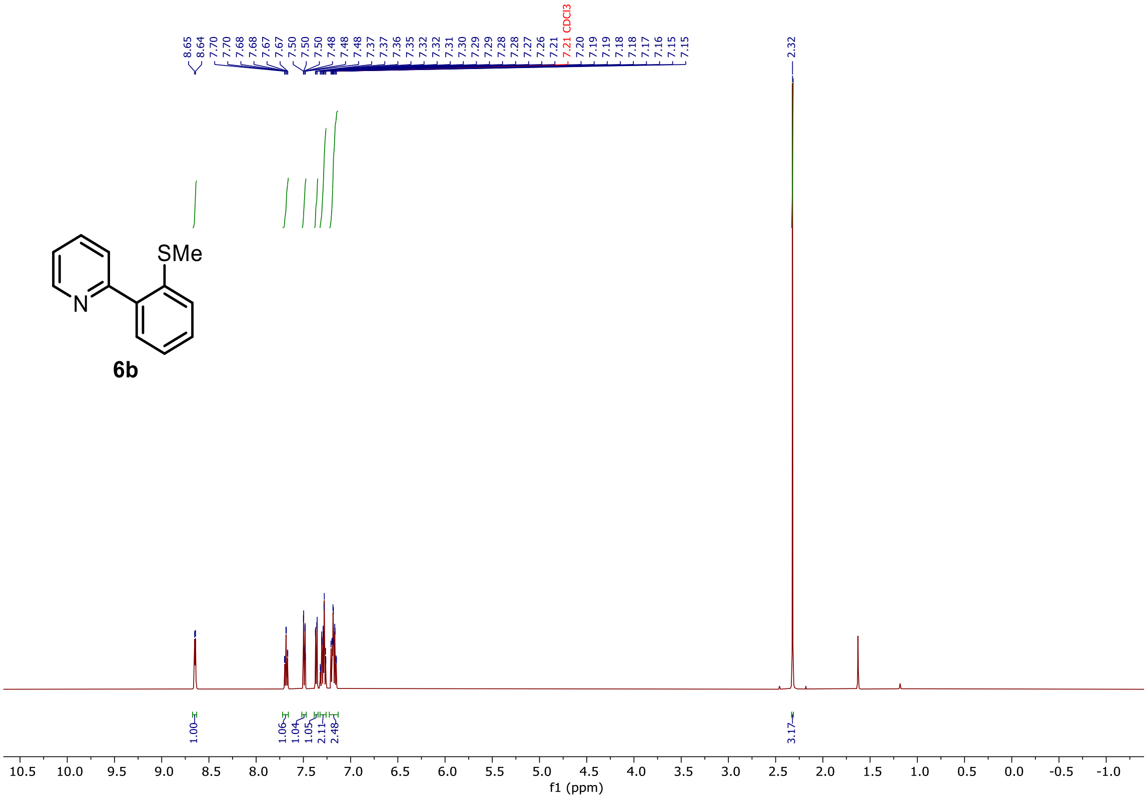

^{13}C NMR (126 MHz, $CDCl_3$) of **6b**

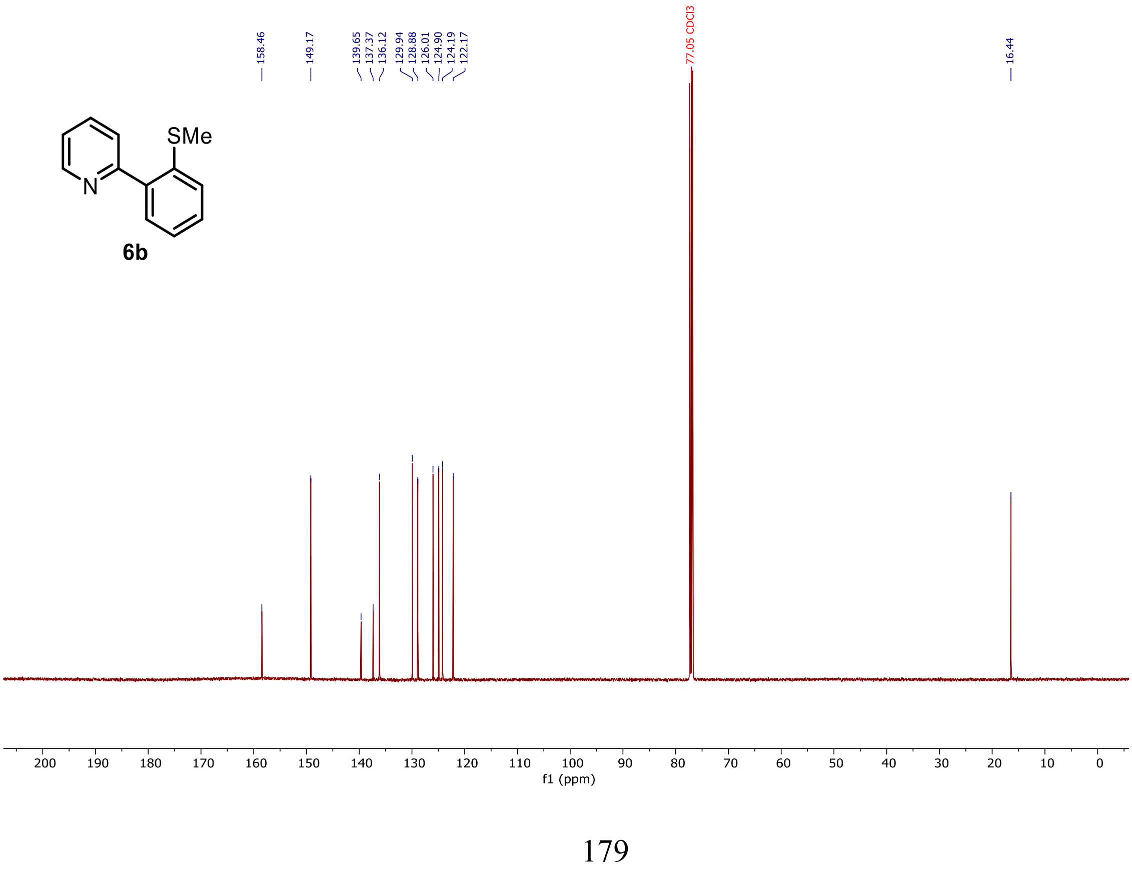

^{1}H NMR (400 MHz, $CDCl_3$) of **6c**

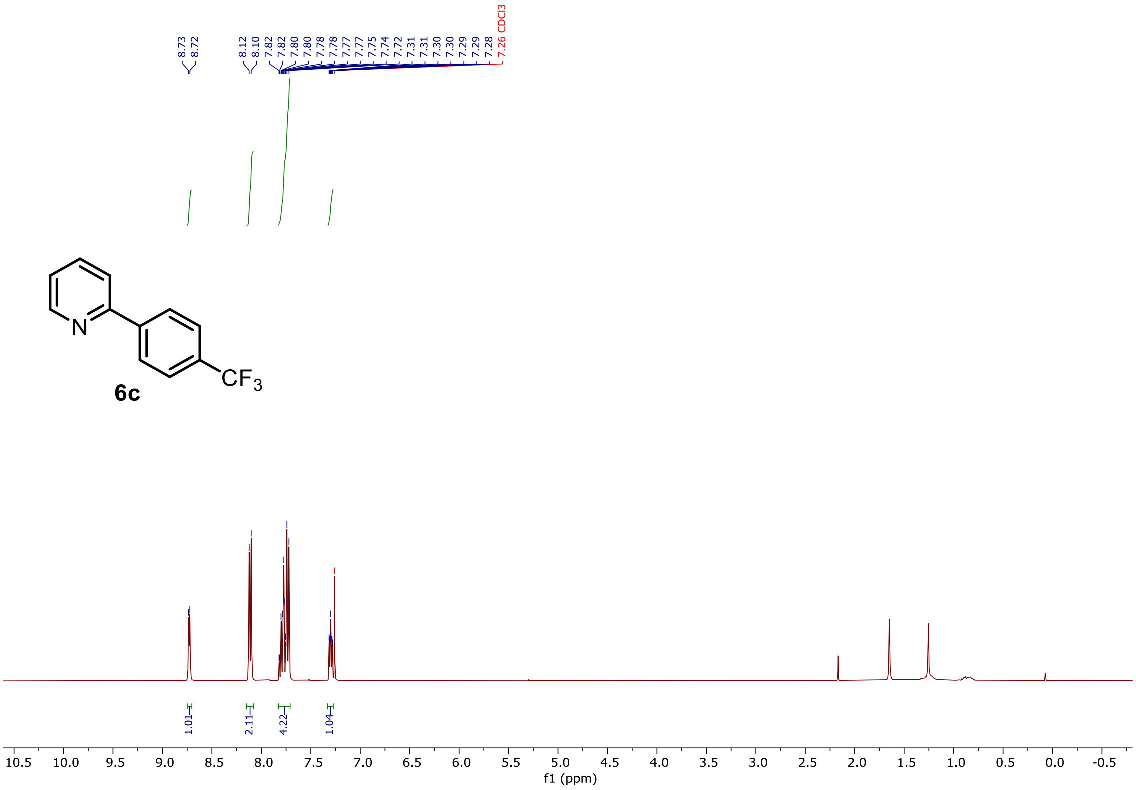

^{13}C NMR (101 MHz, $CDCl_3$) of **6c**

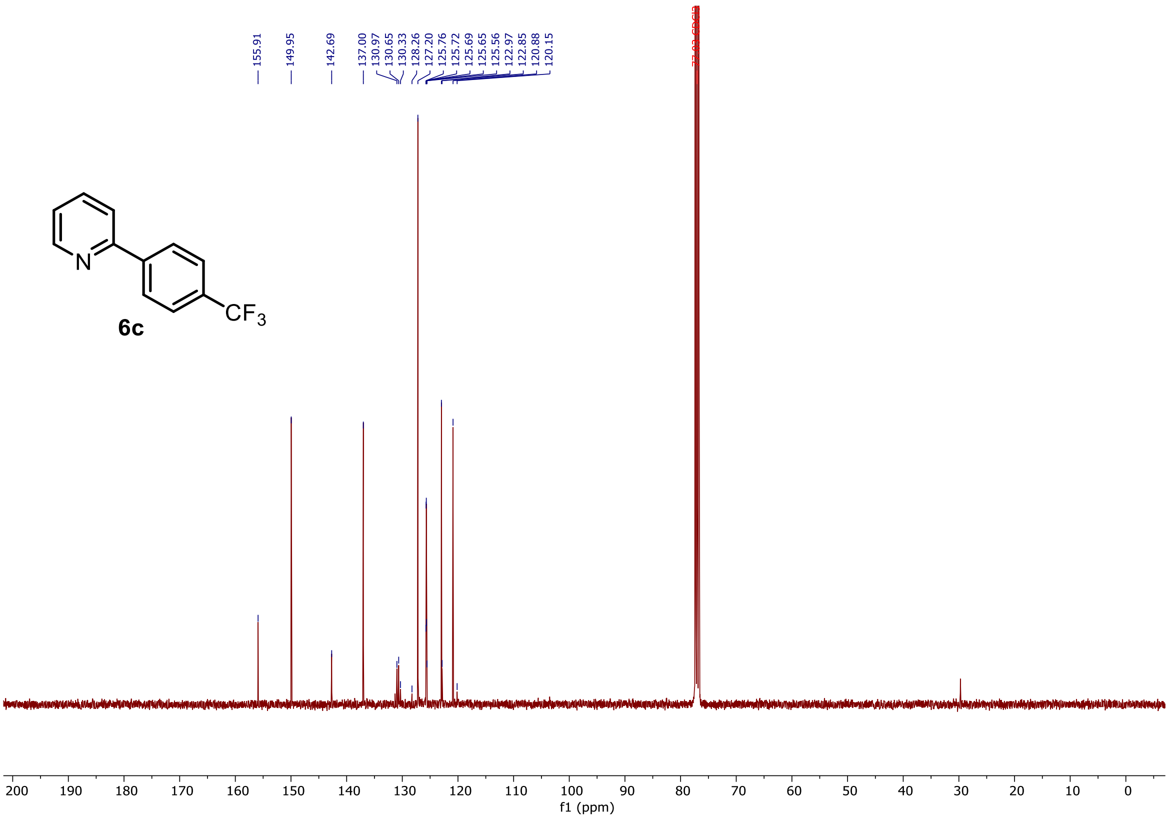

^{19}F NMR (376 MHz, $CDCl_3$) of **6c**

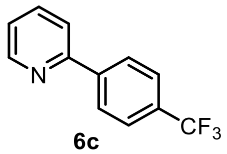

6c

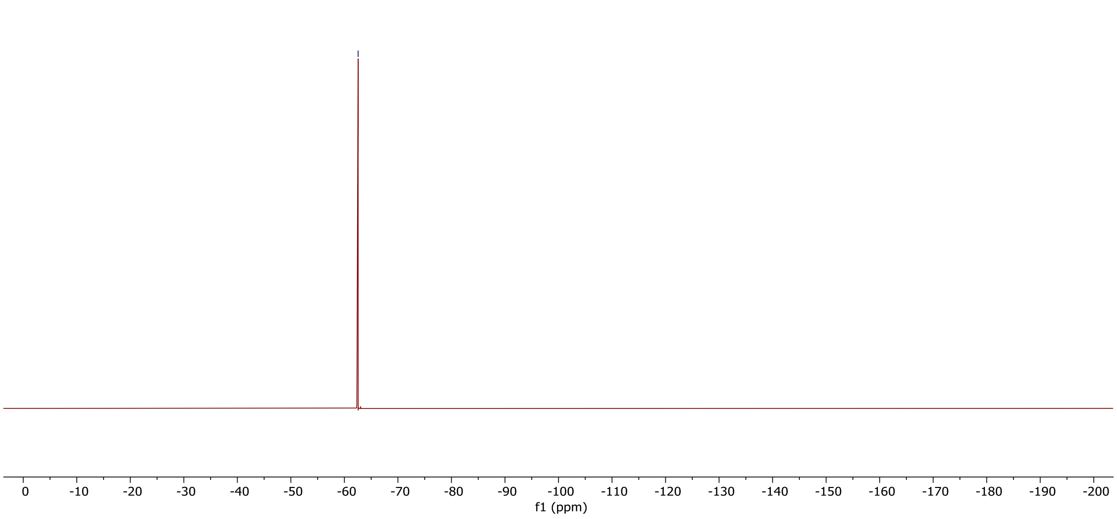

^{1}H NMR (400 MHz, $CDCl_3$) of **6d**

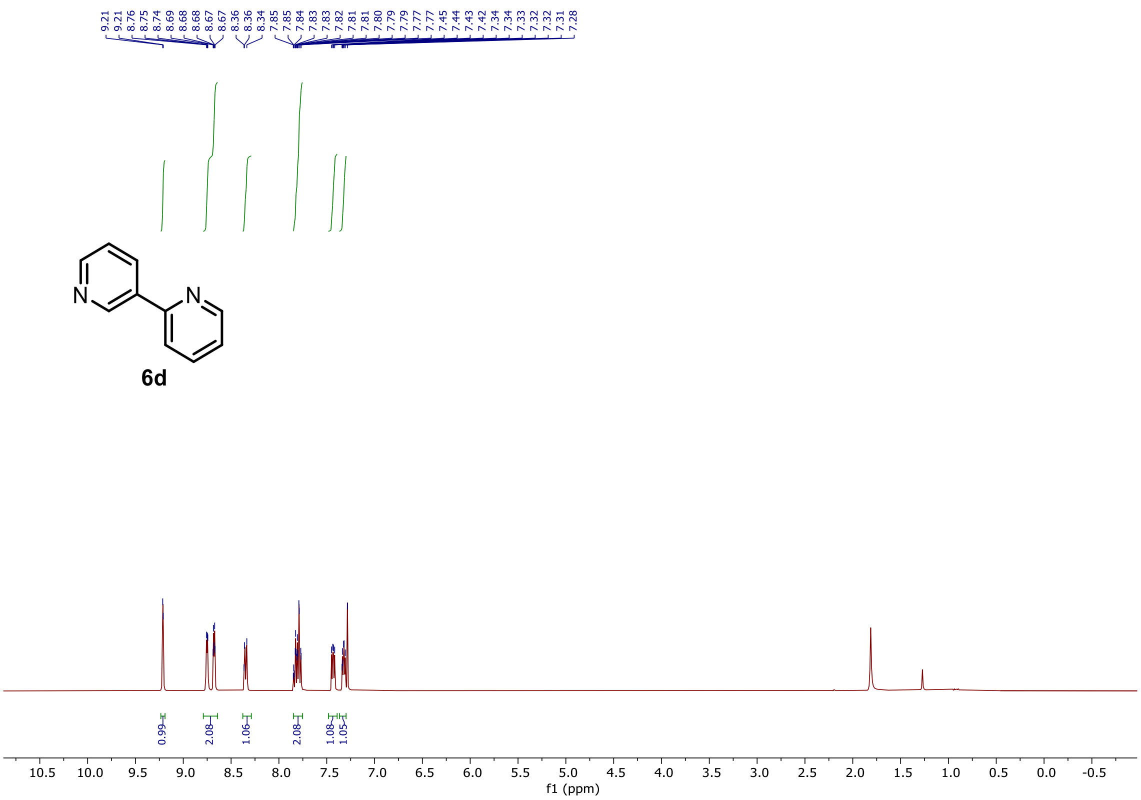

^{13}C NMR (101 MHz, $CDCl_3$) of **6d**

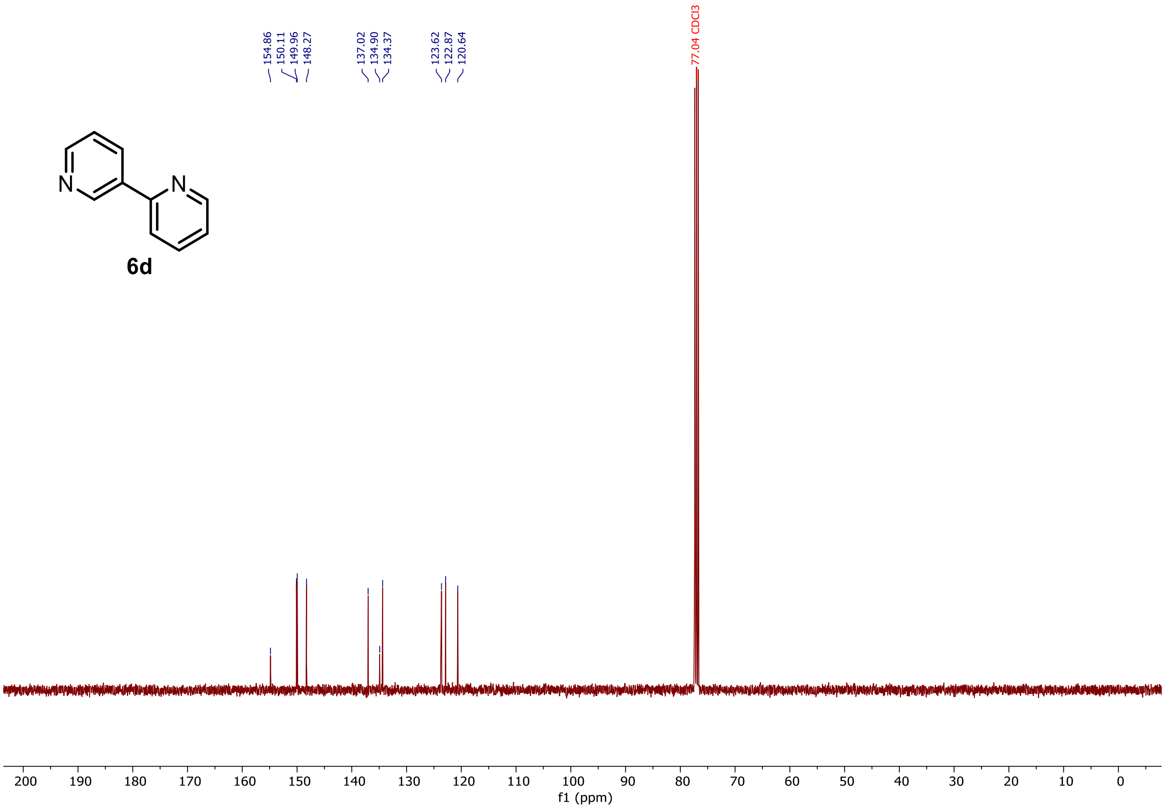

^{1}H NMR (400 MHz, $CDCl_3$) of **6e**

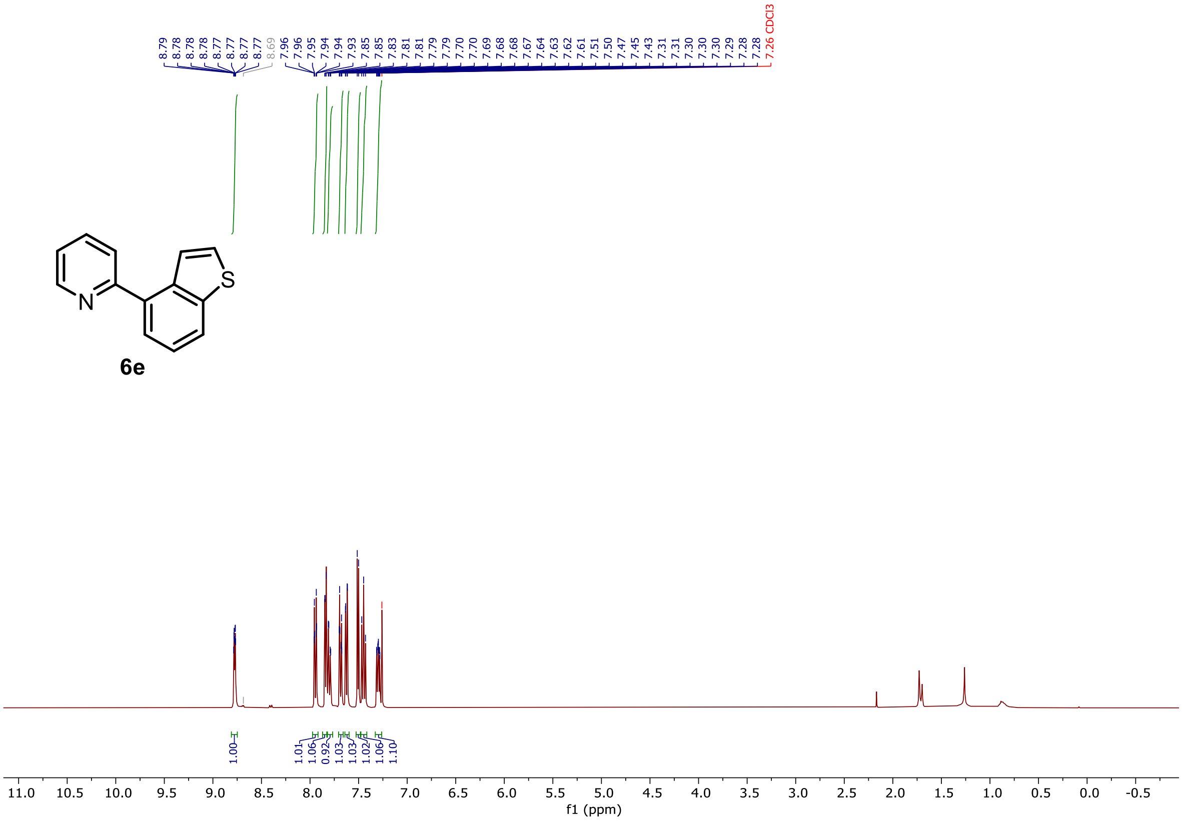

^{13}C NMR (101 MHz, $CDCl_3$) of **6e**

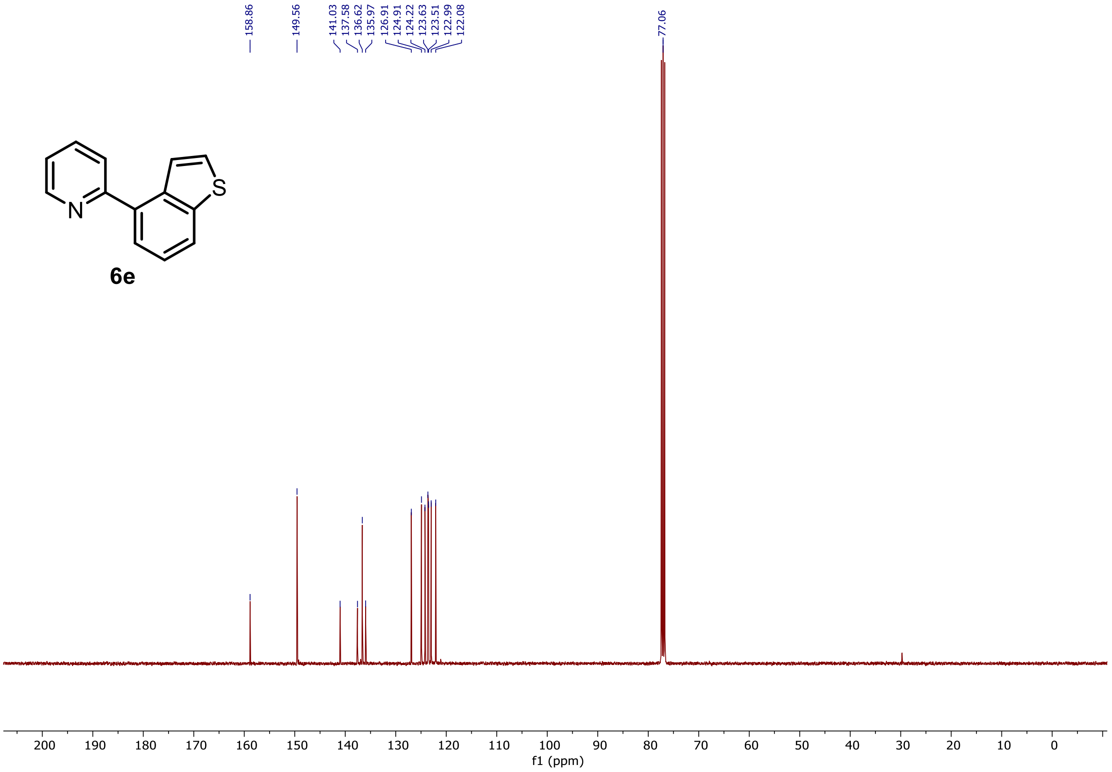

^{1}H NMR (500 MHz, $CDCl_3$) of **6f**

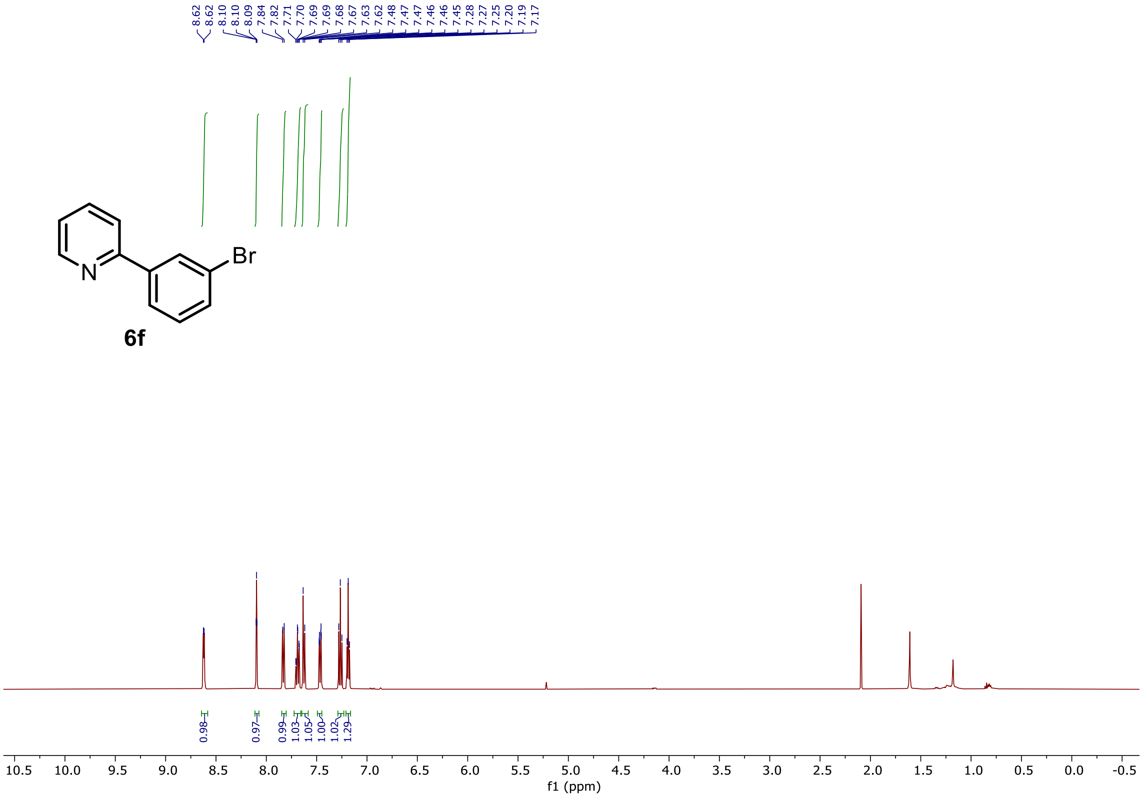

^{13}C NMR (126 MHz, $CDCl_3$) of **6f**

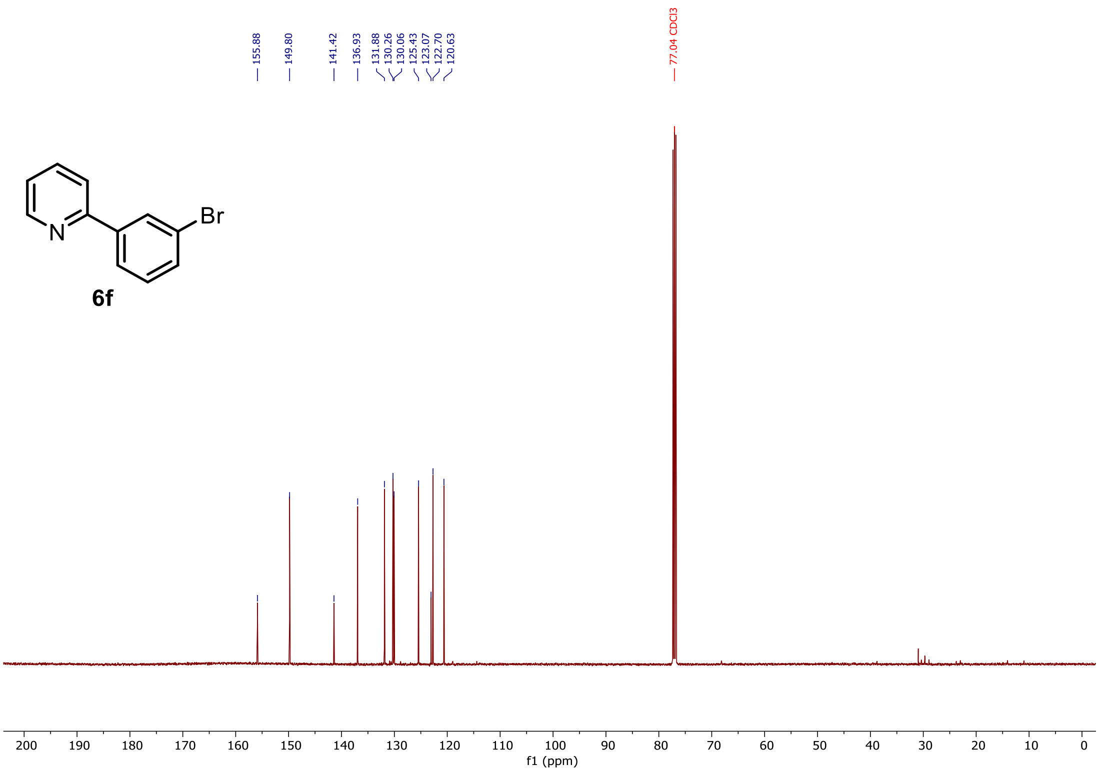

^{1}H NMR (400 MHz, $CDCl_3$) of **6g**

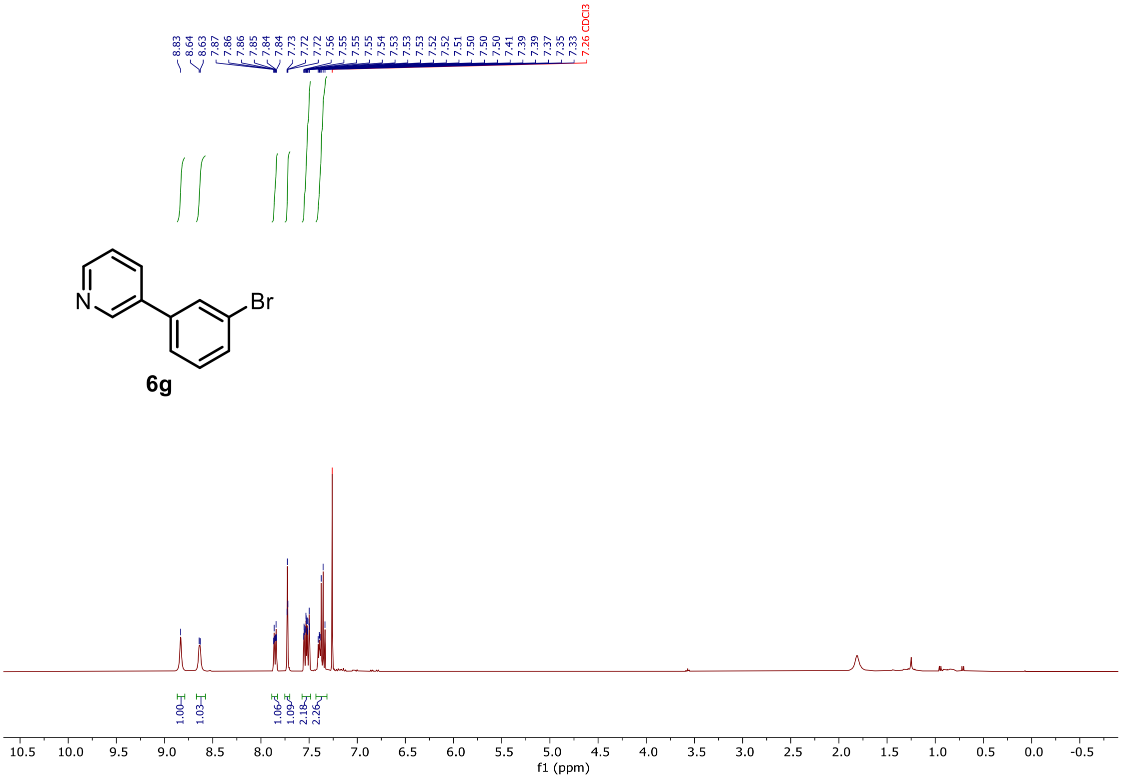

^{13}C NMR (101 MHz, $CDCl_3$) of **6g**

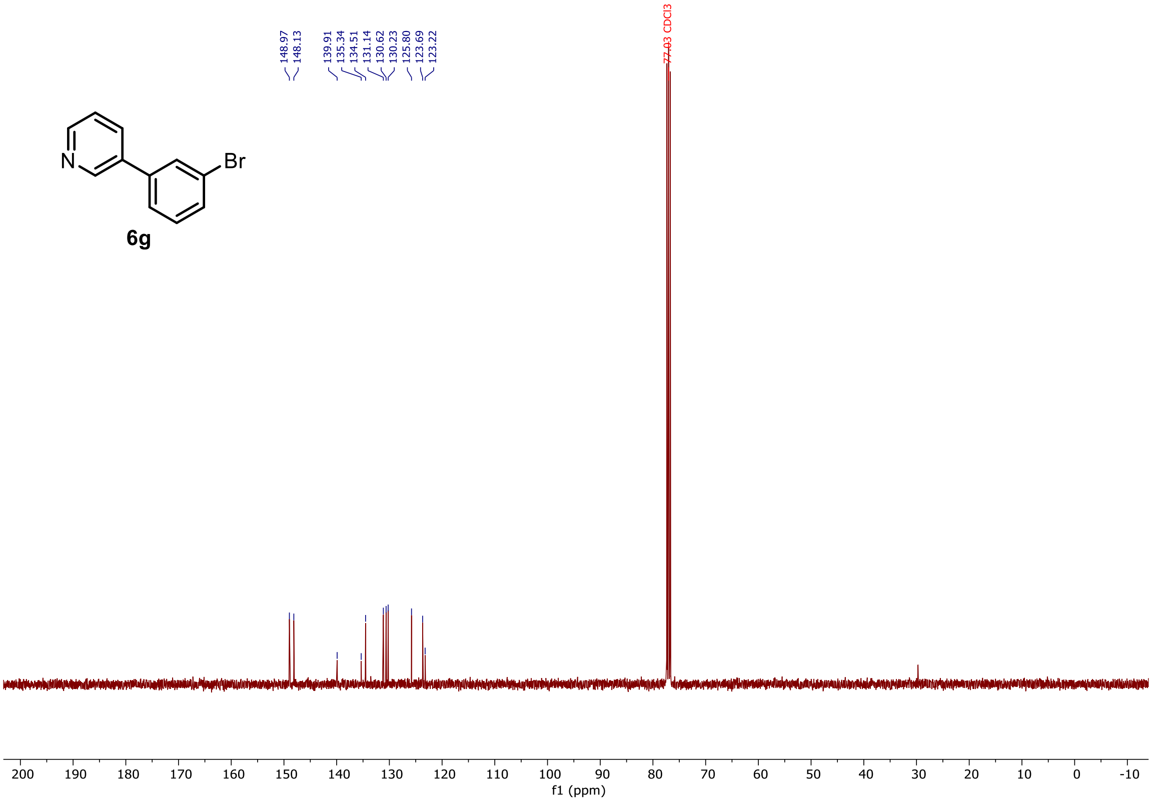

^{1}H NMR (400 MHz, $CDCl_3$) of **S6**

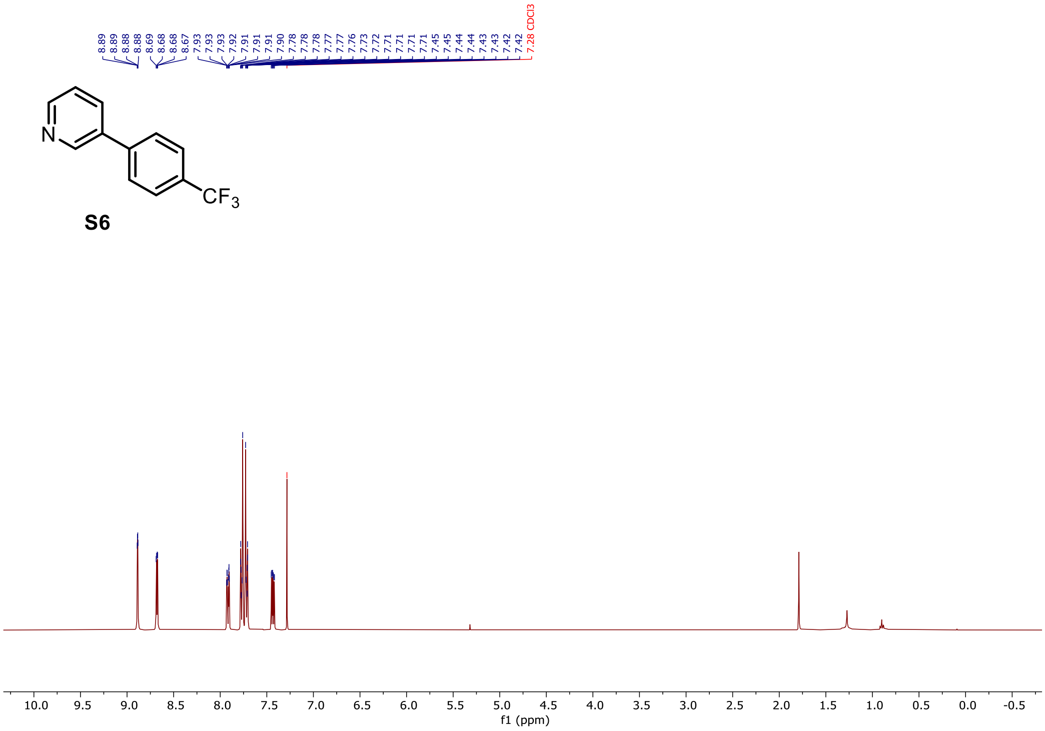

^{13}C NMR (101 MHz, $CDCl_3$) of **S6**

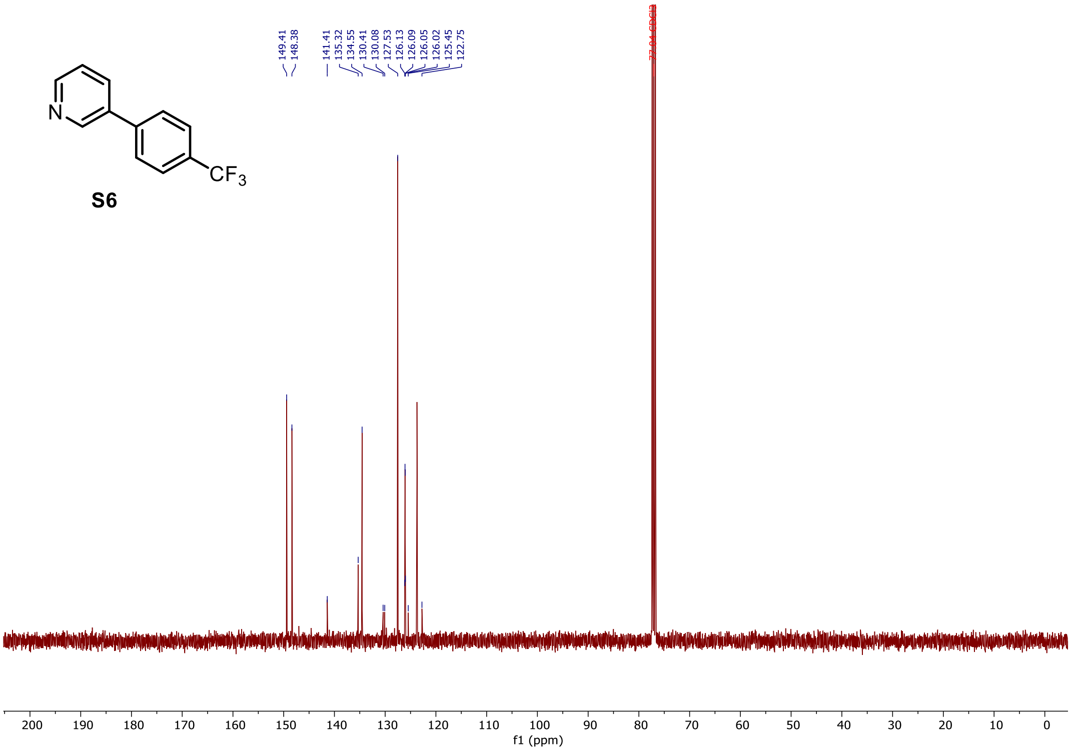

^{19}F NMR (376 MHz, $CDCl_3$) of **S6**

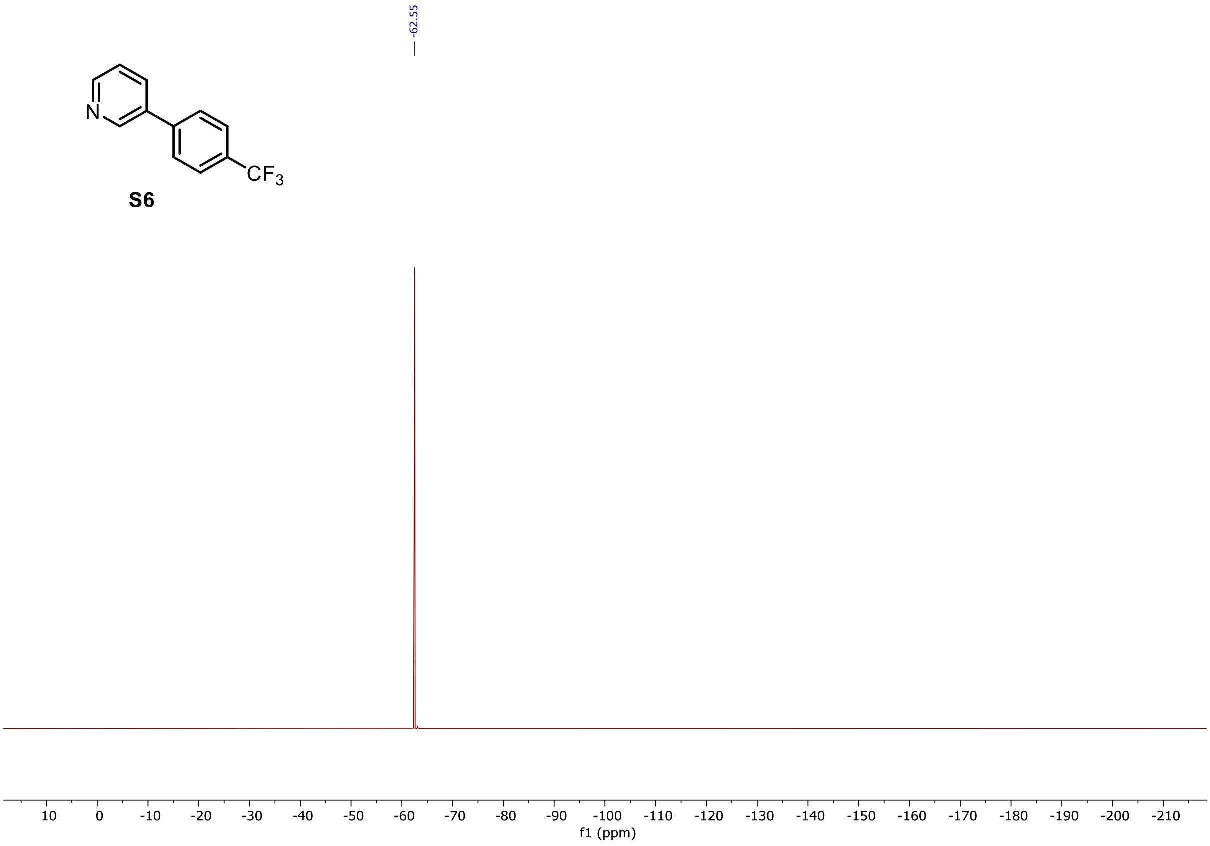

16. References

[1] Z. He, A. P. Pulis, D. J. Procter, *Angew. Chem. Int. Ed.* **2019**, *58*, 7813–7817.

[2] M. Šiaučiulis, N. Ahlsten, A. P. Pulis, D. J. Procter, *Angew. Chem. Int. Ed.* **2019**, *58*, 8779–8783.

[3] H. Marom, S. Antonov, Y. Popowski, M. Gozin, *J. Org. Chem.* **2011**, *76*, 5240–5246.

[4] S.Sau, S. Takizawa, H.Y. Kim, K. Oh. *Org. Lett.* **2024**, *26*, 8821–8826.

[5] K. K. Andersen, M. Cinquini, N. E. Papanikolaou, *J. Org. Chem.* **1970**, *35*, 706

[6] R. M. Acheson, J. K. Stubbs, *J. Chem. Soc., Perkin Trans I*, **1972**, 899

[7] R. Matsubara, T. Yabuta, U. Md Idros, M. Hayashi, F. Ema, Y. Kobori, K. Sakata, *J. Org. Chem.* **2018**, *83*, 9381–9390.

[8] H. Hayashi, B. Wang, X. Wu, S. J. Teo, A. Kaga, K. Watanabe, R. Takita, E. K. L. Yeow, S. Chiba, *Adv. Synth. Catal.* **2020**, *362*, 2223–2231.

[9] A. Dewanji, L. van Dalsen, J. A. Rossi-Ashton, E. Gasson, G. E. M. Crisenza, D. J. Procter, *Nat. Chem.* **2023**, *15*, 43–52.

[10] Quillen, Q. Nguyen, M. Neiser, K. Lindsay, A. Rosen, S. Ramirez, S. Costan, N. Johnson, T. D. Do, O. Rodriguez, D. Rivera, A. Atesin, T. A. Ateşin, L. Ma, *J. Org. Chem.* **2019**, *84*, 7652–7663.

[11] V. A. Ionova, A. S. Abel, A. D. Averin, I. P. Beletskaya, *Adv. Synth. Catal.* **2024**, *366*, 3173–3180.

[12] C. Cavedon, S. Gisbertz, S. Reischauer, S. Vogl, E. Sperlich, J. H. Burke, R. F. Wallick, S. Schrottke, W.-H. Hsu, L. Anghileri, Y. Pfeifer, N. Richter, C. Teutloff, H. Müller-Werkmeister, D. Cambié, P. H. Seeberger, J. Vura-Weis, R. M. van der Veen, A. Thomas, B. Pieber, *Angew. Chem. Int. Ed.* **2022**, *61*, e202211433.

[13] V. G. Pandya, S. B. Mhaske, *Org. Lett.* **2014**, *16*, 3836–3839.

[14] Y. Li, W. Liu, C. Kuang, *Chem. Commun.,* **2014**, *50*, 7124-7127.

[15] P. Sharma, S. Rohilla, N. Jain, *J. Org. Chem*. **2015**, *80*, 4116–4122.

[16] J. Kan, S. Huang, J.Lin, M. Zhang, W. Su, *Angew. Chem. Int. Ed.* **2015**, *54*, 2199 – 2203.

[17] A. M. Horan, V. K. Duong, E. M. McGarrigle, *Org. Lett.* **2021**, *23*, 9089–9093.

[18] Y. Jin, H. Kim, Y. Kwan, J. Lee, J.-H, Sohn, *Org. Lett.* **2025***, 27,* 2930−2935.
